# Characterisation of gas cell reactions for 70+ elements using N_2_O for ICP tandem mass spectrometry measurements†

**DOI:** 10.1039/d3ja00025g

**Published:** 2023-04-28

**Authors:** Shaun T. Lancaster, Thomas Prohaska, Johanna Irrgeher

**Affiliations:** a Department of General, Analytical and Physical Chemistry, Chair of General and Analytical Chemistry, Montanuniversität Leoben Leoben Austria shaun.lancaster@unileoben.ac.at; b Department of Physics and Astronomy, University of Calgary Calgary Canada

## Abstract

One widely utilised method to reduce spectral interferences for measurements using inductively coupled plasma mass spectrometry (ICP-MS) is to employ the use of a reaction cell gas. Nitrous oxide (N_2_O) is a highly reactive gas typically used for mass-shifting only target analytes to a higher mass-to-charge ratio with increased sensitivity (*e.g.* +16, +32, +48 amu for monoxide, dioxide, and trioxide product ions respectively). Traditionally, the use of N_2_O was limited to selected applications due to the creation of new interferences that also interfere with the detected masses of interest. However, with the advent of inductively coupled plasma tandem mass spectrometry (ICP-MS/MS), the use of N_2_O has gained more traction, with a growing number of publications in recent years. Here, a comprehensive study of the use of N_2_O for the determination of 73 elements has been conducted, with a comparison to the most widely used mass-shift method using oxygen (O_2_) as a reaction gas. In total, 59 elements showed improved sensitivity when performing mass-shift with N_2_O compared to O_2_, with 8 elements showing no reaction with either gas. Additionally, N_2_O demonstrated a collisional focusing effect for 36 elements when measuring on-mass. This effect was not observed using O_2_. Monitoring asymmetric charge transfer reactions with N_2_O highlighted 14 elements, primarily non-metals and semi-metals, that enter the gas cell as metastable ions and could be used as an alternative mass-shift option. The results from this study highlight the high versatility of N_2_O as a reaction cell gas for routine ICP-MS/MS measurements.

## Abstract 2

Gas cell interactions of 73 elements with nitrous oxide (N_2_O) have been evaluated using inductively coupled plasma tandem mass spectrometry (ICP-MS/MS). Recommendations for the reduction of interferences are provided.

## Graphical abstract

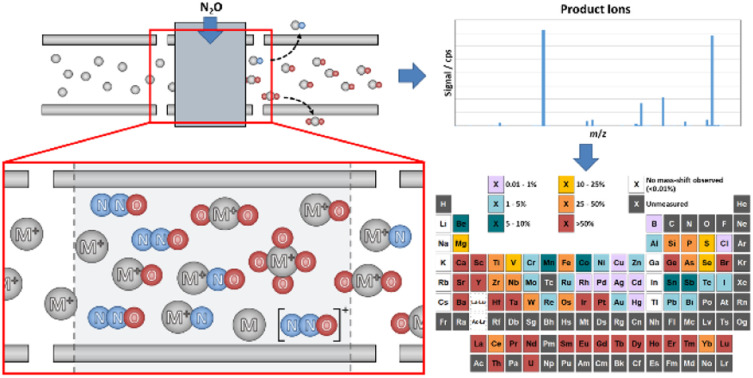

## Introduction

Overcoming spectral interferences in inductively coupled plasma mass spectrometry (ICP-MS) measurements is of utmost importance when striving to achieve reliable data. One widely utilised method to obtain interference free determinations is to employ the use of a reaction cell gas. A multitude of different gases have been used, summarised in detail previously.^1–4^ In the cell, two possible reactions can occur: atom transfer and asymmetric charge transfer. With the advent of tandem mass spectrometry (MS/MS), only interferences with the same mass-to-charge ratio (*m*/*z*) as the target analyte remain of concern as all other ions (that can potentially become interferences depending on the reactions employed) are removed by a mass filter before entering the reaction cell.

Resolution of spectral interferences by atom transfer is often achieved in two ways: atom transfer to the analyte or atom transfer to the interference. In the former case, the most common approach is to perform the reaction of the target analyte with oxygen (O_2_),^3^ such that M^+^ + O_2_ → MO^+^ + O. This is known as a “mass-shift” determination, where the analyte, M, is detected at a higher *m*/*z* (in this case +16 amu). Conversely, mass-shifting the interference allows the target analyte to be analysed at the same *m*/*z* as it enters the cell – known as an “on-mass” determination. Other gases used for atom transfer reactions include nitrous oxide (N_2_O),^5–8^ carbon dioxide,^9^ sulphur hexafluoride,^10^ and methyl fluoride.^11,12^

Asymmetric charge transfer reactions remove interferences by transferring the charge of an interference to the reaction gas (*e.g.* ammonia (NH_3_)), such that M^+^ + NH_3_ → M + NH_3_^+^. Due to the vacuum conditions of the gas cell, only exothermic reactions can proceed. Therefore, for charge transfer to occur, the ionisation energy of the reaction gas must be lower than that of the interfering ion. The use of NH_3_ is a widely-used option^2,13^ due to its low ionisation energy (10.070 eV)^14^ as well as its reactivity to form clusters with selected elements.^4^ Other gases used for charge transfer reactions include hydrogen, xenon, methane, and nitrogen.^1^

The application of a cell gas can also lead to a collisional focusing effect, first observed by Douglas and French.^15^ This effect gives an apparent increase in sensitivity with increased gas pressures due to loss of radial kinetic energy. This focuses the ions to the minimum of the effective potential of the quadrupole, allowing for increased transmission through the exit aperture.^16^ This, in turn, can lead to increased sensitivity of the target analyte. Gases that have previously demonstrated a collisional focusing effect include hydrogen,^12,17^ helium,^1,18,19^ and NH_3_.^20,21^

While the choice of reaction gas is an important consideration for removal of interferences, differences in the types of reaction cells used have shown to have a significant contribution to the removal of interferences. Koppenaal *et al.* summarized that quadrupole-based reaction cells allow for effective mass-selective ejection of unwanted precursor and product ions but lower transmission and higher scattering losses. In contrast to this, multipole-based reaction cells allow for higher transmission but lower selectivity – thus generally requiring more selective reaction gases.^1^ Therefore, although both cell designs give good analytical performance, it should be noted that differences may be seen between instruments with different types of gas cells.

While O_2_ and NH_3_ have been successfully used with ICP-MS/MS determinations, they are not without their disadvantages. NH_3_ is a corrosive and toxic gas that is not universally compatible with every gas cell and O_2_ only has a selective range of elements that react readily enough to benefit from mass-shift. N_2_O is an alternative reaction gas to form oxide species. It has a much higher reactivity than O_2_ and leads to increased sensitivity when used in ICP-MS.^22,23^ However, due to its high reactivity, a tendency to form new spectral interferences from other matrix components historically rendered N_2_O unfavourable for ICP-MS measurements. Nevertheless, with ICP-MS/MS, formations of new interferences are no longer as problematic. Thus, N_2_O becomes a more viable option and is recently gaining traction within the community.

Here, a comprehensive evaluation of N_2_O as a reaction cell gas for ICP-MS/MS measurements with a quadrupole-based reaction cell has been carried out for 73 elements. Mass-shift determinations have been compared against the most widely used alternative of O_2_ for the removal of interferences. Additionally, possibilities for on-mass determinations of these elements using N_2_O have been determined based on collisional focusing effects observed for the target analytes, as well as oxide and doubly-charged interferences. Finally, charge transfer reactions have been considered as possibilities for removal of interferences.

## Experimental

### Reagents

Nitric acid (HNO_3_, *w* = 65%, p.a. grade; Carl Roth GmbH, Karlsruhe, Germany) was purified using a sub-boiling distillation system (Savillex DST-4000, AHF Analysentechnik, Tübingen, Germany). Hydrochloric acid (HCl, *w* = 65%, p.a. grade; Carl Roth GmbH) was purified by sub-boiling (Savillex DST-1000, AHF Analysentechnik). Reagent grade I water (18.2 MΩ cm; MilliQ IQ 7000, Merck-Millipore, Darmstadt, Germany) was used for all acid dilutions. Vials and pipette tips were pre-cleaned by soaking overnight in diluted sub-boiled nitric acid (*w* = 3%) before use.

Single-element standards of beryllium (Be), boron (B), cadmium (Cd), caesium (Cs), chromium (Cr), copper (Cu), gadolinium (Gd), hafnium (Hf), indium (In), lithium (Li), magnesium (Mg), molybdenum (Mo), phosphorus (P), potassium (K), rubidium (Rb), sodium (Na), and tungsten (W) (*β* = 1000 μg mL^−1^; Certipur, Merck); cerium (Ce), dysprosium (Dy), erbium (Er), europium (Eu), germanium (Ge), holmium (Ho), lanthanum (La), lutetium (Lu), neodymium (Nd), osmium (Os), praseodymium (Pr), samarium (Sm), terbium (Tb), thulium (Tm), and ytterbium (Y) (*β* = 1000 μg mL^−1^; High Purity Standards, North Charleston, SC, USA); aluminium (Al), antimony (Sb), barium (Ba), bismuth (Bi) calcium (Ca), cobalt (Co), gold (Au), iodine (I), iron (Fe), lead (Pb), manganese (Mn), niobium (Nb), palladium (Pd), platinum (Pt), rhenium (Re), rhenium (Re), scandium (Sc), strontium (Sr), sulphur (S), tantalum (Ta), thallium (Tl), tin (Sn), titanium (Ti), zinc (Zn), zirconium (Zr), selenium (Se), and vanadium (V) (*β* = 1000 μg mL^−1^; Inorganic Ventures, Christiansburg, VA, USA); selenium (Se) and vanadium (V) (*β* = 1000 μg mL^−1^; Peak Performance, CPI international, Santa Rosa, CA, USA); nickel (Ni, *β* = 1000 μg mL^−1^; Alfa Aesar, Karlsruhe, Germany); silicon (Si, *β* = 1000 μg mL^−1^; SPEX Chemicals, Metuchen, NJ, USA); gallium (Ga) and yttrium (Y) (*β* = 10 μg mL^−1^; Elemental Scientific, Omaha, NE, USA); tellurium (Te, *β* = 10 μg mL^−1^; High Purity Standards); arsenic (As), iridium (Ir), ruthenium (Ru), and silver (Ag) (*β* = 10 μg mL^−1^; Inorganic Ventures); and mercury (Hg, *β* = 1 μg mL^−1^; Inorganic Ventures) were used throughout this work. Additionally, ICP multi-element standard solution VI (Certipur, Merck), rare earth element multi-element standard AHF-CAL-7 (Inorganic Ventures), and precious metal multi-element calibration standard #2 (AccuStandard, New Haven, CT, USA) were also used.

Tetramethylammonium hydroxide (TMAH, *w* = 25%; Sigma-Aldrich, Steinheim, Germany) diluted to *w* = 1% in reagent grade I water was used for analysis of the halogens. Sodium chloride (≥99.5%, p.a. grade; Carl Roth GmbH) and potassium bromide (NORMAPUR grade; VWR, Vienna, Austria) salts were used to prepare standards for the analysis of chlorine (Cl) and bromine (Br) respectively.

### Instrumentation

All work was carried out using a NexION 5000 (PerkinElmer, Waltham, MA, USA), an ICP-MS/MS system equipped with a quadrupole-based dynamic reaction cell (DRC). Here, the mass filter (prior to the reaction cell) is termed Q1 and the mass analyser (after the reaction cell) is termed Q3. Instrument parameters for the different measurement modes applied are given in Table 1. Argon (purity 5.0 (≥99.999%); Linde Gas GmbH, Stadl-Paura, Austria) was used as the plasma gas. An in-house tuning solution containing 400 pg g^−1^ Ce was used for tuning the oxide rate to 1.6–1.9%. The rate of Ce^2+^ formation was monitored (Ce^2+^/Ce^+^ of 4.5–5.0%). Nitrous oxide (medicinal grade; Linde Gas GmbH) and oxygen (purity 3.5 (≥99.95%); Linde Gas GmbH) were used as reaction gases.

**Table 1:** Instrument and plasma parameters for ICP-MS/MS measurements using the PerkinElmer NexION 5000

Parameter	Standard mode	O_2_ DRC mode	N_2_O DRC mode
Scan mode	MS/MS	MS/MS	MS/MS
Cell gas	None	O_2_	N_2_O
RPq	0.25	0.45	0.45
Sample introduction	Self-aspiration	Self-aspiration	Self-aspiration
Nebulizer	PFA MicroFlow	PFA MicroFlow	PFA MicroFlow
Spray chamber	Peltier cooled SilQ cyclonic spray chamber	Peltier cooled SilQ cyclonic spray chamber	Peltier cooled SilQ cyclonic spray chamber
Spray chamber temperature	5 °C	5 °C	5 °C
Interface cones	Nickel	Nickel	Nickel
RF power	1600 W	1600 W	1600 W
Ar nebulizer gas flow	0.96–1.01 L min^−1^	0.96–1.01 L min^−1^	0.96–1.01 L min^−1^
Ar auxiliary gas flow	1.2 L min^−1^	1.2 L min^−1^	1.2 L min^−1^
Ar plasma gas flow	16 L min^−1^	16 L min^−1^	16 L min^−1^
QID fixed voltage	−12 V	−12 V	−12 V
Hyperskimmer park voltage	5 V	5 V	5 V
OmniRing park voltage	−185 V	−185 V	−185 V
Inner target lens voltage	2 V	2 V	2 V
Outer target lens voltage	−7 V	−7 V	−7 V
Deflector exit voltage	−8 V	−8 V	−8 V
Differential aperture voltage	−3.5 V	−3.5 V	−3.5 V
Q1 AC rod offset	−6 V	−10 V	−7.5 V
Q1 rod offset	−2 V	−0 V	0 V
Cell rod offset	−33 V	−5 V	−2 V
Axial field voltage	0 V	125 V	250 V
Cell entrance voltage	−5 V	−8.5 V	−7.5 V
Cell exit voltage	−2 V	−5.5 V	−5 V
Q3 AC rod offset	−2.5 V	−7 V	−8 V
Q3 rod offset	−2 V	−10 V	−10 V
Dwell time	50 ms	50 ms	50 ms
Mass range	7–238 amu	7–280 amu	7–285 amu

### Analytical measurement

#### Mass-shift and on-mass determinations

For each element, a calibration was produced using purchased or in-house multi-element standards (ESI A; Table S1a†) and analysed in standard (=no-gas) mode. For DRC measurements, intensities of selected product ions, as well as on-mass intensities, were determined over the range of 0–1 mL min^−1^ gas flows of N_2_O and O_2_. For product ions that had been observed, but had not reached maximum intensity at 1 mL min^−1^, the range was extended to 2 mL min^−1^. Sensitivity was determined based on the slopes of the calibration curve. The formation rate of the product ions from DRC measurements were calculated based on the ratio of the slope of the product ion calibration curve to the slope of the calibration obtained using standard mode (expressed as a percentage).

A qualitative profile of the on-mass interaction of Xe with N_2_O was carried out using the trace levels of Xe found within the Ar gas supply between 0–1 mL min^−1^ of N_2_O.

In order to determine if interfering oxide and doubly-charged ions (which are formed in the plasma) could be removed using on-mass determinations, further measurements were carried out between 0–1 mL min^−1^ using N_2_O only. The instrument was tuned to a higher oxide-rate of 200–300% in standard mode (compared to 1.6–1.9% used previously) by adjusting the *z*-position of the torch 1.5 mm closer to the sampler cone and increasing the nebulizer gas flow to 1.01 mL min^−1^. Formation of Ce^2+^ was monitored (Ce^2+^/Ce^+^ of 40–50%) but not further tuned. In-house multi-element standards were prepared accounting for the interferences on oxides and doubly-charged ions of analytes (ESI A; Table S1b†).

#### Asymmetric charge transfer

Screening of analytes that show asymmetric charge transfer with N_2_O (over the range of 0–1 mL min^−1^) was carried out using multi-element standards (ESI A; Table S1a†) with Q3 set to *m*/*z* of 44. Elements that showed a charge transfer reaction were re-analysed using single element standards with Q3 set to *m*/*z* of 30, 32 and 44 using N_2_O flow rates of 0–2 mL min^−1^. Ar, N, and O were measured with the addition of dilute HNO_3_ blank (*w* = 2%) and with Q1 set to *m*/*z* 38, 15, and 17 respectively.

## Results and discussion

### General observations

#### Mass-shift determinations

**Fig. 1:**
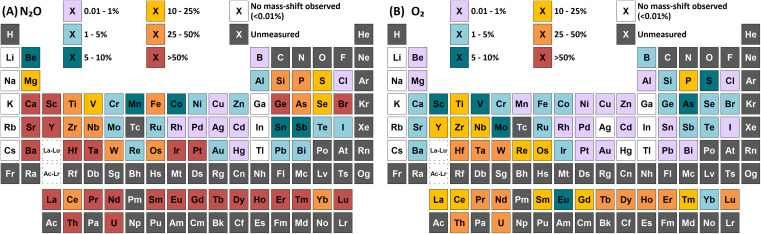
Comparison of maximum achieved sensitivity (relative to standard mode) for mass-shift determinations (by atom transfer) using (A) N_2_O and (B) O_2_ reaction gases.

**Fig. 2:**
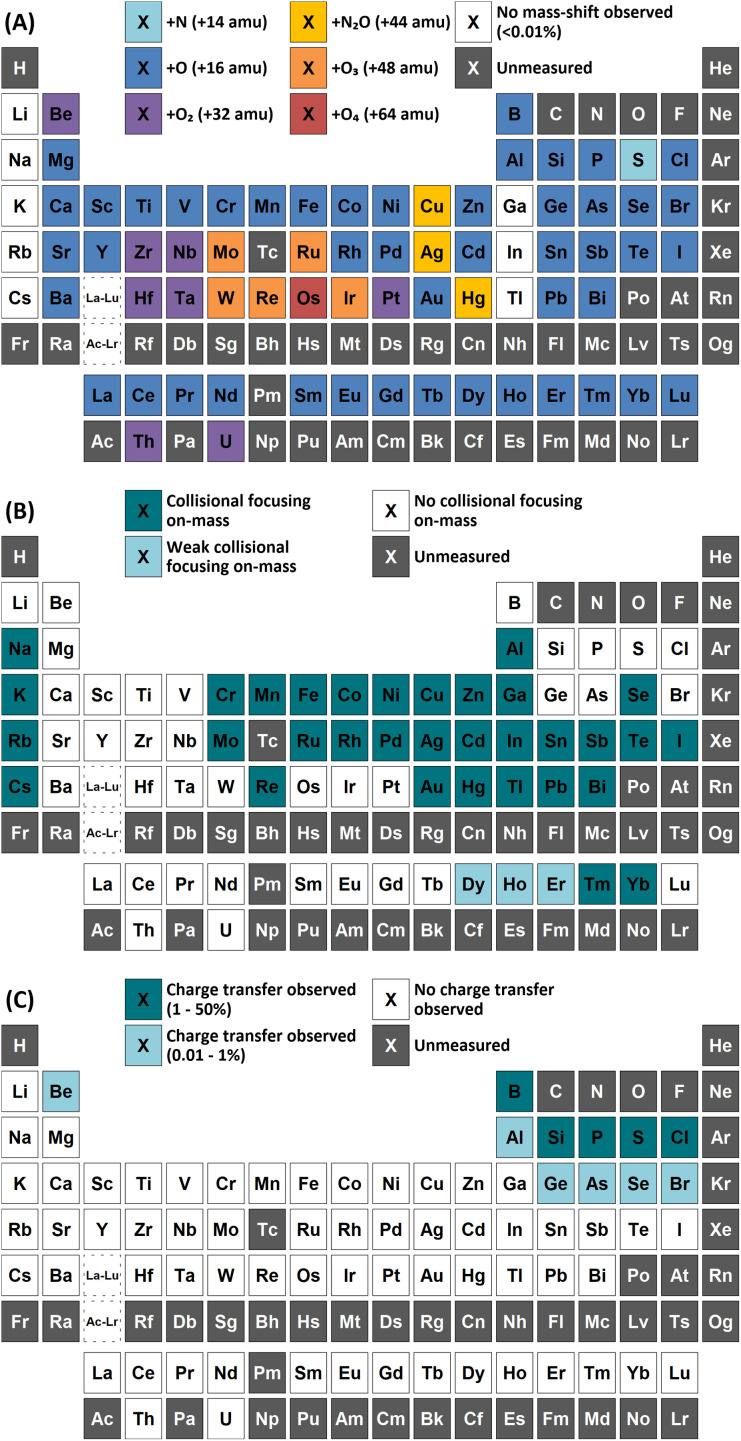
Observations of (A) atom transfer with greatest sensitivity, (B) on-mass collisional focusing, and (C) asymmetric charge transfer reactions using N_2_O reaction gas.

Sensitivities, given by the slope of a calibration curve, were compared between O_2_ and N_2_O reaction gases for mass-shifting of the target analytes (Fig. 1). Of the 73 elements measured, 59 showed greater sensitivities when using N_2_O compared to O_2_. Li, Na, K, Rb, Cs, Ga, In, and Tl did not react with either gas. The use of O_2_ provided higher sensitivities for the analysis of B, Mo, Ru, and Re. Finally, W and Cl showed minimal difference in sensitivity (<10%) using either gas. While the formation of dioxide (MO_2_^+^, +32 amu) and, for very few elements, minimal formation of the trioxide (MO_3_^+^, +48 amu) product ions were observed using O_2_, the monoxide (MO^+^, +16 amu) mass-shift produced the greatest sensitivity for all elements in each case. This was in contrast to previous studies using different ICP-MS/MS instrumentation where certain elements showed greater formation of the dioxide product ions.^6^ Using N_2_O, certain elements showed higher sensitivities for the formation of the MO_2_^+^, MO_3_^+^, or tetroxide (MO_4_^+^, +64 amu) product ions, as well as numerous different N-containing clusters. The product ions that gave the highest sensitivity for each element are highlighted in Fig. 2A. Additionally, it was observed that the variation of sensitivity with reaction gas flow rate generally provided much sharper optimums using N_2_O compared to the broader optimums using O_2_. This suggests that, while N_2_O does generally provide higher sensitivities of formed product ions compared to O_2_, the optimum gas flow range to capitalize on this sensitivity gain is rather narrow, which is a potential limitation since gas flows have to be adapted for each single analyte individually. Plots of variation of selected product ion formations with N_2_O and O_2_ flow rates for each element are included in ESI B.† Tables of maximum sensitivity of selected product ions and their optimum flow rate are provided in ESI A (Table S2 and S3†). Additionally, a comparison of observed limits of detection (LODs) and background equivalence concentrations (BECs) between standard mode and mass shift determinations with N_2_O and O_2_ is also provided (Table S4 and S5†).

#### On-mass determinations

The effect of N_2_O and O_2_ when used for on-mass determinations was also monitored. In total, 36 of the 73 measured elements showed a collisional focusing effect for on-mass determinations using N_2_O (Fig. 2B). Generally, the effect was observed for elements that displayed lower atom transfer reactivity with N_2_O, however Fe, Tm, and Yb (which react well with N_2_O, but not completely) also showed a collisional focusing effect. While the collisional focusing effect is considered to give an apparent increase in sensitivity, the use of N_2_O in this study only provided a moderate increase in sensitivity (compared to standard mode) for Au, Hg, and Tl, and an overall reduction in sensitivity for all other elements. This is likely due to a combination of the high collisional cross section of N_2_O, the high reactivity (which removes some of the analyte by atom transfer), and that the gas cell voltages were optimised specifically for mass-shift. However, the presence of the collisional focusing effect for certain elements meant that suppression of interferences was possible where interferences did not display this effect. No such collisional focusing effect was observed when using O_2_ as the cell gas. Therefore, the use of N_2_O can be a distinct advantage over O_2_ as it can provide greater sensitivities for on-mass determinations for less reactive elements, as well as increased sensitivity for mass-shift determinations of more reactive elements. Variation of analyte sensitivity on-mass with N_2_O flow rate is provided in ESI B.† Equally, variation of doubly-charged (M^2+^) and MO^+^ interferences of selected elements have also been included. Comparison of observed LODs and BECs between standard mode and on-mass determinations with N_2_O is provided in ESI A (Table S6†).

#### Asymmetric charge transfer

The use of N_2_O produced asymmetric charge transfer reactions with N^+^ (as well as N_2_^+^), O^+^, Cl^+^, and Ar^+^, forming ^14^N_2_^16^O^+^ on *m*/*z* 44 as well as ^14^N^16^O^+^ on *m*/*z* 30 and ^16^O_2_^+^ on *m*/*z* 32. However, it was additionally observed that 10 analytes with ionisation energies lower than that of N_2_O also underwent a charge transfer reaction – primarily the lighter p-block elements (Fig. 2C, Table S7†). For such reactions to be thermodynamically favourable, the analyte ions in the gas cell must be in an excited, metastable (M_(m)_^+^) state (formed in the plasma) that have energies greater than the ionisation energy of N_2_O. Such asymmetric charge transfer reactions have previously been reported for selected elements using O_2_ gas^24^ and occurs for ions that exhibit low-lying metastable ionic states. Observations of such charge transfer reactions using N_2_O are discussed further here for individual elements.

### Reaction with argon

Both N_2_O and O_2_ primarily react with Ar by the process of an asymmetric charge transfer reaction. For O_2_, the ^16^O_2_^+^ product was primarily observed, with an additional small formation of ^38^Ar^16^O^+^ (0.008%) on *m*/*z* = 54 at 0.1 mL min^−1^ that fell to baseline levels by 0.5 mL min^−1^. In the case of N_2_O, the primary product formed was the ^14^N_2_^16^O^+^, with ^14^N^16^O^+^ and ^16^O_2_^+^ also present at lower intensities. No formation of ^38^Ar^16^O^+^ was observed on *m*/*z* = 54 and, as reported previously, no significant oxide formation was noted for ^40^Ar above 0.4 mL min^−1^ when considering the oxide formation from blank ^40^Ca.^25^

### s-Block elements

Group 1 metals do not react with either N_2_O or O_2_. However, a collisional focusing effect was observed for on-mass determinations of these elements using N_2_O, with the exception of Li. The magnitude of this effect was observed to increase down the group. On the other hand, group 2 metals react greatly with N_2_O to form the monoxide product ion, with negligible formation of the dioxide product ion (<0.1%) observed (except Be). As such, the use of N_2_O is a highly effective option of avoiding the isobaric interferences of ^40^Ar^+^ and ^40^K^+^ on ^40^Ca^+^ (as well as ^24^Mg^16^O^+^)^25,26^ and ^87^Rb^+^ on ^87^Sr^+^,^26,27^ as well as for the removal of isobaric Ba^+^ interferences for measurements of Cs radioisotopes.^28–30^ Relative sensitivities of the oxide product ion (compared to standard mode) increased down the group, from Mg (13%) to Ba (70%). In the case of Be, both the monoxide and dioxide product ions formed, with relative sensitivities of 5.5% and 7.8% compared to that of standard mode respectively. The group 2 metals did not react well with O_2_ (<2% formation) as it is an endothermic reaction. Be also showed evidence of charge transfer with N_2_O, with 0.28% formation of ^14^N^16^O^+^ and 0.12% formation of ^14^N_2_^16^O^+^.

### p*-*Block elements

#### Group 13 elements

Mass shift of B^+^ to BO^+^ was found to give a greater sensitivity using O_2_ compared to N_2_O, albeit still with a low rate of formation (2.1%). Instead, the asymmetric charge transfer reaction of B_(m)_^+^ with N_2_O gave the greatest sensitivity (19% formation of ^14^N_2_^16^O^+^). Conversely, mass shift of Al was found to give increased sensitivity using N_2_O compared to O_2_, though with similarly low rate of AlO^+^ formation (1.5%). In addition, collisional focusing was observed for on-mass determination of Al using N_2_O. While an asymmetric charge transfer reaction of Al_(m)_^+^ with N_2_O was observed, the rate was much lower than that observed for B (^14^N^16^O^+^: 0.01%, ^14^N_2_^16^O^+^: 0.01%). Ga, In, and Tl did not react with either N_2_O or O_2_, although collisional focusing was observed when measuring on-mass with N_2_O. For Tl, oxide interferences of d-block elements can be effectively reduced using this approach, whereas Al and Ga still suffer from certain oxide-based interferences (Al^+^ from BO^+^ and Be^+^; Ga^+^ from MnO^+^). For In, on-mass determination with N_2_O did not resolve the isobaric interferences of ^113^Cd^+^ and ^115^Sn^+^ as both of these elements were also found to exhibit collisional focusing on-mass.

#### Silicon

The use of N_2_O formed new interferences at lower shifted masses (*e.g.* +14 and +16 amu). The most abundant isotope, ^28^Si (92.23%), was unable to be used for mass-shift to ^28^Si^16^O^+^ (*m*/*z* = 44) with N_2_O as this product ion was masked by a large formation of the isobaric interference ^14^N_2_^16^O^+^, formed by the process of a charge transfer reaction with ^14^N_2_^+^, as the lower ionisation potential of N_2_O (12.886 eV) compared to that of N_2_ (15.5808 eV) renders such a reaction favourable.^14^ Mass-shift of ^30^Si^+^ to ^30^Si^14^N^+^ and ^30^Si^16^O^+^ was found to suffer from interference due to the reaction of ^14^N^16^O^+^ with N_2_O to form ^14^N_2_^16^O^+^ and ^14^N^16^O_2_^+^ respectively and varied with the matrix HNO_3_ concentration. Therefore, ^29^Si was used to assess the product ions formed.

A wide variety of product ions were observed to form when using N_2_O. High formation of the SiO^+^ product ion (29%) was obtained – an improvement of 9 times greater than using O_2_ reaction gas. Additionally, the charge transfer reaction between ^29^Si_(m)_^+^ and N_2_O was found to give higher formations of ^16^O_2_^+^ (28%) compared to the formation of ^14^N_2_^16^O^+^ (23%) and ^14^N_2_^16^O^+^ (7.3%). While mass-shift of ^28^Si with O_2_ to ^28^Si^16^O^+^ has previously been demonstrated to successfully remove interferences and provide low-level Si determinations,^31^ the high background due to charge transfer with ^14^N_2_^+^ that cannot be resolved makes the same approach using N_2_O unfavourable. A good alternative is to mass-shift using the ^28^Si^16^O_2_^+^ product ion, which gave 11% formation. This approach allows for an increase of 3.4 times sensitivity and similar LODs and BECs compared to O_2_.

#### Phosphorus and sulphur

Both P and S showed high formation of the PO^+^ and SO^+^ product ions (28% and 11% respectively) using N_2_O, giving 2.8 times and 1.5 times better sensitivity than for mass-shifting using O_2_. Additionally, both elements formed the MN^+^ product ion at a significant rate. Both gases also displayed similar effective reduction in the LODs and BECs of 1–2 orders of magnitude (Table S4 and S5†). However, as with Si, the interference of NO^+^ from nitric acid causes a significant problem due to formations of N_2_O^+^ and NO_2_^+^. S showed a 5.9% formation of the SO_2_^+^ product ion using N_2_O, whereas minimal formation of SO_2_^+^ was observed using O_2_. Such formation of SO_2_^+^ allows for reduction of interference of NO^+^, which is not possible with the SN^+^ or SO^+^ product ions. Conversely, P did not show similarly high rates of PO_2_^+^ formation and therefore still suffers from NO^+^ interference.

In addition, both P and S showed a high rate of charge transfer with N_2_O due to the formation of metastable ions. In both cases, formation of the ^14^N^16^O^+^ product ion was the greatest, at 16% and 12% for ^31^P_(m)_^+^ and ^34^S_(m)_^+^ respectively. Charge transfer of ^31^P_(m)_^+^ additionally formed ^14^N_2_^16^O^+^ at 12% and ^16^O_2_^+^ at 1.9% (although this may also include partial formation of ^31^P^1^H^+^ from trace impurities in the N_2_O, such as water vapour), while charge transfer of ^34^S_(m)_^+^ additionally formed ^14^N_2_^16^O^+^ at 5.5% and ^16^O_2_^+^ at 6.2%. Such a process may also be an option for removing interferences for both elements.

#### Germanium, arsenic, and selenium

Ge, As, and Se all showed greater sensitivity using N_2_O to mass-shift from M^+^ to MO^+^ compared to O_2_, with the formation rates of these elements using N_2_O decreasing across the period. Ge showed the greatest improvement, with 29 times greater sensitivity using N_2_O (68% formation), allowing for more effective reduction of interferences from the d-block metal oxide interferences. However, in extremely high Fe-containing matrices, interference of FeO^+^ on *m*/*z* 70, 72, 73, and 74 is likely not completely removed, as a small degree FeO_2_^+^ was observed after mass-shift of Fe^+^ (0.4%).

As and Se showed improvements in sensitivity of 4.4 times and 2.9 times respectively using N_2_O, while the formation rate achieved was 37% and 11% respectively. The use of N_2_O still allows for the effective removal of interferences of d-block element oxides and doubly-charged f-block elements, as well as interferences of ArCl^+^ (by charge transfer),^32^ while boosting sensitivity and detection limits compared to O_2_. ^74^Se^+^ also shares an isobaric interference with ^74^Ge^+^. The mass-shift approach only allowed for the measurement of ^74^Ge^+^ with reduced interference from ^74^Se^+^, as optimum sensitivity for ^74^Ge^+^ was obtained with a lower flow rate of N_2_O (0.4 mL min^−1^) compared to that of ^74^Se^+^ (1.3 mL min^−1^) but the sensitivity of ^74^Ge^+^ still remained relatively high at higher flow rates. Alternatively, Se showed collisional focusing on-mass using N_2_O, whereas Ge did not. Therefore, on-mass determinations of ^74^Se^+^ may be more suited to reducing the isobaric interference from ^74^Ge^+^ and ^76^Ge^+^ in addition to the aforementioned interferences, with the exception of Sm^2+^ and Eu^2+^, which also showed collisional focusing (see f-Block section).

A charge transfer reaction was also observed between Ge_(m)_^+^ and N_2_O, forming ^14^N_2_^16^O^+^ at 0.19%. Interestingly, As and Se also showed evidence of charge transfer with N_2_O despite the ionisation potential of the low-lying energy levels of metastable As_(m)_^+^ (9.920–12.590 eV) and Se_(m)_^+^ (11.385–12.715 eV)^24^ being slightly lower than that of N_2_O (12.886 eV), thus leading to an endothermic reaction that should be unfavourable. ^14^N_2_^16^O^+^ formations of 0.57% and 0.12% were observed for As and Se. It could be that the charge transfer reaction proceeds due to the addition of the kinetic energy component of the ions passing through the cell, or perhaps that the ions exist in an even higher energy state (induced by charge transfer reactions with metastable Ar_(m)_^+^). Further investigation is required to evaluate the reason for this anomaly.

#### Tin, lead, antimony, bismuth, and tellurium

Similar to Ge, As, and Se, the heavier p-block elements tended to only form MO^+^ product ions with N_2_O, with formations decreasing along both the periods and the groups. Additionally, collisional focusing was observed when using N_2_O. This was contrary to the observed mass-shifts with O_2_, where only Sb and Te showed significant MO^+^ formation (4.8% and 1.9% respectively), with Sn, Pb, and Bi showing minimal reaction (<1%).

Of these elements, Sn showed the greatest benefit from mass-shift using N_2_O, with 9.8% formation. This allows for the removal of the isobaric interference of ^115^In^+^ on ^115^Sn^+^, as well as the removal of interferences from oxides of d-block elements. However, ^238^U^2+^ may be an issue due to observed high UO_2_^+^ formation with N_2_O, which may also indicate high formation ^238^U^16^O_2_^2+^ that would interfere on ^119^Sn^16^O^+^. Here, the collisional focusing effect is advantageous, as this offers reduction of ^238^U^2+^ as well as the aforementioned interferences. For Sb, Te, Pb, and Bi, on-mass determinations may be preferable for maintaining higher sensitivity and reducing oxide-based interferences, however the isobaric interferences between Sn, Sb, and Te cannot be resolved using N_2_O due to similarities in reactivity and collisional focusing effects.

#### Halogens

Reaction of Br and I with N_2_O gave much higher sensitivities for the formation of the MO^+^ product ion compared to that of O_2_ reaction gas (48× and 3.6× greater sensitivity respectively). However, only Br showed a high rate of oxide formation (57%), allowing for reduced interference from CuO^+^, ZnO^+^, and doubly-charged f-block elements. Conversely, formation of IO^+^ was much lower at 1.8%. Instead, the observed collisional focusing on-mass using N_2_O allows for a greater sensitivity. The radionuclide ^129^I (used for isotope dilution) suffers interference from ^129^Xe, which can be present in trace quantities in the Ar gas supply. O_2_ has been used previously to successfully resolve this interference on-mass.^33^ Here, on-mass measurements of trace ^129^Xe and ^132^Xe with N_2_O showed a collisional focusing effect, which indicates that the isobaric interference of ^129^Xe on ^129^In cannot be resolved in this way. Therefore, O_2_ is likely more preferable in this case.

Cl did not show a significant improvement in sensitivity of oxide formation, with a ClO^+^ formation rate of approximately 0.7% for both reaction gases. Instead, Cl primarily underwent a charge transfer reaction process, as the ionisation potential of Cl^+^ (12.968 eV) is greater than that of both N_2_O and O_2_. Here, the formation of ^14^N_2_^16^O^+^ was 50%, with additional formations of ^16^O_2_^+^ and ^14^N^16^O^+^ at 0.76% and 0.88% respectively. Asymmetric charge transfer was also observed for Br (due to Br_(m)_^+^) but to a much lower degree, with ^14^N_2_^16^O^+^ formation of 0.60% and ^14^N^16^O^+^ formation of 0.07% observed.

### d-Block elements

**Fig. 3:**
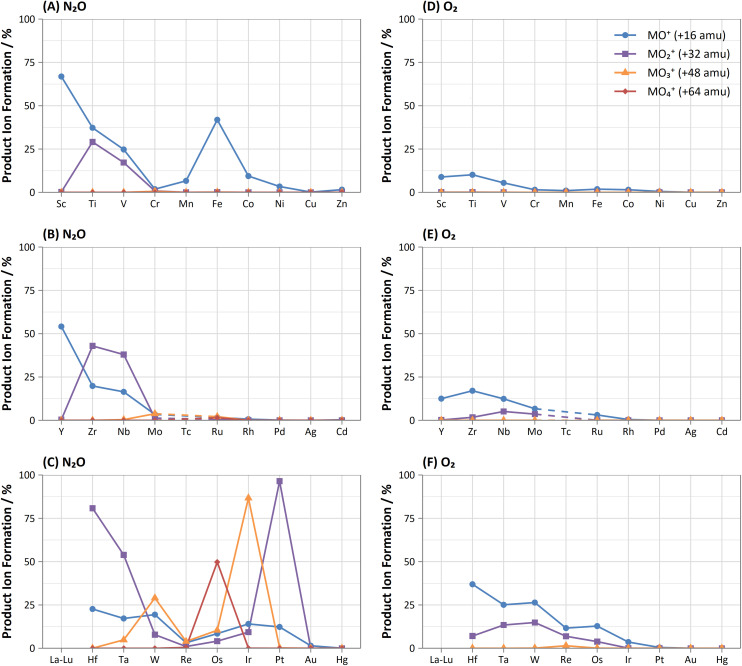
Comparison of obtained maximum formations of metal-oxide (blue, circle), -dioxide (purple, square), -trioxide (orange, triangle), and -tetroxide (red, diamond) product ions for d-block elements using (A–C) N_2_O and (D–F) O_2_ reaction gases between 0–1 mL min^−1^.

Comparison of the formations of selected product ions for the d-block elements using N_2_O and O_2_ reaction gases is provided in Fig. 3.

#### Scandium and yttrium

Using N_2_O, Sc and Y both showed high oxide formation (67% and 54% formation respectively) with minimal dioxide formation (<0.4%). Compared to the use of O_2_, N_2_O provides a sensitivity enhancement of 7.5 times for Sc and 4.1 times for Y. Given both elements are monoisotopic, it is vital to resolve the spectral interferences. In the case of ^89^Y, mass shift to ^89^Y^16^O^+^ can resolve interferences of ^73^Ge^16^O^+^ and ^71^Ga^18^O^+^, as well as interferences from transition metals that form clusters with Ar, as they do not readily react with N_2_O. However, the observed high formations of HfO_2_^+^ in the reaction cell may indicate that the isobaric interference of ^178^Hf^2+^ may not be removed using either reaction gas. In the case of Sc, interference of ^89^Y^2+^ (on *m*/*z* = 44.5) may be removed (due to observed low formations of the dioxide product ion), however the major interference of SiO^+^ (primarily ^28^Si^17^O^+^ and ^29^Si^16^O^+^) is not removed using N_2_O due to the observed high formation of the SiO_2_^+^ product ion. Therefore, in high Si-containing matrices, O_2_ may perform better as a reaction gas due to the much lower observed formation of SiO_2_^+^ in the reaction cell. The discussion for La and Lu is contained within the lanthanides section (f-Block elements).

#### Group 4 and 5 elements

Both the Ti- and V-group elements also react very well with O_2_ and N_2_O, with relative sensitivities of the product ions generally increasing down the groups. For example, oxide formations using O_2_ gave relative sensitivities varying from 10% (for Ti) to 33% (for Hf), and from 5.5% (for V) to 37% (for Ta). When using N_2_O as a reaction gas, the dioxide product ion gave the best sensitivities for Zr, Nb, Hf, and Ta, with the relative sensitivities of Hf and Ta notably reaching 81% and 54% respectively. This allows for effective removal of interferences from p-block and f*-*block element oxides, as well as the removal of Hf^2+^, Ta^2+^ and W^2+^ interferences on Zr^+^ and Nb^+^ (however Os was found to form OsO_4_^+^ with a high rate, so Os^2+^ interferences may not be removed). Ti and V gave the greatest relative sensitivities when monitoring the MO^+^ product ion (37% and 25% respectively). Although, it should be noted that the MO_2_^+^ product ion also formed at a high rate (29% and 17% respectively) and could also be utilized – for example, to remove Zr^2+^, Nb^2+^, Mo^2+^, and Ru^2+^ interferences, and to remove SO^+^ interferences in high S-containing matrixes. Fu *et al.* proposed an alternative approach to remove SO^+^ interferences on V^+^ using the VN^+^ product ion.^34^ However this approach was found not to be viable in this study, as the VN^+^ product ion formed at only 0.5%, which could be due to the difference in the type of reaction cell design used in the NexION 5000. Ti also showed only a small formation of TiN^+^ at 2.6% however, formation of the MN^+^ product ion was observed to be much greater for Zr (16%), Nb (8.1%), Hf (10%) and Ta (7.7%).

#### Chromium, manganese, iron, and cobalt

Fe showed high reactivity with N_2_O, forming the FeO^+^ product ion at a rate of 42%. Mn and Co reacted less with N_2_O, with 6.6% and 9.4% formation of the MO^+^ product ion respectively, while Cr only showed 1.9% formation. Mass-shift of these elements allowed for effective reduction of Ar-based interferences (such as ArC^+^ and ArO^+^),^34^ M^2+^ interferences (from p-block elements), and MO^+^ interferences of ClO^+^, KO^+^ and CaO^+^. Of these analytes, Fe showed the greatest benefit from mass-shifting, as the LOD and BEC were lowered by 340 times and 8300 times respectively (Table S4†). Cr, Fe, Mn, and Co additionally showed collisional focusing on-mass with N_2_O, which provides an alternative approach to reducing interferences and maintaining high sensitivity, particularly in the case of Cr as the LOD and BEC were reduced by 180 times and 5600 times respectively (Table S6†).

#### Molybdenum, ruthenium, and rhodium

Mo, Ru, and Rh showed generally low atom transfer reactivity with N_2_O. The highest sensitivity for Mo and Ru was obtained for the MO_3_^+^ product ion (3.8% and 2.1% respectively). Rh showed the highest formation for the RhO^+^ product ion, although the formation was <1% for both gases. In comparison, O_2_ performed 1.8 and 1.5 times better for Mo and Ru respectively. While the use of N_2_O for mass-shift of these elements can reduce interferences, the low sensitivity is a limiting factor. On-mass determinations with N_2_O showed collisional focusing for all three elements. This allows for the reduction of doubly-charged interferences from W^2+^, Os^2+^, and Ir^2+^, as well as isobaric interferences from Zr^+^ on Mo^+^, which are removed by the process of atom transfer. However, interferences from high levels of BrO^+^, SeO^+^, and SrO^+^ cannot be resolved on-mass.

#### Tungsten, rhenium, osmium, and iridium

Numerous product ions were observed for W, Os, and Ir using N_2_O. In particular, high formations of MN^+^, MO^+^, MO_2_^+^, and MO_3_^+^ were observed. Both W and Ir showed the highest sensitivity for the MO_3_^+^ product ion, at 29% and 87% formation respectively. Compared to the use of O_2_, N_2_O provided little benefit in terms of sensitivity for W. However, for Ir, a marked improvement in sensitivity of 24 times was observed. Both gases can be used to effectively remove the lanthanide oxide interference. However, neither gas can eliminate the isobaric interferences of ^180^Ta^+^ and ^180^Hf^+^ on ^180^W^+^, or ^186^Os^+^ on ^186^W^+^ due to the similar formation of product ions. Os additionally formed the OsO_4_^+^ product ion at a high rate (50% formation) with N_2_O, which provided a 3.8 times greater sensitivity than for O_2_. Additionally, N_2_O allows for the resolution of isobaric interferences from ^184^W^+^, ^186^W^+^, ^190^Pt^+^ and ^192^Pt^+^, which did not form significant MO_4_^+^ product ions, as well as the reduction of isobaric interference from ^187^Re^+^, since Re was observed to also form ReO_4_^+^ but at a much lower rate (0.48%). In comparison, Re did not show high sensitivity using mass-shift with N_2_O (maximum of 3.8% formation for ReO_3_^+^), with O_2_ providing 3.2 times greater sensitivities. Collisional focusing was observed on-mass using N_2_O, which also allows for the reduction of isobaric interference from ^187^Os^+^ and higher sensitivity, however interferences from lanthanide oxides persist.

#### Group 10, 11 and 12 elements

With the exception of Pt, the Ni-, Cu-, and Zn-group elements generally showed minimal reaction with O_2_ (≤1% formation) or N_2_O (≤3.4% formation). Ag showed no oxide formation using either gas and Hg showed no oxide formation using O_2_. These elements also formed the MN_2_O^+^ product ion using N_2_O reaction gas, albeit also with very low formation rates (≤1% formation). However, observed formations of the MO_2_^+^ and MN_2_O·O^+^ product ions from interfering Ti- and V-group transition metals may mean that resolution of interfering transition metal oxides is not likely to be possible by mass-shifting.

Collisional focusing of Au and Hg, as well as removal of TaO^+^, HfO^+^, WO^+^, ReO^+^ and OsO^+^ with N_2_O, allows for on-mass determinations. Equally, the same approach could resolve the interferences of ZrO^+^, NbO^+^, and MoO^+^ on Ag^+^ and Cd^+^. However, Cd also suffers from isobaric interferences of Pd^+^, In^+^, and Sn^+^, which could not be resolved in this way due to similar collisional focusing effects of these elements. Therefore, for routine measurements, on-mass determinations of Cd with N_2_O on *m*/*z* = 111 would be advisable. Oxide interferences of Be- and Sc-group elements, as well as VO^+^, CrO^+^, and MnO^+^ demonstrated collisional focussing on-mass. Therefore, these interferences cannot be resolved for Co, Ni, and Cu, as well as Pd using N_2_O.

Contrary to the other elements in these groups, Pt shows markedly higher reactivity with N_2_O, while still producing minimal oxide formation using O_2_. In this case, the PtO_2_^+^ product ion gave the highest sensitivity, with 96% formation. Therefore, mass-shift of Pt to PtO_2_^+^ could be used to resolve interferences from HfO^+^, TaO^+^, and even the isobaric interferences of ^196^Hg^+^ and ^198^Hg^+^, however the observed high formation of WO_3_^+^ may indicate that the WO^+^ interference (on *m*/*z* = 196 and 198) may not be resolved. Additionally, isobaric interferences from ^190^Os^+^ and ^192^Os^+^ cannot be resolved due to high formation of OsO_2_^+^.

### f-Block elements

#### Lanthanides

**Fig. 4:**
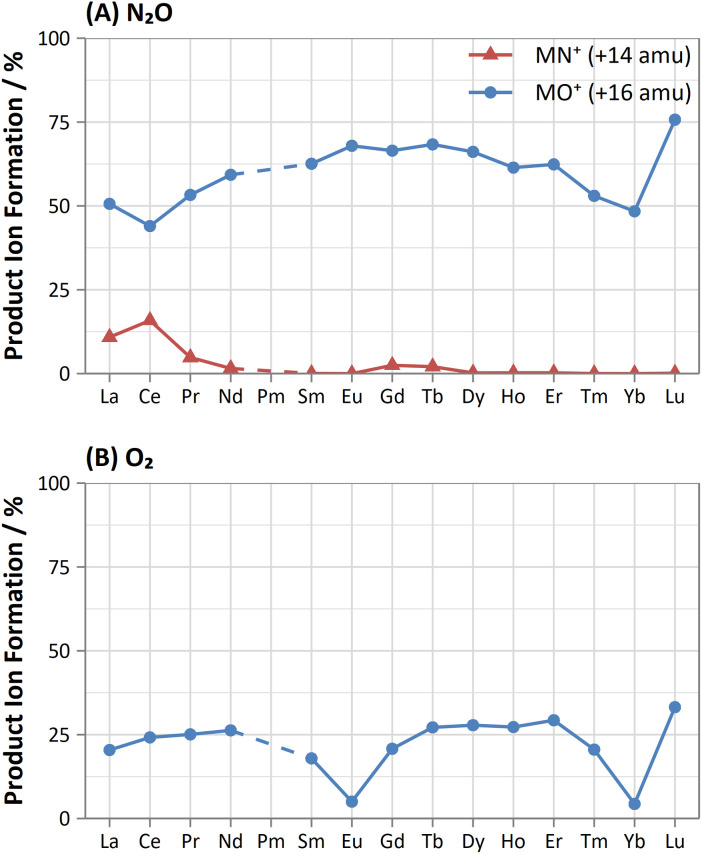
Comparison of obtained product ion formations for oxide (blue, circle) and nitride (red, triangle) product ion formation using (A) N_2_O and (B) O_2_ reaction gases.

A comparison of the obtained relative sensitivities of lanthanide oxides for N_2_O and O_2_ is given in Fig. 4. Similar to the Sc-group elements (see d-block elements), the lanthanides show high oxide formation (44–76%) and low dioxide formation (<0.6%, with the exception of Lu, which gave 2.8%) using N_2_O as a reaction gas. Additionally, N_2_O provided greater sensitivity compared to O_2_ for all lanthanides, especially for Eu and Yb (13.6 and 11.2 times greater sensitivity respectively), which showed much lower sensitivities with O_2_ (due to the lower bond dissociation energies of EuO^+^ and YbO^+^ that result from the half- and fully-filled f-orbital respectively)^35,36^ whereas similarly low sensitivities were not observed when using N_2_O (also observed in literature).^6,23^ However, other examples of a similar periodicity were observed using N_2_O. High formation of the MN^+^ product ion was obtained for La and Ce (11% and 16% respectively), with further elements showing decreasing formation towards Eu. Gd and Tb also showed elevated MN^+^ formation (2.5% and 2.1% respectively), with subsequent elements, again, showing lower formations.

The flow rate of N_2_O that gave the greatest MO^+^ sensitivity increased across the period, from 0.5 mL min^−1^ for La to 0.8 mL min^−1^ and 0.7 mL min^−1^ for Yb and Lu respectively, whereas for O_2_ the flow rate was in most cases 0.3 mL min^−1^ (but slightly higher for Eu and Yb). Both N_2_O and O_2_ are capable of resolving oxide-based interferences of other lanthanides, as well as SnO^+^, SbO^+^, TeO^+^, CsO^+^, and BaO^+^, however the increased sensitivity offered by N_2_O makes it more preferable.

**Fig. 5:**
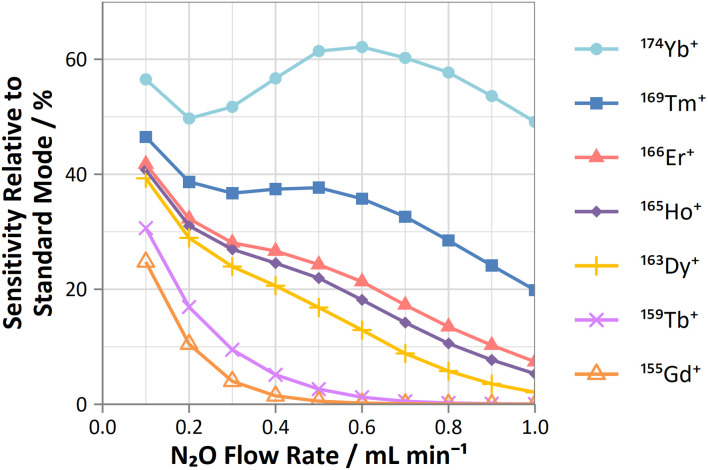
Profiles of sensitivities for Gd, Tb, Dy, Ho, Er, Tm, and Yb (relative to standard mode) determined on-mass using N_2_O reaction gas.

On-mass determinations with N_2_O showed evidence of collisional focusing from Dy to Yb, which became more prevalent across the period (Fig. 5). The observed strong on-mass collisional focusing effect for Yb allows for the reduction of isobaric interferences from ^174^Hf^+^, ^176^Hf^+^ and ^176^Lu^+^, as no collisional focusing effect was observed for these interfering elements. However, the reduction of isobaric interferences from ^168^Er^+^ and ^170^Er^+^ are less effective as the interference also exhibits some collisional focusing effect. Equally so, any interferences of lanthanide oxides formed in the plasma also could not be resolved on-mass due to the collisional focusing effect. Therefore, this observation may be limited to a few specific applications.

Furthermore, on-mass collisional focusing of doubly-charged lanthanides with N_2_O was observed primarily for Sm^2+^, Eu^2+^, Tm^2+^ and Yb^2+^ (ESI B†), whereas other doubly-charged lanthanides were generally removed with the addition of N_2_O. This indicates that on-mass determinations of other analytes (such as As^+^ and Se^+^) will continue to suffer interferences from these four lanthanides. Product ion scans of the doubly-charged lanthanides revealed minimal product ion formation where a strong collisional focusing effect was observed, which was similarly observed for other singly-charged elements with low reactivity (Fig. 2A and B). Doubly-charged lanthanides that did not show collisional focusing were removed by mass-shifting with N_2_O to a different *m*/*z*. For these lanthanides, MO^+^ was the major product ion observed, with MO^2+^ and N_2_O^+^ product ions additionally present. The M^+^ product ion was not observed in any case. The observed product ions likely indicate an initial atom transfer reaction to form MO^2+^, followed by a charge transfer reaction with N_2_O to form MO^+^ and N_2_O^+^. This is supported in part by the thermodynamically unfavourable (endothermic) charge transfer reaction between N_2_O and doubly-charged lanthanides (second ionisation potentials between 10.55–12.18 eV for La–Yb)^14^ combined with the absence of M^+^ product ions. However, this cannot yet be stated with certainty as literature values for the second ionisation potential of gas phase lanthanide oxides is, to the authors' knowledge, not presently available.

#### Actinides

Contrary to the reactions observed for the lanthanides, the two actinides measured in this study (Th and U) showed substantially higher formation of the MO_2_^+^ product ion (63% and 51% respectively), with a reduced formation of the MO^+^ product ion (23% and 27% respectively), when using N_2_O as a reaction gas. Additionally, higher formation of the MNO^+^ product ion, as well as the MN^+^ product ion, were observed. Reaction with O_2_ also showed the formation of both the MO^+^ and MO_2_^+^ product ions, however the MO^+^ product ion showed greater relative sensitivities.

## Conclusions

This work highlights the versatility and applicability of N_2_O as a reaction cell gas for determinations of 73 elements. While it may not be suitable for every individual application, its high effectiveness of oxide formations compared to oxygen, as well as its ability to induce a collisional focusing effect on-mass, makes it a strong choice for routine analysis using ICP-MS/MS.

While the rate of product ion formation by atom transfer may traditionally be the primary determining factor for performing mass-shift or on-mass determinations, the data provided here demonstrates considerable overlap of effectiveness of both approaches. Given this context, different interference removal approaches using N_2_O may be preferable depending on the level of interfering matrix elements and the concentration of the target analyte in solution.

## Author contributions

Shaun T. Lancaster: conceptualization, investigation, data curation, writing–original draft preparation; Thomas Prohaska; conceptualization, supervision, writing–review and editing; Johanna Irrgeher: conceptualization, funding acquisition, supervision, writing–review and editing.

## Conflicts of interest

There are no conflicts to declare.

## Supplementary Material

JA-038-D3JA00025G-s001

JA-038-D3JA00025G-s002
