# Supplementary material for: Characterisation of gas cell reactions for 70+ elements using N2O for ICP tandem mass spectrometry measurements
Source: J Anal At Spectrom. 2023 Apr 28;38(5):1135–45. doi: 10.1039/d3ja00025g (PMC10171245; doi:10.1039/d3ja00025g)
Supplement: JA-038-D3JA00025G-s001 [file JA-038-D3JA00025G-s001.pdf]

Electronic Supplementary Material (ESI) for JAAS.
This journal is © The Royal Society of Chemistry 2023

Supplementary information to

Characterisation of gas cell reactions for 70+ elements using N_2O for ICP tandem mass spectrometry measurements

Shaun T. Lancaster[a*], Thomas Prohaska[a,b] and Johanna Irrgeher[a,b]

[a]Department of General, Analytical and Physical Chemistry, Chair of General and Analytical Chemistry, Montanuniversität Leoben, Leoben, Austria.

[b]Department of Physics and Astronomy, University of Calgary, Calgary, Canada.

Table S1: Composition of multi-element standards used for evaluation of on-mass and mass-shift determinations. Measured analytes in each standard are highlighted in bold. Elements measured as single element standard solutions are given in italics.

Standard	Matrix	Analytes
ICP multi-element standard solution VI	*w* = 2% HNO_3	Li, **Be**, **B**, Na, Mg, Al, K, Ca, V, **Cr**, Mn, Fe, Co, Ni, Cu, Zn, Ga, **As**, Se, Rb, Sr, Mo, Ag, Cd, Ba, **Pb**, **Bi**, U.
AHF-CAL-7	*w* = 2% HNO_3	**Y**, **La**, **Ce**, **Pr**, **Nd**, **Sm**, **Eu**, Gd, Tb, Dy, Ho, Er, Tm, Yb, Lu, **Th**, **U**.
Precious Metals	*w* = 2% HNO_3	**Ru**, **Rh**, **Pd**, **Ir**, **Pt**, **Au**.
In-house A	*w* = 2% HNO_3	**Sc**, **Ti**, **Ge**, **Re**, **W**.
In-house B	*w* = 2% HNO_3	**Nb**, **Sn**, **Sb**, **Ta**.
In-house C	*w* = 2% HNO_3	**Al**, **Ca**, **V**, **Ni**, **Cu**, **Se**, **Cd**, **Ba**.
In-house D	*w* = 2% HNO_3	**Mg**, **Mn**, **Fe**, **Zn**, **Sr**, **Mo**, **Te**.
In-house E	*w* = 2% HNO_3	**Gd**, **Tb**, **Dy**, **Ho**, **Er**, **Tm**.
In-house F	*w* = 2% HNO_3	**Yb**, **Lu**.
In-house G	*w* = 2% HNO_3	**Li**, **K**, **Ga**, **Ag**, **Cs**, **Tl**.
In-house H	*w* = 2% HNO_3	**Na**, **Rb**.
In-house I	*w* = 2% HNO_3	**S**, **P**, **Zr**.
In-house J	*w* = 2% HCl	**Hf**, **Os**, **Hg**.
In-house K	*w* = 1% TMAH	**Cl**, **Br**, **I**.
In-house L	*w* = 2% HNO_3	**Co**, **In**
Single Element	*w = 2%* HNO_3	***Si***

Table S1b: Composition of multi-element standards used for evaluation of on-mass removal of doubly-charged and oxide-based interferences. Analytes measured in each standard are highlighted in bold. Elements measured as single element standard solutions are given in italics.

Standard	Matrix	Analytes
AHF-CAL-7	*w* = 2% HNO_3	Y, La, Ce, Pr, Nd, Sm, Eu, Gd, Tb, Dy, Ho, Er, Tm, Yb, Lu, **Th**, **U**.
In-house G	*w* = 2% HNO_3	**Li**, **K**, **Ga**, **Ag**, **Cs**, **Tl**.
In-house K	*w* = 1% TMAH	**Cl**, **Br**, **I**.
In-house L	*w* = 2% HNO_3	**Co**, **In**
In-house M	*w* = 2% HNO_3	**Y**, **La**, **Pr**, **Tb**¸ **Ho**, **Tm**
In-house N	*w* = 2% HNO_3	**Ca**, **V**, **Sb**, **Sm**, **Lu**, **Pb**
In-house O	*w* = 2% HNO_3	**Sc**, **Nb**, **Ce**, **Eu**, **Yb**, **Ta**
In-house P	*w* = 2% HNO_3	**Na**, **Cu**, **Rb**, **Sn**, **Nd**, **Bi**
In-house Q	*w* = 2% HNO_3	**Fe**, **Rh**, **Te**, **Er**, **Au**
In-house R	*w* = 2% HNO_3	**Mg**, **Mn**, **Ba**, **Dy**, **Pt**
In-house S	*w* = 2% HNO_3	**Ti**, **Sr**, **Cd**, **Gd**, **Re**
In-house T	*w* = 2% HNO_3	**Cr**, **Ni**, **Zr**
In-house U	*w* = 2% HNO_3	**Zn**, **Mo**
Single Element	*w = 2%* HNO_3	***Be, B, Al, Si, P, S, Ge, As, Se***
Single Element	*w = 2% HCl*	***Ru, Pd, Hf, W, Os, Ir, Hg***

Table S2: Maximum obtained formation of selected product ions for 73 analytes using N_2O reaction gas. Flow rates of N_2O provided are at the optimum formation using a Perkin Elmer NexION 5000.

Element	Isotope	MN^+ (+14 amu)		MO^+ (+16 amu)		MNO^+ (+30 amu)		MO_2^+ (+32 amu)		MN_2O^+ (+44 amu)		MNO_2^+ (+46 amu)	
		Formation (%)	Flow Rate (mL min^{-1})	Formation (%)	Flow Rate (mL min^{-1})	Formation (%)	Flow Rate (mL min^{-1})	Formation (%)	Flow Rate (mL min^{-1})	Formation (%)	Flow Rate (mL min^{-1})	Formation (%)	Flow Rate (mL min^{-1})
Li	7	<0.01	-	<0.01	-	<0.01	-	<0.01	-	<0.01	-	<0.01	-
Be	9	0.03	0.1	5.5	0.1	0.02	0.1	7.8	0.3	0.07	0.2	<0.01	-
B	11	<0.01	-	0.98	0.1	<0.01	-	0.14	0.1	0.01	0.1	<0.01	-
Na	23	<0.01	-	<0.01	-	<0.01	-	<0.01	-	<0.01	-	<0.01	-
Mg	24	<0.01	-	13	0.5	<0.01	-	<0.01	-	0.06	0.5	<0.01	-
Al	27	<0.01	-	1.5	0.1	<0.01	-	0.48	0.2	0.06	0.6	<0.01	-
Si	29	0.47	0.1	29	0.2	0.20	0.3	11	0.3	0.05	0.3	<0.01	-
P	31	5.2	0.1	28	0.4	0.09	0.4	0.01	0.2	<0.01	-	<0.01	-
S	34	12	0.5	11	0.4	0.02	0.5	5.9	0.7	<0.01	-	<0.01	-
Cl	35	0.06	0.1	0.69	0.2	<0.01	-	<0.01	-	<0.01	-	<0.01	-
K	39	<0.01	-	<0.01	-	<0.01	-	<0.01	-	<0.01	-	<0.01	-
Ca	44	<0.01	-	51	0.4	0.09	0.4	0.02	0.6	<0.01	-	<0.01	-
Sc	45	0.57	0.3	67	0.4	0.10	0.3	0.28	0.5	<0.01	-	<0.01	-
Ti	47	2.6	0.4	37	0.3	0.07	0.3	29	0.6	<0.01	-	<0.01	-
V	51	0.52	0.5	25	0.4	0.12	0.3	17	0.5	<0.01	-	<0.01	-
Cr	52	0.09	0.4	1.9	0.4	<0.01	-	0.82	0.5	0.06	0.5	<0.01	-
Mn	55	<0.01	-	6.6	0.4	<0.01	-	<0.01	-	0.02	1.8	<0.01	-
Fe	57	0.02	0.4	42	0.7	<0.01	-	0.09	0.4	<0.01	-	<0.01	-
Co	59	0.01	0.4	9.4	0.5	0.02	0.4	<0.01	-	0.09	0.3	<0.01	-
Ni	60	<0.01	-	3.4	0.4	<0.01	-	<0.01	-	0.20	0.4	<0.01	-
Cu	63	<0.01	-	0.15	0.2	<0.01	-	<0.01	-	0.89	0.5	<0.01	-
Zn	66	<0.01	-	1.6	0.4	<0.01	-	<0.01	-	0.07	1.8	<0.01	-
Ga	69	<0.01	-	<0.01	-	<0.01	-	<0.01	-	<0.01	-	<0.01	-
Ge	74	0.08	0.5	68	0.4	0.02	0.4	0.03	0.5	<0.01	-	<0.01	-
As	75	0.39	0.1	37	0.4	<0.01	-	0.08	0.5	0.03	0.5	<0.01	-
Se	82	3.0	0.6	11	1.3	<0.01	-	0.18	0.7	<0.01	-	<0.01	-
Br	81	0.24	0.4	57	0.5	<0.01	-	<0.01	-	0.07	0.5	<0.01	-
Rb	85	<0.01	-	<0.01	-	<0.01	-	<0.01	-	<0.01	-	<0.01	-
Sr	88	<0.01	-	56	0.5	0.12	0.5	<0.01	-	<0.01	-	<0.01	-
Y	89	1.7	0.4	54	0.4	0.04	0.2	0.39	0.5	<0.01	-	<0.01	-
Zr	90	16	0.4	20	0.1	0.47	0.3	43	0.5	<0.01	-	0.03	0.6
Nb	93	8.1	0.2	16	0.1	12	0.5	38	0.5	<0.01	-	<0.01	-
Mo	98	1.8	0.5	3.4	0.1	0.47	0.5	1.3	0.3	0.07	1.7	<0.01	-
Ru	101	0.47	0.7	1.7	0.2	0.10	0.7	0.56	0.3	0.09	1.3	<0.01	-
Rh	103	0.08	0.6	0.67	0.6	0.03	0.7	<0.01	-	0.11	1.6	<0.01	-
Pd	105	<0.01	-	0.13	0.6	<0.01	-	<0.01	-	0.09	1.0	<0.01	-
Ag	107	<0.01	-	<0.01	-	<0.01	-	<0.01	-	0.11	1.7	<0.01	-

(Table S2 continued)

Element	Isotope	MO_3^+ (+48 amu)		$MN_2O_2^+$ (+60 amu)		MO_4^+ (+64 amu)		$MN_2O.O_2$ (+76 amu)		$MNO.O_4$ (+94 amu)		$M(N_2O)_2.O_2$	
		Formation (%)	Flow Rate (mL min^{-1})	Formation (%)	Flow Rate (mL min^{-1})	Formation (%)	Flow Rate (mL min^{-1})	Formation (%)	Flow Rate (mL min^{-1})	Formation (%)	Flow Rate (mL min^{-1})	Formation (%)	Flow Rate (mL min^{-1})
Li	7	<0.01	-	<0.01	-	<0.01	-	<0.01	-	<0.01	-	<0.01	-
Be	9	0.10	0.3	2.7	0.4	<0.01	-	1.8	0.6	0.04	1.2	0.12	0.9
B	11	<0.01	-	0.05	0.4	<0.01	-	<0.01	-	<0.01	-	<0.01	-
Na	23	<0.01	-	<0.01	-	<0.01	-	<0.01	-	<0.01	-	<0.01	-
Mg	24	<0.01	-	0.26	1.2	<0.01	-	<0.01	-	<0.01	-	<0.01	-
Al	27	0.69	0.4	0.04	0.3	0.06	0.6	0.07	0.4	<0.01	-	<0.01	-
Si	29	2.8	0.4	0.62	0.4	0.03	0.4	0.28	0.6	0.08	1.2	0.05	1.0
P	31	<0.01	-	<0.01	-	<0.01	-	<0.01	-	<0.01	-	<0.01	-
S	34	<0.01	-	<0.01	-	<0.01	-	<0.01	-	<0.01	-	<0.01	-
Cl	35	<0.01	-	<0.01	-	<0.01	-	<0.01	-	<0.01	-	<0.01	-
K	39	<0.01	-	<0.01	-	<0.01	-	<0.01	-	<0.01	-	<0.01	-
Ca	44	<0.01	-	0.16	1.3	<0.01	-	<0.01	-	<0.01	-	<0.01	-
Sc	45	<0.01	-	0.32	1.0	<0.01	-	<0.01	-	<0.01	-	<0.01	-
Ti	47	<0.01	-	0.02	0.3	<0.01	-	1.8	0.8	1.4	1.3	2.1	1.1
V	51	<0.01	-	0.11	0.8	<0.01	-	1.3	0.8	1.2	1.8	2.4	1.3
Cr	52	0.59	0.7	0.05	0.7	<0.01	-	0.08	0.8	0.04	1.4	0.07	1.1
Mn	55	<0.01	-	0.19	1.3	<0.01	-	<0.01	-	<0.01	-	<0.01	-
Fe	57	0.14	0.7	1.4	1.0	<0.01	-	<0.01	-	<0.01	-	<0.01	-
Co	59	<0.01	-	0.54	1.3	<0.01	-	<0.01	-	<0.01	-	<0.01	-
Ni	60	<0.01	-	0.14	1.3	<0.01	-	<0.01	-	<0.01	-	<0.01	-
Cu	63	<0.01	-	<0.01	-	<0.01	-	<0.01	-	<0.01	-	<0.01	-
Zn	66	<0.01	-	0.10	1.3	<0.01	-	<0.01	-	<0.01	-	<0.01	-
Ga	69	<0.01	-	<0.01	-	<0.01	-	<0.01	-	<0.01	-	<0.01	-
Ge	74	0.01	1.0	0.19	1.0	<0.01	-	<0.01	-	<0.01	-	<0.01	-
As	75	<0.01	-	0.03	0.5	<0.01	-	<0.01	-	<0.01	-	<0.01	-
Se	82	<0.01	-	0.03	2.0	<0.01	-	<0.01	-	<0.01	-	<0.01	-
Br	81	<0.01	-	<0.01	-	<0.01	-	<0.01	-	<0.01	-	<0.01	-
Rb	85	<0.01	-	<0.01	-	<0.01	-	<0.01	-	<0.01	-	<0.01	-
Sr	88	<0.01	-	0.18	1.6	<0.01	-	<0.01	-	<0.01	-	<0.01	-
Y	89	<0.01	-	0.18	1.2	<0.01	-	<0.01	-	<0.01	-	<0.01	-
Zr	90	0.01	0.8	<0.01	-	<0.01	-	1.8	0.8	1.4	1.4	1.4	1.1
Nb	93	0.39	0.5	<0.01	-	<0.01	-	2.0	0.9	2.7	1.3	2.1	1.1
Mo	98	3.8	0.4	<0.01	-	<0.01	-	<0.01	-	0.03	0.8	<0.01	-
Ru	101	2.1	0.5	<0.01	-	1.5	1.0	<0.01	-	<0.01	-	<0.01	-
Rh	103	<0.01	-	0.01	1.6	<0.01	-	<0.01	-	<0.01	-	<0.01	-
Pd	105	<0.01	-	<0.01	-	<0.01	-	<0.01	-	<0.01	-	<0.01	-
Ag	107	<0.01	-	<0.01	-	<0.01	-	<0.01	-	<0.01	-	<0.01	-

(Table S2 continued)

Element	Isotope	MN^+ (+14 amu)		MO^+ (+16 amu)		MNO^+ (+30 amu)		MO_2^+ (+32 amu)		MN_2O^+ (+44 amu)		MNO_2^+ (+46 amu)	
		Formation (%)	Flow Rate (mL min^{-1})	Formation (%)	Flow Rate (mL min^{-1})	Formation (%)	Flow Rate (mL min^{-1})	Formation (%)	Flow Rate (mL min^{-1})	Formation (%)	Flow Rate (mL min^{-1})	Formation (%)	Flow Rate (mL min^{-1})
Cd	114	<0.01	-	0.31	0.8	<0.01	-	<0.01	-	0.04	1.9	<0.01	-
In	115	<0.01	-	<0.01	-	<0.01	-	<0.01	-	<0.01	-	<0.01	-
Sn	118	<0.01	-	9.8	0.6	<0.01	-	<0.01	-	0.01	0.9	<0.01	-
Sb	121	0.03	0.5	7.8	0.7	<0.01	-	<0.01	-	0.03	0.9	<0.01	-
Te	128	0.19	0.9	3.5	0.9	<0.01	-	<0.01	-	<0.01	-	<0.01	-
I	127	0.07	0.8	1.8	0.9	<0.01	-	<0.01	-	0.01	1.0	<0.01	-
Cs	133	<0.01	-	<0.01	-	<0.01	-	<0.01	-	<0.01	-	<0.01	-
Ba	138	0.01	0.8	70	0.6	0.33	0.6	0.08	0.8	<0.01	-	<0.01	-
La	139	11	0.5	51	0.5	0.06	0.5	0.06	1.0	<0.01	-	<0.01	-
Ce	140	16	0.4	44	0.6	0.01	0.5	0.40	0.7	<0.01	-	<0.01	-
Pr	141	4.8	0.6	53	0.6	0.03	0.5	0.04	0.9	<0.01	-	<0.01	-
Nd	143	1.5	0.5	59	0.6	0.04	0.5	0.02	0.9	<0.01	-	<0.01	-
Sm	147	0.05	0.7	63	0.6	0.04	0.5	0.02	1.0	<0.01	-	<0.01	-
Eu	153	<0.01	-	68	0.7	0.09	0.7	0.03	0.9	<0.01	-	<0.01	-
Gd	155	2.5	0.5	66	0.6	0.04	0.5	0.13	0.9	<0.01	-	<0.01	-
Tb	159	2.1	0.6	68	0.7	0.02	0.4	0.62	0.8	<0.01	-	<0.01	-
Dy	163	0.22	0.8	66	0.8	<0.01	-	0.06	0.7	<0.01	-	<0.01	-
Ho	165	0.24	0.7	61	0.8	<0.01	-	0.17	0.7	<0.01	-	<0.01	-
Er	166	0.24	0.6	62	0.7	<0.01	-	0.56	0.9	<0.01	-	<0.01	-
Tm	169	0.02	0.7	53	0.7	<0.01	-	0.15	0.7	<0.01	-	<0.01	-
Yb	174	<0.01	-	48	0.8	<0.01	-	0.01	0.9	<0.01	-	<0.01	-
Lu	175	0.13	0.2	76	0.7	<0.01	-	2.8	0.6	<0.01	-	<0.01	-
Hf	180	10	0.1	23	0.1	1.7	0.2	81	0.5	<0.01	-	0.02	0.4
Ta	181	7.7	0.1	17	0.1	4.3	0.2	54	0.5	<0.01	-	<0.01	-
W	184	8.4	0.1	19	0.2	5.0	0.2	7.8	0.3	<0.01	-	0.27	0.3
Re	187	3.2	0.3	3.2	0.2	0.73	0.4	1.0	0.4	<0.01	-	0.39	0.4
Os	189	7.5	0.3	8.5	0.2	2.6	0.4	4.1	0.4	<0.01	-	2.4	0.4
Ir	193	2.5	0.2	14	0.2	0.58	0.3	9.3	0.3	0.02	0.2	2.5	0.6
Pt	195	0.44	0.7	12	0.2	0.05	0.4	96	0.7	0.07	0.2	0.05	0.7
Au	197	<0.01	-	1.5	0.7	<0.01	-	<0.01	-	1.0	0.7	<0.01	-
Hg	202	<0.01	-	0.05	0.8	<0.01	-	<0.01	-	0.13	1.8	<0.01	-
Tl	205	<0.01	-	<0.01	-	<0.01	-	<0.01	-	<0.01	-	<0.01	-
Pb	208	<0.01	-	3.4	1.0	<0.01	-	<0.01	-	<0.01	-	<0.01	-
Bi	209	<0.01	-	1.2	1.3	<0.01	-	<0.01	-	<0.01	-	<0.01	-
Th	232	22	0.2	23	0.1	10	0.8	63	0.7	<0.01	-	0.03	0.6
U	238	15	0.2	27	0.2	9.8	0.9	51	0.9	<0.01	-	<0.01	-

(Table S2 continued)

Element	Isotope	MO_3^+ (+48 amu)		$MN_2O_2^+$ (+60 amu)		MO_4^+ (+64 amu)		$MN_2O.O_2$ (+76 amu)		$MNO.O_4$ (+94 amu)		$M(N_2O)_2.O_2$	
		Formation (%)	Flow Rate (mL min^{-1})	Formation (%)	Flow Rate (mL min^{-1})	Formation (%)	Flow Rate (mL min^{-1})	Formation (%)	Flow Rate (mL min^{-1})	Formation (%)	Flow Rate (mL min^{-1})	Formation (%)	Flow Rate (mL min^{-1})
Cd	114	<0.01	-	<0.01	-	<0.01	-	<0.01	-	<0.01	-	<0.01	-
In	115	<0.01	-	<0.01	-	<0.01	-	<0.01	-	<0.01	-	<0.01	-
Sn	118	<0.01	-	0.01	1.0	<0.01	-	<0.01	-	<0.01	-	<0.01	-
Sb	121	<0.01	-	<0.01	-	<0.01	-	<0.01	-	<0.01	-	<0.01	-
Te	128	<0.01	-	<0.01	-	<0.01	-	<0.01	-	<0.01	-	<0.01	-
I	127	<0.01	-	<0.01	-	<0.01	-	<0.01	-	<0.01	-	<0.01	-
Cs	133	<0.01	-	<0.01	-	<0.01	-	<0.01	-	<0.01	-	<0.01	-
Ba	138	<0.01	-	0.06	1.7	<0.01	-	<0.01	-	<0.01	-	<0.01	-
La	139	<0.01	-	0.06	1.7	<0.01	-	<0.01	-	<0.01	-	<0.01	-
Ce	140	<0.01	-	0.08	1.7	<0.01	-	<0.01	-	<0.01	-	<0.01	-
Pr	141	<0.01	-	0.09	1.7	<0.01	-	<0.01	-	<0.01	-	<0.01	-
Nd	143	<0.01	-	0.17	1.7	<0.01	-	<0.01	-	<0.01	-	<0.01	-
Sm	147	<0.01	-	0.31	1.7	<0.01	-	<0.01	-	<0.01	-	<0.01	-
Eu	153	<0.01	-	0.33	1.6	<0.01	-	<0.01	-	<0.01	-	<0.01	-
Gd	155	<0.01	-	0.28	1.2	<0.01	-	<0.01	-	<0.01	-	<0.01	-
Tb	159	<0.01	-	0.56	1.3	<0.01	-	0.03	1.2	<0.01	-	<0.01	-
Dy	163	<0.01	-	0.53	1.4	<0.01	-	<0.01	-	<0.01	-	<0.01	-
Ho	165	<0.01	-	0.81	1.4	<0.01	-	<0.01	-	<0.01	-	<0.01	-
Er	166	<0.01	-	0.49	1.4	<0.01	-	0.02	1.0	<0.01	-	-	-
Tm	169	<0.01	-	0.58	1.5	<0.01	-	<0.01	-	<0.01	-	-	-
Yb	174	<0.01	-	0.39	1.4	<0.01	-	<0.01	-	<0.01	-	-	-
Lu	175	<0.01	-	0.65	1.2	<0.01	-	0.09	0.9	0.05	1.4	-	-
Hf	180	0.02	0.4	<0.01	-	<0.01	-	7.7	0.7	5.7	1.3	-	-
Ta	181	4.9	0.4	<0.01	-	<0.01	-	6.5	0.7	12	1.1	-	-
W	184	29	0.4	0.03	0.3	<0.01	-	<0.01	-	0.33	0.7	-	-
Re	187	3.9	0.5	<0.01	-	0.48	0.5	<0.01	-	0.06	0.9	-	-
Os	189	10	0.5	0.02	0.4	50	0.9	0.06	1.0	<0.01	-	-	-
Ir	193	87	0.8	0.18	0.6	0.06	1.8	0.29	1.0	-	-	-	-
Pt	195	0.20	0.7	0.23	0.7	<0.01	-	2.6	1.7	-	-	-	-
Au	197	<0.01	-	0.08	1.6	<0.01	-	<0.01	-	-	-	-	-
Hg	202	<0.01	-	<0.01	-	<0.01	-	<0.01	-	-	-	-	-
Tl	205	<0.01	-	<0.01	-	<0.01	-	<0.01	-	-	-	-	-
Pb	208	<0.01	-	<0.01	-	<0.01	-	<0.01	-	-	-	-	-
Bi	209	<0.01	-	<0.01	-	<0.01	-	<0.01	-	-	-	-	-
Th	232	0.03	1.0	-	-	-	-	-	-	-	-	-	-
U	238	-	-	-	-	-	-	-	-	-	-	-	-

Table S3: Maximum obtained formation of selected product ions for 73 analytes using O_2 reaction gas. Flow rates of O_2 provided are at the optimum formation using a Perkin Elmer NexION 5000.

Element	Isotope	MO^+ (+16 amu)		MO_2^+ (+32 amu)		MO_3^+ (+48 amu)	
		Formation (%)	Flow Rate (mL min^{-1})	Formation (%)	Flow Rate (mL min^{-1})	Formation (%)	Flow Rate (mL min^{-1})
Li	7	<0.01	-	<0.01	-	<0.01	-
Be	9	0.21	0.3	<0.01	-	<0.01	-
B	11	2.1	0.2	0.07	0.2	<0.01	-
Na	23	<0.01	-	<0.01	-	<0.01	-
Mg	24	0.41	0.3	<0.01	-	<0.01	-
Al	27	0.43	0.2	<0.01	-	<0.01	-
Si	29	3.2	0.3	0.06	0.4	<0.01	-
P	31	10	0.4	0.02	0.4	<0.01	-
S	34	7.6	0.3	0.02	0.4	<0.01	-
Cl	35	0.73	0.2	<0.01	-	<0.01	-
K	39	<0.01	-	<0.01	-	<0.01	-
Ca	44	1.1	0.3	0.04	0.4	<0.01	-
Sc	45	8.9	0.4	0.10	0.5	<0.01	-
Ti	47	10	0.4	0.12	0.5	<0.01	-
V	51	5.5	0.4	0.07	0.5	<0.01	-
Cr	52	1.5	0.3	<0.01	-	<0.01	-
Mn	55	0.99	0.3	<0.01	-	<0.01	-
Fe	57	1.9	0.3	<0.01	-	<0.01	-
Co	59	1.5	0.3	<0.01	-	<0.01	-
Ni	60	0.52	0.3	<0.01	-	<0.01	-
Cu	63	0.06	0.3	<0.01	-	<0.01	-
Zn	66	0.11	0.3	<0.01	-	<0.01	-
Ga	69	<0.01	-	<0.01	-	<0.01	-
Ge	74	2.3	0.3	0.04	0.5	<0.01	-
As	75	8.3	0.4	<0.01	-	<0.01	-
Se	82	3.6	0.4	<0.01	-	<0.01	-
Br	81	1.2	0.3	<0.01	-	<0.01	-
Rb	85	<0.01	-	<0.01	-	<0.01	-
Sr	88	1.0	0.3	0.04	0.5	<0.01	-
Y	89	13	0.3	0.20	0.4	<0.01	-
Zr	90	17	0.3	1.7	0.5	0.01	0.9
Nb	93	12	0.3	5.1	0.6	<0.01	-
Mo	98	6.7	0.3	3.6	0.7	<0.01	-
Ru	101	3.1	0.4	0.03	0.6	<0.01	-
Rh	103	0.46	0.4	<0.01	-	<0.01	-
Pd	105	0.04	0.4	<0.01	-	<0.01	-
Ag	107	<0.01	-	<0.01	-	<0.01	-
Cd	114	0.02	0.3	<0.01	-	<0.01	-
In	115	<0.01	-	<0.01	-	<0.01	-
Sn	118	0.46	0.3	0.01	0.8	<0.01	-
Sb	121	4.8	0.5	<0.01	-	<0.01	-
Te	128	1.9	0.4	<0.01	-	<0.01	-
I	127	0.51	0.5	<0.01	-	<0.01	-
Cs	133	<0.01	-	<0.01	-	<0.01	-
Ba	138	1.8	0.4	0.07	0.6	<0.01	-
La	139	20	0.3	0.27	0.4	<0.01	-
Ce	140	24	0.3	0.22	0.4	<0.01	-
Pr	141	25	0.3	0.11	0.4	<0.01	-
Nd	143	26	0.3	0.10	0.4	<0.01	-
Sm	147	18	0.4	0.07	0.6	<0.01	-
Eu	153	5.0	0.4	0.08	0.7	<0.01	-
Gd	155	21	0.3	0.23	0.4	<0.01	-
Tb	159	27	0.3	0.20	0.5	<0.01	-
Dy	163	28	0.3	0.09	0.5	<0.01	-
Ho	165	27	0.3	0.12	0.5	<0.01	-
Er	166	29	0.3	0.16	0.4	<0.01	-
Tm	169	21	0.4	0.08	0.5	<0.01	-
Yb	174	4.3	0.5	0.05	0.7	<0.01	-
Lu	175	33	0.4	0.28	0.5	<0.01	-
Hf	180	37	0.3	7.1	0.5	0.03	0.6
Ta	181	25	0.2	13	0.5	<0.01	-
W	184	26	0.2	15	0.5	0.10	0.7
Re	187	12	0.4	6.9	0.7	1.5	0.9
Os	189	13	0.4	3.9	0.8	0.07	0.9
Ir	193	3.6	0.5	0.07	1.0	<0.01	-
Pt	195	0.46	0.6	<0.01	-	<0.01	-
Au	197	0.04	0.4	<0.01	-	<0.01	-
Hg	202	<0.01	-	<0.01	-	<0.01	-
Tl	205	<0.01	-	<0.01	-	<0.01	-
Pb	208	0.03	0.5	<0.01	-	<0.01	-
Bi	209	0.25	0.7	<0.01	-	<0.01	-
Th	232	39	0.2	13	0.5	<0.01	-
U	238	31	0.1	22	0.4	-	-

Table S4: Comparison of determined limit of detection (LOD) and background equivalence concentration (BEC) for standard mode and mass-shift determinations with N_2O reaction gas using a Perkin Elmer NexION 5000. LODs were calculated based on 3 times the standard deviation of the blank and expressed in ng g^{-1}. BECs were calculated by division of the blank signal with the calibration slope and expressed in ng g^{-1}. Improvement in LOD and BEC are given as a factor of how much lower these parameters are when compared to standard mode. Values are presented to 2 significant figures.

Element	Isotope	Standard Mode		N_2O Mode						
		LOD (ng g^{-1})	BEC (ng g^{-1})	Measured Product Ion	Measured Mass	Gas Flow Rate (mL min^{-1})	LOD (ng g^{-1})	LOD Improvement	BEC (ng g^{-1})	BEC Improvement
Be	9	0.003 9	0.003 4	BeO_2	41	0.3	0.011	0.34	0.003 8	0.90
B	11	0.59	5.9	BO	27	0.1	0.29	2.0	3.7	1.6
Mg	24	0.001 7	0.016	MgO	40	0.5	0.002 2	0.77	0.019	0.84
Al	27	0.001 6	0.019	AlO	43	0.1	0.014	0.113	0.022	0.87
Si	28	19	270	SiO_2	60	0.3	0.14	130	2.1	130
Si	29	35	610	SiO	45	0.2	51	0.68	1000	0.59
P	31	3.4	20	PO	47	0.4	0.062	56	0.43	47
S	34	47	1000	SN	48	0.5	1.3	35	1.9	530
Cl	35	7.8	210	ClO	51	0.2	28	46	170	120
Ca	44	0.44	18	CaO	60	0.4	0.12	0.28	2.8	1.2
Sc	45	0.002 7	0.054	ScO	61	0.4	0.000 15	3.64	0.000 096	6.4
Ti	47	0.000 86	0.006 3	TiO	63	0.3	0.000 33	18	0.002 9	560
Ti	47	0.000 86	0.006 3	TiO_2	79	0.6	0.000 31	2.6	0.000 36	2.2
V	51	0.000 36	0.002 1	VO	67	0.4	0.000 18	2.7	0.000 024	18
Cr	52	0.15	10	CrO	68	0.4	0.005 4	2.0	0.002 3	88
Mn	55	0.001 3	0.015	MnO	71	0.4	0.000 71	27	0.000 83	4500
Fe	57	3.5	65	FeO	73	0.7	0.010	1.8	0.007 8	19
Co	59	0.000 79	0.004 8	CoO	75	0.5	0.000 25	340	0.000 092	8300
Ni	60	0.002 1	0.007 9	NiO	76	0.4	0.009 5	3.1	0.006 1	52
Cu	63	0.000 50	0.005 2	CuN_2O	107	0.5	0.007 9	0.22	0.003 1	1.3
Zn	66	0.004 0	0.019	ZnO	82	0.4	0.023	0.064	0.010	1.7
Ge	74	0.001 9	0.004 8	GeO	90	0.4	0.000 082	0.17	0.000 011	1.9
As	75	0.000 34	0.000 53	AsO	91	0.4	0.000 60	23	0.000 13	430
Se	82	0.036	0.16	SeO	98	1.3	0.012	0.57	0.001 7	4.1
Br	81	5.0	130	BrO	97	0.5	1.5	2.9	77	97
Sr	88	0.000 21	0.001 1	SrO	104	0.5	0.000 43	3.4	0.001 0	1.7
Y	89	0.000 033	0.000 036	YO	105	0.4	0.000 078	0.49	0.000 026	1.0
Zr	90	0.000 59	0.000 29	ZrO_2	122	0.5	0.000 038	0.42	0.000 026	1.4
Nb	93	0.000 12	0.000 070	NbO_2	125	0.5	0.000 46	16	0.000 13	11
Mo	98	0.000 33	0.000 25	MoO_3	146	0.4	0.001 3	0.25	0.000 17	0.53
Ru	101	0.001 4	0.006 7	RuO_3	149	0.5	0.003 5	0.25	0.000 48	1.4
Rh	103	0.000 12	0.000 17	RhO	119	0.6	0.002 5	0.41	0.000 54	14
Pd	105	0.000 29	0.000 38	PdO	121	0.6	0.066	0.049	0.014	0.32
Ag	107	0.000 51	0.001 7	AgN_2O	151	1.7	0.062	0.004 4	0.008 4	0.027

(Table S4 continued)

Element	Isotope	Standard Mode		N₂O DRC Mode						
		LOD (ng g^{-1})	BEC (ng g^{-1})	Measured Product Ion	Measured Mass	Gas Flow Rate (mL min^{-1})	LOD (ng g^{-1})	LOD Improvement	BEC (ng g^{-1})	BEC Improvement
Cd	114	0.000 26	0.000 23	CdO	130	0.8	0.024	0.011	0.003 2	0.071
Sn	118	0.000 34	0.000 50	SnO	134	0.6	0.001 7	0.19	0.000 53	0.95
Sb	121	0.000 72	0.000 96	SbO	137	0.7	0.003 8	0.19	0.001 6	0.59
Te	128	0.004 8	0.014	TeO	144	0.9	0.007	0.69	0.001 5	9.4
I	127	0.016	0.71	IO	143	0.9	0.14	0.11	0.79	0.90
Ba	138	0.000 22	0.000 19	BaO	154	0.6	0.000 33	0.65	0.000 53	0.36
La	139	0.000 055	0.000 034	LaO	155	0.5	0.000 13	0.44	0.000 067	0.51
Ce	140	0.000 083	0.000 040	CeO	156	0.6	0.000 29	0.284	0.000 12	0.34
Pr	141	0.000 015	0.000 027	PrO	157	0.6	0.000 059	0.26	0.000 035	0.77
Nd	143	0.000 47	0.000 14	NdO	159	0.6	0.000 29	1.6	0.000 087	1.6
Sm	147	0.000 13	0.000 029	SmO	163	0.6	0.000 17	0.79	0.000 023	1.3
Eu	153	0.000 032	0.000 004	EuO	169	0.7	0.000 047	0.68	0.000 006	0.68
Gd	155	0.000 77	0.001 4	GdO	171	0.6	0.001 6	0.48	0.001 2	1.2
Tb	159	0.000 13	0.000 29	TbO	175	0.7	0.000 16	0.83	0.000 25	1.1
Dy	163	0.001 5	0.001 4	DyO	179	0.8	0.001 3	1.2	0.000 85	1.6
Ho	165	0.000 17	0.000 27	HoO	181	0.8	0.000 22	0.80	0.000 25	1.1
Er	166	0.000 81	0.000 60	ErO	182	0.7	0.000 47	1.7	0.000 83	0.72
Tm	169	0.000 11	0.000 078	TmO	185	0.7	0.000 12	0.94	0.000 061	1.3
Yb	174	0.000 16	0.000 067	YbO	190	0.8	0.000 23	0.73	0.000 031	2.2
Lu	175	0.000 11	0.000 062	LuO	191	0.7	0.000 051	2.3	0.000 007	9.1
Hf	180	0.000 10	0.000 014	HfO_2	212	0.5	0.000 16	0.64	0.000 035	0.40
Ta	181	0.000 094	0.000 019	TaO_2	213	0.5	0.000 21	0.46	0.000 035	0.54
W	184	0.000 26	0.000 087	WO_3	232	0.4	0.000 26	1.0	0.000 035	2.4
Re	187	0.000 11	0.000 044	ReO_3	235	0.5	0.001 2	0.093	0.000 26	0.17
Os	189	0.000 26	0.000 051	OsO_4	253	0.9	0.000 25	1.0	0.000 034	1.5
Ir	193	0.000 42	0.000 46	IrO_3	241	0.8	0.000 074	5.6	0.000 023	20
Pt	195	0.001 4	0.000 85	PtO_2	227	0.7	0.001 1	1.3	0.001 0	0.83
Au	197	0.002 0	0.004 4	AuO	213	0.7	0.016	0.12	0.004 5	0.99
Hg	202	0.006 0	0.006 1	HgN_2O	246	1.8	0.88	0.006 8	0.12	0.051
Pb	208	0.000 15	0.000 020	PbO	224	1.0	0.002 2	0.067	0.000 30	0.067
Bi	209	0.000 049	0.000 067	BiO	225	1.3	0.004 1	0.012	0.000 56	0.012
Th	232	0.000 084	0.000 026	ThO_2	264	0.7	0.000 10	0.842	0.000 014	1.9
U	238	0.000 063	0.000 014	UO_2	270	0.9	0.000 19	0.33	0.000 053	0.26

Table S5: Comparison of determined limit of detection (LOD) and background equivalence concentration (BEC) for standard mode and mass-shift determinations with O_2 reaction gas using a Perkin Elmer NexION 5000. LODs were calculated based on 3 times the standard deviation of the blank and expressed in ng g^{-1}. BECs were calculated by division of the blank signal with the calibration slope and expressed in ng g^{-1}. Improvement in LOD and BEC are given as a factor of how much lower these parameters are when compared to standard mode. Values are presented to 2 significant figures.

Element	Isotope	Standard Mode		O_2 Mode						
		LOD (ng g^{-1})	BEC (ng g^{-1})	Measured Product Ion	Measured Mass	Gas Flow Rate (mL min^{-1})	LOD (ng g^{-1})	LOD Improvement	BEC (ng g^{-1})	BEC Improvement
Be	9	0.003 9	0.003 4	BeO	25	0.3	0.11	0.035	0.015	0.22
B	11	0.59	5.9	BO	27	0.2	0.58	1.0	2.4	2.5
Mg	24	0.001 7	0.016	MgO	40	0.3	0.073	0.023	0.069	0.22
Al	27	0.001 6	0.019	AlO	43	0.2	0.036	0.044	0.019	0.98
Si	28	19	270	SiO	44	0.3	0.39	18	5.9	63
Si	29	35	610	SiO	45	0.3	2.0	48	9.7	46
P	31	3.4	20	PO	47	0.4	0.11	31	0.4	51
S	34	47	1000	SO	50	0.3	0.51	92	3.0	330
Cl	35	7.8	210	ClO	51	0.2	27	0.30	73	2.8
Ca	44	0.44	18	CaO	60	0.3	0.97	0.46	2.0	8.7
Sc	45	0.002 7	0.054	ScO	61	0.4	0.000 75	3.6	0.000 39	140
Ti	47	0.000 86	0.006 3	TiO	63	0.4	0.000 92	0.93	0.000 81	7.9
V	51	0.000 36	0.002 1	VO	67	0.4	0.000 53	0.67	0.000 072	29
Cr	52	0.15	10	CrO	68	0.3	0.004 4	34	0.001 8	5700
Mn	55	0.001 3	0.015	MnO	71	0.3	0.002 4	0.56	0.000 93	17
Fe	57	3.5	65	FeO	73	0.3	0.036	98	0.015	4200
Co	59	0.000 79	0.004 8	CoO	75	0.3	0.001 1	0.74	0.000 23	21
Ni	60	0.002 1	0.007 9	NiO	76	0.3	0.032	0.067	0.011	0.75
Cu	63	0.000 50	0.005 2	CuO	79	0.3	0.045	0.011	0.006 1	0.85
Zn	66	0.004 0	0.019	ZnO	82	0.3	0.11	0.036	0.015	1.3
Ge	74	0.001 9	0.004 8	GeO	90	0.3	0.002 4	0.78	0.000 33	15
As	75	0.000 34	0.000 53	AsO	91	0.4	0.002 1	0.16	0.000 28	1.9
Se	82	0.036	0.16	SeO	98	0.4	0.036	0.98	0.005	33
Br	81	5.0	130	BrO	97	0.3	4.0	1.2	21	6.3
Sr	88	0.000 21	0.001 1	SrO	104	0.3	0.003 4	0.062	0.001 7	0.63
Y	89	0.000 033	0.000 036	YO	105	0.3	0.000 094	0.35	0.000 037	0.96
Zr	90	0.000 59	0.000 29	ZrO	106	0.3	0.000 44	1.4	0.000 13	2.3
Nb	93	0.000 12	0.000 070	NbO	109	0.3	0.000 23	0.51	0.000 16	0.45
Mo	98	0.000 33	0.000 25	MoO	114	0.3	0.000 73	0.45	0.000 099	2.5
Ru	101	0.001 4	0.006 7	RuO	117	0.4	0.010	0.14	0.006 2	1.1
Rh	103	0.000 12	0.000 17	RhO	119	0.4	0.002 9	0.042	0.000 40	0.43
Pd	105	0.000 29	0.000 38	PdO	121	0.4	0.19	0.001 5	0.042	0.009 2

(Table S5 continued)

Element	Isotope	Standard Mode		O$_2$ DRC Mode						
		LOD (ng g^{-1})	BEC (ng g^{-1})	Measured Product Ion	Measured Mass	Gas Flow Rate (mL min^{-1})	LOD (ng g^{-1})	LOD Improvement	BEC (ng g^{-1})	BEC Improvement
Cd	114	0.000 26	0.000 23	CdO	130	0.3	0.38	0.000 70	0.051	0.004 5
Sn	118	0.000 34	0.000 50	SnO	134	0.3	0.017	0.019	0.003 7	0.13
Sb	121	0.000 72	0.000 96	SbO	137	0.5	0.006 6	0.11	0.001 3	0.72
Te	128	0.004 8	0.014	TeO	144	0.4	0.010	0.47	0.001 4	10
I	127	0.016	0.71	IO	143	0.5	0.21	0.076	0.28	2.6
Ba	138	0.000 22	0.000 19	BaO	154	0.4	0.001 6	0.14	0.000 33	0.57
La	139	0.000 055	0.000 034	LaO	155	0.3	0.000 19	0.30	0.000 069	0.49
Ce	140	0.000 083	0.000 040	CeO	156	0.3	0.000 22	0.38	0.000 094	0.42
Pr	141	0.000 015	0.000 027	PrO	157	0.3	0.000 094	0.16	0.000 050	0.54
Nd	143	0.000 47	0.000 14	NdO	159	0.3	0.001 4	0.34	0.000 33	0.42
Sm	147	0.000 13	0.000 029	SmO	163	0.4	0.000 79	0.17	0.000 24	0.12
Eu	153	0.000 032	0.000 004	EuO	169	0.4	0.000 64	0.050	0.000 087	0.050
Gd	155	0.000 77	0.001 4	GdO	171	0.3	0.002 9	0.27	0.001 5	0.95
Tb	159	0.000 13	0.000 29	TbO	175	0.3	0.000 47	0.28	0.000 30	0.98
Dy	163	0.001 5	0.001 4	DyO	179	0.3	0.002 5	0.59	0.001 5	0.90
Ho	165	0.000 17	0.000 27	HoO	181	0.3	0.000 50	0.35	0.000 24	1.1
Er	166	0.000 81	0.000 60	ErO	182	0.3	0.000 86	0.94	0.000 65	0.92
Tm	169	0.000 11	0.000 078	TmO	185	0.4	0.000 21	0.53	0.000 045	1.7
Yb	174	0.000 16	0.000 067	YbO	190	0.5	0.002 5	0.065	0.000 34	0.20
Lu	175	0.000 11	0.000 062	LuO	191	0.4	0.000 13	0.91	0.000 047	1.3
Hf	180	0.000 10	0.000 014	HfO	196	0.3	0.000 35	0.29	0.000 076	0.18
Ta	181	0.000 094	0.000 019	TaO	197	0.2	0.000 18	0.51	0.000 025	0.75
W	184	0.000 26	0.000 087	WO	200	0.2	0.000 69	0.38	0.000 094	0.92
Re	187	0.000 11	0.000 044	ReO	203	0.4	0.000 39	0.28	0.000 054	0.82
Os	189	0.000 26	0.000 051	OsO	205	0.4	0.001 3	0.20	0.000 40	0.13
Ir	193	0.000 42	0.000 46	IrO	209	0.5	0.002 5	0.17	0.001 3	0.36
Pt	195	0.001 4	0.000 85	PtO	211	0.6	0.031	0.045	0.004 3	0.20
Au	197	0.002 0	0.004 4	AuO	213	0.4	0.30	0.006 5	0.041	0.11
Pb	208	0.000 15	0.000 020	PbO	224	0.5	0.50	0.000 30	0.067	0.000 30
Bi	209	0.000 049	0.000 067	BiO	225	0.7	0.020	0.002 5	0.002 7	0.002 5
Th	232	0.000 084	0.000 026	ThO	248	0.2	0.000 16	0.52	0.000 022	1.2
U	238	0.000 063	0.000 014	UO	254	0.1	0.000 33	0.19	0.000 065	0.21

Table S6: Comparison of determined limit of detection (LOD) and background equivalence concentration (BEC) for standard mode and on-mass determinations with N_2O using a Perkin Elmer NexION 5000. LODs were calculated based on 3 times the standard deviation of the blank and expressed in ng g^{-1}. BECs were calculated by division of the blank signal with the calibration slope and expressed in ng g^{-1}. Improvement in LOD and BEC are given as a factor of how much lower these parameters are when compared to standard mode. Values are presented to 2 significant figures.

Element	Isotope	Standard Mode		N_2O DRC Mode				
		LOD (ng g^{-1})	BEC (ng g^{-1})	Gas Flow Rate (mL min^{-1})	LOD (ng g^{-1})	LOD Improvement	BEC (ng g^{-1})	BEC Improvement
Na	23	0.12	4.4	0.4	0.23	0.53	3.9	1.1
Al	27	0.001 6	0.019	0.3	0.003 9	0.41	0.017	1.1
K	39	0.88	32	0.5	0.037	23	3.6	9.1
Cr	52	0.15	10	0.3	0.000 80	180	0.001 8	5600
Mn	55	0.001 3	0.015	0.4	0.000 31	4.2	0.000 94	16
Fe	57	3.5	65	0.2	0.058	60	0.53	120
Co	59	0.000 79	0.004 8	0.3	0.000 52	1.5	0.001 2	4.0
Ni	60	0.002 1	0.007 9	0.3	0.002 3	0.92	0.006 3	1.3
Cu	63	0.000 50	0.005 2	0.3	0.000 62	0.81	0.003 4	1.5
Zn	66	0.004	0.019	0.4	0.004 2	0.96	0.023	0.85
Ga	69	0.000 63	0.001 3	0.5	0.000 92	0.69	0.000 90	1.5
Se	82	0.036	0.16	0.5	0.010	3.6	0.006 4	25
Rb	85	0.000 43	0.001 5	0.5	0.000 36	1.2	0.001 6	0.96
Mo	98	0.000 33	0.000 25	0.4	0.000 40	0.81	0.000 28	0.90
Ru	101	0.001 4	0.006 7	0.4	0.000 38	3.8	0.000 28	24
Rh	103	0.000 12	0.000 17	0.4	0.000 078	1.6	0.000 087	2.0
Pd	105	0.000 29	0.000 38	0.4	0.000 23	1.2	0.000 15	2.6
Ag	107	0.000 51	0.001 7	0.5	0.000 97	0.53	0.001 4	1.2
Cd	114	0.000 26	0.000 23	0.6	0.000 43	0.61	0.000 22	1.0
In	115	0.000 037	0.000 009	0.6	0.000 052	0.71	0.000 011	0.81
Sn	118	0.000 34	0.000 50	0.5	0.000 70	0.48	0.000 42	1.2
Sb	121	0.000 72	0.000 96	0.5	0.000 41	1.8	0.000 20	4.7
Te	128	0.004 8	0.014	0.7	0.004 9	1.0	0.013	1.1
I	127	0.016	0.71	0.6	0.025	0.63	0.93	0.77
Cs	133	0.000 098	0.000 088	0.6	0.000 22	0.46	0.000 052	1.7
Tm	169	0.000 11	0.000 078	0.5	0.000 30	0.37	0.000 11	0.72
Yb	174	0.000 16	0.000 067	0.6	0.000 18	0.90	0.000 039	1.7
Re	187	0.000 11	0.000 044	0.8	0.000 10	1.1	0.000 020	2.2
Au	197	0.002 0	0.004 4	0.6	0.000 77	2.6	0.000 39	11
Hg	202	0.006 0	0.006 1	0.8	0.004 5	1.3	0.001 1	5.7
Tl	205	0.000 46	0.000 31	0.8	0.000 83	0.56	0.000 79	0.40
Pb	208	0.000 15	0.000 020	0.8	0.000 078	1.9	0.000 27	0.076
Bi	209	0.000 049	0.000 007	0.9	0.000 092	0.53	0.000 047	0.14

Table S7: Maximum obtained formation of product ions resulting from charge transfer reactions for selected elements. Flow rates of N_2O provided are at the optimum formation using a Perkin Elmer NexION 5000.

Element	Isotope	NO^+ (m/z = 30)		O_2^+ (m/z = 32)		N_2O^+ (m/z = 44)	
		Formation (%)	Flow Rate (mL min^{-1})	Formation (%)	Flow Rate (mL min^{-1})	Formation (%)	Flow Rate (mL min^{-1})
Be	9	0.28	0.4	0.01	0.7	0.12	0.9
B	11	5.5	0.4	3.2	1.2	19	0.3
Al	27	0.01	1.1	<0.01	-	0.01	0.2
Si	29	7.3	0.3	28	0.7	23	0.4
P	31	16	0.4	1.9	0.9	12	0.3
S	34	12	0.5	6.2	1.3	5.5	0.4
Cl	35	0.88	0.1	0.76	0.5	50	0.1
Ge	74	<0.01	-	<0.01	-	0.19	0.2
As	75	0.04	0.2	<0.01	-	0.57	0.2
Se	82	0.02	0.2	<0.01	-	0.12	0.1
Br	81	0.07	0.1	<0.01	-	0.60	0.1
