# Supplementary material for: Characterisation of gas cell reactions for 70+ elements using N2O for ICP tandem mass spectrometry measurements
Source: J Anal At Spectrom. 2023 Apr 28;38(5):1135–45. doi: 10.1039/d3ja00025g (PMC10171245; doi:10.1039/d3ja00025g)
Supplement: JA-038-D3JA00025G-s002 [file JA-038-D3JA00025G-s002.pdf]

Electronic Supplementary Material (ESI) for JAAS.
This journal is © The Royal Society of Chemistry 2023

Supplementary Information B to: Characterisation of gas cell reactions for 70+ elements using N_2O for ICP tandem mass spectrometry measurements

Shaun T. Lancaster[a]*, Thomas Prohaska[ab] and Johanna Irrgeher[ab]

[a]Department of General, Analytical and Physical Chemistry, Chair of General and Analytical Chemistry, Montanuniversität Leoben, Leoben, Austria.

[b]Department of Physics and Astronomy, University of Calgary, Calgary, Canada.

H																	He
Li	Be											B	C	N	O	F	Ne
Na	Mg											Al	Si	P	S	Cl	Ar
K	Ca	Sc	Ti	V	Cr	Mn	Fe	Co	Ni	Cu	Zn	Ga	Ge	As	Se	Br	Kr
Rb	Sr	Y	Zr	Nb	Mo	Tc	Ru	Rh	Pd	Ag	Cd	In	Sn	Sb	Te	I	Xe
Cs	Ba	Ln-Lu	Hf	Ta	W	Re	Os	Ir	Pt	Au	Hg	Tl	Pb	Bi	Po	At	Rn
Fr	Ra	Ac-Lr	Rf	Db	Sg	Bh	Hs	Mt	Ds	Rg	Cn	Nh	Fl	Mc	Lv	Ts	Og
		La	Ce	Pr	Nd	Pm	Sm	Eu	Gd	Tb	Dy	Ho	Er	Tm	Yb	Lu	
		Ac	Th	Pa	U	Np	Pu	Am	Cm	Bk	Cf	Es	Fm	Md	No	Lr	

Lithium

Supplementary Information B to: Characterisation of gas cell reactions for 70+ elements using N_2O for ICP tandem mass spectrometry measurements
Shaun T. Lancaster[a]*, Thomas Prohaska[ab] and Johanna Irrgeher[ab]
[a]Department of General, Analytical and Analytical Chemistry, Chair of General and Analytical Chemistry, Montanuniversität Leoben, Leoben, Austria.
[b]Department of Physics and Astronomy, University of Calgary, Calgary, Canada.

Mass-Shift with N_2O and O_2

Major Product Ions of $^{7}Li^{+}$ with N_2O Reaction Gas

[NO ATOM TRANSFER REACTION OBSERVED]

Major Product Ions of $^{7}Li^{+}$ with O_2 Reaction Gas

[NO ATOM TRANSFER REACTION OBSERVED]

On-Mass with N_2O and O_2

On-Mass Profile of $^{7}Li^{+}$ with N_2O and O_2

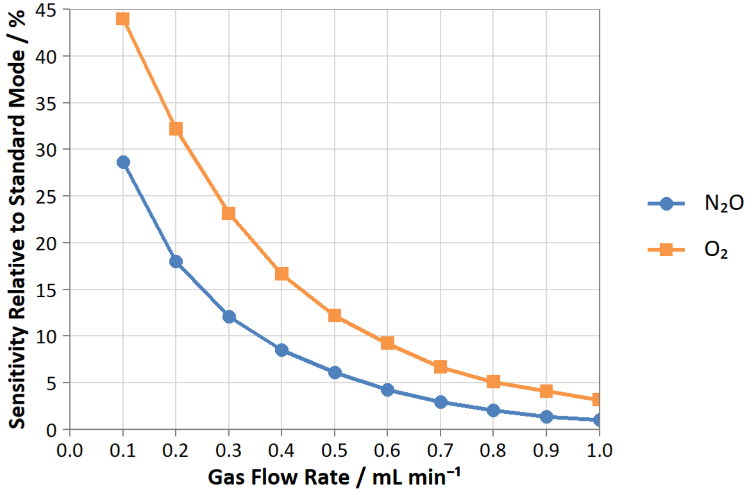

On-Mass Removal (as Interference) with N_2O

Variation of $^{7}Li^{16}O^{+}$ Interference with N_2O Flow Rate (On-Mass: Q1 = 23, Q3 = 23)

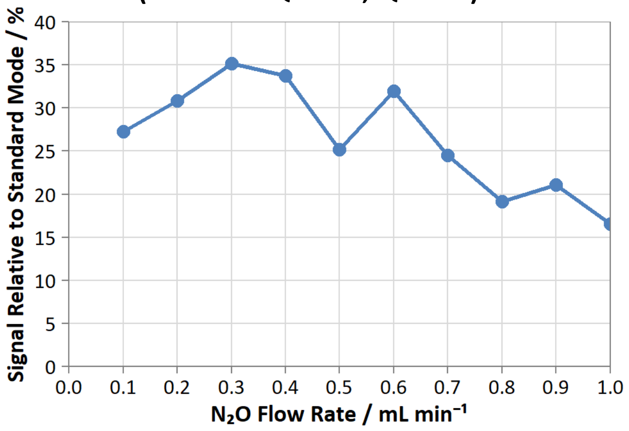

Variation of $^{7}Li^{2+}$ Interference with N_2O Flow Rate (On-Mass: Q1 = 3.5, Q3 = 3.5)

[INSUFFICIENT DOUBLY-CHARGED ION FORMATION OBSERVED]

Asymmetric Charge Transfer (with N_2O)

[NO CHARGE TRANSFER REACTION OBSERVED]

Beryllium

Supplementary Information B to: Characterisation of gas cell reactions for 70+ elements using N_2O for ICP tandem mass spectrometry measurements
Shaun T. Lancaster[a]*, Thomas Prohaska[ab] and Johanna Irrgeher[ab]
[a]Department of General, Analytical and Physical Chemistry, Chair of General and Analytical Chemistry, Montanuniversität Leoben, Leoben, Austria.
[b]Department of Physics and Astronomy, University of Calgary, Calgary, Canada.

Mass-Shift with N_2O and O_2

Major Product Ions of $^9Be^+$ with N_2O Reaction Gas

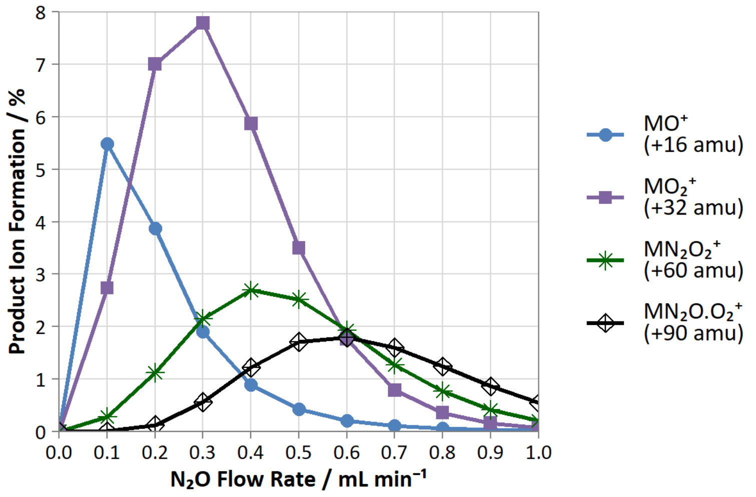

Major Product Ions of $^9Be^+$ with O_2 Reaction Gas

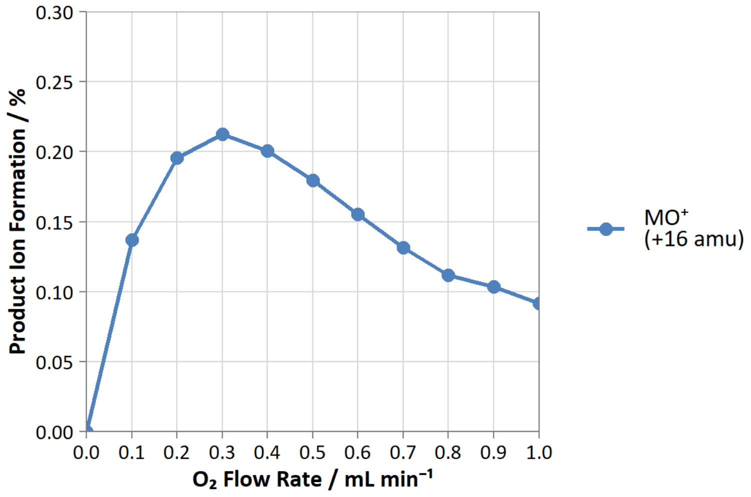

On-Mass with N_2O and O_2

On-Mass Profile of $^9Be^+$ with N_2O and O_2

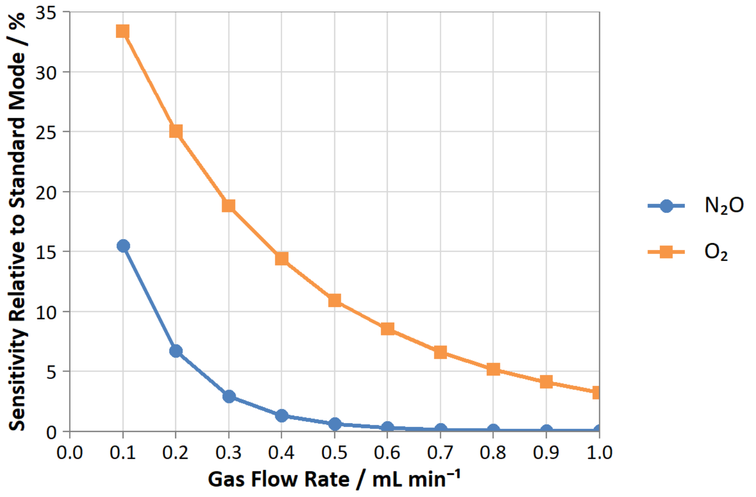

On-Mass Removal (as Interference) with N_2O

Variation of $^9Be^{16}O^+$ Interference with N_2O Flow Rate (On-Mass: Q1 = 25, Q3 = 25)

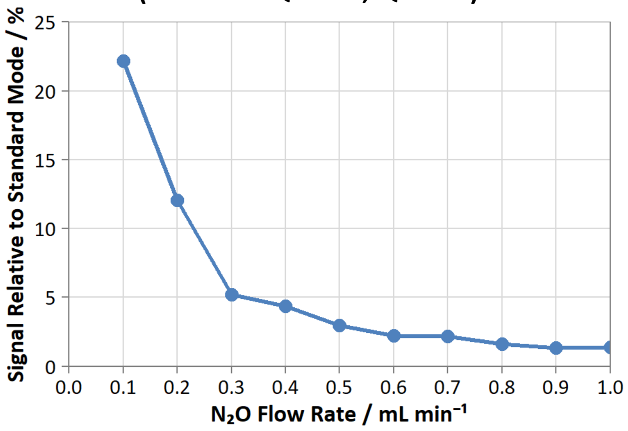

Variation of $^9Be^{2+}$ Interference with N_2O Flow Rate (On-Mass: Q1 = 4.5, Q3 = 4.5)

[INSUFFICIENT DOUBLY-CHARGED ION FORMATION OBSERVED]

Asymmetric Charge Transfer (with N_2O)

Profile of Charge Transfer Reaction Between $^9Be_{(m)}^+$ and N_2O

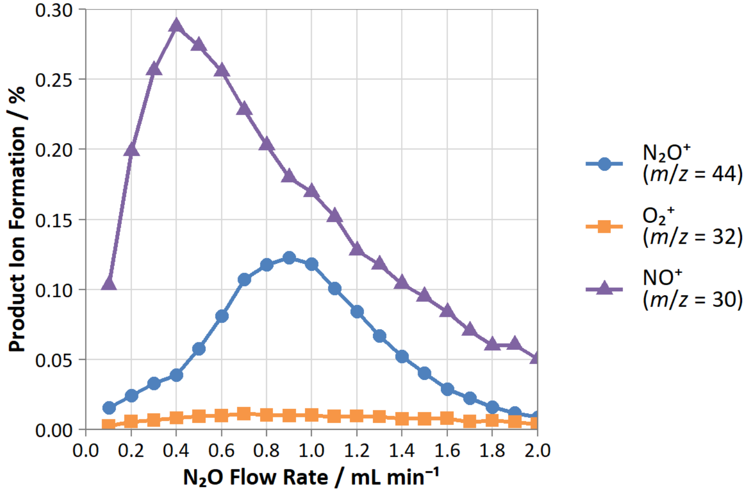

Boron

Supplementary Information B to: Characterisation of gas cell reactions for 70+ elements using N_2O for ICP tandem mass spectrometry measurements
Shaun T. Lancaster[a]*, Thomas Prohaska[ab] and Johanna Irrgeher[ab]
[a]Department of General, Analytical and Physical Chemistry, Chair of General and Analytical Chemistry, Montanuniversität Leoben, Leoben, Austria.
[b]Department of Physics and Astronomy, University of Calgary, Calgary, Canada.

Mass-Shift with N_2O and O_2

Major Product Ions of $^{11}B^+$ with N_2O Reaction Gas

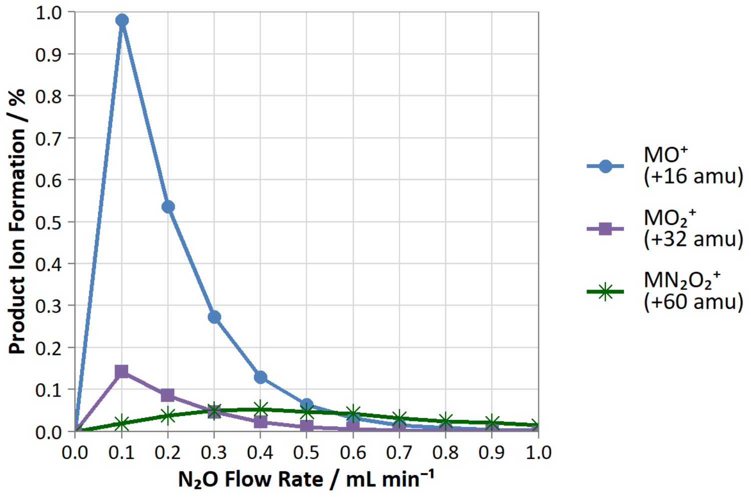

Major Product Ions of $^{11}B^+$ with O_2 Reaction Gas

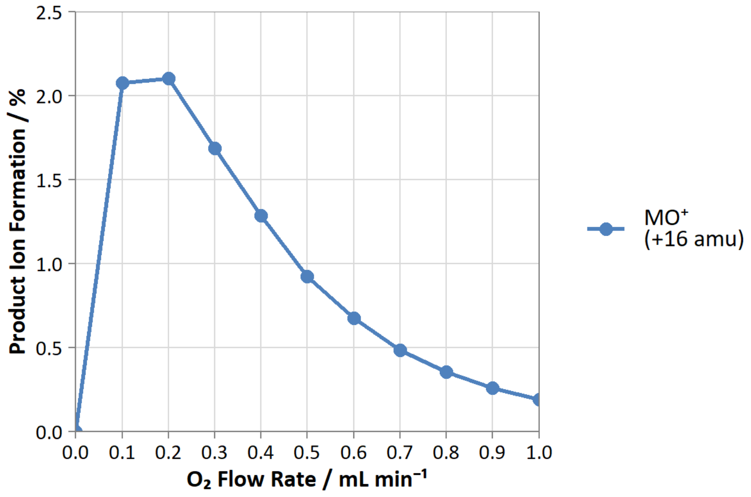

On-Mass with N_2O and O_2

On-Mass Profile of $^{11}B^+$ with N_2O and O_2

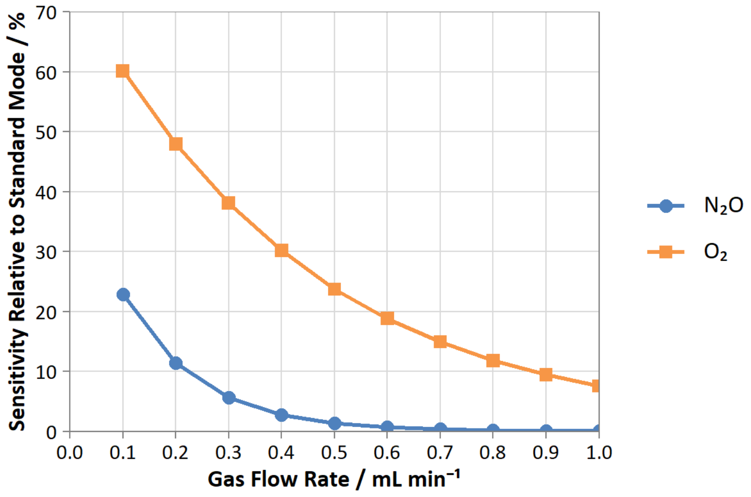

On-Mass Removal (as Interference) with N_2O

Variation of $^{11}B^{16}O^+$ Interference with N_2O Flow Rate (On-Mass: Q1 = 27, Q3 = 27)

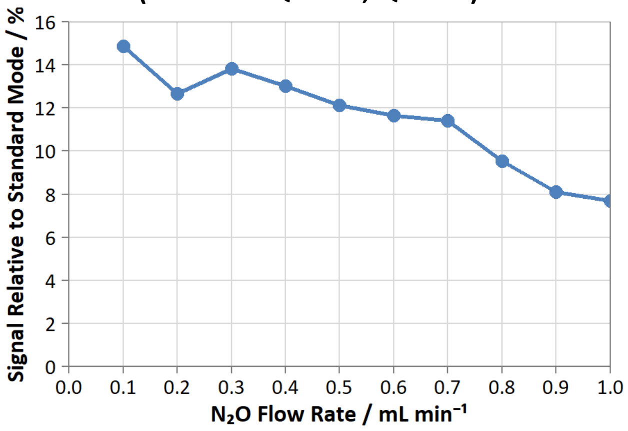

Variation of $^{11}B^{2+}$ Interference with N_2O Flow Rate (On-Mass: Q1 = 5.5, Q3 = 5.5)

[INSUFFICIENT DOUBLY-CHARGED ION FORMATION OBSERVED]

Asymmetric Charge Transfer (with N_2O)

Profile of Charge Transfer Reaction Between $^{11}B_{(m)}^+$ and N_2O

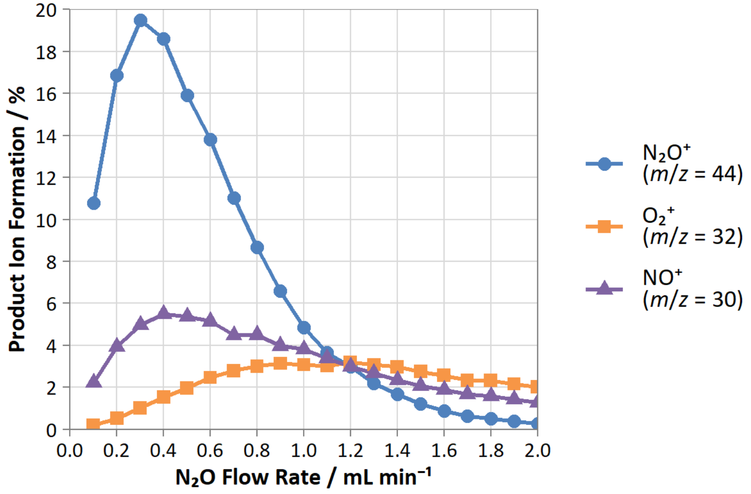

Sodium

Shaun T. Lancaster[a]*, Thomas Prohaska[ab] and Johanna Irrgeher[ab]
[a]Department of General, Analytical and Physical Chemistry, Chair of General and Analytical Chemistry, Montanuniversität Leoben, Leoben, Austria.
[b]Department of Physics and Astronomy, University of Calgary, Calgary, Canada.

Mass-Shift with N_2O and O_2

Major Product Ions of $^{23}Na^+$ with N_2O Reaction Gas

[NO ATOM TRANSFER REACTION OBSERVED]

Major Product Ions of $^{23}Na^+$ with O_2 Reaction Gas

[NO ATOM TRANSFER REACTION OBSERVED]

On-Mass with N_2O and O_2

On-Mass Profile of $^{23}Na^+$ with N_2O and O_2

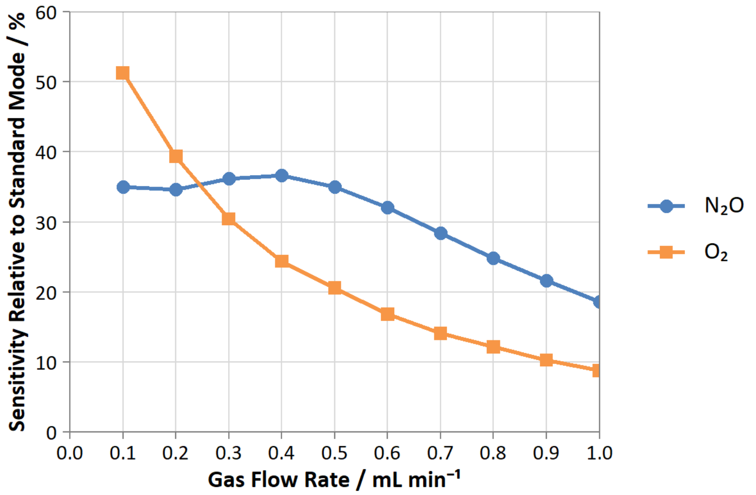

On-Mass Removal (as Interference) with N_2O

Variation of $^{23}Na^{16}O^+$ Interference with N_2O Flow Rate (On-Mass: Q1 = 39, Q3 = 39)

[INSUFFICIENT OXIDE FORMATION OBSERVED]

Variation of $^{23}Na^{2+}$ Interference with N_2O Flow Rate (On-Mass: Q1 = 11.5, Q3 = 11.5)

[INSUFFICIENT DOUBLY-CHARGED ION FORMATION OBSERVED]

Asymmetric Charge Transfer (with N_2O)

[NO CHARGE TRANSFER REACTION OBSERVED]

Magnesium

Supplementary Information B to: Characterisation of gas cell reactions for 70+ elements using N_2O for ICP tandem mass spectrometry measurements
Shaun T. Lancaster[a]*, Thomas Prohaska[ab] and Johanna Irrgeher[ab]
[a]Department of General, Analytical and Physical Chemistry, Chair of General and Analytical Chemistry, Montanuniversität Leoben, Leoben, Austria.
[b]Department of Physics and Astronomy, University of Calgary, Calgary, Canada.

Mass-Shift with N_2O and O_2

Major Product Ions of $^{24}Mg^+$ with N_2O Reaction Gas

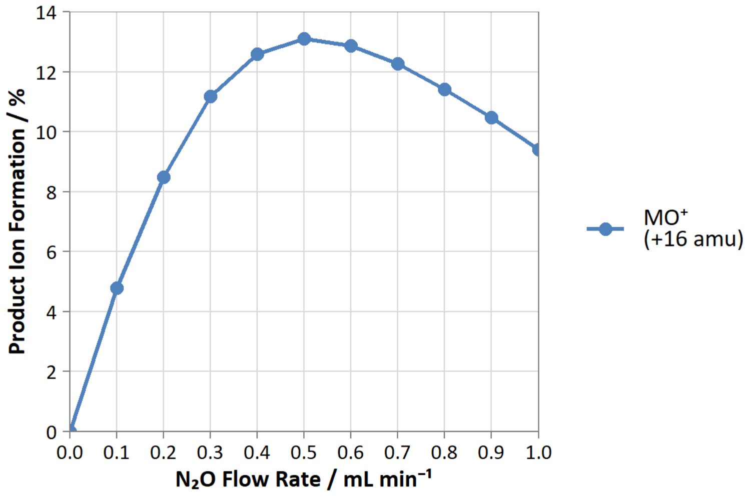

Major Product Ions of $^{24}Mg^+$ with O_2 Reaction Gas

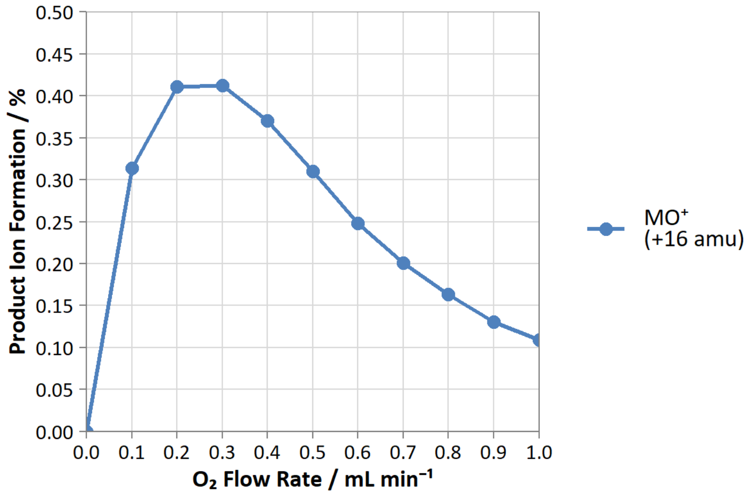

On-Mass with N_2O and O_2

On-Mass Profile of $^{24}Mg^+$ with N_2O and O_2

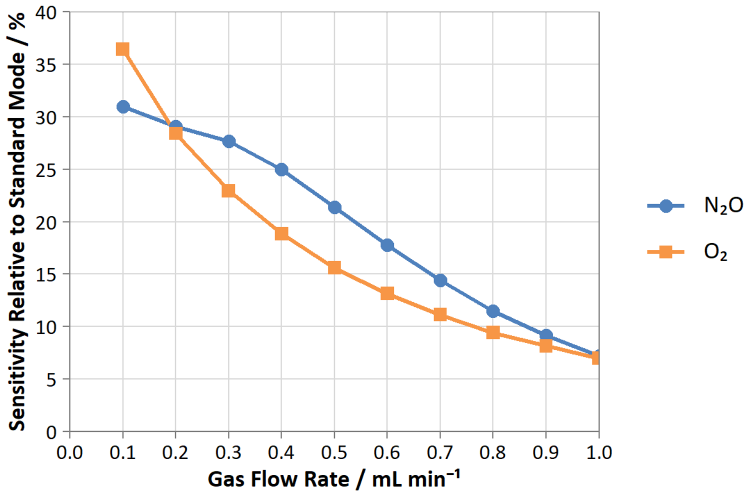

On-Mass Removal (as Interference) with N_2O

Variation of $^{24}Mg^{16}O^+$ Interference with N_2O Flow Rate (On-Mass: Q1 = 40, Q3 = 40)

[NOT MEASURED]

Variation of $^{24}Mg^{2+}$ Interference with N_2O Flow Rate (On-Mass: Q1 = 12, Q3 = 12)

[INSUFFICIENT DOUBLY-CHARGED ION FORMATION OBSERVED]

Asymmetric Charge Transfer (with N_2O)

[NO CHARGE TRANSFER REACTION OBSERVED]

Aluminium

Supplementary Information B to: Characterisation of gas cell reactions for 70+ elements using N_2O for ICP tandem mass spectrometry measurements
Shaun T. Lancaster[a]*, Thomas Prohaska[ab] and Johanna Irrgeher[ab]
[a]Department of General, Analytical and Physical Chemistry, Chair of General and Analytical Chemistry, Montanuniversität Leoben, Leoben, Austria.
[b]Department of Physics and Astronomy, University of Calgary, Calgary, Canada.

Mass-Shift with N_2O and O_2

Major Product Ions of $^{27}Al^+$ with N_2O Reaction Gas

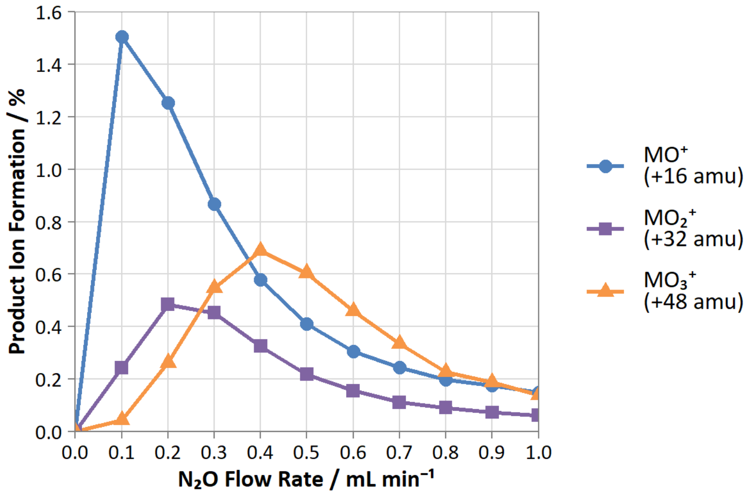

Major Product Ions of $^{27}Al^+$ with O_2 Reaction Gas

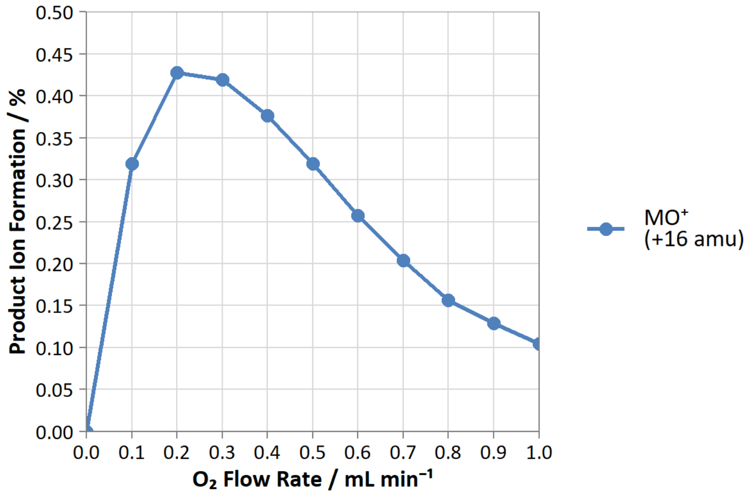

On-Mass with N_2O and O_2

On-Mass Profile of $^{27}Al^+$ with N_2O and O_2

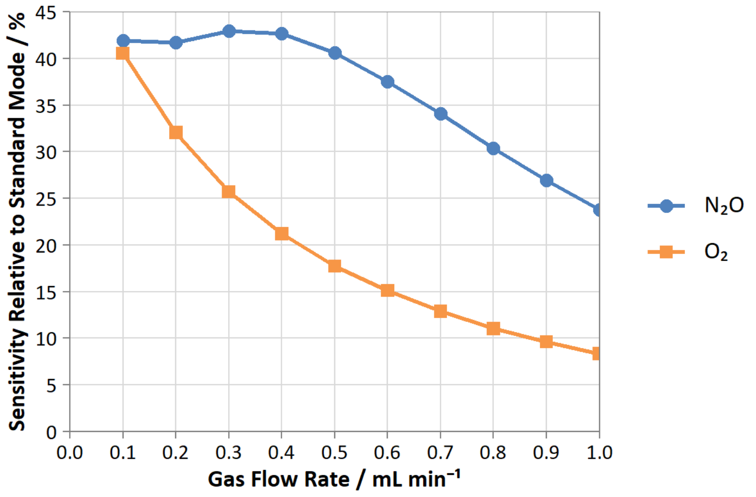

On-Mass Removal (as Interference) with N_2O

Variation of $^{27}Al^{16}O^+$ Interference with N_2O Flow Rate (On-Mass: Q1 = 43, Q3 = 43)

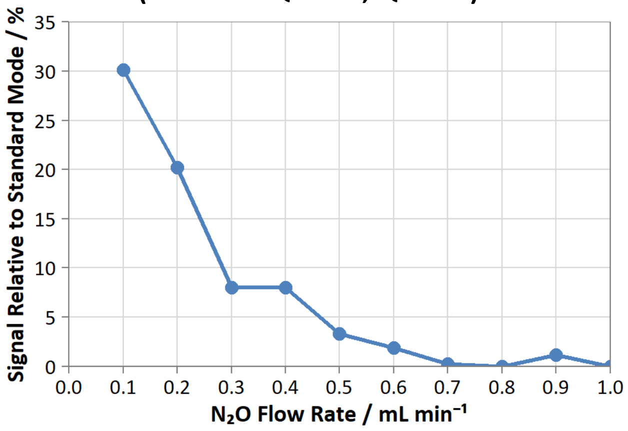

Variation of $^{27}Al^{2+}$ Interference with N_2O Flow Rate (On-Mass: Q1 = 13.5, Q3 = 13.5)

[INSUFFICIENT DOUBLY-CHARGED ION FORMATION OBSERVED]

Asymmetric Charge Transfer (with N_2O)

Profile of Charge Transfer Reaction Between $^{27}Al_{(m)}^+$ and N_2O

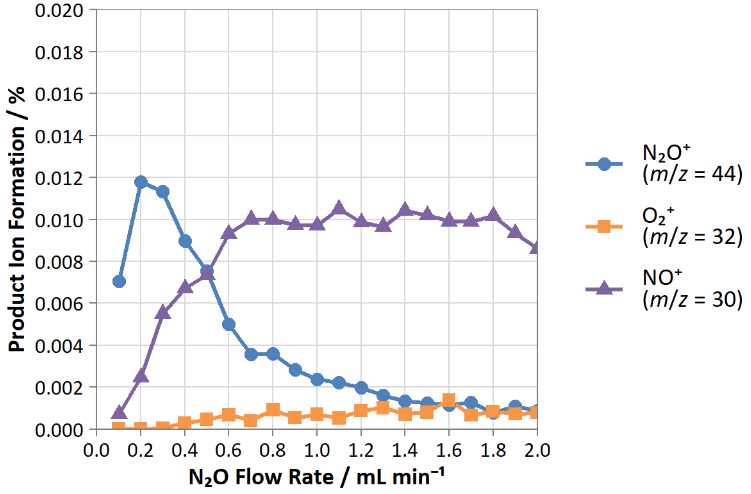

Silicon

Supplementary Information B to: Characterisation of gas cell reactions for 70+ elements using N_2O for ICP tandem mass spectrometry measurements
Shaun T. Lancaster[a]*, Thomas Prohaska[ab] and Johanna Irrgeher[ab]
[a]Department of General, Analytical and Physical Chemistry, Chair of General and Analytical Chemistry, Montanuniversität Leoben, Leoben, Austria.
[b]Department of Physics and Astronomy, University of Calgary, Calgary, Canada.

Mass-Shift with N_2O and O_2

Major Product Ions of $^{29}Si^+$ with N_2O Reaction Gas

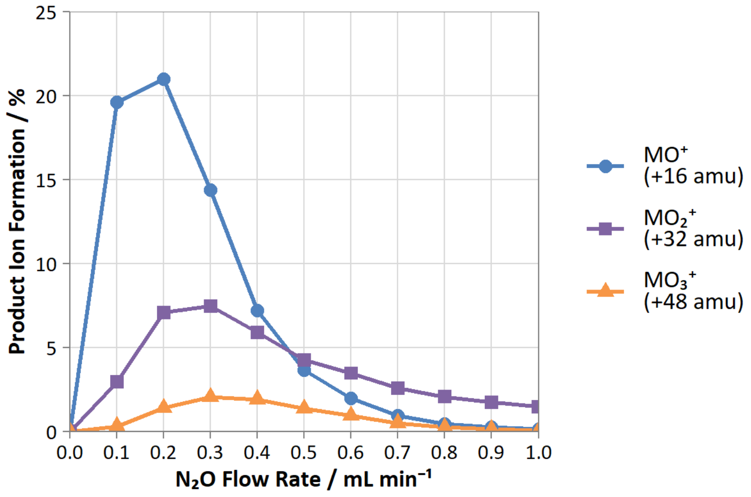

Major Product Ions of $^{29}Si^+$ with O_2 Reaction Gas

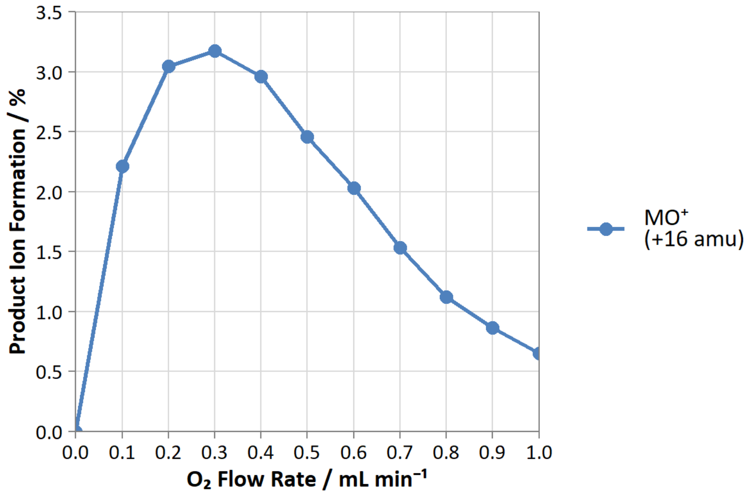

On-Mass with N_2O and O_2

On-Mass Profile of $^{29}Si^+$ with N_2O and O_2

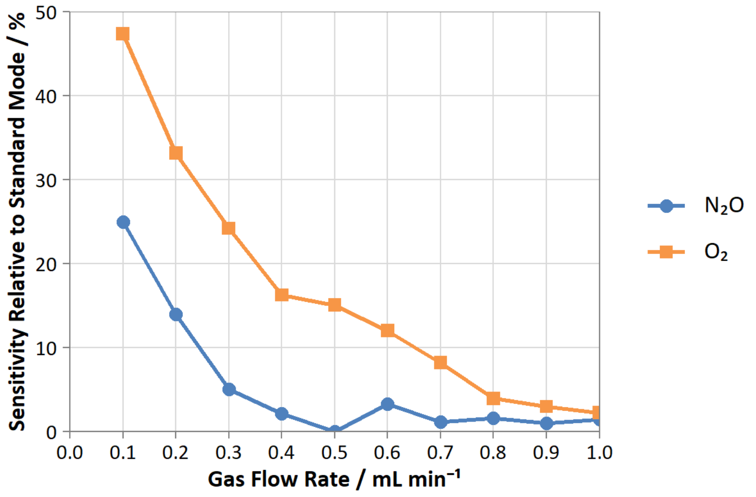

On-Mass Removal (as Interference) with N_2O

Variation of $^{29}Si^{16}O^+$ Interference with N_2O Flow Rate (On-Mass: Q1 = 45, Q3 = 45)

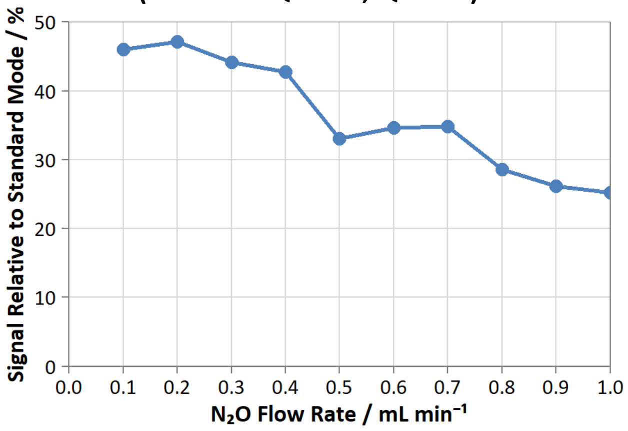

Variation of $^{29}Si^{2+}$ Interference with N_2O Flow Rate (On-Mass: Q1 = 14.5, Q3 = 14.5)

[INSUFFICIENT DOUBLY-CHARGED ION FORMATION OBSERVED]

Asymmetric Charge Transfer (with N_2O)

Profile of Charge Transfer Reaction Between $^{29}Si_{(m)}^+$ and N_2O

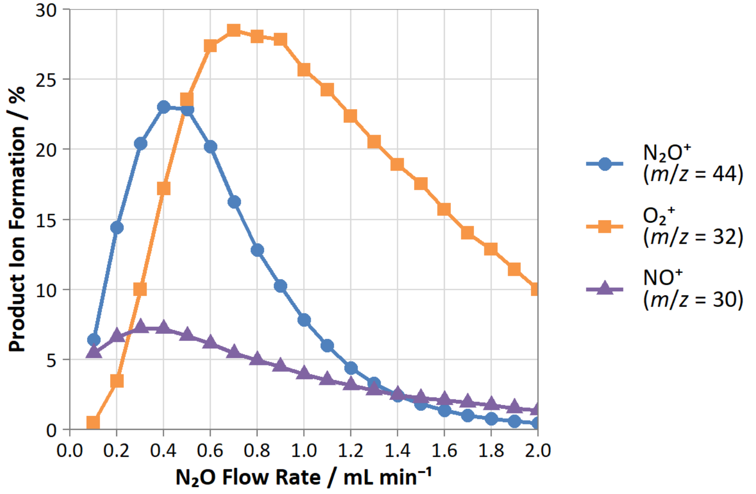

Phosphorus

Supplementary Information B to: Characterisation of gas cell reactions for 70+ elements using N_2O for ICP tandem mass spectrometry measurements
Shaun T. Lancaster[a]*, Thomas Prohaska[ab] and Johanna Irrgeher[ab]
[a]Department of General, Analytical and Physical Chemistry, Chair of General and Analytical Chemistry, Montanuniversität Leoben, Leoben, Austria.
[b]Department of Physics and Astronomy, University of Calgary, Calgary, Canada.

Mass-Shift with N_2O and O_2

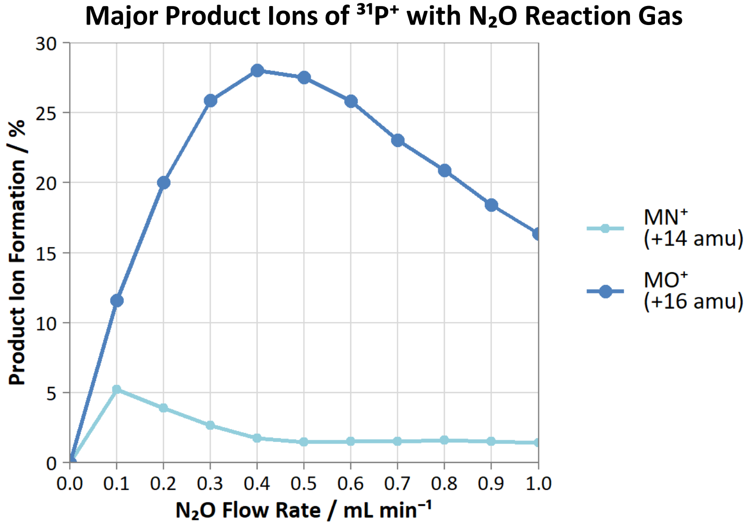

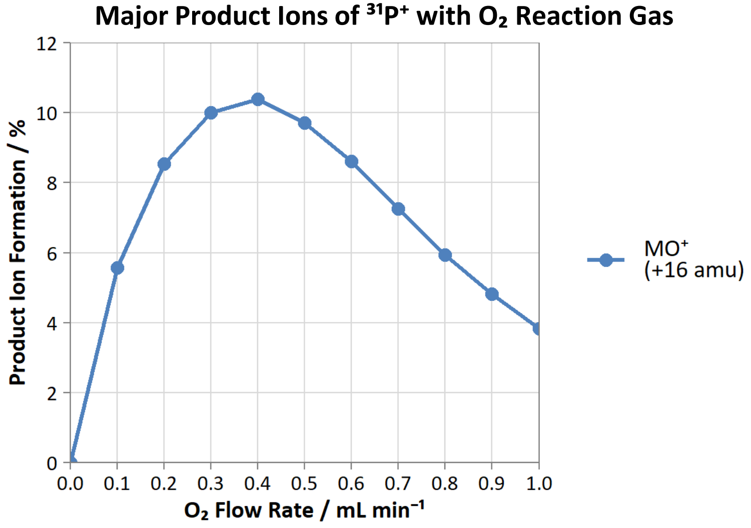

On-Mass with N_2O and O_2

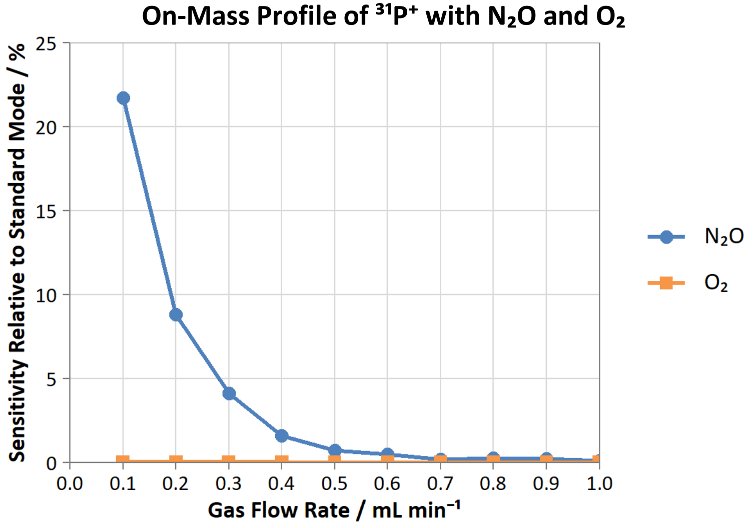

On-Mass Removal (as Interference) with N_2O

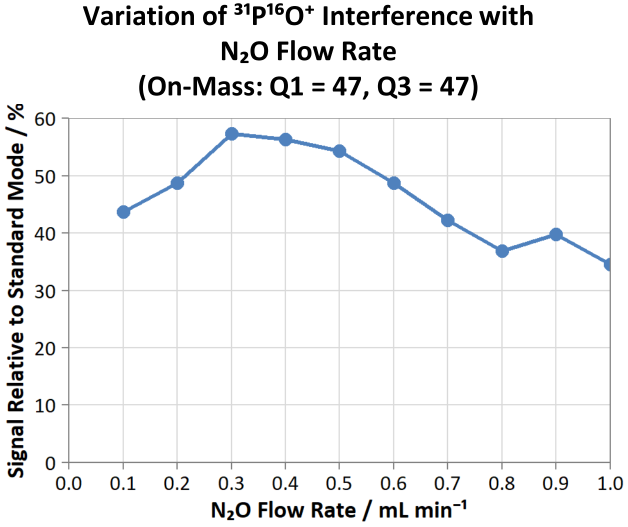

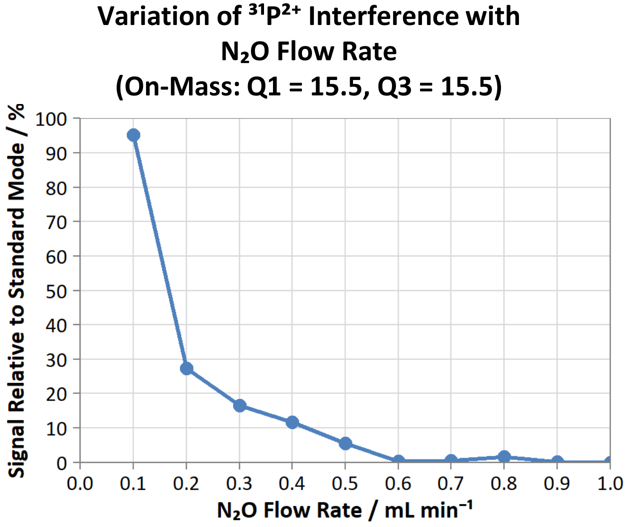

Asymmetric Charge Transfer (with N_2O)

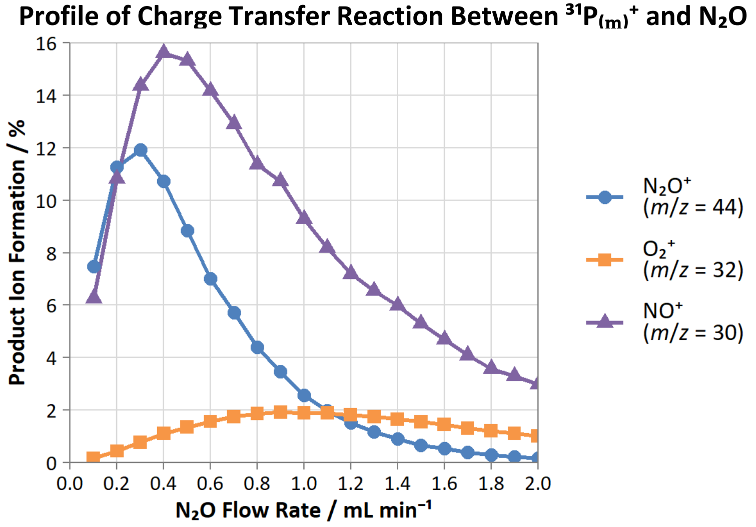

Sulphur

Supplementary Information B to: Characterisation of gas cell reactions for 70+ elements using N_2O for ICP tandem mass spectrometry measurements
Shaun T. Lancaster[a]*, Thomas Prohaska[ab] and Johanna Irrgeher[ab]
[a]Department of General, Analytical and Physical Chemistry, Chair of General and Analytical Chemistry, Montanuniversität Leoben, Leoben, Austria.
[b]Department of Physics and Astronomy, University of Calgary, Calgary, Canada.

Mass-Shift with N_2O and O_2

Major Product Ions of $^{34}S^+$ with N_2O Reaction Gas

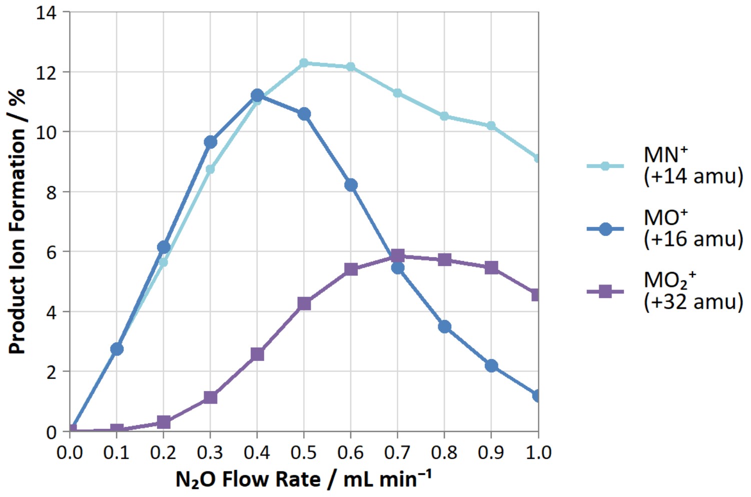

Major Product Ions of $^{34}S^+$ with O_2 Reaction Gas

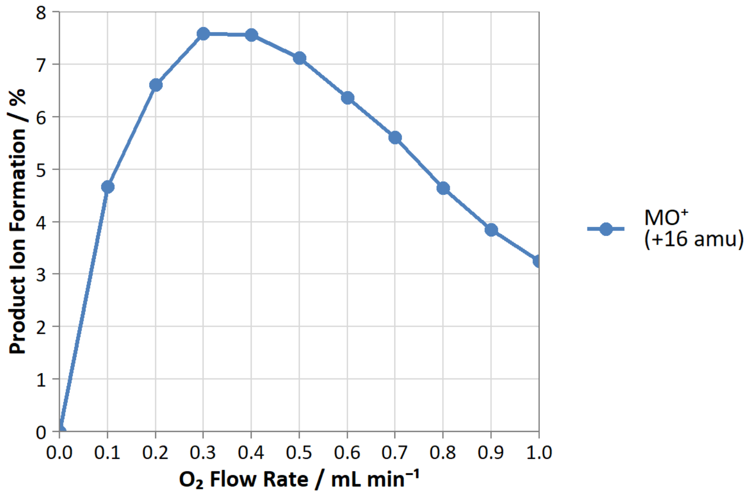

On-Mass with N_2O and O_2

On-Mass Profile of $^{34}S^+$ with N_2O and O_2

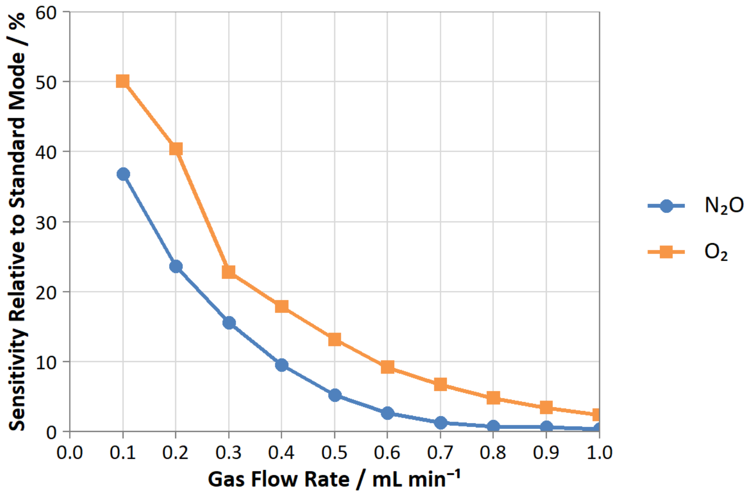

On-Mass Removal (as Interference) with N_2O

Variation of $^{34}S^{16}O^+$ Interference with N_2O Flow Rate (On-Mass: Q1 = 50, Q3 = 50)

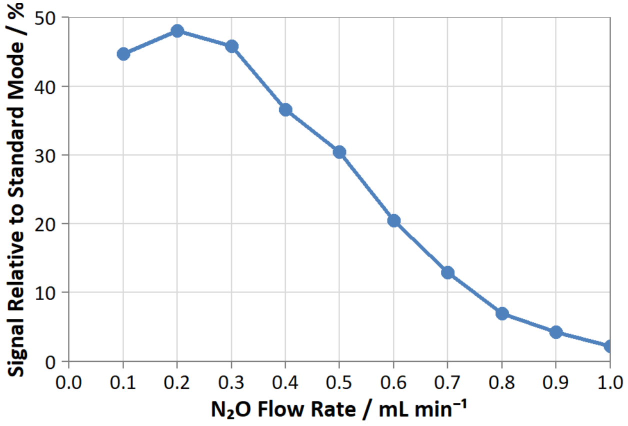

Variation of $^{34}S^{2+}$ Interference with N_2O Flow Rate (On-Mass: Q1 = 17, Q3 = 17)

[NOT MEASURED]

Asymmetric Charge Transfer (with N_2O)

Profile of Charge Transfer Reaction Between $^{34}S_{(m)}^+$ and N_2O

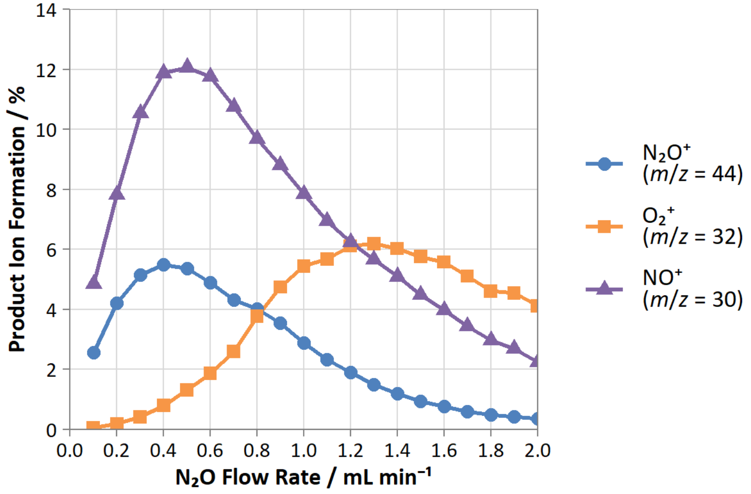

Chlorine

Shaun T. Lancaster[a]*, Thomas Prohaska[ab] and Johanna Irrgeher[ab]
[a]Department of General, Analytical and Physical Chemistry, Chair of General and Analytical Chemistry, Montanuniversität Leoben, Leoben, Austria.
[b]Department of Physics and Astronomy, University of Calgary, Calgary, Canada.

Mass-Shift with N_2O and O_2

Major Product Ions of $^{35}Cl^+$ with N_2O Reaction Gas

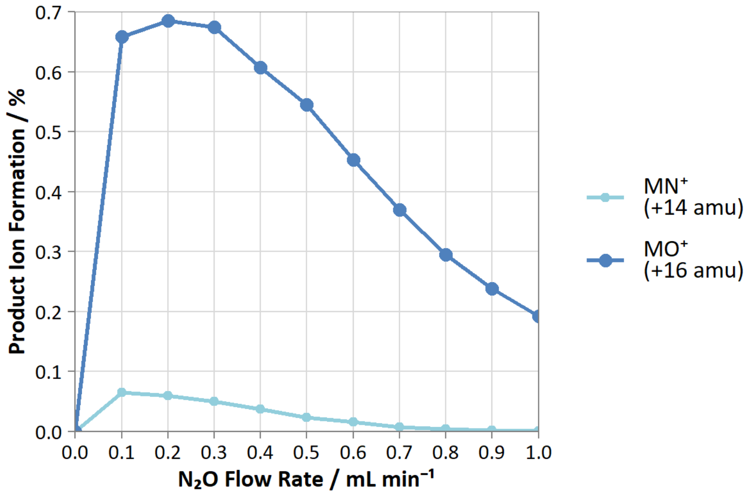

Major Product Ions of $^{35}Cl^+$ with O_2 Reaction Gas

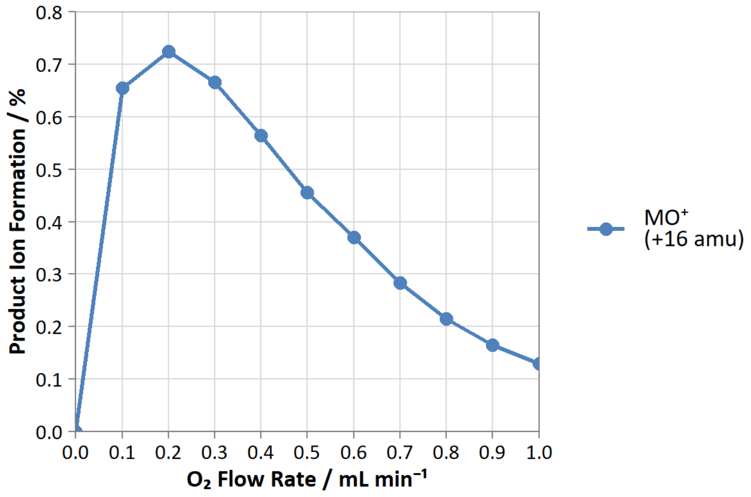

On-Mass with N_2O and O_2

On-Mass Profile of $^{35}Cl^+$ with N_2O and O_2

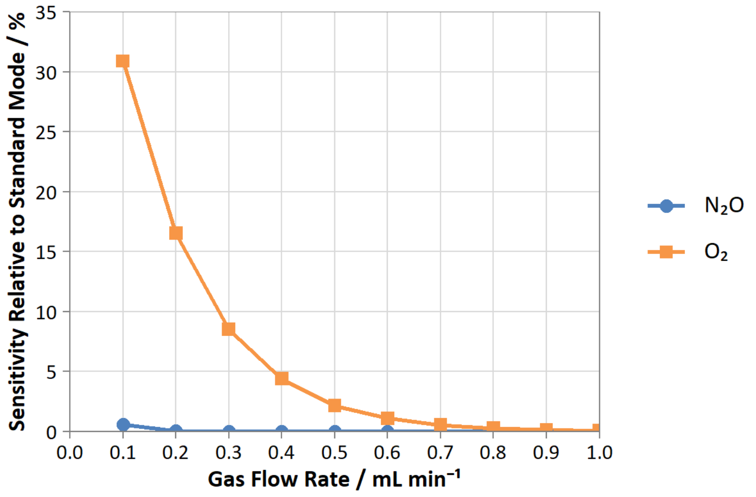

On-Mass Removal (as Interference) with N_2O

Variation of $^{35}Cl^{16}O^+$ Interference with N_2O Flow Rate (On-Mass: Q1 = 51, Q3 = 51)

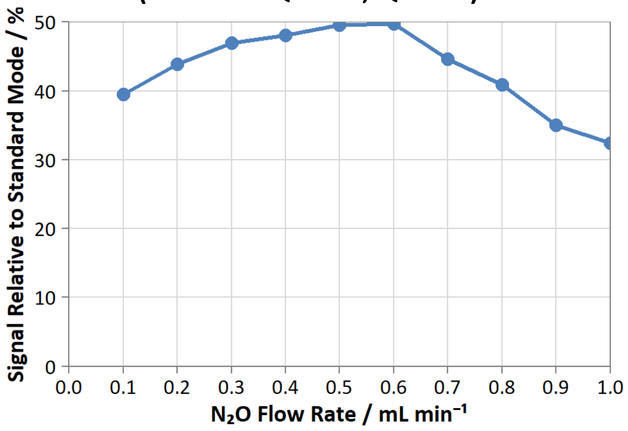

Variation of $^{35}Cl^{2+}$ Interference with N_2O Flow Rate (On-Mass: Q1 = 17.5, Q3 = 17.5)

[INSUFFICIENT DOUBLY-CHARGED ION FORMATION OBSERVED]

Asymmetric Charge Transfer (with N_2O)

Profile of Charge Transfer Reaction Between $^{35}Cl^+$ and N_2O

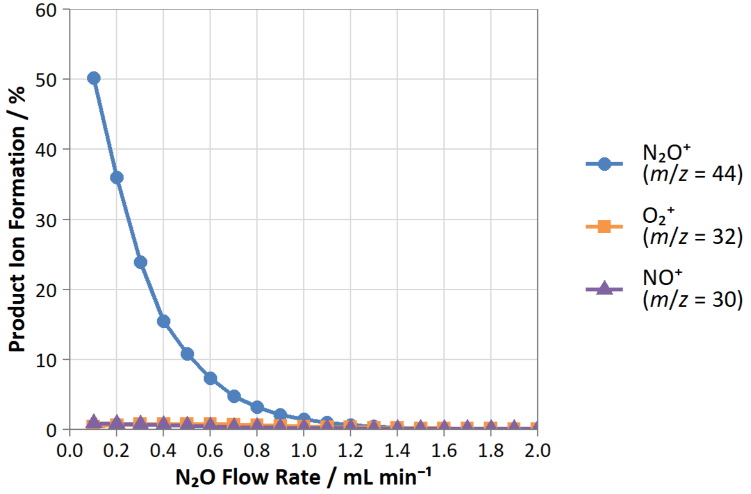

Potassium

Supplementary Information B to: Characterisation of gas cell reactions for 70+ elements using N_2O for ICP tandem mass spectrometry measurements
Shaun T. Lancaster[a]*, Thomas Prohaska[ab] and Johanna Irrgeher[ab]
[a]Department of General, Analytical and Physical Chemistry, Chair of General and Analytical Chemistry, Montanuniversität Leoben, Leoben, Austria.
[b]Department of Physics and Astronomy, University of Calgary, Calgary, Canada.

Mass-Shift with N_2O and O_2

Major Product Ions of $^{39}K^+$ with N_2O Reaction Gas

[NO ATOM TRANSFER REACTION OBSERVED]

Major Product Ions of $^{39}K^+$ with O_2 Reaction Gas

[NO ATOM TRANSFER REACTION OBSERVED]

On-Mass with N_2O and O_2

On-Mass Profile of $^{39}K^+$ with N_2O and O_2

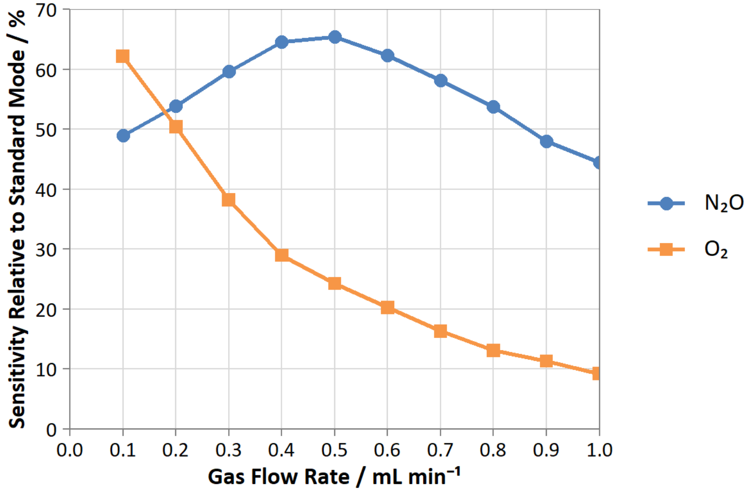

On-Mass Removal (as Interference) with N_2O

Variation of $^{39}K^{16}O^+$ Interference with N_2O Flow Rate (On-Mass: Q1 = 55, Q3 = 55)

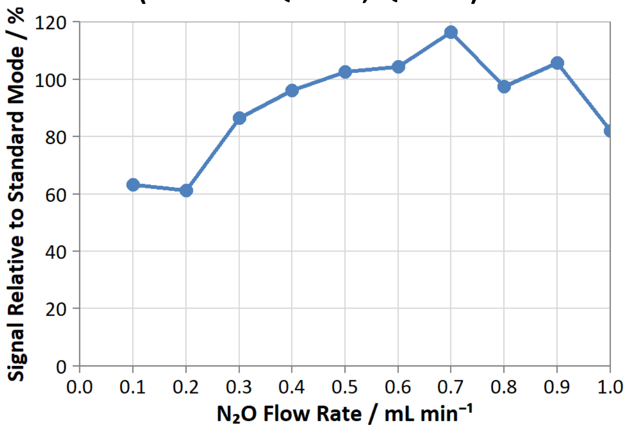

Variation of $^{39}K^{2+}$ Interference with N_2O Flow Rate (On-Mass: Q1 = 19.5, Q3 = 19.5)

[INSUFFICIENT DOUBLY-CHARGED ION FORMATION OBSERVED]

Asymmetric Charge Transfer (with N_2O)

[NO CHARGE TRANSFER REACTION OBSERVED]

Calcium

Supplementary Information B to: Characterisation of gas cell reactions for 70+ elements using N_2O for ICP tandem mass spectrometry measurements
Shaun T. Lancaster[a]*, Thomas Prohaska[ab] and Johanna Irrgeher[ab]
[a]Department of General, Analytical and Physical Chemistry, Chair of General and Analytical Chemistry, Montanuniversität Leoben, Leoben, Austria.
[b]Department of Physics and Astronomy, University of Calgary, Calgary, Canada.

Mass-Shift with N_2O and O_2

Major Product Ions of $^{44}Ca^+$ with N_2O Reaction Gas

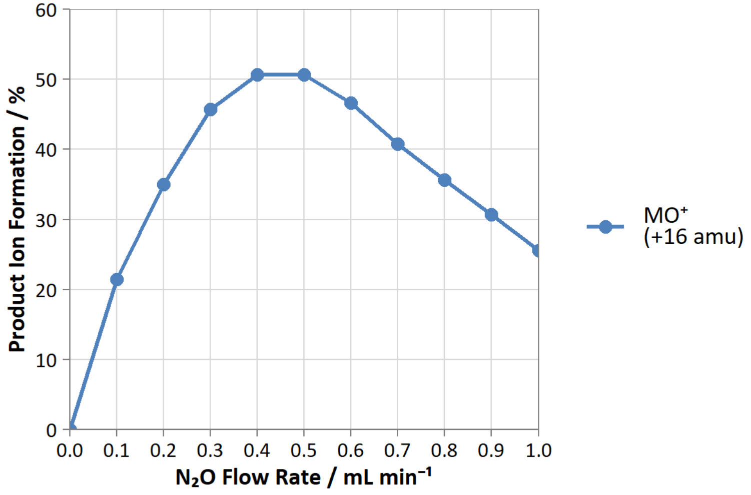

Major Product Ions of $^{44}Ca^+$ with O_2 Reaction Gas

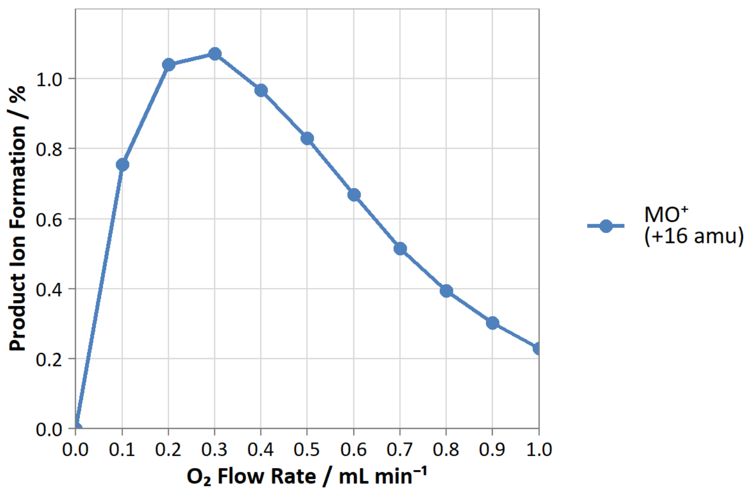

On-Mass with N_2O and O_2

On-Mass Profile of $^{44}Ca^+$ with N_2O and O_2

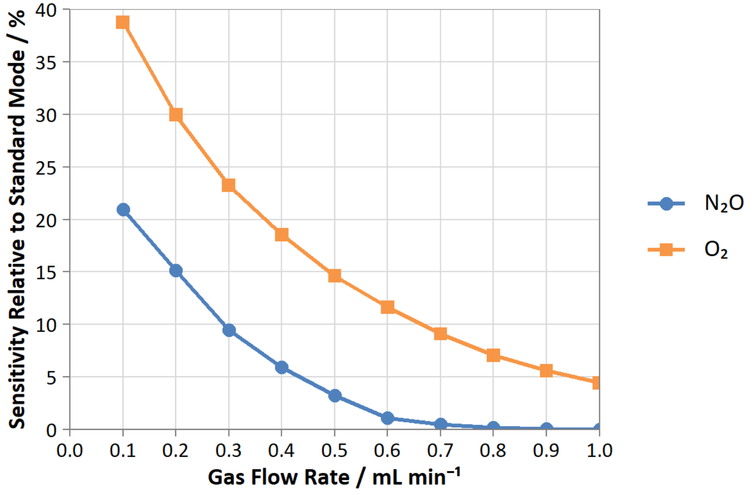

On-Mass Removal (as Interference) with N_2O

Variation of $^{44}Ca^{16}O^+$ Interference with N_2O Flow Rate (On-Mass: Q1 = 60, Q3 = 60)

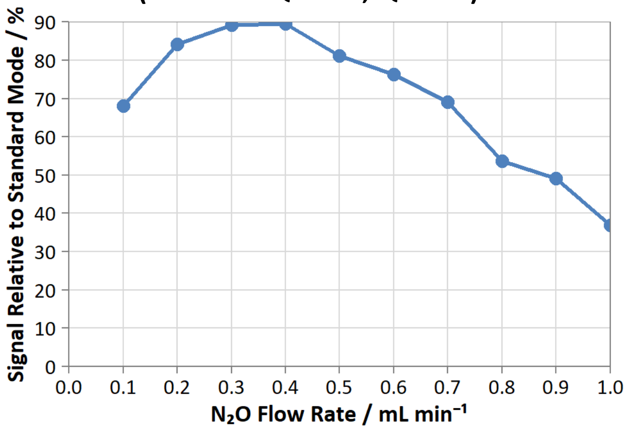

Variation of $^{44}Ca^{2+}$ Interference with N_2O Flow Rate (On-Mass: Q1 = 22, Q3 = 22)

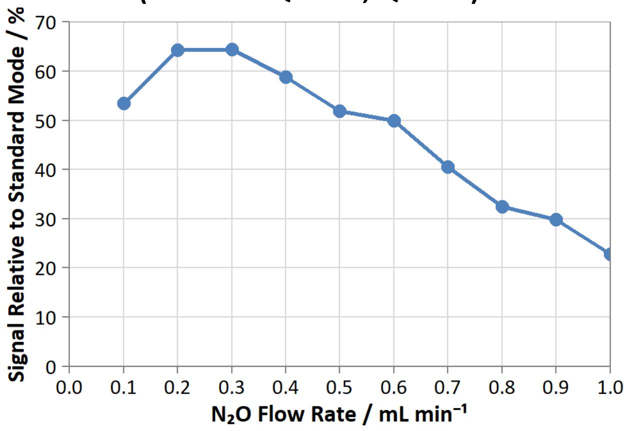

Asymmetric Charge Transfer (with N_2O)

[NO CHARGE TRANSFER REACTION OBSERVED]

Scandium

Supplementary Information B to: Characterisation of gas cell reactions for 70+ elements using N_2O for ICP tandem mass spectrometry measurements
Shaun T. Lancaster[a]*, Thomas Prohaska[ab] and Johanna Irrgeher[ab]
[a]Department of General, Analytical and Physical Chemistry, Chair of General and Analytical Chemistry, Montanuniversität Leoben, Leoben, Austria.
[b]Department of Physics and Astronomy, University of Calgary, Calgary, Canada.

Mass-Shift with N_2O and O_2

Major Product Ions of $^{45}Sc^+$ with N_2O Reaction Gas

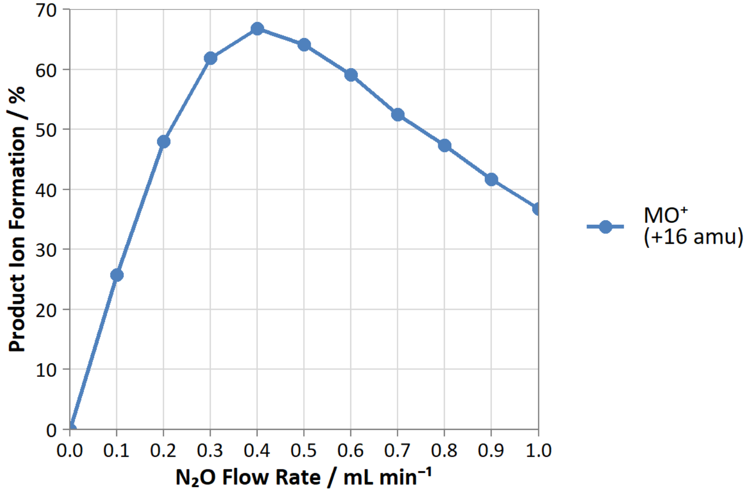

Major Product Ions of $^{45}Sc^+$ with O_2 Reaction Gas

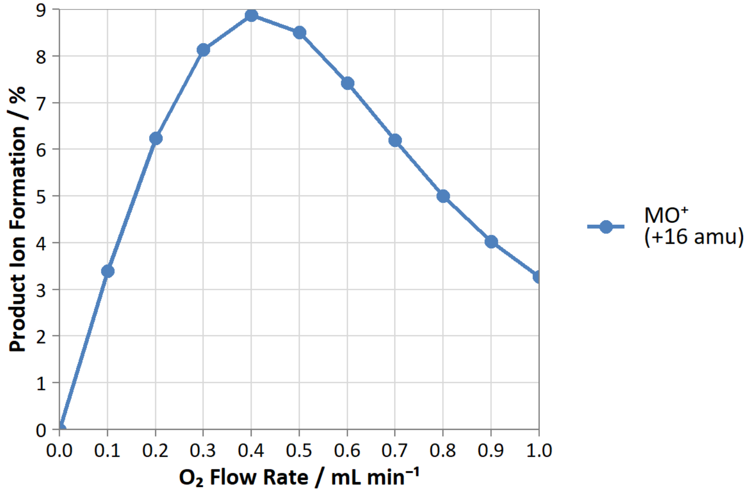

On-Mass with N_2O and O_2

On-Mass Profile of $^{45}Sc^+$ with N_2O and O_2

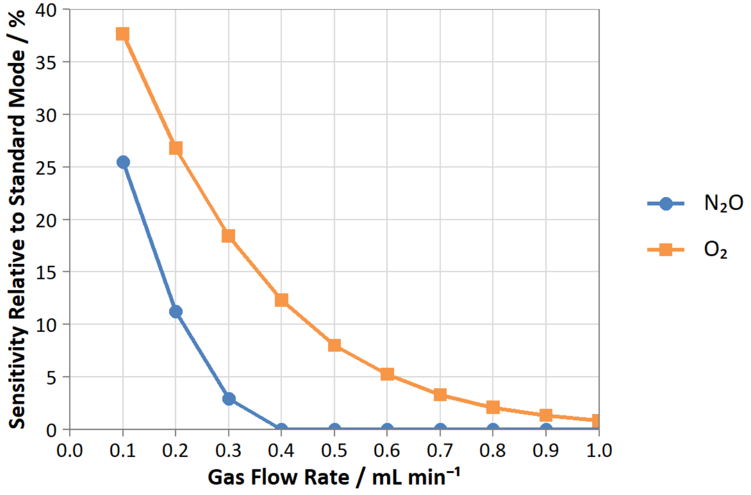

On-Mass Removal (as Interference) with N_2O

Variation of $^{45}Sc^{16}O^+$ Interference with N_2O Flow Rate (On-Mass: Q1 = 61, Q3 = 61)

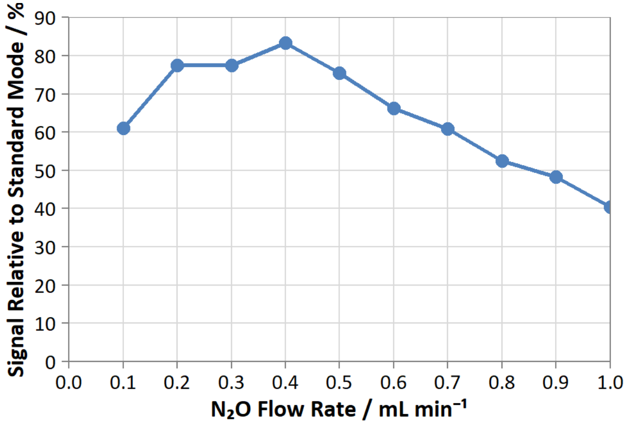

Variation of $^{45}Sc^{2+}$ Interference with N_2O Flow Rate (On-Mass: Q1 = 22.5, Q3 = 22.5)

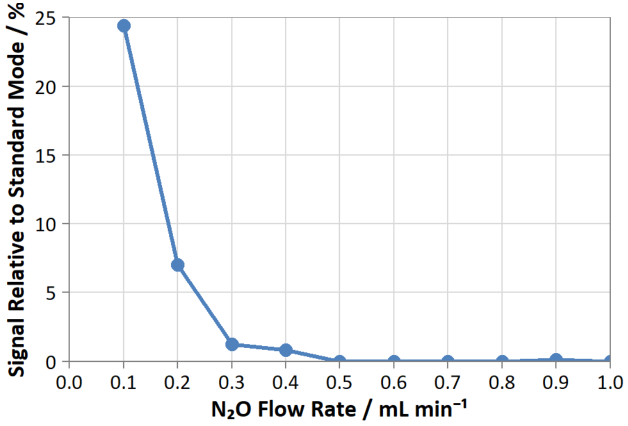

Asymmetric Charge Transfer (with N_2O)

[NO CHARGE TRANSFER REACTION OBSERVED]

Titanium

Supplementary Information B to: Characterisation of gas cell reactions for 70+ elements using N_2O for ICP tandem mass spectrometry measurements
Shaun T. Lancaster[a]*, Thomas Prohaska[ab] and Johanna Irrgeher[ab]
[a]Department of General, Analytical and Physical Chemistry, Chair of General and Analytical Chemistry, Montanuniversität Leoben, Leoben, Austria.
[b]Department of Physics and Astronomy, University of Calgary, Calgary, Canada.

Mass-Shift with N_2O and O_2

Major Product Ions of $^{47}Ti^+$ with N_2O Reaction Gas

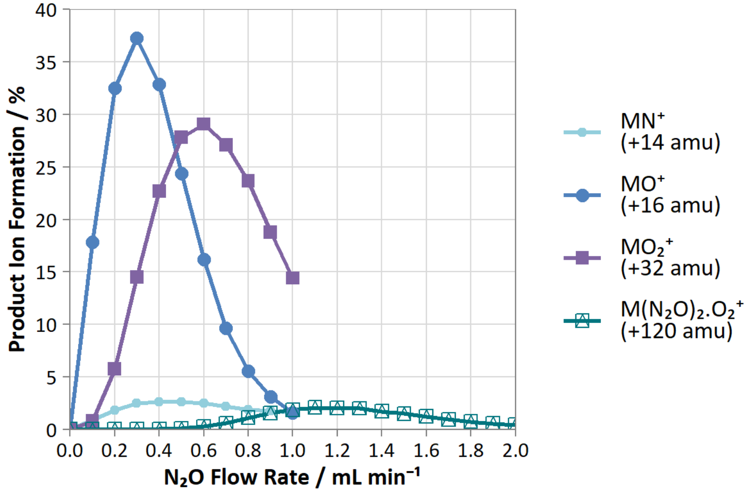

Major Product Ions of $^{47}Ti^+$ with O_2 Reaction Gas

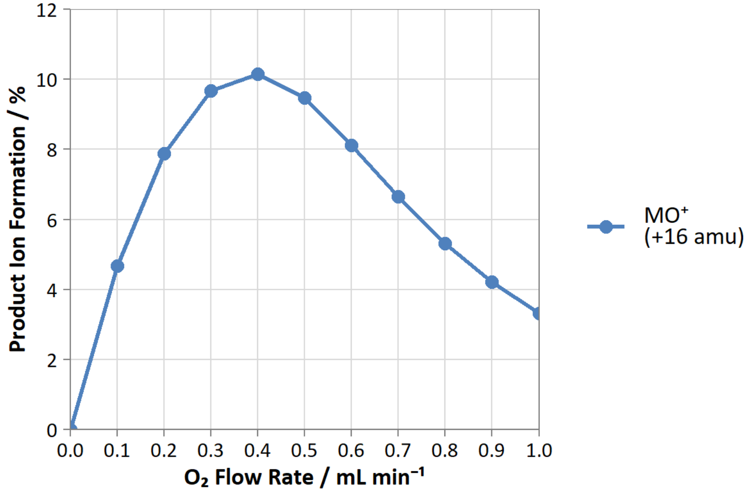

On-Mass with N_2O and O_2

On-Mass Profile of $^{47}Ti^+$ with N_2O and O_2

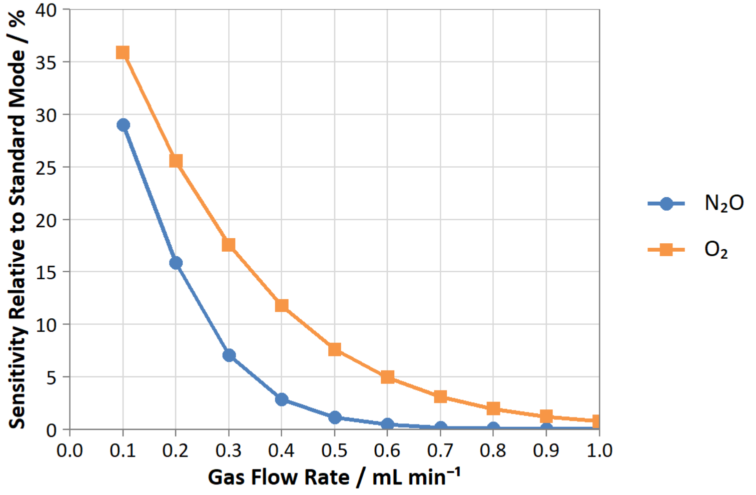

On-Mass Removal (as Interference) with N_2O

Variation of $^{47}Ti^{16}O^+$ Interference with N_2O Flow Rate (On-Mass: Q1 = 63, Q3 = 63)

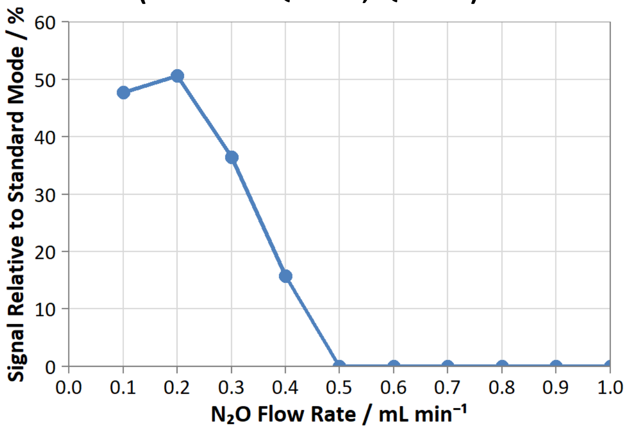

Variation of $^{47}Ti^{2+}$ Interference with N_2O Flow Rate (On-Mass: Q1 = 23.5, Q3 = 23.5)

[INSUFFICIENT DOUBLY-CHARGED ION FORMATION OBSERVED]

Asymmetric Charge Transfer (with N_2O)

[NO CHARGE TRANSFER REACTION OBSERVED]

Vanadium

Shaun T. Lancaster[a]*, Thomas Prohaska[ab] and Johanna Irrgeher[ab]
[a]Department of General, Analytical and Physical Chemistry, Chair of General and Analytical Chemistry, Montanuniversität Leoben, Leoben, Austria.
[b]Department of Physics and Astronomy, University of Calgary, Calgary, Canada.

Mass-Shift with N_2O and O_2

Major Product Ions of $^{51}V^+$ with N_2O Reaction Gas

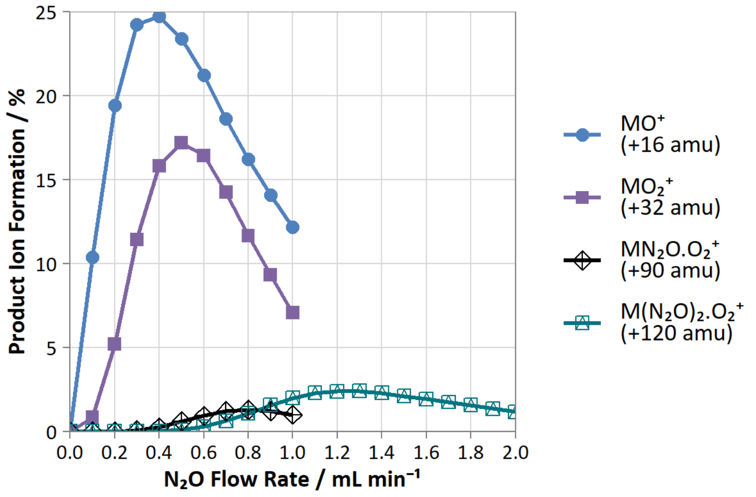

Major Product Ions of $^{51}V^+$ with O_2 Reaction Gas

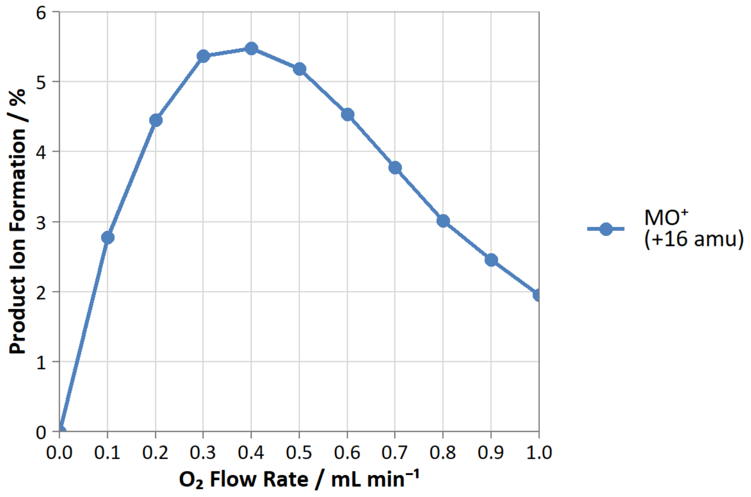

On-Mass with N_2O and O_2

On-Mass Profile of $^{51}V^+$ with N_2O and O_2

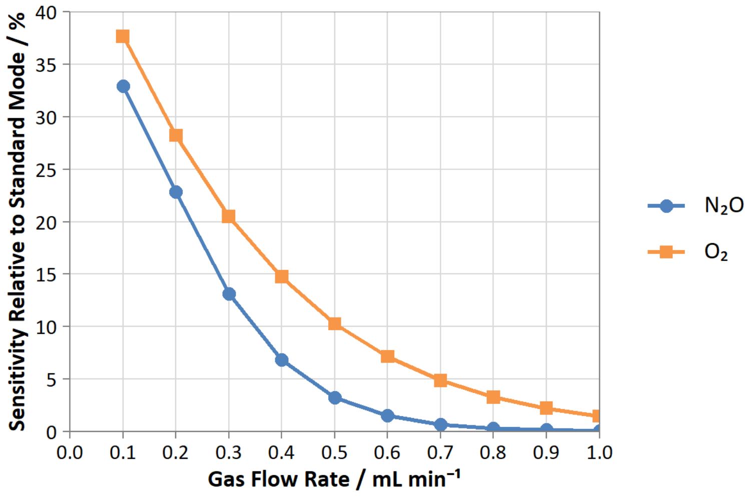

On-Mass Removal (as Interference) with N_2O

Variation of $^{51}V^{16}O^+$ Interference with N_2O Flow Rate (On-Mass: Q1 = 67, Q3 = 67)

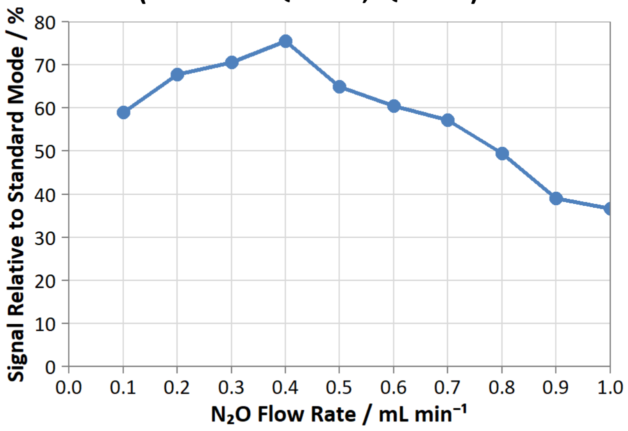

Variation of $^{51}V^{2+}$ Interference with N_2O Flow Rate (On-Mass: Q1 = 25.5, Q3 = 25.5)

[INSUFFICIENT DOUBLY-CHARGED ION FORMATION OBSERVED]

Asymmetric Charge Transfer (with N_2O)

[NO CHARGE TRANSFER REACTION OBSERVED]

Chromium

Supplementary Information B to: Characterisation of gas cell reactions for 70+ elements using N_2O for ICP tandem mass spectrometry measurements
Shaun T. Lancaster[a]*, Thomas Prohaska[ab] and Johanna Irrgeher[ab]
[a]Department of General, Analytical and Physical Chemistry, Chair of General and Analytical Chemistry, Montanuniversität Leoben, Leoben, Austria.
[b]Department of Physics and Astronomy, University of Calgary, Calgary, Canada.

Mass-Shift with N_2O and O_2

Major Product Ions of $^{52}Cr^+$ with N_2O Reaction Gas

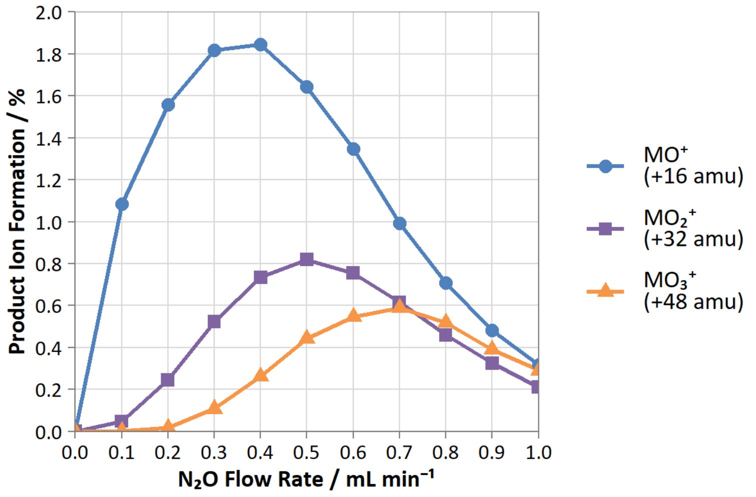

Major Product Ions of $^{52}Cr^+$ with O_2 Reaction Gas

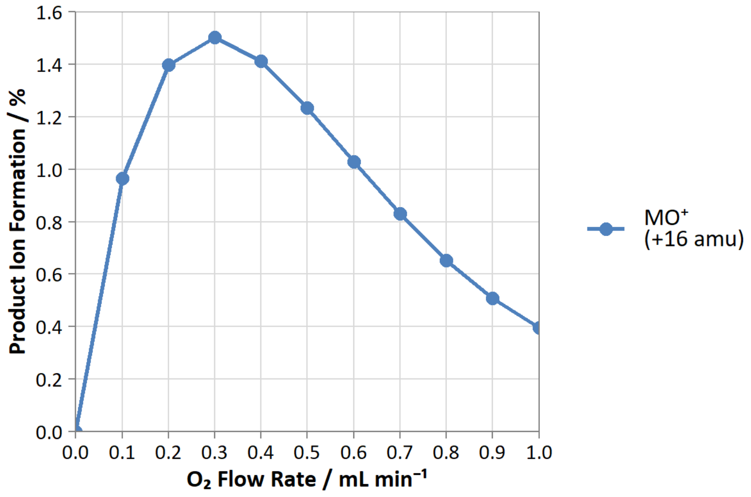

On-Mass with N_2O and O_2

On-Mass Profile of $^{52}Cr^+$ with N_2O and O_2

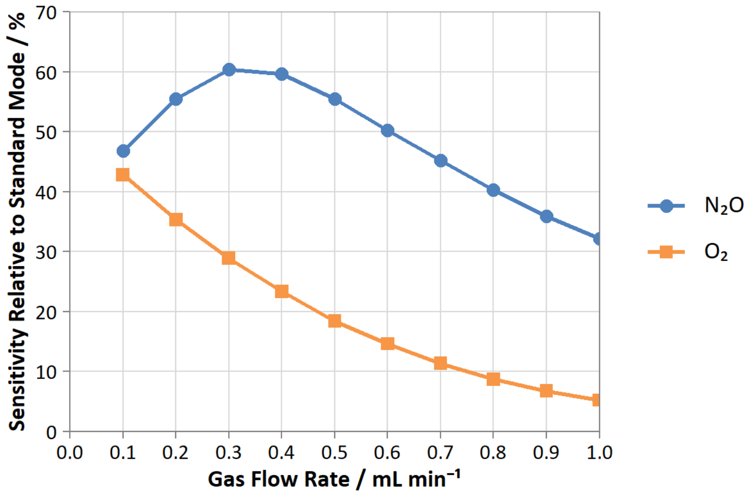

On-Mass Removal (as Interference) with N_2O

Variation of $^{52}Cr^{16}O^+$ Interference with N_2O Flow Rate (On-Mass: Q1 = 68, Q3 = 68)

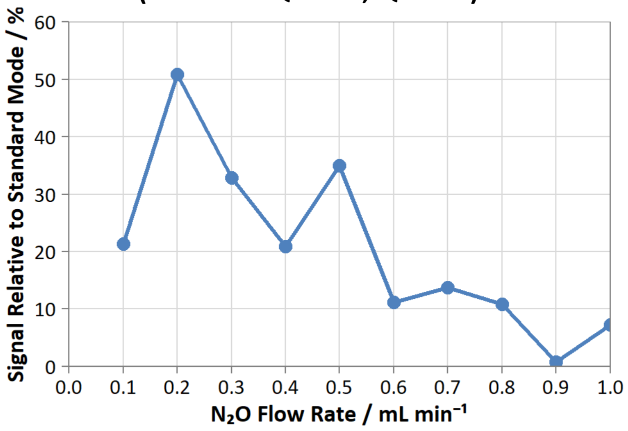

Variation of $^{52}Cr^{2+}$ Interference with N_2O Flow Rate (On-Mass: Q1 = 26, Q3 = 26)

[INSUFFICIENT DOUBLY-CHARGED ION FORMATION OBSERVED]

Asymmetric Charge Transfer (with N_2O)

[NO CHARGE TRANSFER REACTION OBSERVED]

Manganese

Supplementary Information B to: Characterisation of gas cell reactions for 70+ elements using N_2O for ICP tandem mass spectrometry measurements
Shaun T. Lancaster[a]*, Thomas Prohaska[ab] and Johanna Irrgeher[ab]
[a]Department of General, Analytical and Physical Chemistry, Chair of General and Analytical Chemistry, Montanuniversität Leoben, Leoben, Austria.
[b]Department of Physics and Astronomy, University of Calgary, Calgary, Canada.

Mass-Shift with N_2O and O_2

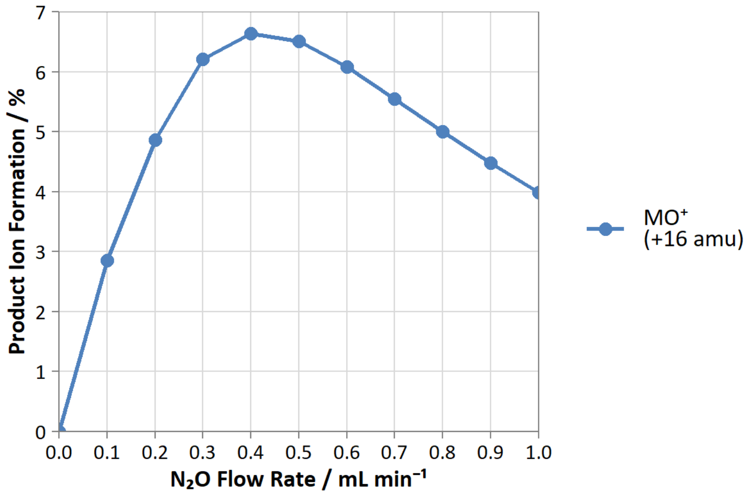

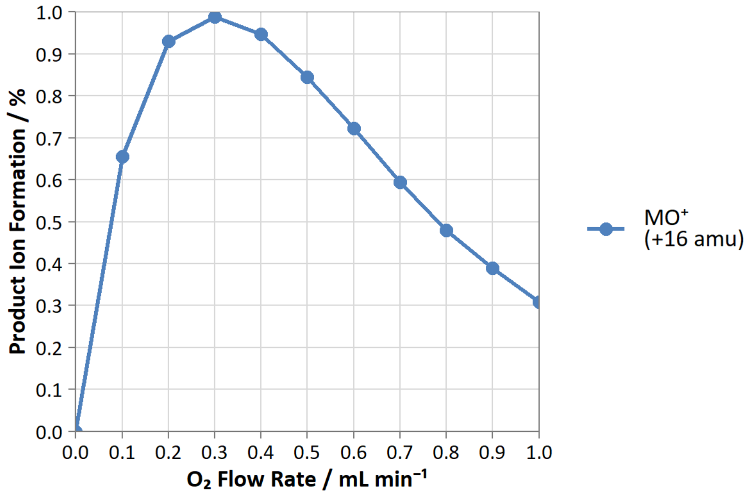

On-Mass with N_2O and O_2

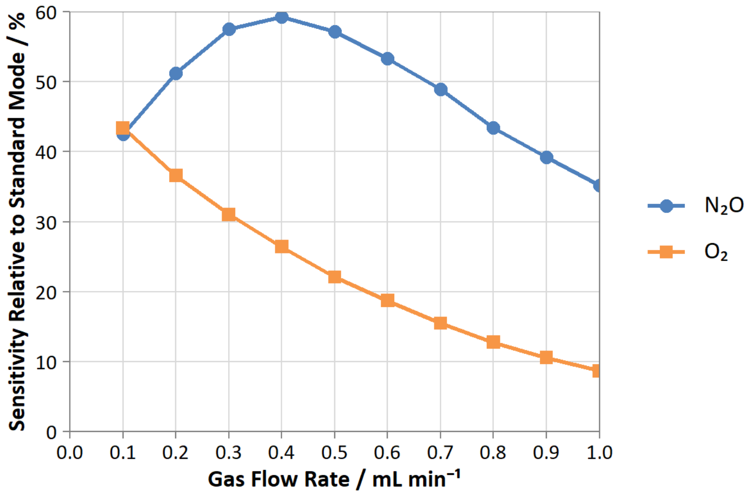

On-Mass Removal (as Interference) with N_2O

Variation of $^{55}Mn^{16}O^+$ Interference with N_2O Flow Rate (On-Mass: Q1 = 71, Q3 = 71)

[INSUFFICIENT OXIDE FORMATION OBSERVED]

Variation of $^{55}Mn^{2+}$ Interference with N_2O Flow Rate (On-Mass: Q1 = 27.5, Q3 = 27.5)

[INSUFFICIENT DOUBLY-CHARGED ION FORMATION OBSERVED]

Asymmetric Charge Transfer (with N_2O)

[NO CHARGE TRANSFER REACTION OBSERVED]

Iron

Supplementary Information B to: Characterisation of gas cell reactions for 70+ elements using N_2O for ICP tandem mass spectrometry measurements
Shaun T. Lancaster[a*], Thomas Prohaska[ab] and Johanna Irrgeher[ab]
[a]Department of General, Analytical and Physical Chemistry, Chair of General and Analytical Chemistry, Montanuniversität Leoben, Leoben, Austria.
[b]Department of Physics and Astronomy, University of Calgary, Calgary, Canada.

Mass-Shift with N_2O and O_2

Major Product Ions of $^{57}Fe^+$ with N_2O Reaction Gas

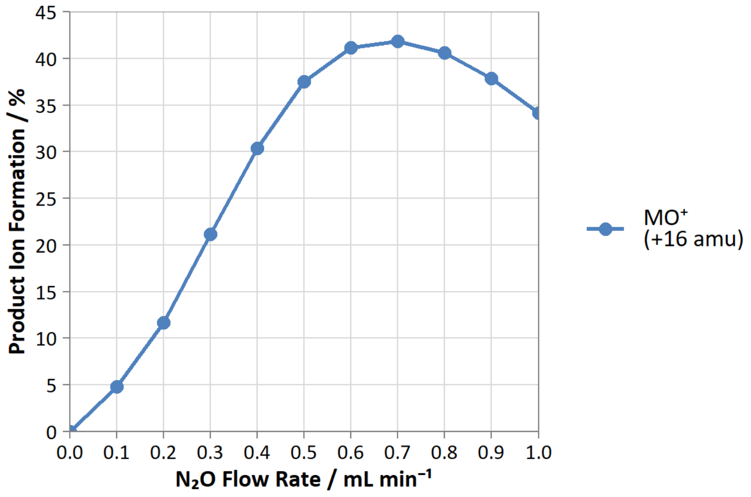

Major Product Ions of $^{57}Fe^+$ with O_2 Reaction Gas

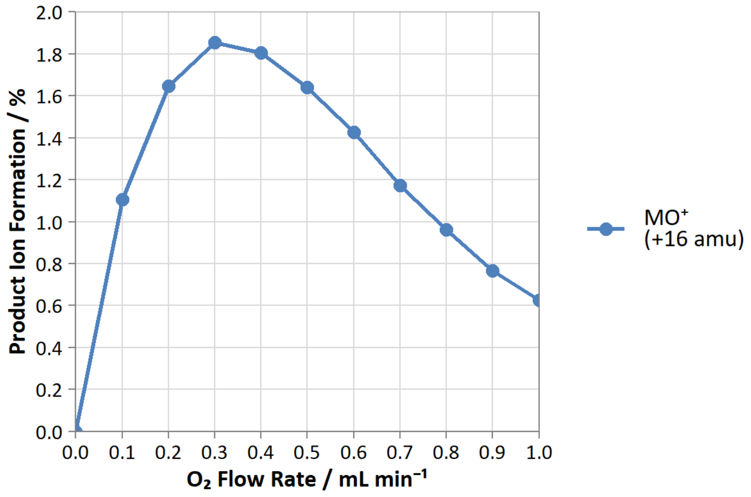

On-Mass with N_2O and O_2

On-Mass Profile of $^{57}Fe^+$ with N_2O and O_2

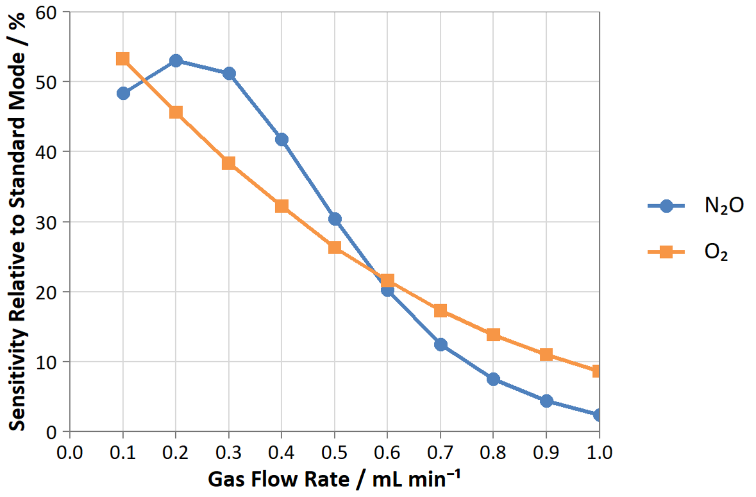

On-Mass Removal (as Interference) with N_2O

Variation of $^{57}Fe^{16}O^+$ Interference with N_2O Flow Rate (On-Mass: Q1 = 73, Q3 = 73)

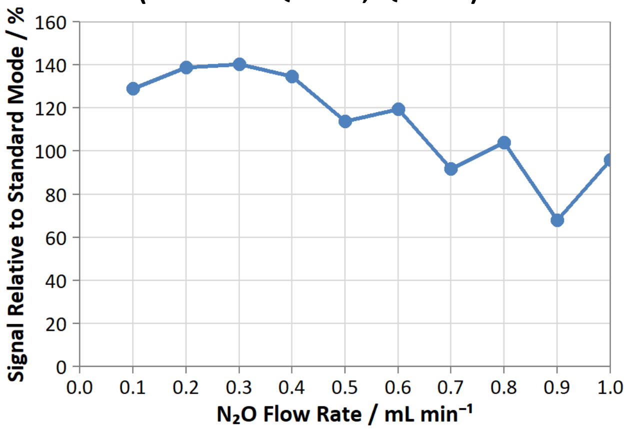

Variation of $^{57}Fe^{2+}$ Interference with N_2O Flow Rate (On-Mass: Q1 = 28.5, Q3 = 28.5)

[INSUFFICIENT DOUBLY-CHARGED ION FORMATION OBSERVED]

Asymmetric Charge Transfer (with N_2O)

[NO CHARGE TRANSFER REACTION OBSERVED]

Cobalt

Supplementary Information B to: Characterisation of gas cell reactions for 70+ elements using N_2O for ICP tandem mass spectrometry measurements
Shaun T. Lancaster[a]*, Thomas Prohaska[ab] and Johanna Irrgeher[ab]
[a]Department of General, Analytical and Physical Chemistry, Chair of General and Analytical Chemistry, Montanuniversität Leoben, Leoben, Austria.
[b]Department of Physics and Astronomy, University of Calgary, Calgary, Canada.

Mass-Shift with N_2O and O_2

Major Product Ions of $^{59}Co^+$ with N_2O Reaction Gas

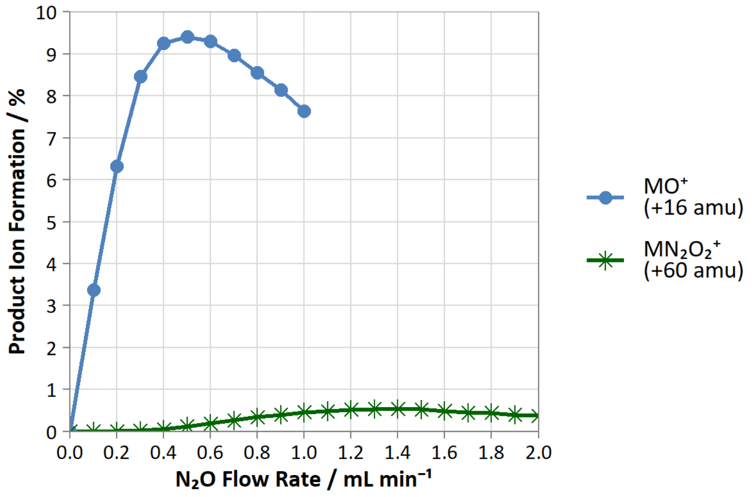

Major Product Ions of $^{59}Co^+$ with O_2 Reaction Gas

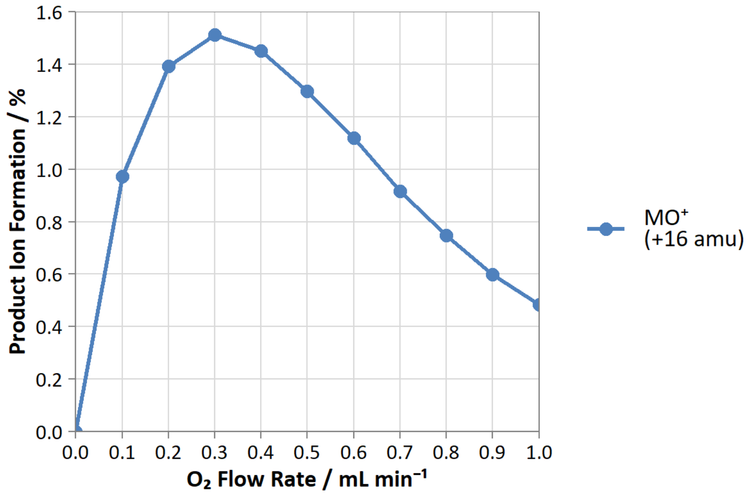

On-Mass with N_2O and O_2

On-Mass Profile of $^{59}Co^+$ with N_2O and O_2

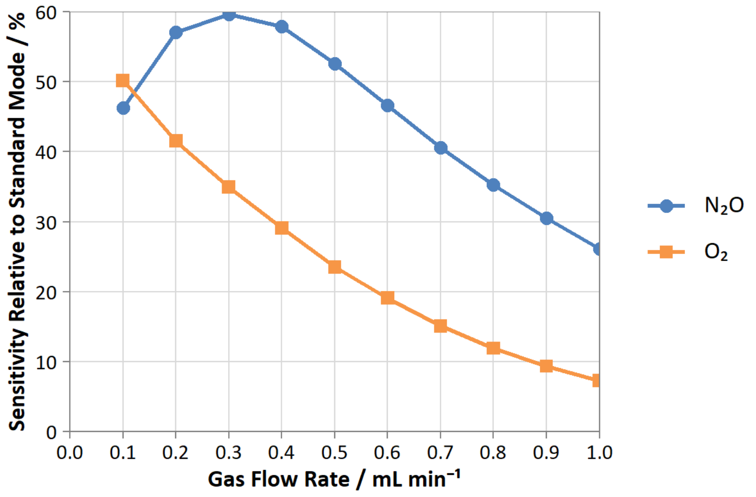

On-Mass Removal (as Interference) with N_2O

Variation of $^{59}Co^{16}O^+$ Interference with N_2O Flow Rate (On-Mass: Q1 = 75, Q3 = 75)

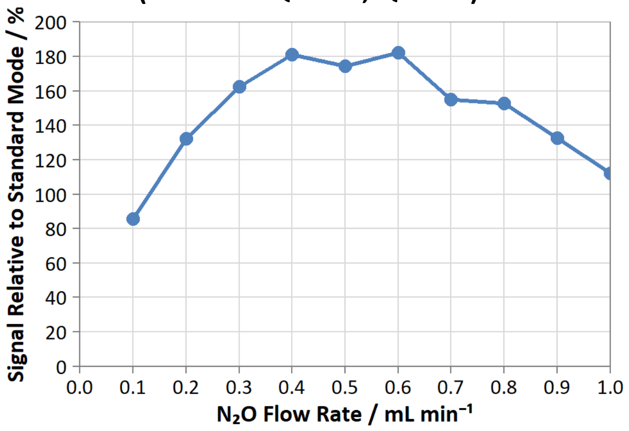

Variation of $^{59}Co^{2+}$ Interference with N_2O Flow Rate (On-Mass: Q1 = 29.5, Q3 = 29.5)

[INSUFFICIENT DOUBLY-CHARGED ION FORMATION OBSERVED]

Asymmetric Charge Transfer (with N_2O)

[NO CHARGE TRANSFER REACTION OBSERVED]

Nickel

Shaun T. Lancaster[a]*, Thomas Prohaska[ab] and Johanna Irrgeher[ab]
[a]Department of General, Analytical and Physical Chemistry, Chair of General and Analytical Chemistry, Montanuniversität Leoben, Leoben, Austria.
[b]Department of Physics and Astronomy, University of Calgary, Calgary, Canada.

Mass-Shift with N_2O and O_2

Major Product Ions of $^{60}Ni^+$ with N_2O Reaction Gas

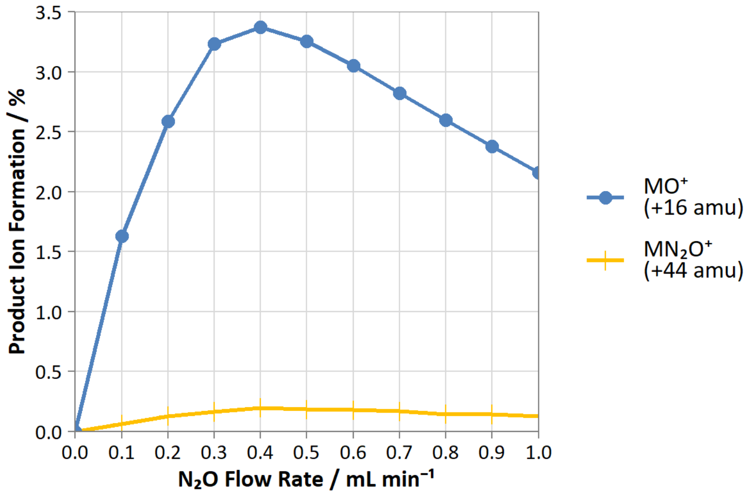

Major Product Ions of $^{60}Ni^+$ with O_2 Reaction Gas

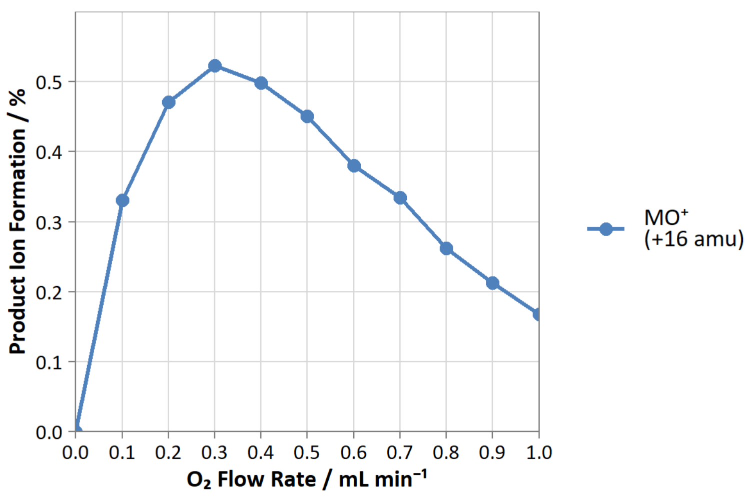

On-Mass with N_2O and O_2

On-Mass Profile of $^{60}Ni^+$ with N_2O and O_2

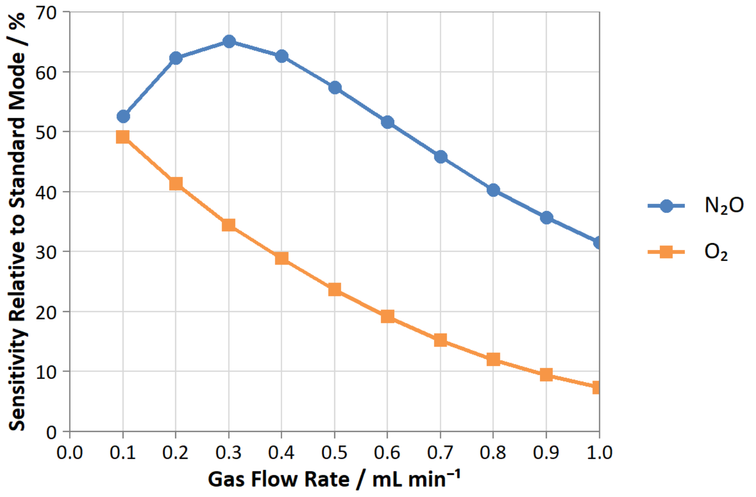

On-Mass Removal (as Interference) with N_2O

Variation of $^{60}Ni^{16}O^+$ Interference with
N_2O Flow Rate
(On-Mass: Q1 = 76, Q3 = 76)

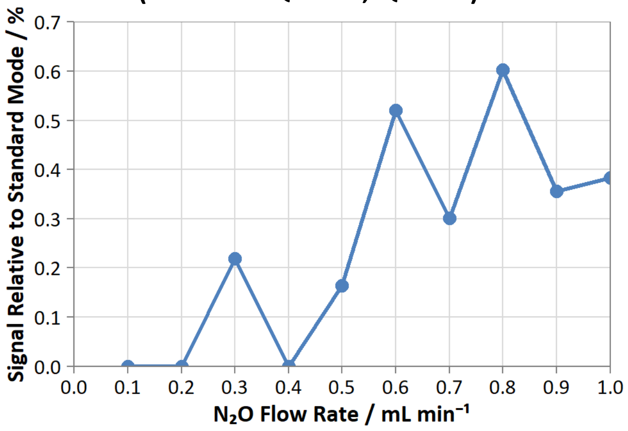

Variation of $^{60}Ni^{2+}$ Interference with
N_2O Flow Rate
(On-Mass: Q1 = 30, Q3 = 30)

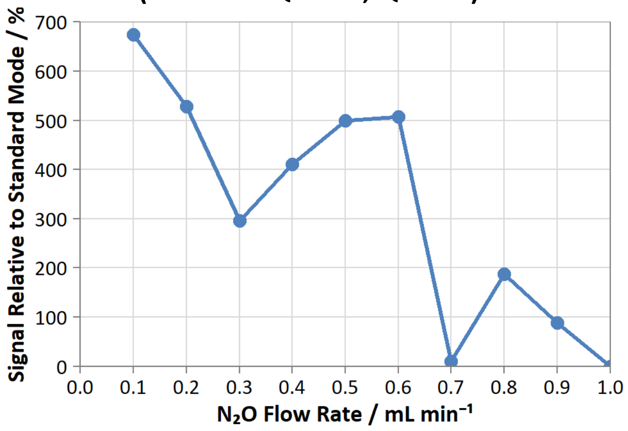

Asymmetric Charge Transfer (with N_2O)

[NO CHARGE TRANSFER REACTION OBSERVED]

Copper

Supplementary Information B to: Characterisation of gas cell reactions for 70+ elements using N_2O for ICP tandem mass spectrometry measurements
Shaun T. Lancaster[a]*, Thomas Prohaska[ab] and Johanna Irrgeher[ab]
[a]Department of General, Analytical and Physical Chemistry, Chair of General and Analytical Chemistry, Montanuniversität Leoben, Leoben, Austria.
[b]Department of Physics and Astronomy, University of Calgary, Calgary, Canada.

Mass-Shift with N_2O and O_2

Major Product Ions of $^{63}Cu^+$ with N_2O Reaction Gas

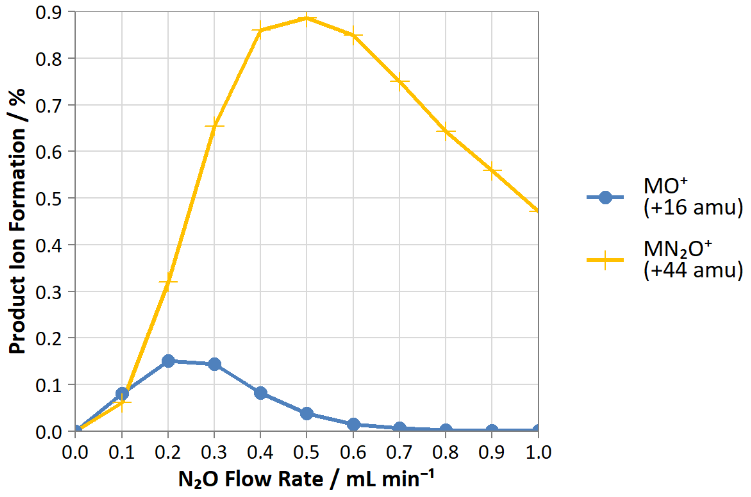

Major Product Ions of $^{63}Cu^+$ with O_2 Reaction Gas

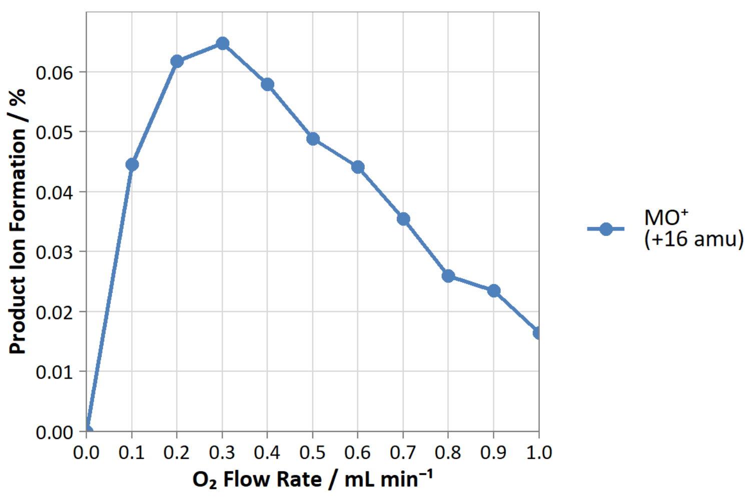

On-Mass with N_2O and O_2

On-Mass Profile of $^{63}Cu^+$ with N_2O and O_2

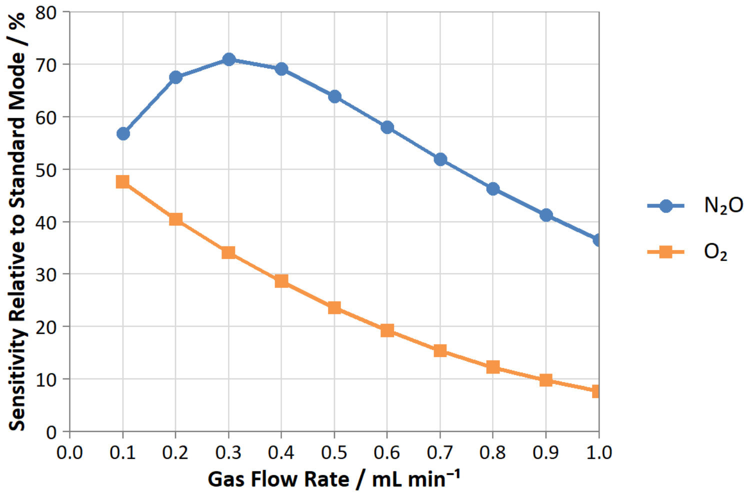

On-Mass Removal (as Interference) with N_2O

Variation of $^{63}Cu^{16}O^+$ Interference with N_2O Flow Rate (On-Mass: Q1 = 79, Q3 = 79)

[INSUFFICIENT OXIDE FORMATION OBSERVED]

Variation of $^{63}Cu^{2+}$ Interference with N_2O Flow Rate (On-Mass: Q1 = 31.5, Q3 = 31.5)

[INSUFFICIENT DOUBLY-CHARGED ION FORMATION OBSERVED]

Asymmetric Charge Transfer (with N_2O)

[NO CHARGE TRANSFER REACTION OBSERVED]

Zinc

Shaun T. Lancaster[a*], Thomas Prohaska[ab] and Johanna Irrgeher[ab]
[a]Department of General, Analytical and Physical Chemistry, Chair of General and Analytical Chemistry, Montanuniversität Leoben, Leoben, Austria.
[b]Department of Physics and Astronomy, University of Calgary, Calgary, Canada.

Mass-Shift with N_2O and O_2

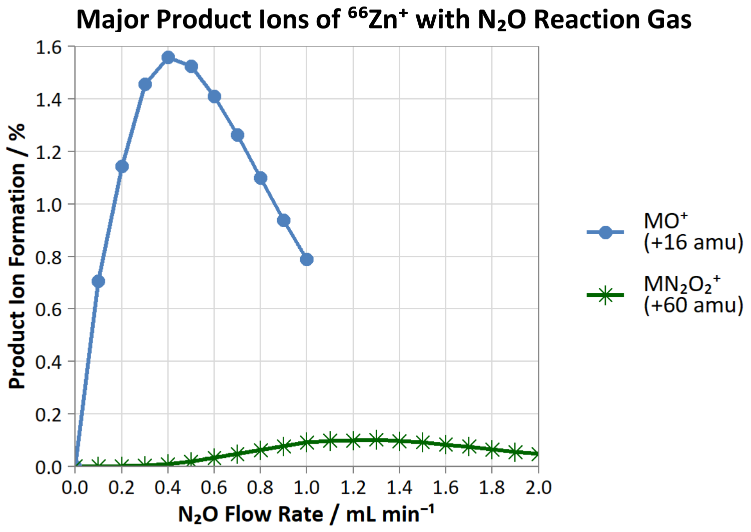

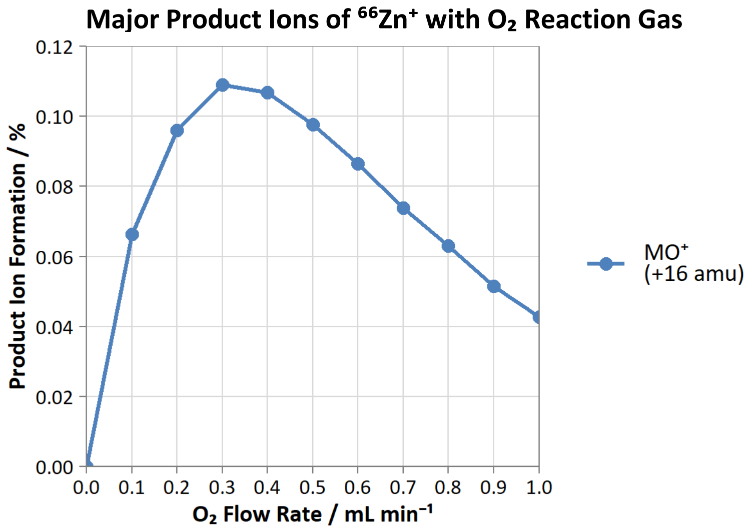

On-Mass with N_2O and O_2

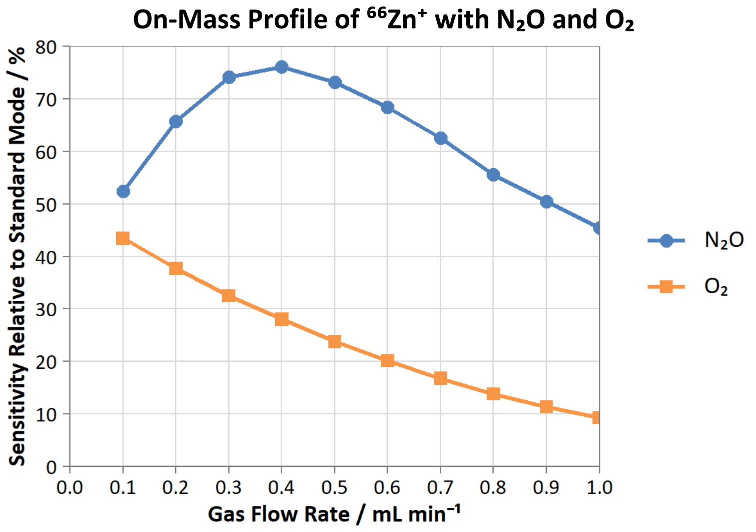

On-Mass Removal (as Interference) with N_2O

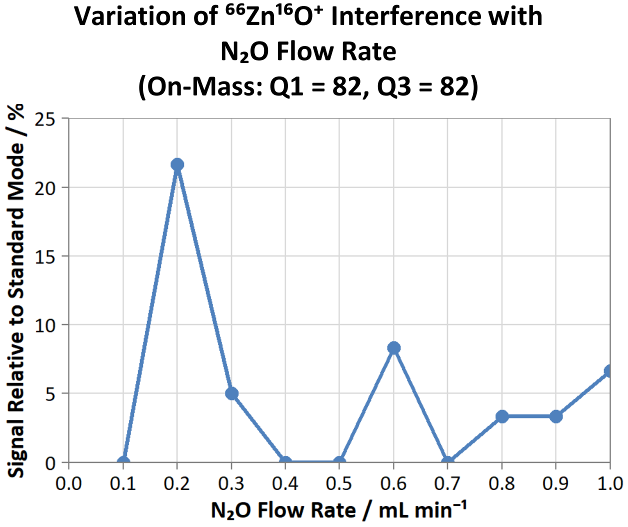

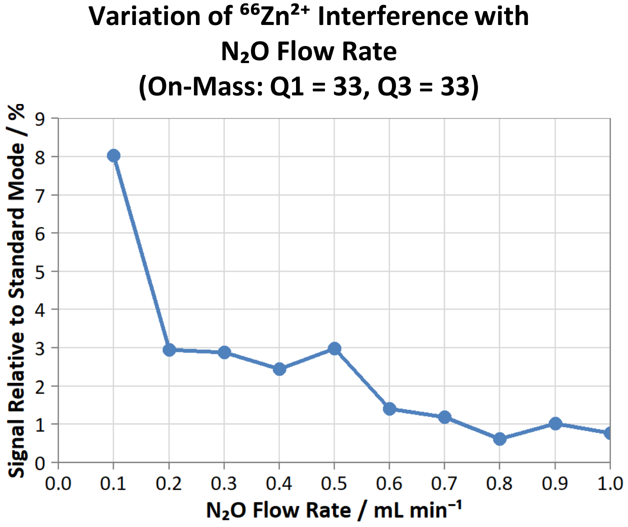

Asymmetric Charge Transfer (with N_2O)

[NO CHARGE TRANSFER REACTION OBSERVED]

Gallium

Supplementary Information B to: Characterisation of gas cell reactions for 70+ elements using N_2O for ICP tandem mass spectrometry measurements
Shaun T. Lancaster[a*], Thomas Prohaska[ab] and Johanna Irrgeher[ab]
[a]Department of General, Analytical and Physical Chemistry, Chair of General and Analytical Chemistry, Montanuniversität Leoben, Leoben, Austria.
[b]Department of Physics and Astronomy, University of Calgary, Calgary, Canada.

Mass-Shift with N_2O and O_2

Major Product Ions of $^{69}Ga^+$ with N_2O Reaction Gas

[NO ATOM TRANSFER REACTION OBSERVED]

Major Product Ions of $^{69}Ga^+$ with O_2 Reaction Gas

[NO ATOM TRANSFER REACTION OBSERVED]

On-Mass with N_2O and O_2

On-Mass Profile of $^{69}Ga^+$ with N_2O and O_2

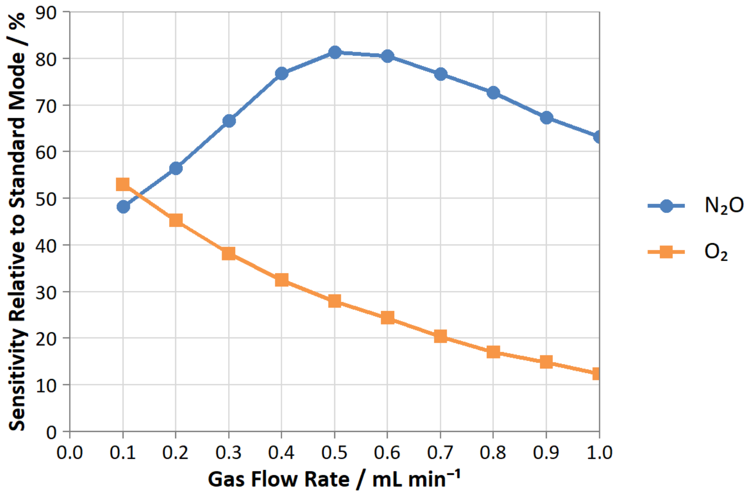

On-Mass Removal (as Interference) with N_2O

Variation of $^{69}Ga^{16}O^+$ Interference with N_2O Flow Rate (On-Mass: Q1 = 85, Q3 = 85)

[INSUFFICIENT OXIDE FORMATION OBSERVED]

Variation of $^{69}Ga^{2+}$ Interference with N_2O Flow Rate (On-Mass: Q1 = 34.5, Q3 = 34.5)

[INSUFFICIENT DOUBLY-CHARGED ION FORMATION OBSERVED]

Asymmetric Charge Transfer (with N_2O)

[NO CHARGE TRANSFER REACTION OBSERVED]

Germanium

Supplementary Information B to: Characterisation of gas cell reactions for 70+ elements using N_2O for ICP tandem mass spectrometry measurements
Shaun T. Lancaster[a]*, Thomas Prohaska[ab] and Johanna Irrgeher[ab]
[a]Department of General, Analytical and Physical Chemistry, Chair of General and Analytical Chemistry, Montanuniversität Leoben, Leoben, Austria.
[b]Department of Physics and Astronomy, University of Calgary, Calgary, Canada.

Mass-Shift with N_2O and O_2

Major Product Ions of $^{74}Ge^+$ with N_2O Reaction Gas

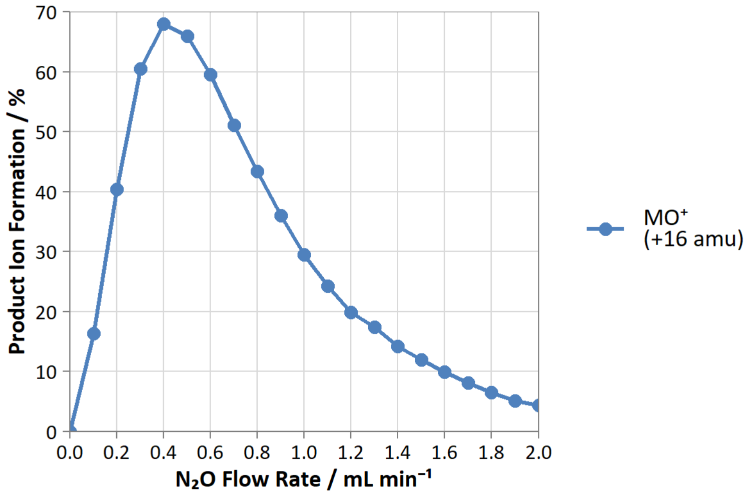

Major Product Ions of $^{74}Ge^+$ with O_2 Reaction Gas

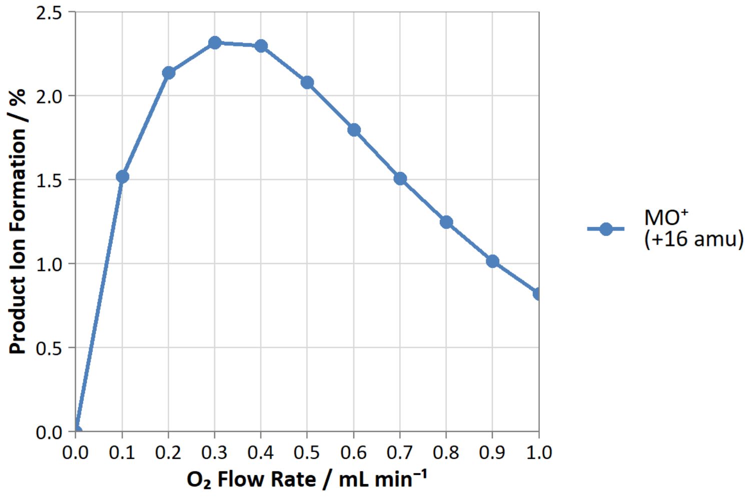

On-Mass with N_2O and O_2

On-Mass Profile of $^{74}Ge^+$ with N_2O and O_2

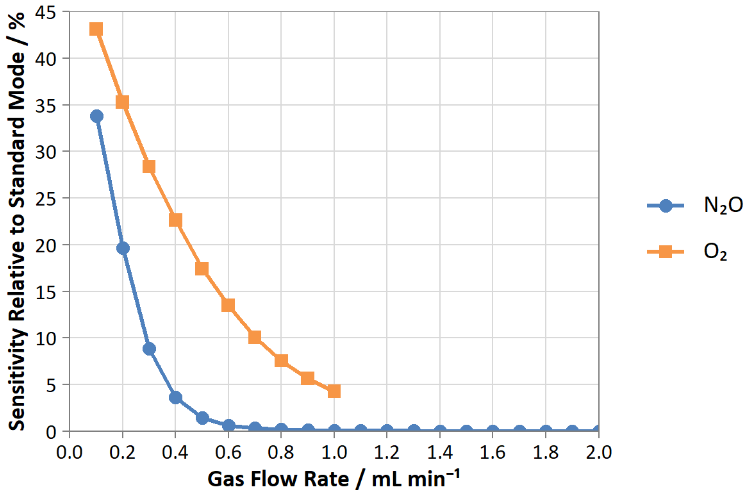

On-Mass Removal (as Interference) with N_2O

Variation of $^{74}Ge^{16}O^+$ Interference with N_2O Flow Rate (On-Mass: Q1 = 90, Q3 = 90)

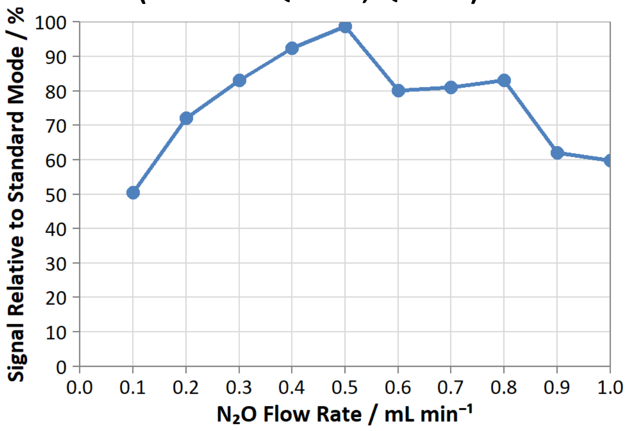

Variation of $^{74}Ge^{2+}$ Interference with N_2O Flow Rate (On-Mass: Q1 = 37, Q3 = 37)

[NOT MEASURED]

Asymmetric Charge Transfer (with N_2O)

Profile of Charge Transfer Reaction Between $^{74}Ge_{(m)}^+$ and N_2O

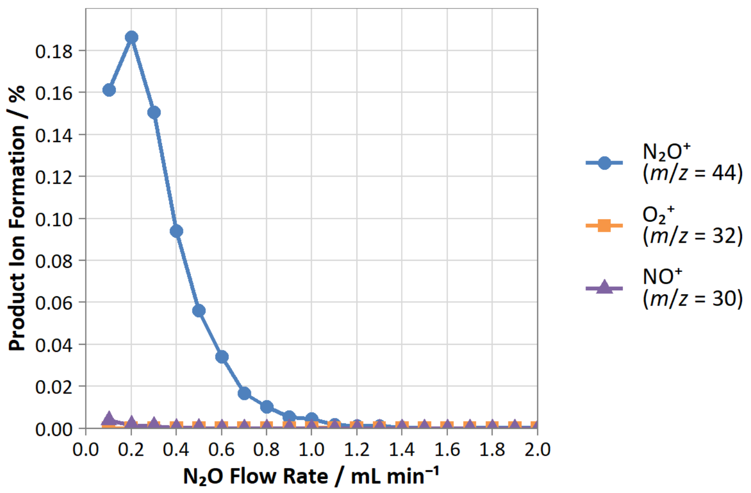

Arsenic

Supplementary Information B to: Characterisation of gas cell reactions for 70+ elements using N_2O for ICP tandem mass spectrometry measurements

Shaun T. Lancaster[a]*, Thomas Prohaska[ab] and Johanna Irrgeher[ab]
[a]Department of General, Analytical and Physical Chemistry, Chair of General and Analytical Chemistry, Montanuniversität Leoben, Leoben, Austria.
[b]Department of Physics and Astronomy, University of Calgary, Calgary, Canada.

Mass-Shift with N_2O and O_2

Major Product Ions of $^{75}As^+$ with N_2O Reaction Gas

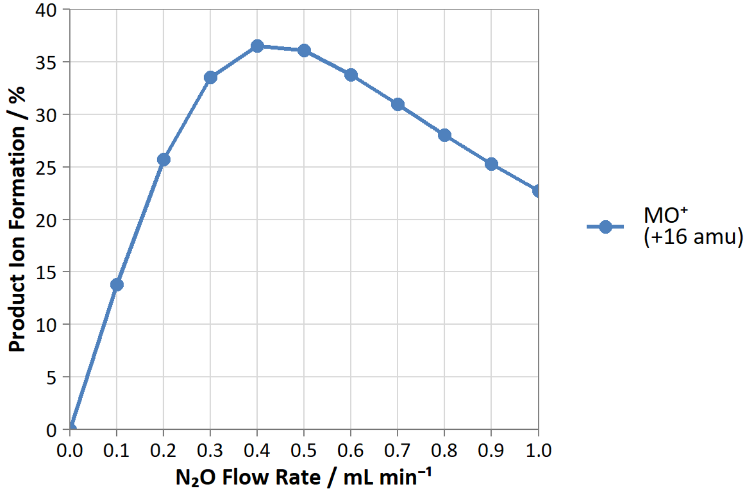

Major Product Ions of $^{75}As^+$ with O_2 Reaction Gas

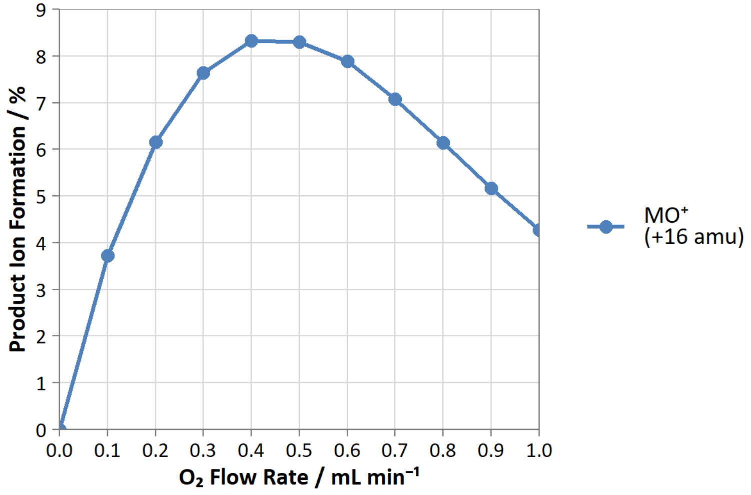

On-Mass with N_2O and O_2

On-Mass Profile of $^{75}As^+$ with N_2O and O_2

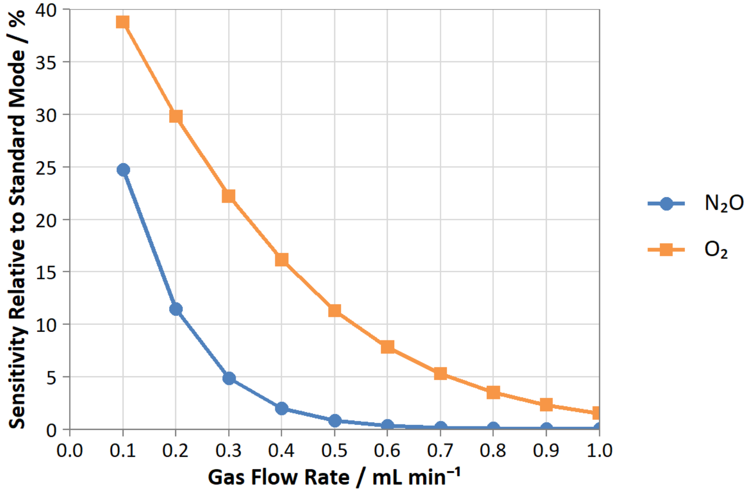

On-Mass Removal (as Interference) with N_2O

Variation of $^{75}As^{16}O^+$ Interference with N_2O Flow Rate (On-Mass: Q1 = 91, Q3 = 91)

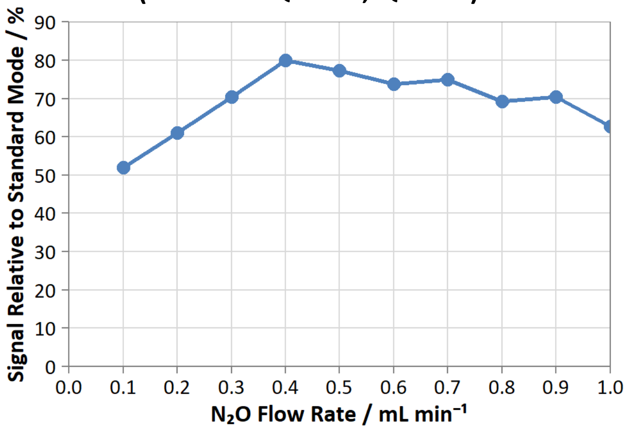

Variation of $^{75}As^{2+}$ Interference with N_2O Flow Rate (On-Mass: Q1 = 37.5, Q3 = 37.5)

[INSUFFICIENT DOUBLY-CHARGED ION FORMATION OBSERVED]

Asymmetric Charge Transfer (with N_2O)

Profile of Charge Transfer Reaction Between $^{75}As_{(m)}^+$ and N_2O

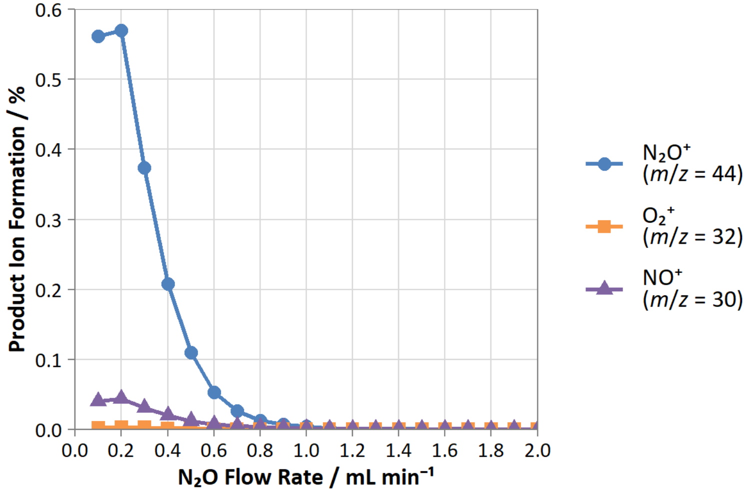

Selenium

Supplementary Information B to: Characterisation of gas cell reactions for 70+ elements using N_2O for ICP tandem mass spectrometry measurements
Shaun T. Lancaster[a]*, Thomas Prohaska[ab] and Johanna Irrgeher[ab]
[a]Department of General, Analytical and Physical Chemistry, Chair of General and Analytical Chemistry, Montanuniversität Leoben, Leoben, Austria.
[b]Department of Physics and Astronomy, University of Calgary, Calgary, Canada.

Mass-Shift with N_2O and O_2

Major Product Ions of $^{82}Se^+$ with N_2O Reaction Gas

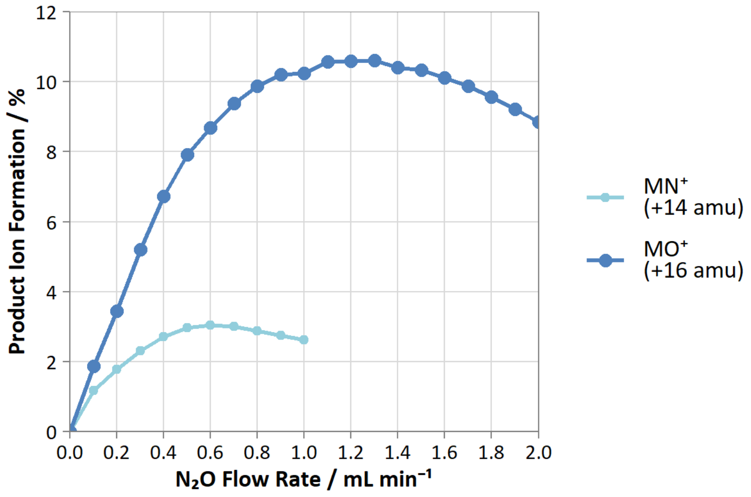

Major Product Ions of $^{82}Se^+$ with O_2 Reaction Gas

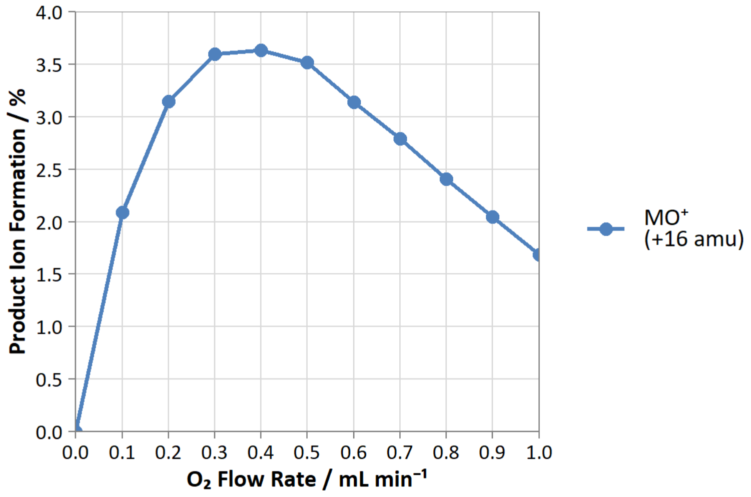

On-Mass with N_2O and O_2

On-Mass Profile of $^{82}Se^+$ with N_2O and O_2

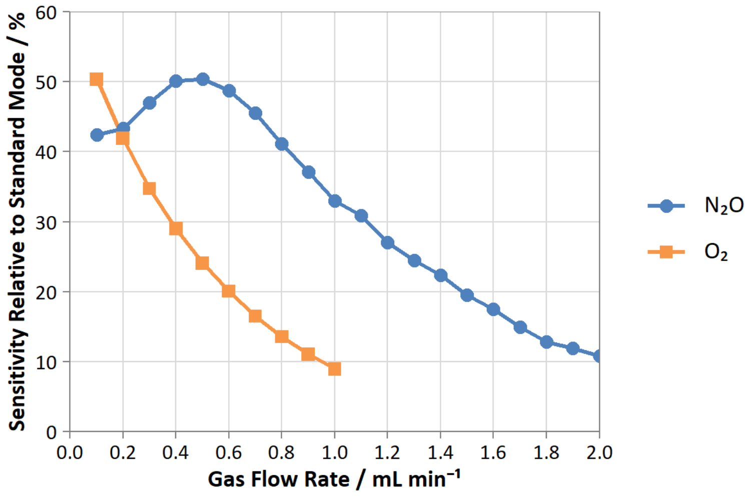

On-Mass Removal (as Interference) with N_2O

Variation of $^{78}Se^{16}O^+$ Interference with N_2O Flow Rate (On-Mass: Q1 = 94, Q3 = 94)

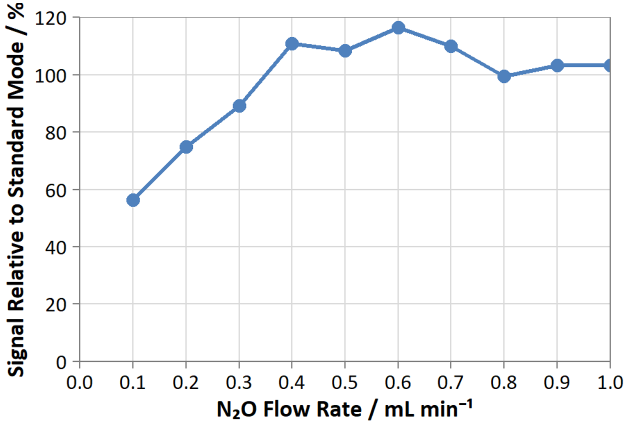

Variation of $^{78}Se^{2+}$ Interference with N_2O Flow Rate (On-Mass: Q1 = 39, Q3 = 39)

[NOT MEASURED]

Asymmetric Charge Transfer (with N_2O)

Profile of Charge Transfer Reaction Between $^{78}Se_{(m)}^+$ and N_2O

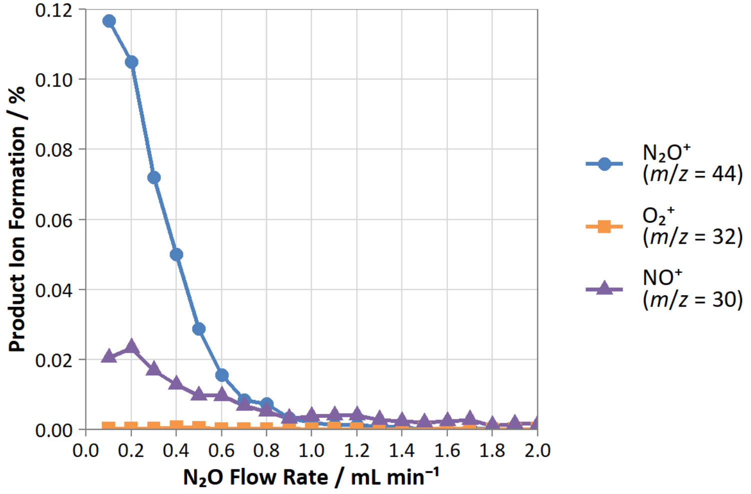

Bromine

Supplementary Information B to: Characterisation of gas cell reactions for 70+ elements using N_2O for ICP tandem mass spectrometry measurements
Shaun T. Lancaster[a]*, Thomas Prohaska[ab] and Johanna Irrgeher[ab]
[a]Department of General, Analytical and Physical Chemistry, Chair of General and Analytical Chemistry, Montanuniversität Leoben, Leoben, Austria.
[b]Department of Physics and Astronomy, University of Calgary, Calgary, Canada.

Mass-Shift with N_2O and O_2

Major Product Ions of $^{81}Br^+$ with N_2O Reaction Gas

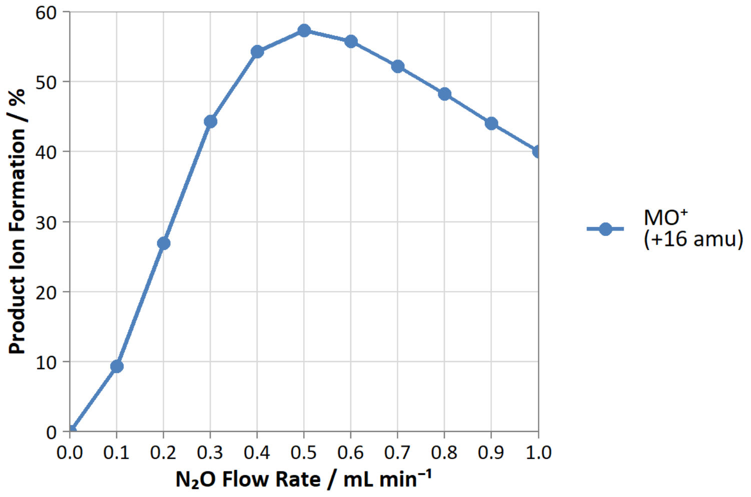

Major Product Ions of $^{81}Br^+$ with O_2 Reaction Gas

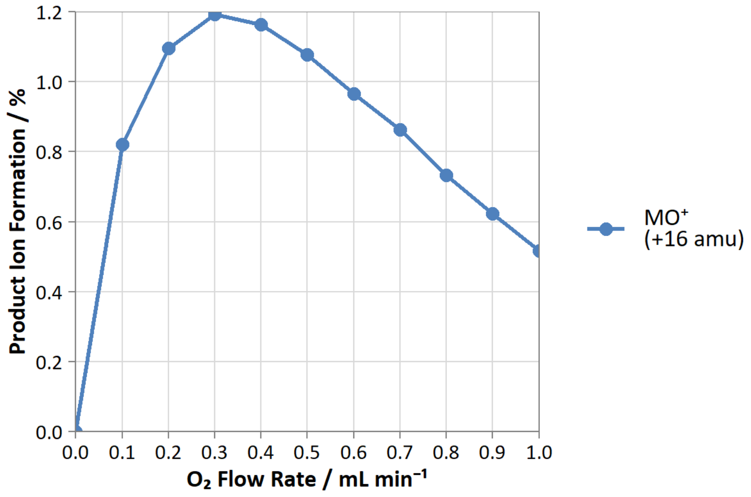

On-Mass with N_2O and O_2

On-Mass Profile of $^{81}Br^+$ with N_2O and O_2

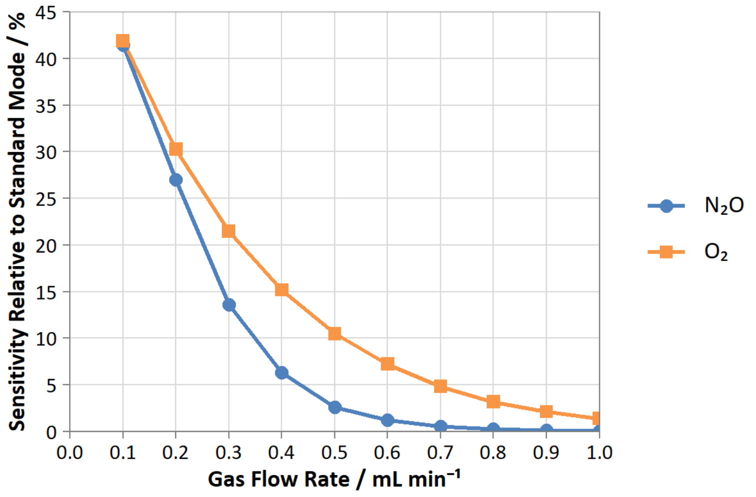

On-Mass Removal (as Interference) with N_2O

Variation of $^{81}Br^{16}O^+$ Interference with N_2O Flow Rate (On-Mass: Q1 = 97, Q3 = 97)

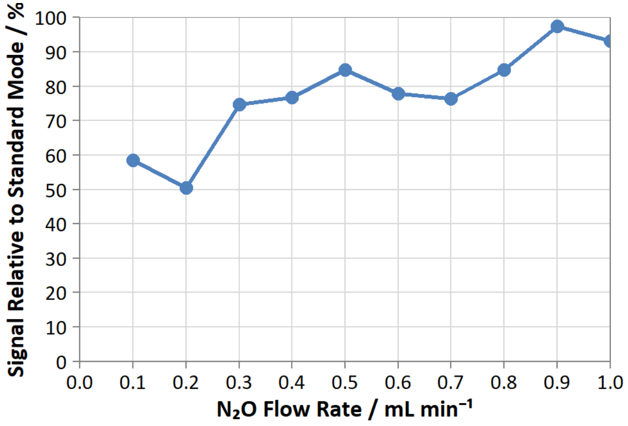

Variation of $^{81}Br^{2+}$ Interference with N_2O Flow Rate (On-Mass: Q1 = 40.5, Q3 = 40.5)

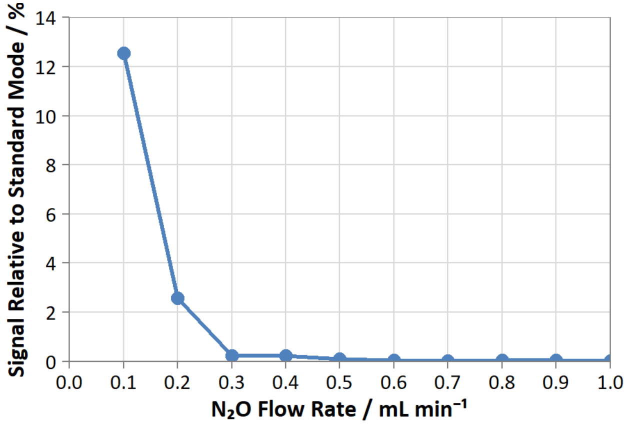

Asymmetric Charge Transfer (with N_2O)

Profile of Charge Transfer Reaction Between $^{81}Br_{(m)}^+$ and N_2O

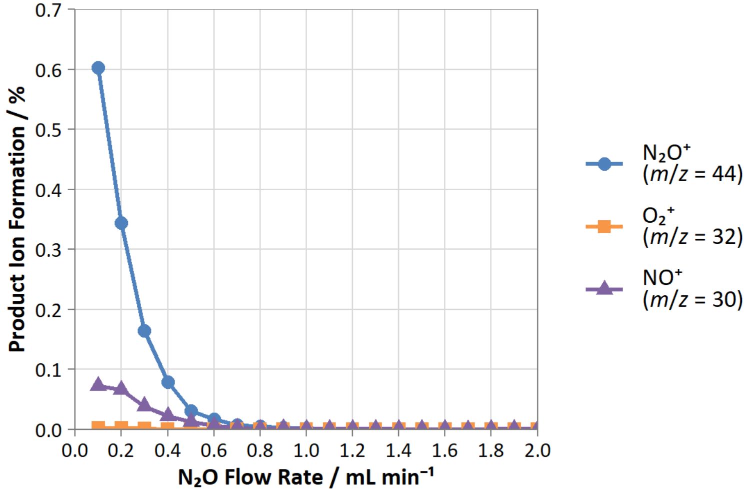

Rubidium

Supplementary Information B to: Characterisation of gas cell reactions for 70+ elements using N_2O for ICP tandem mass spectrometry measurements
Shaun T. Lancaster[a*], Thomas Prohaska[ab] and Johanna Irrgeher[ab]
[a]Department of General, Analytical and Physical Chemistry, Chair of General and Analytical Chemistry, Montanuniversität Leoben, Leoben, Austria.
[b]Department of Physics and Astronomy, University of Calgary, Calgary, Canada.

Mass-Shift with N_2O and O_2

Major Product Ions of $^{85}Rb^+$ with N_2O Reaction Gas

[NO ATOM TRANSFER REACTION OBSERVED]

Major Product Ions of $^{85}Rb^+$ with O_2 Reaction Gas

[NO ATOM TRANSFER REACTION OBSERVED]

On-Mass with N_2O and O_2

On-Mass Profile of $^{85}Rb^+$ with N_2O and O_2

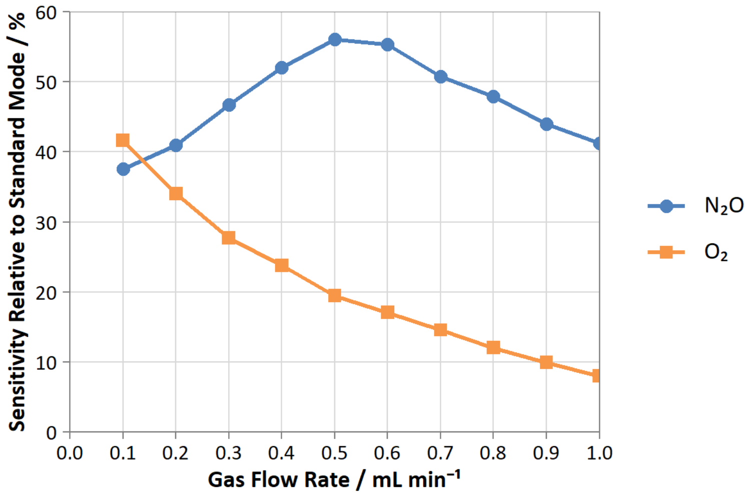

On-Mass Removal (as Interference) with N_2O

Variation of $^{85}Rb^{16}O^+$ Interference with N_2O Flow Rate (On-Mass: Q1 = 101, Q3 = 101)

[INSUFFICIENT OXIDE FORMATION OBSERVED]

Variation of $^{85}Rb^{2+}$ Interference with N_2O Flow Rate (On-Mass: Q1 = 42.5, Q3 = 42.5)

[INSUFFICIENT DOUBLY-CHARGED ION FORMATION OBSERVED]

Asymmetric Charge Transfer (with N_2O)

[NO CHARGE TRANSFER REACTION OBSERVED]

Strontium

Supplementary Information B to: Characterisation of gas cell reactions for 70+ elements using N_2O for ICP tandem mass spectrometry measurements
Shaun T. Lancaster[a]*, Thomas Prohaska[ab] and Johanna Irrgeher[ab]
[a]Department of General, Analytical and Physical Chemistry, Chair of General and Analytical Chemistry, Montanuniversität Leoben, Leoben, Austria.
[b]Department of Physics and Astronomy, University of Calgary, Calgary, Canada.

Mass-Shift with N_2O and O_2

Major Product Ions of $^{88}Sr^+$ with N_2O Reaction Gas

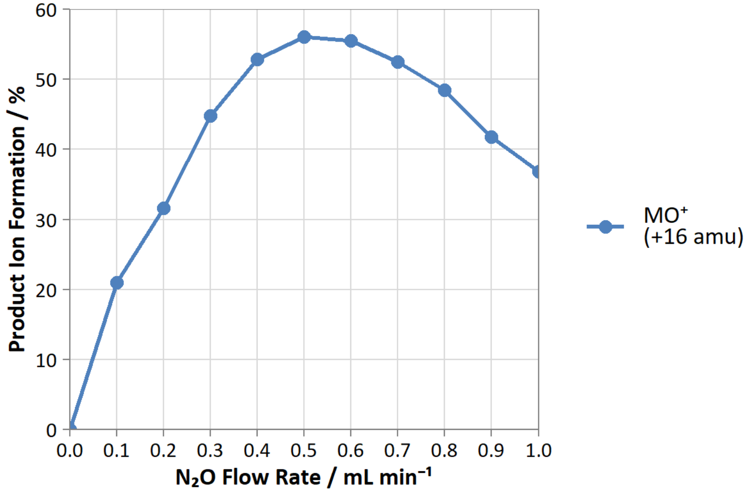

Major Product Ions of $^{88}Sr^+$ with O_2 Reaction Gas

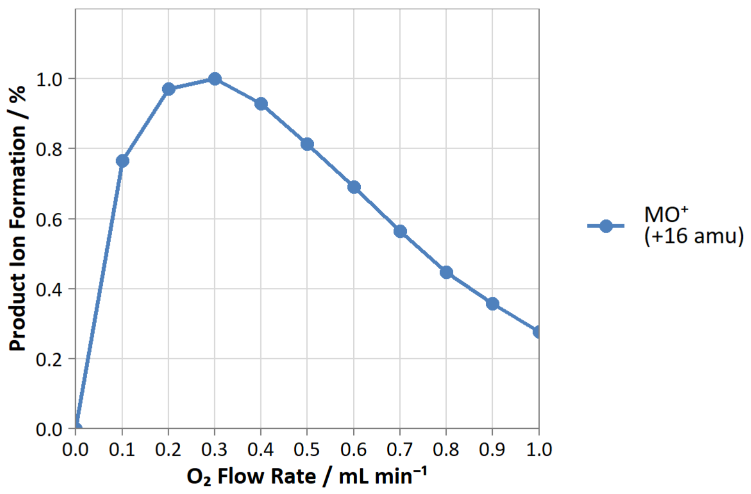

On-Mass with N_2O and O_2

On-Mass Profile of $^{88}Sr^+$ with N_2O and O_2

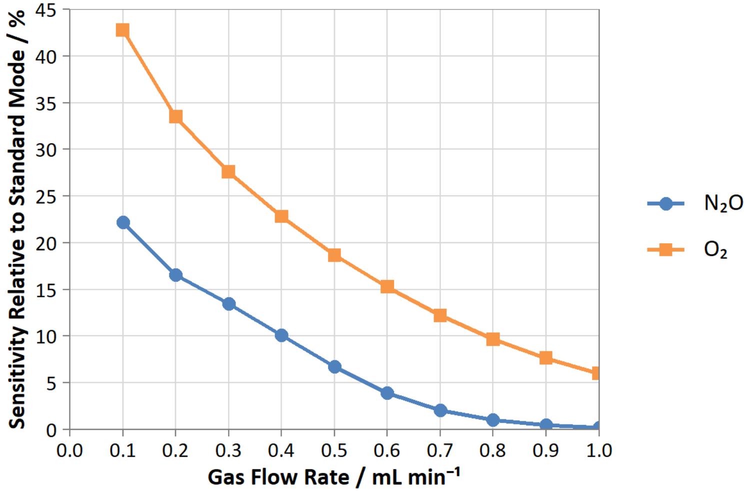

On-Mass Removal (as Interference) with N_2O

Variation of $^{88}Sr^{16}O^+$ Interference with N_2O Flow Rate (On-Mass: Q1 = 104, Q3 = 104)

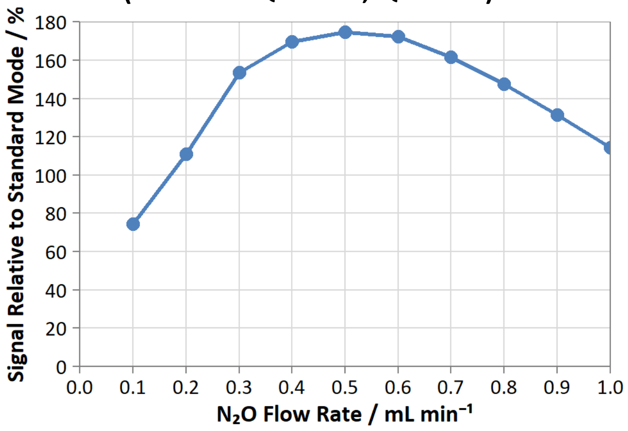

Variation of $^{88}Sr^{2+}$ Interference with N_2O Flow Rate (On-Mass: Q1 = 44, Q3 = 44)

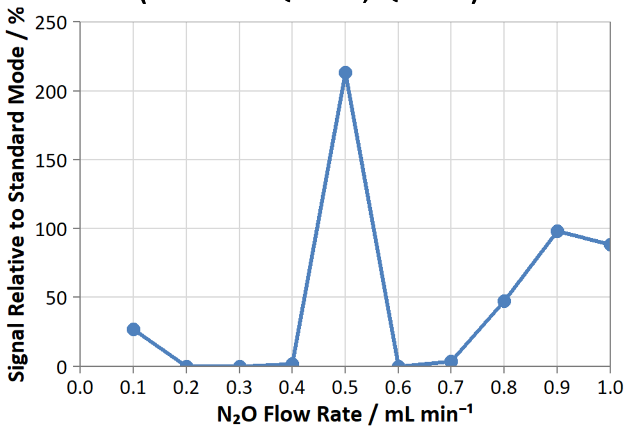

Asymmetric Charge Transfer (with N_2O)

[NO CHARGE TRANSFER REACTION OBSERVED]

Yttrium

Shaun T. Lancaster[a]*, Thomas Prohaska[ab] and Johanna Irrgeher[ab]
[a]Department of General, Analytical and Physical Chemistry, Chair of General and Analytical Chemistry, Montanuniversität Leoben, Leoben, Austria.
[b]Department of Physics and Astronomy, University of Calgary, Calgary, Canada.

Mass-Shift with N_2O and O_2

Major Product Ions of $^{89}Y^+$ with N_2O Reaction Gas

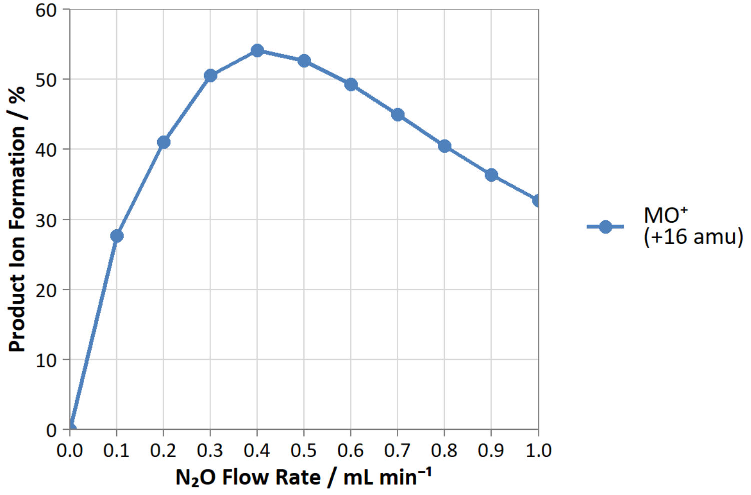

Major Product Ions of $^{89}Y^+$ with O_2 Reaction Gas

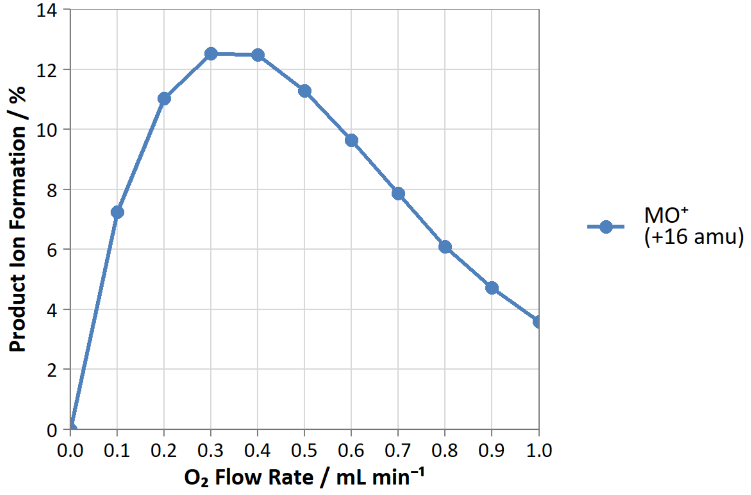

On-Mass with N_2O and O_2

On-Mass Profile of $^{89}Y^+$ with N_2O and O_2

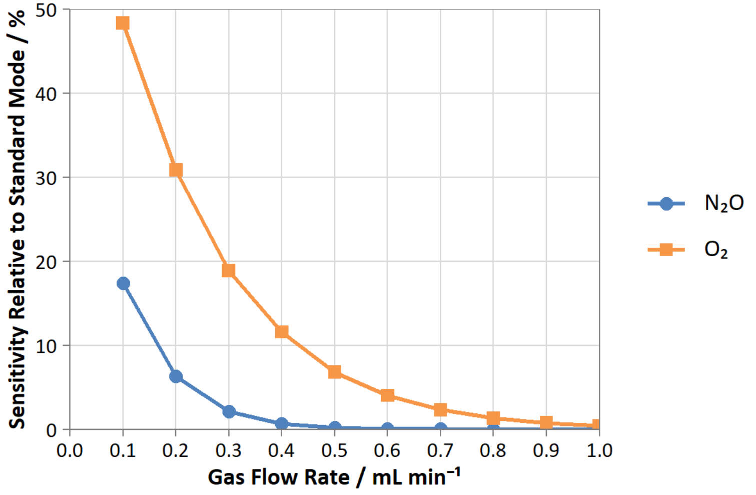

On-Mass Removal (as Interference) with N_2O

Variation of $^{89}Y^{16}O^+$ Interference with N_2O Flow Rate (On-Mass: Q1 = 105, Q3 = 105)

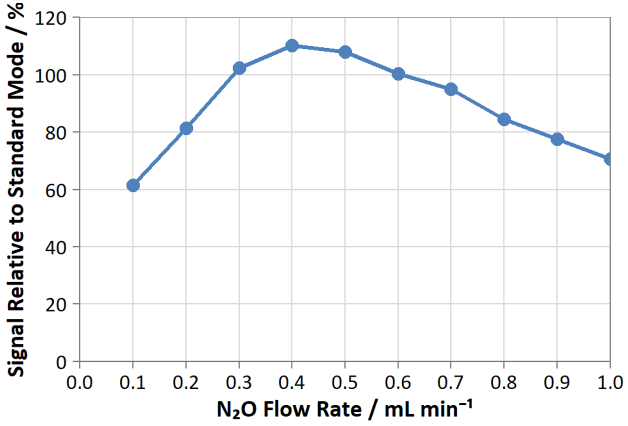

Variation of $^{89}Y^{2+}$ Interference with N_2O Flow Rate (On-Mass: Q1 = 44.5, Q3 = 44.5)

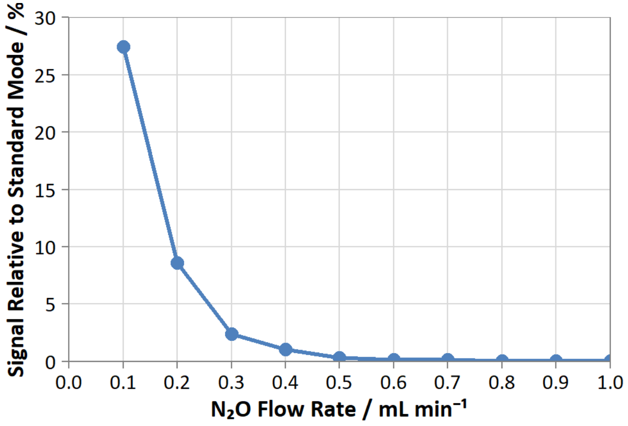

Asymmetric Charge Transfer (with N_2O)

[NO CHARGE TRANSFER REACTION OBSERVED]

Zirconium

Supplementary Information B to: Characterisation of gas cell reactions for 70+ elements using N_2O for ICP tandem mass spectrometry measurements
Shaun T. Lancaster[a]*, Thomas Prohaska[ab] and Johanna Irrgeher[ab]
[a]Department of General, Analytical and Physical Chemistry, Chair of General and Analytical Chemistry, Montanuniversität Leoben, Leoben, Austria.
[b]Department of Physics and Astronomy, University of Calgary, Calgary, Canada.

Mass-Shift with N_2O and O_2

Major Product Ions of $^{90}Zr^+$ with N_2O Reaction Gas

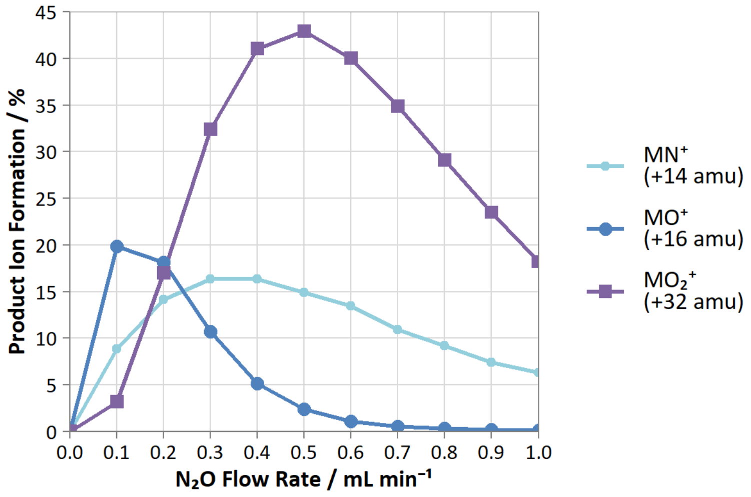

Major Product Ions of $^{90}Zr^+$ with O_2 Reaction Gas

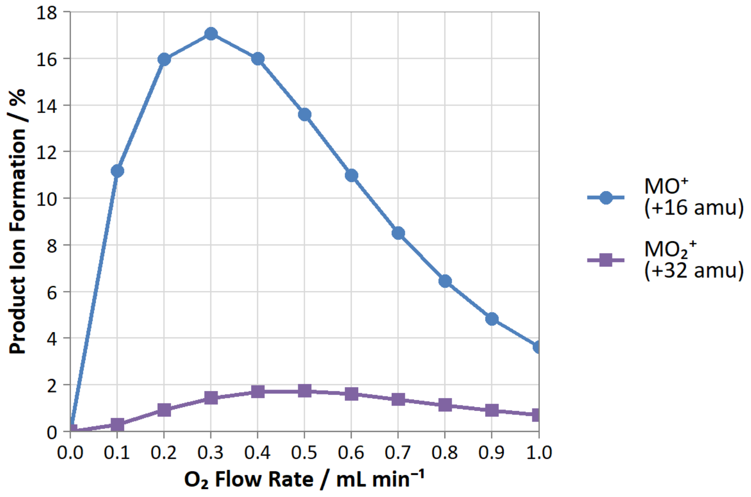

On-Mass with N_2O and O_2

On-Mass Profile of $^{90}Zr^+$ with N_2O and O_2

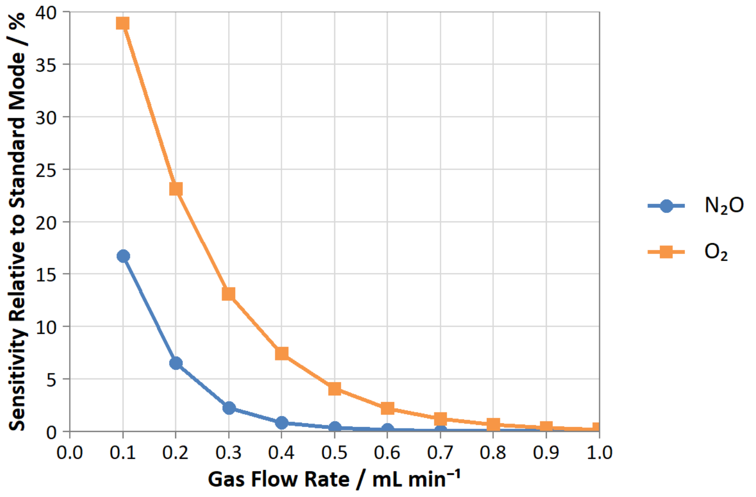

On-Mass Removal (as Interference) with N_2O

Variation of $^{90}Zr^{16}O^+$ Interference with N_2O Flow Rate (On-Mass: Q1 = 106, Q3 = 106)

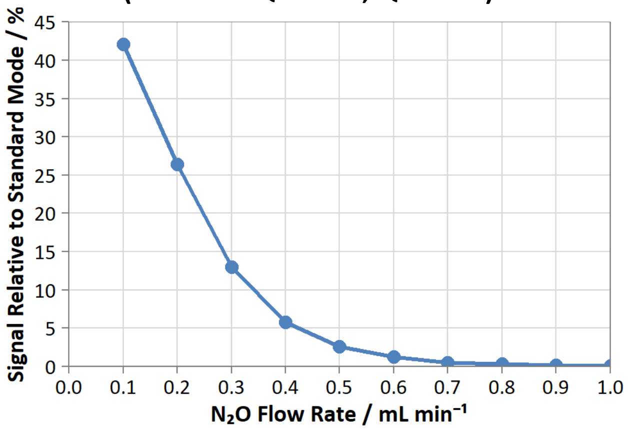

Variation of $^{90}Zr^{2+}$ Interference with N_2O Flow Rate (On-Mass: Q1 = 45, Q3 = 45)

[INSUFFICIENT DOUBLY-CHARGED ION FORMATION OBSERVED]

Asymmetric Charge Transfer (with N_2O)

[NO CHARGE TRANSFER REACTION OBSERVED]

Niobium

Shaun T. Lancaster[a]*, Thomas Prohaska[ab] and Johanna Irrgeher[ab]
[a]Department of General, Analytical and Physical Chemistry, Chair of General and Analytical Chemistry, Montanuniversität Leoben, Leoben, Austria.
[b]Department of Physics and Astronomy, University of Calgary, Calgary, Canada.

Mass-Shift with N_2O and O_2

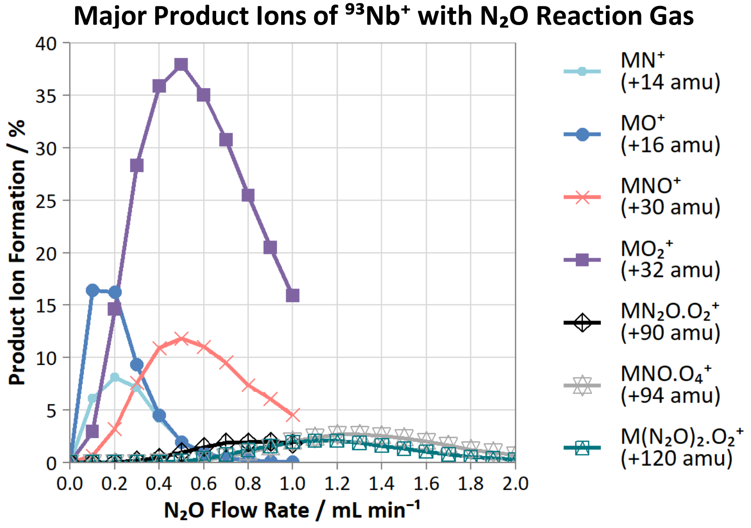

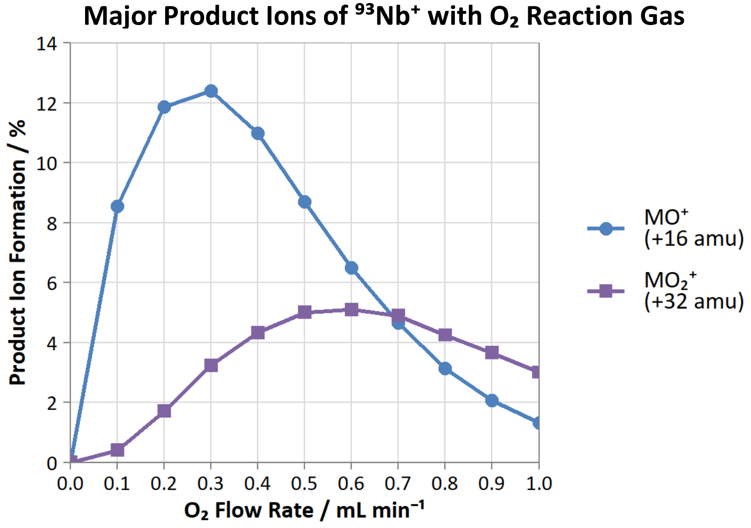

On-Mass with N_2O and O_2

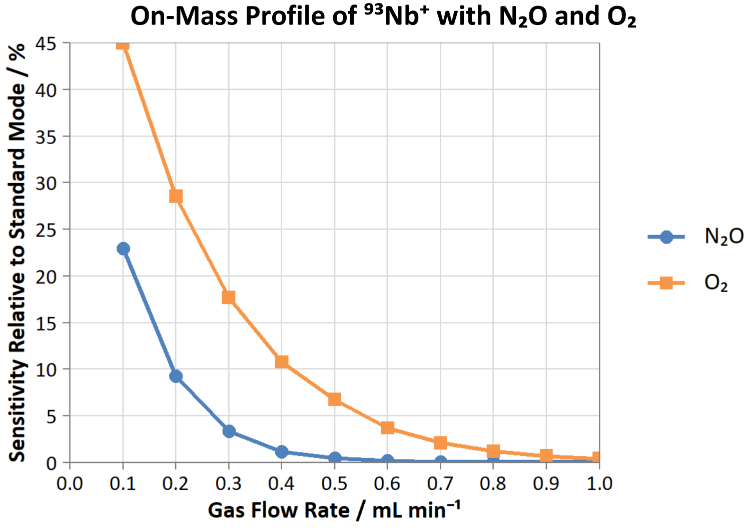

On-Mass Removal (as Interference) with N_2O

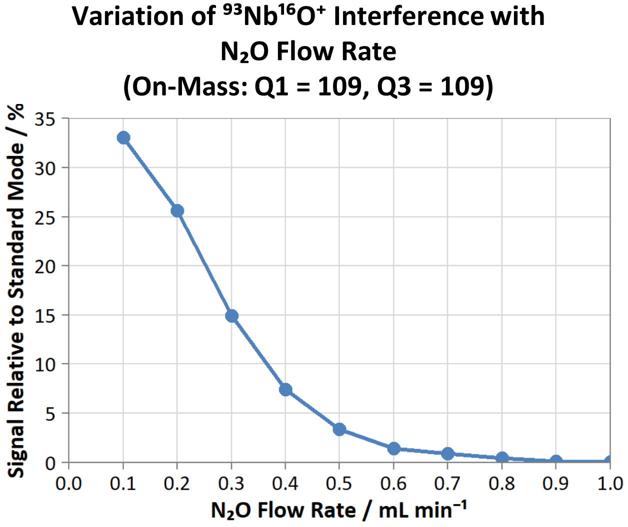

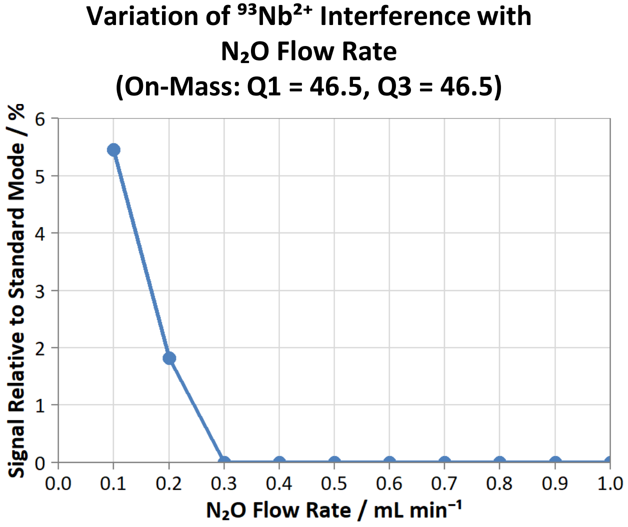

Asymmetric Charge Transfer (with N_2O)

[NO CHARGE TRANSFER REACTION OBSERVED]

Molybdenum

Supplementary Information B to: Characterisation of gas cell reactions for 70+ elements using N_2O for ICP tandem mass spectrometry measurements
Shaun T. Lancaster[a]*, Thomas Prohaska[ab] and Johanna Irrgeher[ab]
[a]Department of General, Analytical and Physical Chemistry, Chair of General and Analytical Chemistry, Montanuniversität Leoben, Leoben, Austria.
[b]Department of Physics and Astronomy, University of Calgary, Calgary, Canada.

Mass-Shift with N_2O and O_2

Major Product Ions of $^{98}Mo^+$ with N_2O Reaction Gas

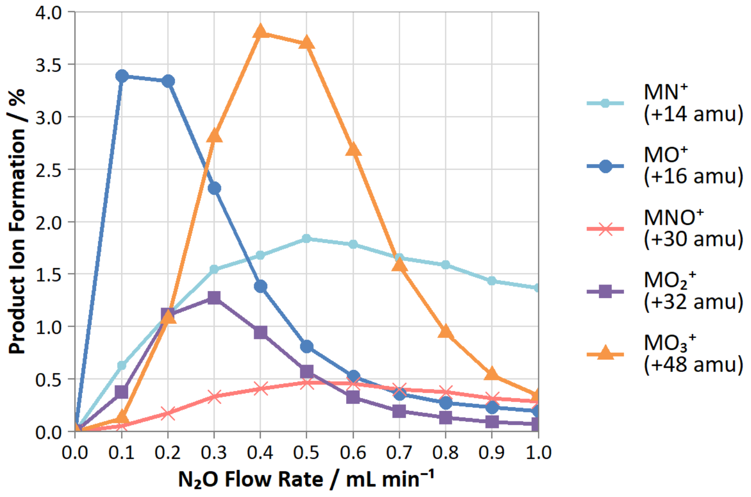

Major Product Ions of $^{98}Mo^+$ with O_2 Reaction Gas

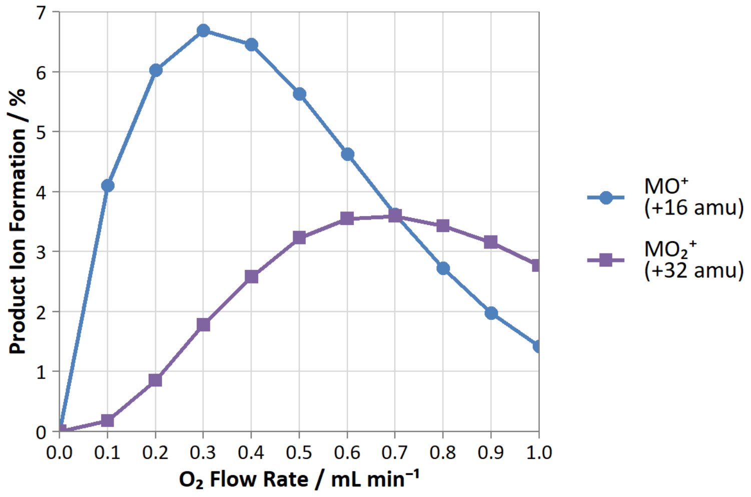

On-Mass with N_2O and O_2

On-Mass Profile of $^{98}Mo^+$ with N_2O and O_2

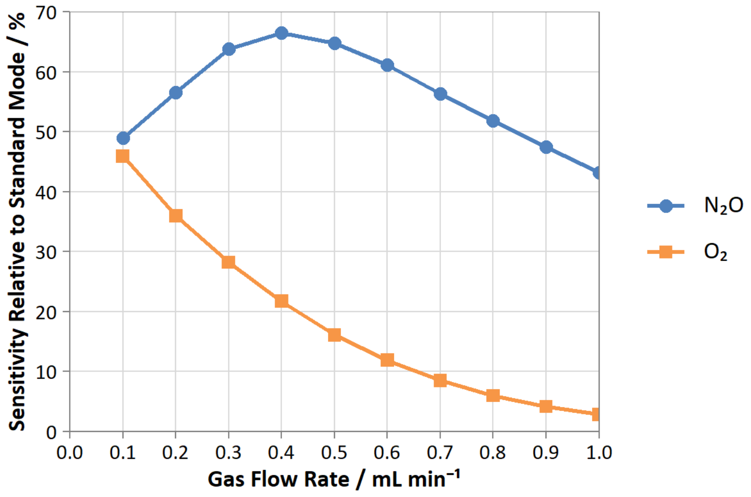

On-Mass Removal (as Interference) with N_2O

Variation of $^{98}Mo^{16}O^+$ Interference with N_2O Flow Rate (On-Mass: Q1 = 114, Q3 = 114)

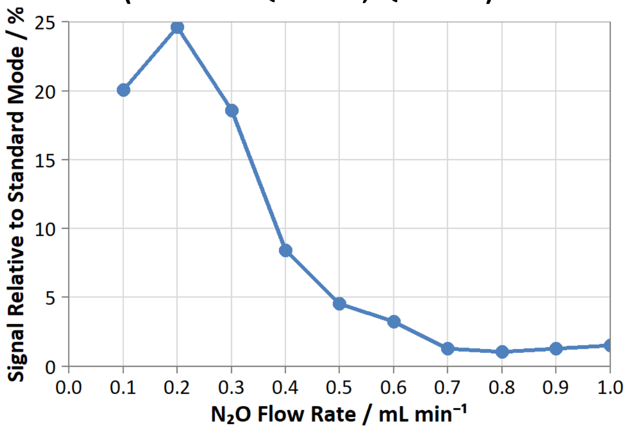

Variation of $^{98}Mo^{2+}$ Interference with N_2O Flow Rate (On-Mass: Q1 = 49, Q3 = 49)

[INSUFFICIENT DOUBLY-CHARGED ION FORMATION OBSERVED]

Asymmetric Charge Transfer (with N_2O)

[NO CHARGE TRANSFER REACTION OBSERVED]

Ruthenium

Supplementary Information B to: Characterisation of gas cell reactions for 70+ elements using N_2O for ICP tandem mass spectrometry measurements
Shaun T. Lancaster[a]*, Thomas Prohaska[ab] and Johanna Irrgeher[ab]
[a]Department of General, Analytical and Physical Chemistry, Chair of General and Analytical Chemistry, Montanuniversität Leoben, Leoben, Austria.
[b]Department of Physics and Astronomy, University of Calgary, Calgary, Canada.

Mass-Shift with N_2O and O_2

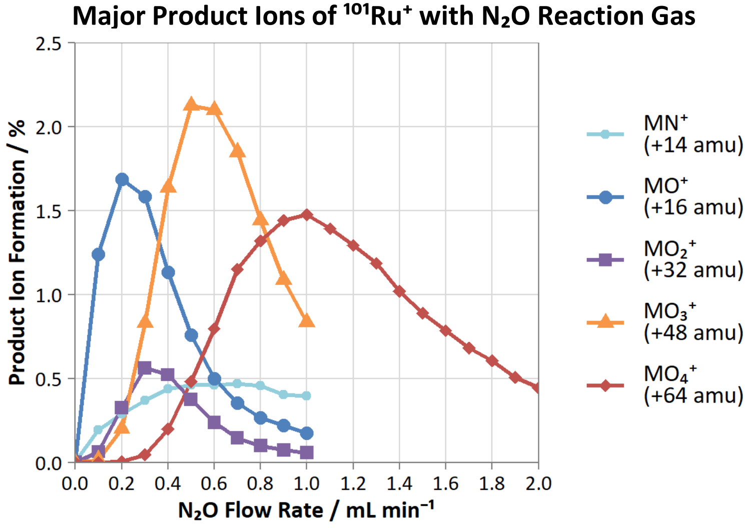

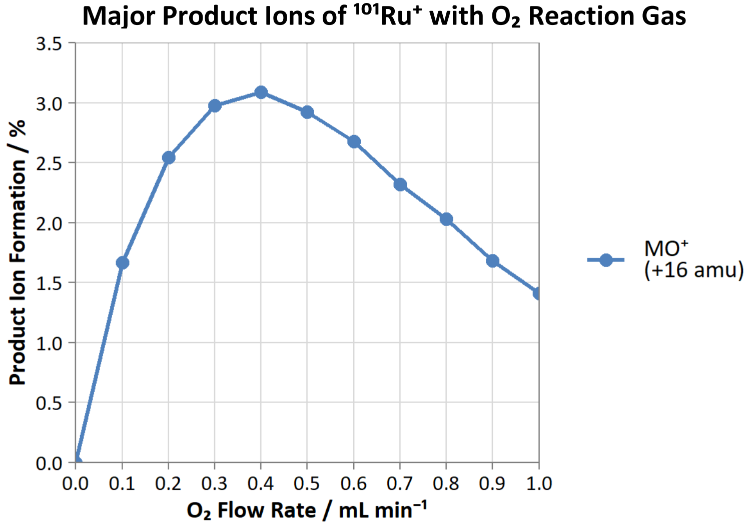

On-Mass with N_2O and O_2

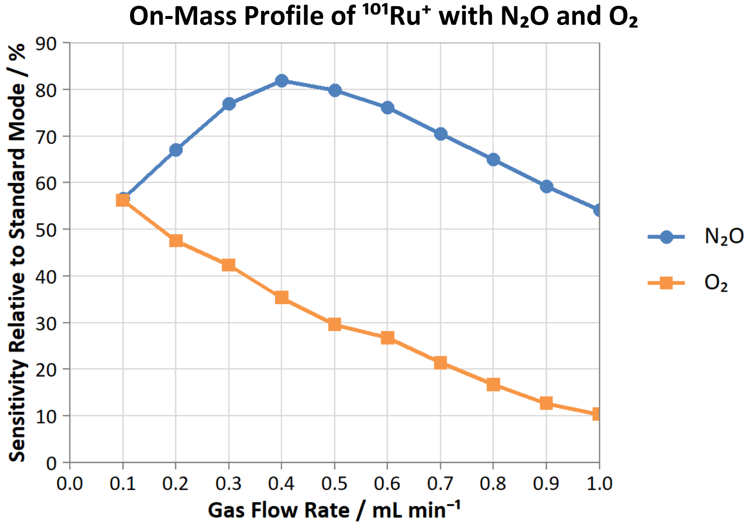

On-Mass Removal (as Interference) with N_2O

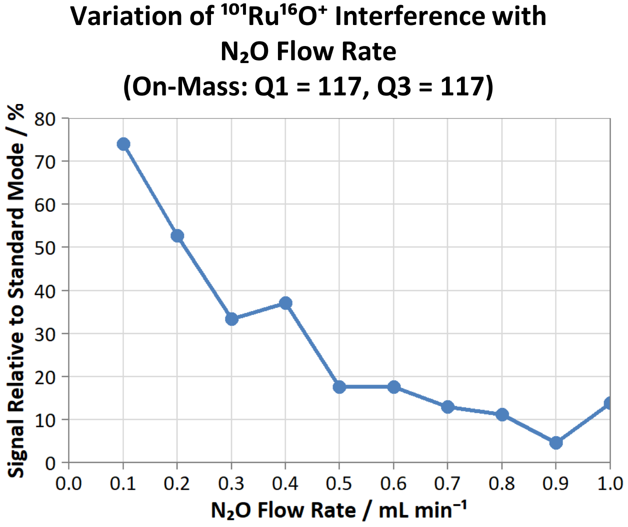

Variation of $^{101}Ru^{2+}$ Interference with N_2O Flow Rate (On-Mass: Q1 = 50.5, Q3 = 50.5)

[INSUFFICIENT DOUBLY-CHARGED ION FORMATION OBSERVED]

Asymmetric Charge Transfer (with N_2O)

[NO CHARGE TRANSFER REACTION OBSERVED]

Rhodium

Supplementary Information B to: Characterisation of gas cell reactions for 70+ elements using N_2O for ICP tandem mass spectrometry measurements
Shaun T. Lancaster[a]*, Thomas Prohaska[ab] and Johanna Irrgeher[ab]
[a]Department of General, Analytical and Physical Chemistry, Chair of General and Analytical Chemistry, Montanuniversität Leoben, Leoben, Austria.
[b]Department of Physics and Astronomy, University of Calgary, Calgary, Canada.

Mass-Shift with N_2O and O_2

Major Product Ions of $^{103}Rh^{+}$ with N_2O Reaction Gas

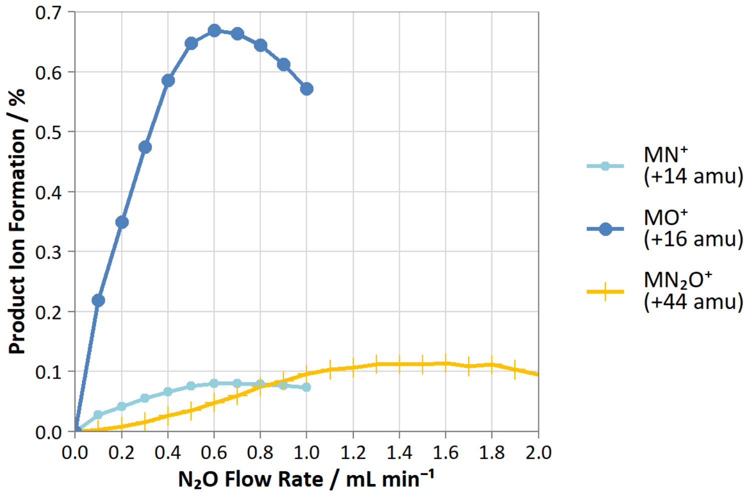

Major Product Ions of $^{103}Rh^{+}$ with O_2 Reaction Gas

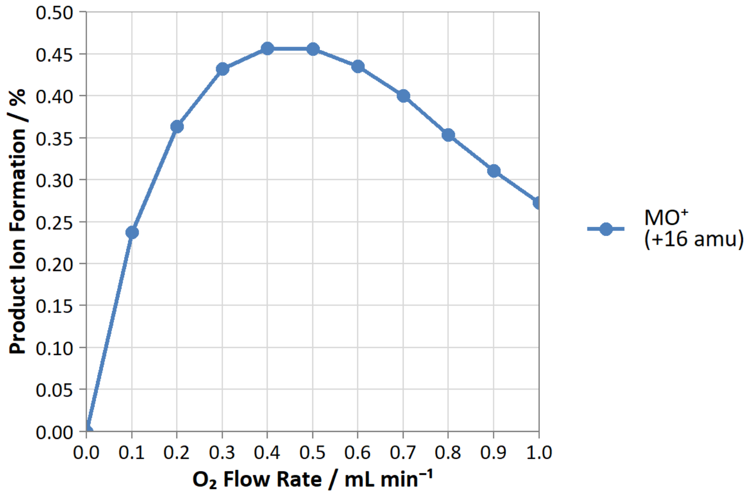

On-Mass with N_2O and O_2

On-Mass Profile of $^{103}Rh^{+}$ with N_2O and O_2

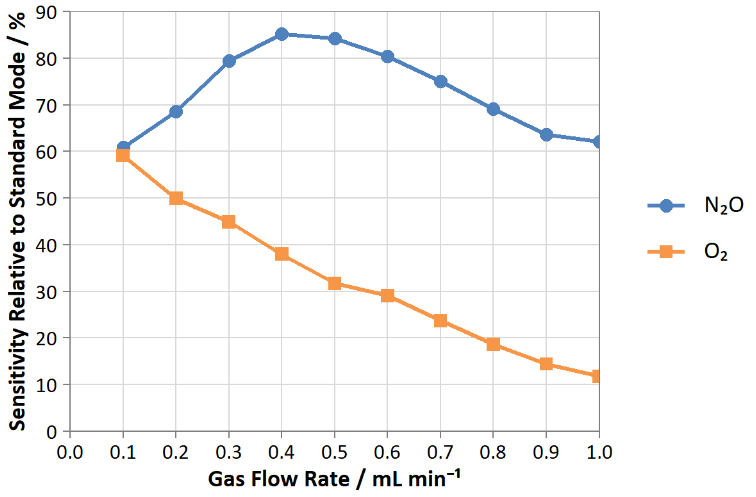

On-Mass Removal (as Interference) with N_2O

Variation of $^{103}Rh^{16}O^{+}$ Interference with N_2O Flow Rate (On-Mass: Q1 = 119, Q3 = 119)

[INSUFFICIENT OXIDE FORMATION OBSERVED]

Variation of $^{103}Rh^{2+}$ Interference with N_2O Flow Rate (On-Mass: Q1 = 51.5, Q3 = 51.5)

[INSUFFICIENT DOUBLY-CHARGED ION FORMATION OBSERVED]

Asymmetric Charge Transfer (with N_2O)

[NO CHARGE TRANSFER REACTION OBSERVED]

Palladium

Supplementary Information B to: Characterisation of gas cell reactions for 70+ elements using N_2O for ICP tandem mass spectrometry measurements
Shaun T. Lancaster[a]*, Thomas Prohaska[ab] and Johanna Irrgeher[ab]
[a]Department of General, Analytical and Physical Chemistry, Chair of General and Analytical Chemistry, Montanuniversität Leoben, Leoben, Austria.
[b]Department of Physics and Astronomy, University of Calgary, Calgary, Canada.

Mass-Shift with N_2O and O_2

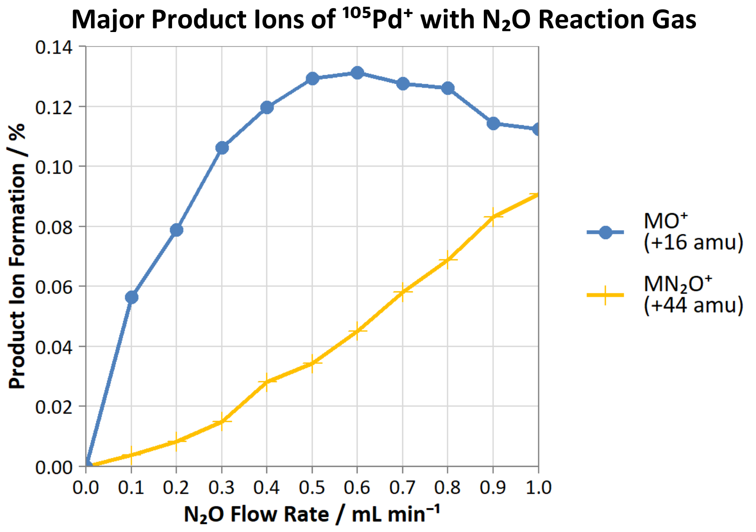

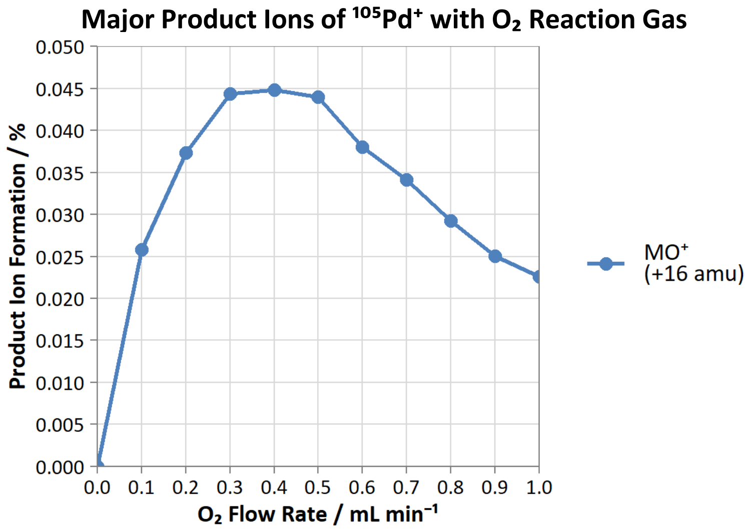

On-Mass with N_2O and O_2

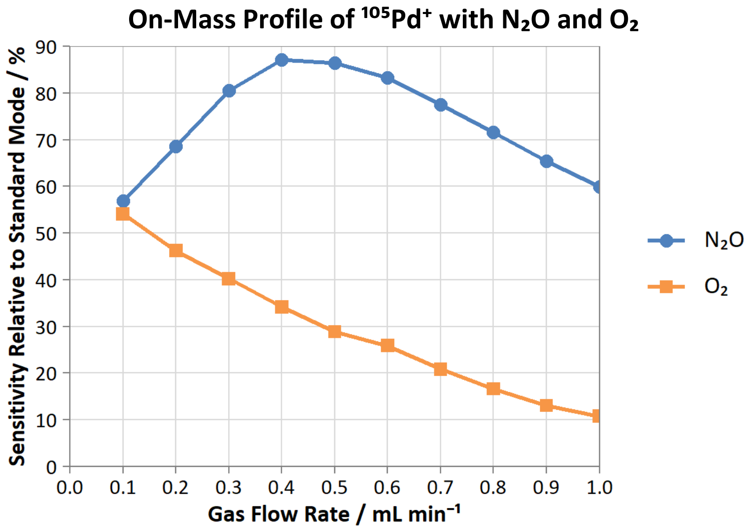

On-Mass Removal (as Interference) with N_2O

Variation of $^{105}Pd^{16}O^+$ Interference with N_2O Flow Rate (On-Mass: Q1 = 121, Q3 = 121)

[INSUFFICIENT OXIDE FORMATION OBSERVED]

Variation of $^{105}Pd^{2+}$ Interference with N_2O Flow Rate (On-Mass: Q1 = 52.5, Q3 = 52.5)

[INSUFFICIENT DOUBLY-CHARGED ION FORMATION OBSERVED]

Asymmetric Charge Transfer (with N_2O)

[NO CHARGE TRANSFER REACTION OBSERVED]

Silver

Supplementary Information B to: Characterisation of gas cell reactions for 70+ elements using N_2O for ICP tandem mass spectrometry measurements
Shaun T. Lancaster[a]*, Thomas Prohaska[ab] and Johanna Irrgeher[ab]
[a]Department of General, Analytical and Physical Chemistry, Chair of General and Analytical Chemistry, Montanuniversität Leoben, Leoben, Austria.
[b]Department of Physics and Astronomy, University of Calgary, Calgary, Canada.

Mass-Shift with N_2O and O_2

Major Product Ions of $^{107}Ag^+$ with N_2O Reaction Gas

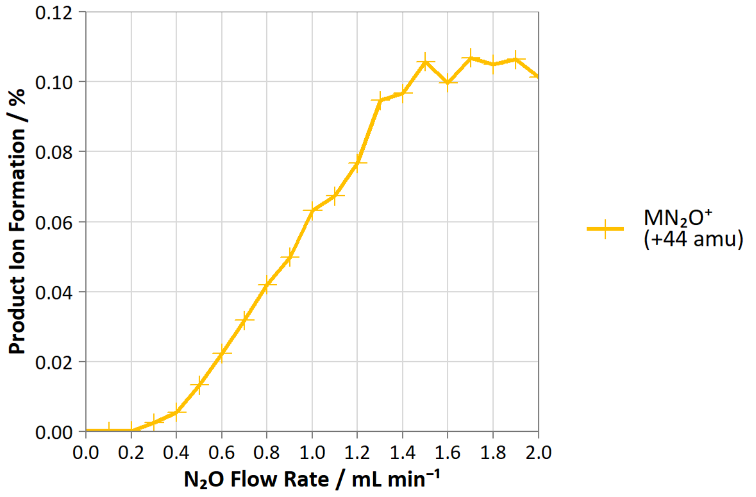

Major Product Ions of $^{107}Ag^+$ with O_2 Reaction Gas

[NO ATOM TRANSFER REACTION OBSERVED]

On-Mass with N_2O and O_2

On-Mass Profile of $^{107}Ag^+$ with N_2O and O_2

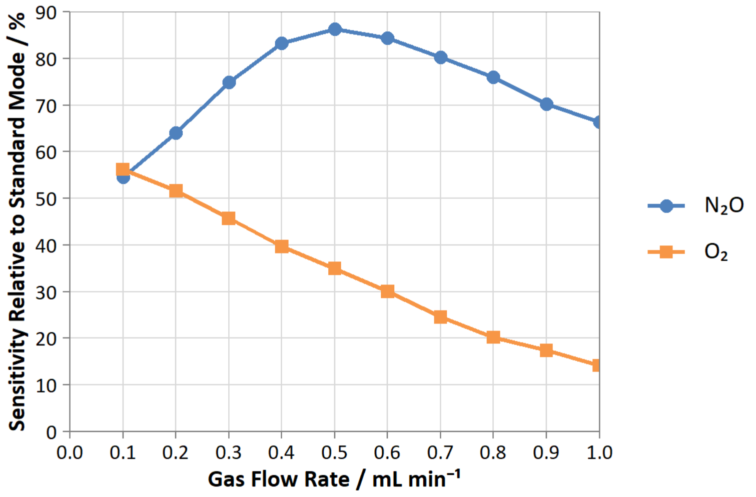

On-Mass Removal (as Interference) with N_2O

Variation of $^{107}Ag^{16}O^+$ Interference with N_2O Flow Rate (On-Mass: Q1 = 123, Q3 = 123)

[INSUFFICIENT OXIDE FORMATION OBSERVED]

Variation of $^{107}Ag^{2+}$ Interference with N_2O Flow Rate (On-Mass: Q1 = 53.5, Q3 = 53.5)

[INSUFFICIENT DOUBLY-CHARGED ION FORMATION OBSERVED]

Asymmetric Charge Transfer (with N_2O)

[NO CHARGE TRANSFER REACTION OBSERVED]

Cadmium

Supplementary Information B to: Characterisation of gas cell reactions for 70+ elements using N_2O for ICP tandem mass spectrometry measurements
Shaun T. Lancaster[a]*, Thomas Prohaska[ab] and Johanna Irrgeher[ab]
[a]Department of General, Analytical and Physical Chemistry, Chair of General and Analytical Chemistry, Montanuniversität Leoben, Leoben, Austria.
[b]Department of Physics and Astronomy, University of Calgary, Calgary, Canada.

Mass-Shift with N_2O and O_2

Major Product Ions of $^{114}Cd^+$ with N_2O Reaction Gas

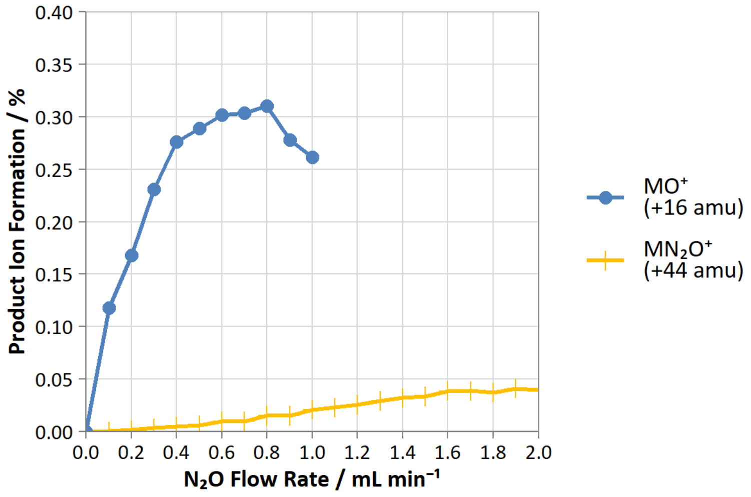

Major Product Ions of $^{114}Cd^+$ with O_2 Reaction Gas

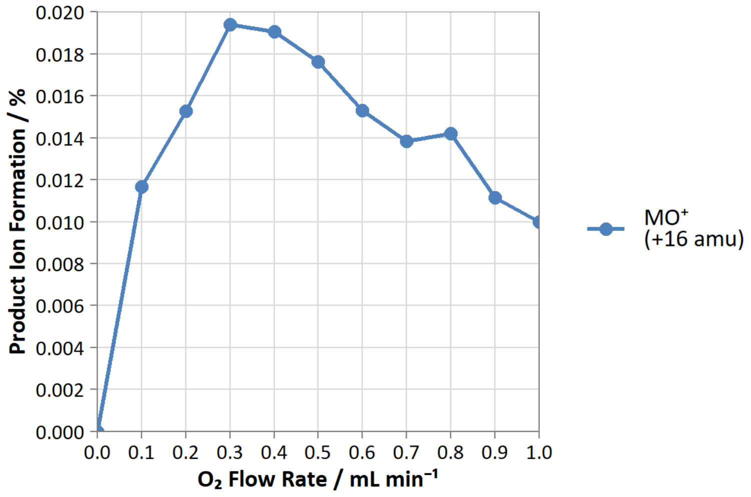

On-Mass with N_2O and O_2

On-Mass Profile of $^{114}Cd^+$ with N_2O and O_2

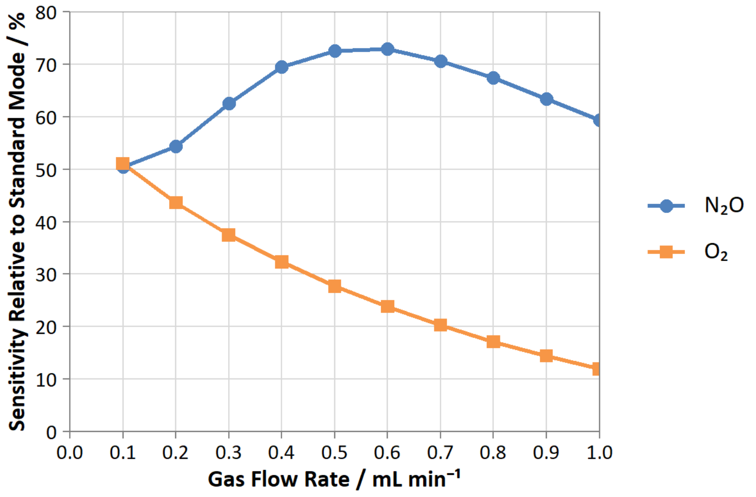

On-Mass Removal (as Interference) with N_2O

Variation of $^{114}Cd^{16}O^+$ Interference with N_2O Flow Rate (On-Mass: Q1 = 130, Q3 = 130)

[INSUFFICIENT OXIDE FORMATION OBSERVED]

Variation of $^{114}Cd^{2+}$ Interference with N_2O Flow Rate (On-Mass: Q1 = 57, Q3 = 57)

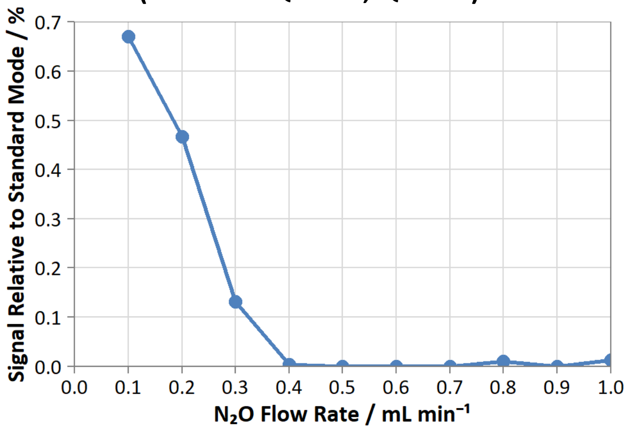

Asymmetric Charge Transfer (with N_2O)

[NO CHARGE TRANSFER REACTION OBSERVED]

Indium

Supplementary Information B to: Characterisation of gas cell reactions for 70+ elements using N_2O for ICP tandem mass spectrometry measurements
Shaun T. Lancaster[a*], Thomas Prohaska[ab] and Johanna Irrgeher[ab]
[a]Department of General, Analytical and Physical Chemistry, Chair of General and Analytical Chemistry, Montanuniversität Leoben, Leoben, Austria.
[b]Department of Physics and Astronomy, University of Calgary, Calgary, Canada.

Mass-Shift with N_2O and O_2

Major Product Ions of $^{115}In^+$ with N_2O Reaction Gas

[NO ATOM TRANSFER REACTION OBSERVED]

Major Product Ions of $^{115}In^+$ with O_2 Reaction Gas

[NO ATOM TRANSFER REACTION OBSERVED]

On-Mass with N_2O and O_2

On-Mass Profile of $^{115}In^+$ with N_2O and O_2

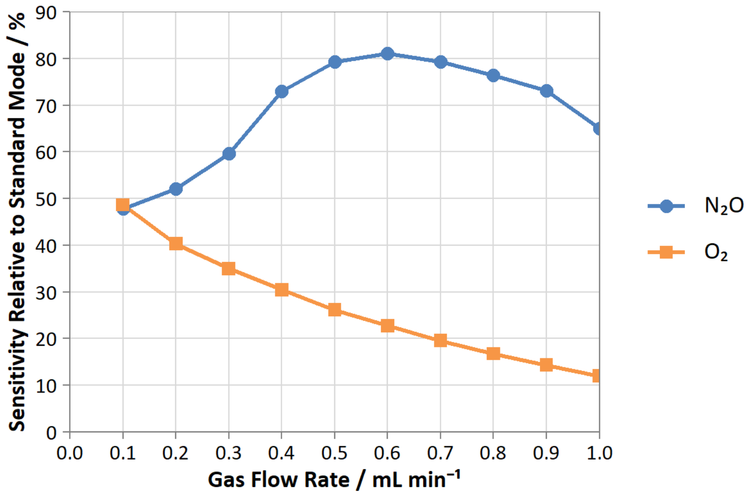

On-Mass Removal (as Interference) with N_2O

Variation of $^{115}In^{16}O^+$ Interference with N_2O Flow Rate (On-Mass: Q1 = 131, Q3 = 131)

[INSUFFICIENT OXIDE FORMATION OBSERVED]

Variation of $^{115}In^{2+}$ Interference with N_2O Flow Rate (On-Mass: Q1 = 57.5, Q3 = 57.5)

[INSUFFICIENT DOUBLY-CHARGED ION FORMATION OBSERVED]

Asymmetric Charge Transfer (with N_2O)

[NO CHARGE TRANSFER REACTION OBSERVED]

Tin

Supplementary Information B to: Characterisation of gas cell reactions for 70+ elements using N_2O for ICP tandem mass spectrometry measurements
Shaun T. Lancaster[a]*, Thomas Prohaska[ab] and Johanna Irrgeher[ab]
[a]Department of General, Analytical and Physical Chemistry, Chair of General and Analytical Chemistry, Montanuniversität Leoben, Leoben, Austria.
[b]Department of Physics and Astronomy, University of Calgary, Calgary, Canada.

Mass-Shift with N_2O and O_2

Major Product Ions of $^{118}Sn^{+}$ with N_2O Reaction Gas

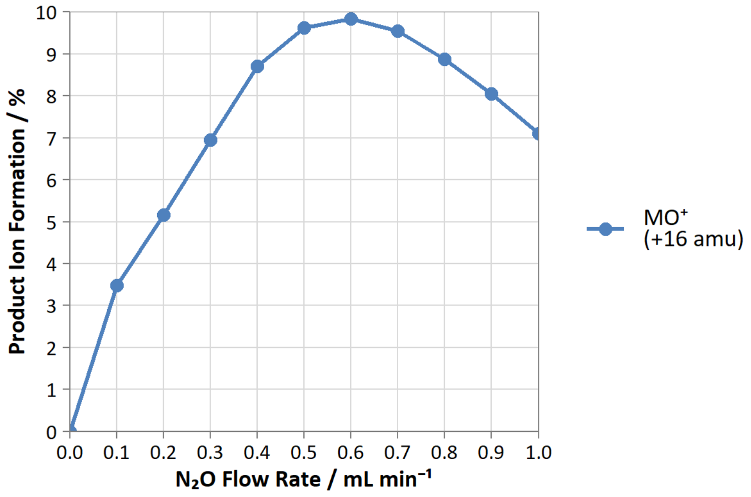

Major Product Ions of $^{118}Sn^{+}$ with O_2 Reaction Gas

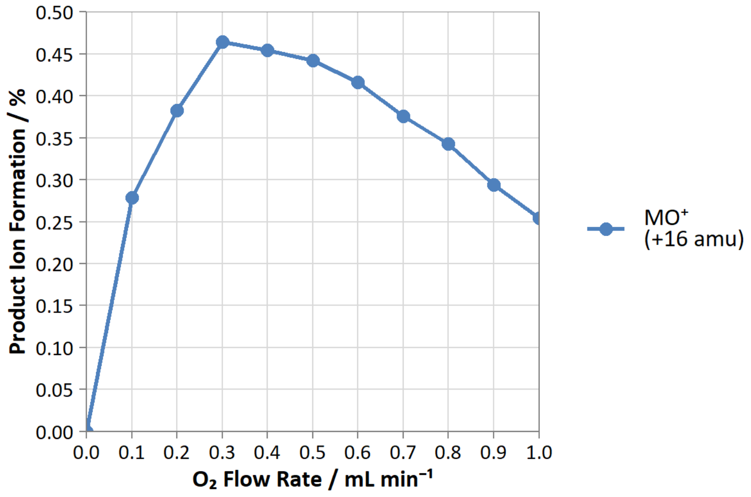

On-Mass with N_2O and O_2

On-Mass Profile of $^{118}Sn^{+}$ with N_2O and O_2

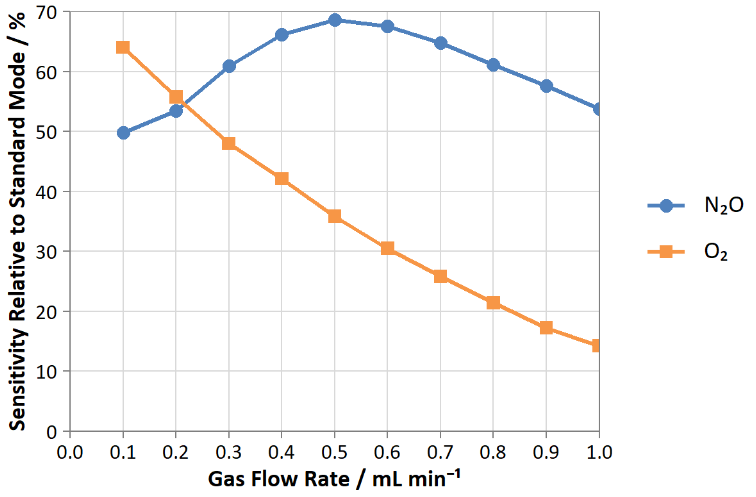

On-Mass Removal (as Interference) with N_2O

Variation of $^{118}Sn^{16}O^{+}$ Interference with N_2O Flow Rate (On-Mass: Q1 = 134, Q3 = 134)

[INSUFFICIENT OXIDE FORMATION OBSERVED]

Variation of $^{118}Sn^{2+}$ Interference with N_2O Flow Rate (On-Mass: Q1 = 59, Q3 = 59)

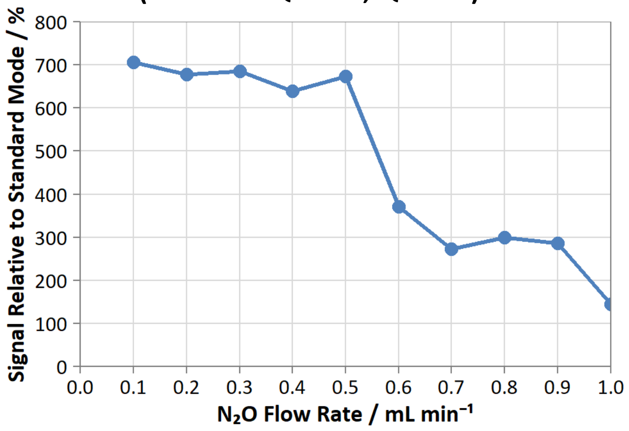

Asymmetric Charge Transfer (with N_2O)

[NO CHARGE TRANSFER REACTION OBSERVED]

Antimony

Supplementary Information B to: Characterisation of gas cell reactions for 70+ elements using N_2O for ICP tandem mass spectrometry measurements
Shaun T. Lancaster[a]*, Thomas Prohaska[ab] and Johanna Irrgeher[ab]
[a]Department of General, Analytical and Physical Chemistry, Chair of General and Analytical Chemistry, Montanuniversität Leoben, Leoben, Austria.
[b]Department of Physics and Astronomy, University of Calgary, Calgary, Canada.

Mass-Shift with N_2O and O_2

Major Product Ions of $^{121}Sb^+$ with N_2O Reaction Gas

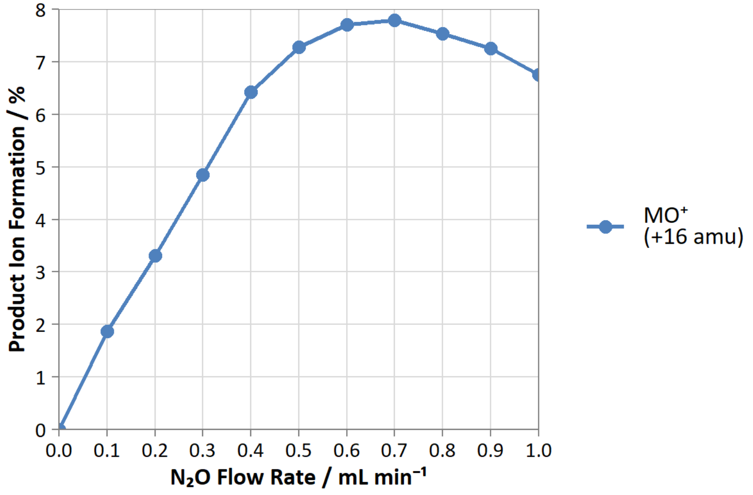

Major Product Ions of $^{121}Sb^+$ with O_2 Reaction Gas

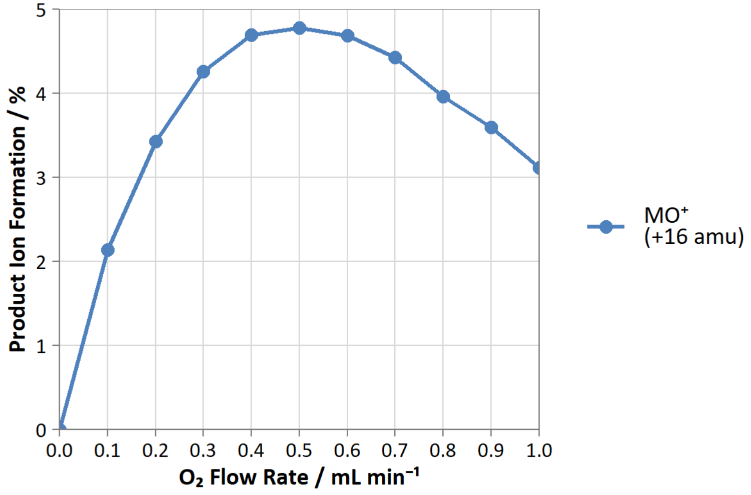

On-Mass with N_2O and O_2

On-Mass Profile of $^{121}Sb^+$ with N_2O and O_2

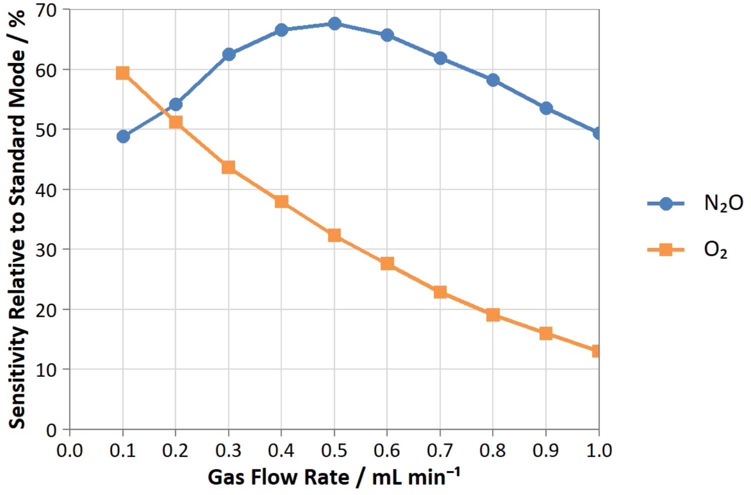

On-Mass Removal (as Interference) with N_2O

Variation of $^{121}Sb^{16}O^+$ Interference with N_2O Flow Rate (On-Mass: Q1 = 137, Q3 = 137)

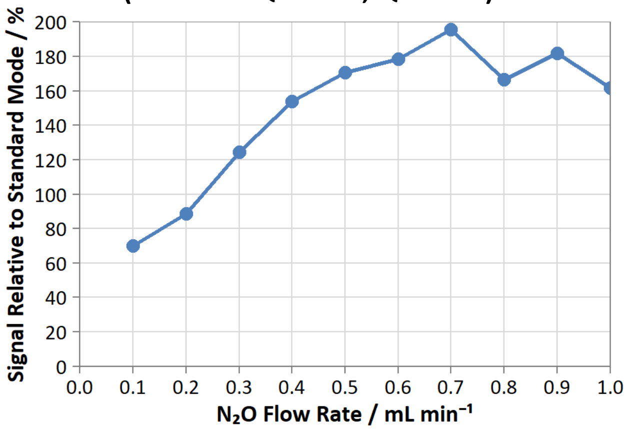

Variation of $^{121}Sb^{2+}$ Interference with N_2O Flow Rate (On-Mass: Q1 = 60.5, Q3 = 60.5)

[INSUFFICIENT DOUBLY-CHARGED ION FORMATION OBSERVED]

Asymmetric Charge Transfer (with N_2O)

[NO CHARGE TRANSFER REACTION OBSERVED]

Tellurium

Supplementary Information B to: Characterisation of gas cell reactions for 70+ elements using N_2O for ICP tandem mass spectrometry measurements
Shaun T. Lancaster[a]*, Thomas Prohaska[ab] and Johanna Irrgeher[ab]
[a]Department of General, Analytical and Physical Chemistry, Chair of General and Analytical Chemistry, Montanuniversität Leoben, Leoben, Austria.
[b]Department of Physics and Astronomy, University of Calgary, Calgary, Canada.

Mass-Shift with N_2O and O_2

Major Product Ions of $^{128}Te^+$ with N_2O Reaction Gas

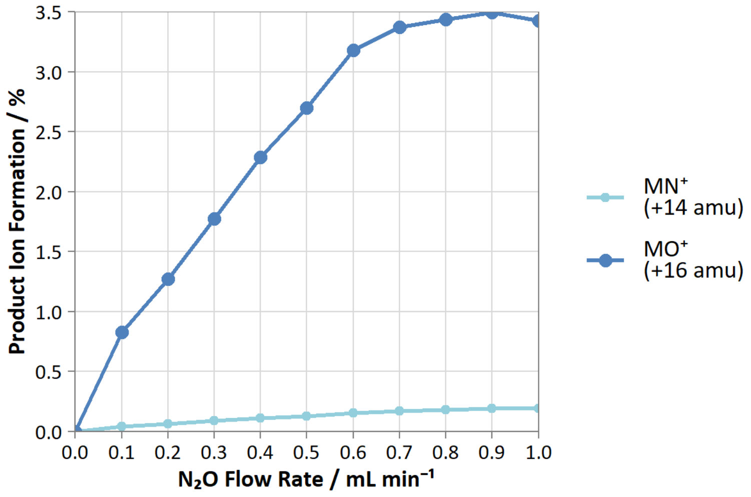

Major Product Ions of $^{128}Te^+$ with O_2 Reaction Gas

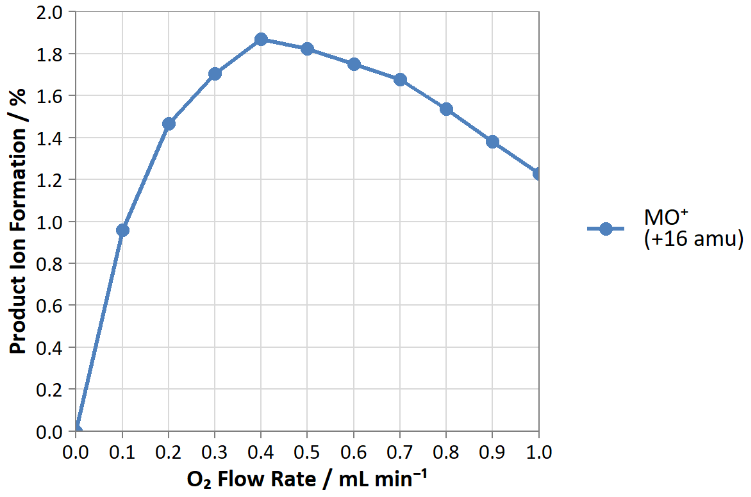

On-Mass with N_2O and O_2

On-Mass Profile of $^{128}Te^+$ with N_2O and O_2

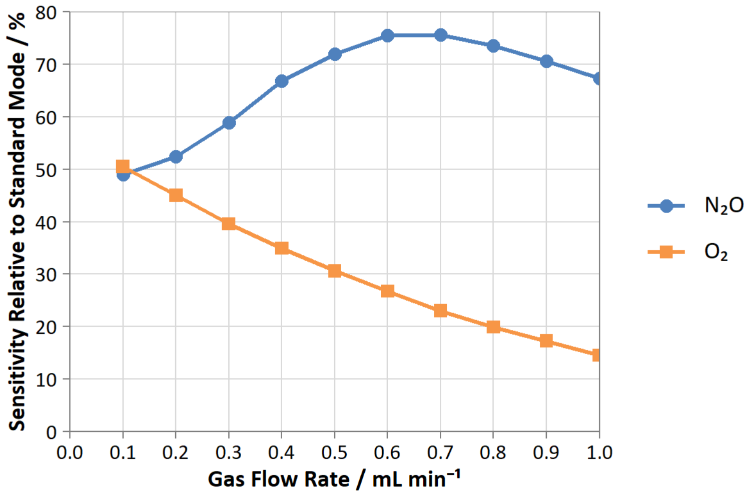

On-Mass Removal (as Interference) with N_2O

Variation of $^{128}Te^{16}O^+$ Interference with N_2O Flow Rate (On-Mass: Q1 = 144, Q3 = 144)

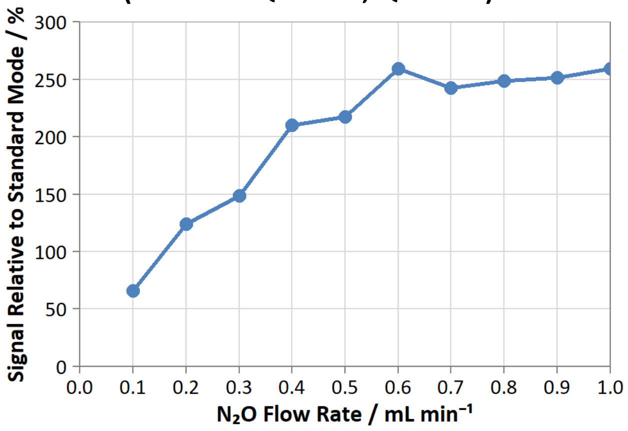

Variation of $^{128}Te^{2+}$ Interference with N_2O Flow Rate (On-Mass: Q1 = 64, Q3 = 64)

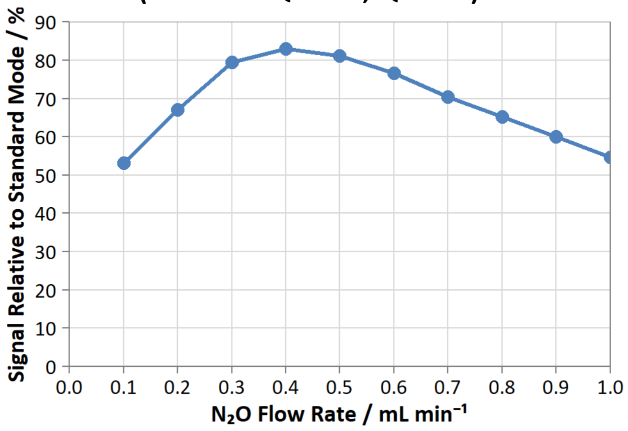

Asymmetric Charge Transfer (with N_2O)

[NO CHARGE TRANSFER REACTION OBSERVED]

Iodine

Supplementary Information B to: Characterisation of gas cell reactions for 70+ elements using N_2O for ICP tandem mass spectrometry measurements
Shaun T. Lancaster[a]*, Thomas Prohaska[ab] and Johanna Irrgeher[ab]
[a]Department of General, Analytical and Physical Chemistry, Chair of General and Analytical Chemistry, Montanuniversität Leoben, Leoben, Austria.
[b]Department of Physics and Astronomy, University of Calgary, Calgary, Canada.

Mass-Shift with N_2O and O_2

Major Product Ions of $^{127}I^+$ with N_2O Reaction Gas

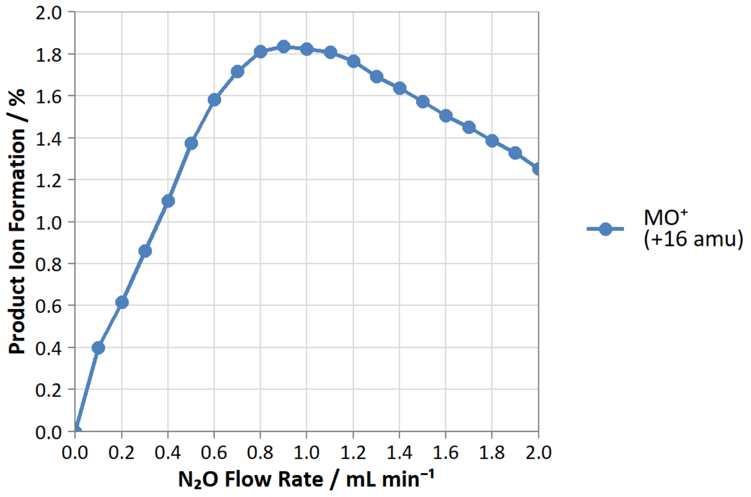

Major Product Ions of $^{127}I^+$ with O_2 Reaction Gas

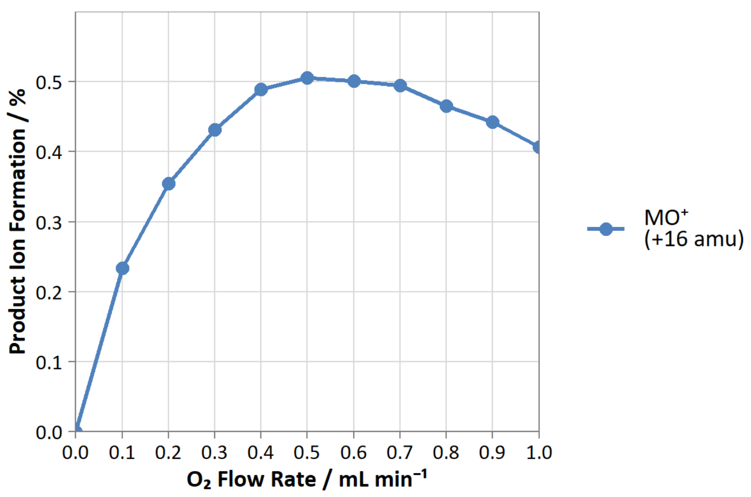

On-Mass with N_2O and O_2

On-Mass Profile of $^{127}I^+$ with N_2O and O_2

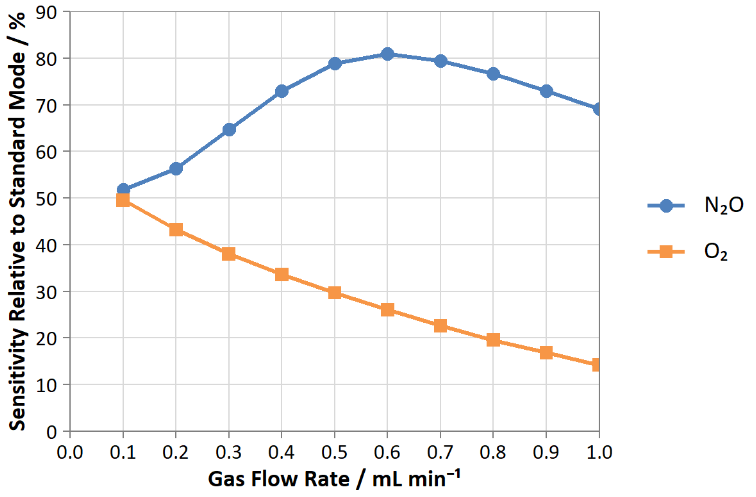

On-Mass Removal (as Interference) with N_2O

Variation of $^{127}I^{16}O^+$ Interference with N_2O Flow Rate (On-Mass: Q1 = 143, Q3 = 143)

[INSUFFICIENT OXIDE FORMATION OBSERVED]

Variation of $^{127}I^{2+}$ Interference with N_2O Flow Rate (On-Mass: Q1 = 63.5, Q3 = 63.5)

[INSUFFICIENT DOUBLY-CHARGED ION FORMATION OBSERVED]

Asymmetric Charge Transfer (with N_2O)

[NO CHARGE TRANSFER REACTION OBSERVED]

Cesium

Supplementary Information B to: Characterisation of gas cell reactions for 70+ elements using N_2O for ICP tandem mass spectrometry measurements
Shaun T. Lancaster[a]*, Thomas Prohaska[ab] and Johanna Irrgeher[ab]
[a]Department of General, Analytical and Physical Chemistry, Chair of General and Analytical Chemistry, Montanuniversität Leoben, Leoben, Austria.
[b]Department of Physics and Astronomy, University of Calgary, Calgary, Canada.

Mass-Shift with N_2O and O_2

Major Product Ions of $^{133}Cs^+$ with N_2O Reaction Gas

[NO ATOM TRANSFER REACTION OBSERVED]

Major Product Ions of $^{133}Cs^+$ with O_2 Reaction Gas

[NO ATOM TRANSFER REACTION OBSERVED]

On-Mass with N_2O and O_2

On-Mass Profile of $^{133}Cs^+$ with N_2O and O_2

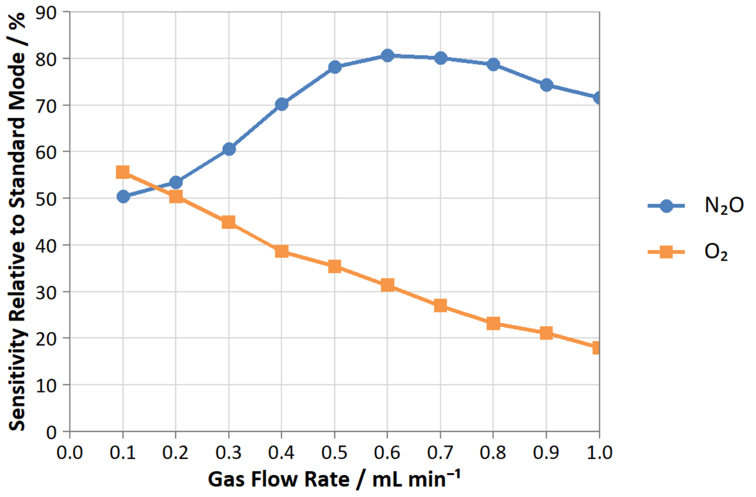

On-Mass Removal (as Interference) with N_2O

Variation of $^{133}Cs^{16}O^+$ Interference with N_2O Flow Rate (On-Mass: Q1 = 149, Q3 = 149)

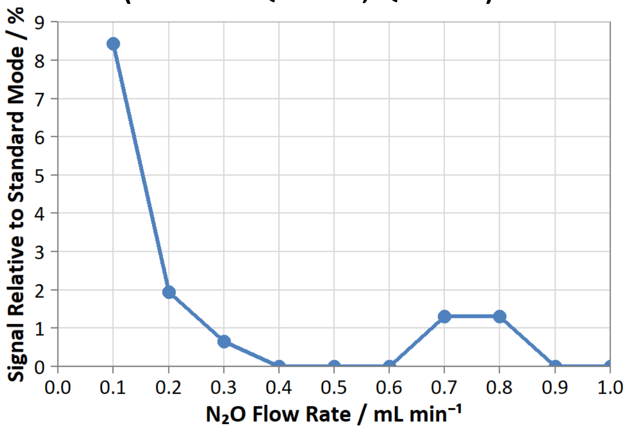

Variation of $^{133}Cs^{2+}$ Interference with N_2O Flow Rate (On-Mass: Q1 = 66.5, Q3 = 66.5)

[INSUFFICIENT DOUBLY-CHARGED ION FORMATION OBSERVED]

Asymmetric Charge Transfer (with N_2O)

[NO CHARGE TRANSFER REACTION OBSERVED]

Barium

Supplementary Information B to: Characterisation of gas cell reactions for 70+ elements using N_2O for ICP tandem mass spectrometry measurements
Shaun T. Lancaster[a]*, Thomas Prohaska[ab] and Johanna Irrgeher[ab]
[a]Department of General, Analytical and Physical Chemistry, Chair of General and Analytical Chemistry, Montanuniversität Leoben, Leoben, Austria.
[b]Department of Physics and Astronomy, University of Calgary, Calgary, Canada.

Mass-Shift with N_2O and O_2

Major Product Ions of $^{138}Ba^+$ with N_2O Reaction Gas

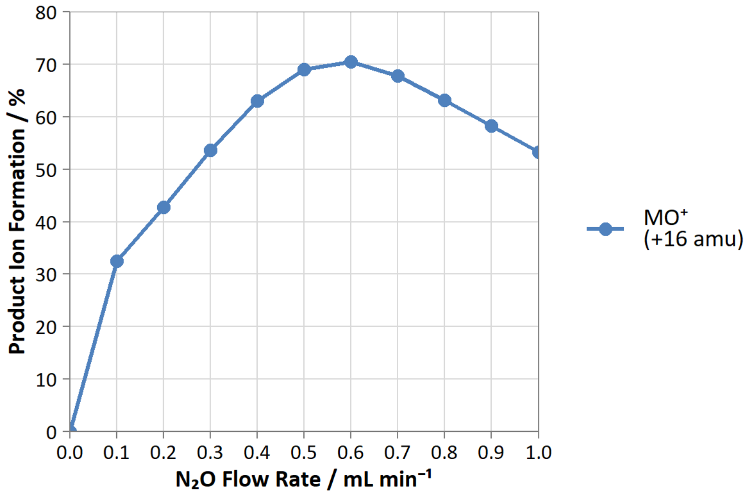

Major Product Ions of $^{138}Ba^+$ with O_2 Reaction Gas

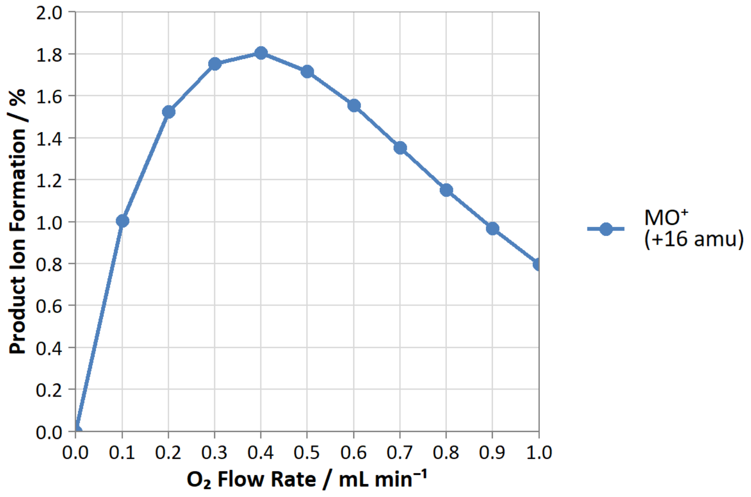

On-Mass with N_2O and O_2

On-Mass Profile of $^{138}Ba^+$ with N_2O and O_2

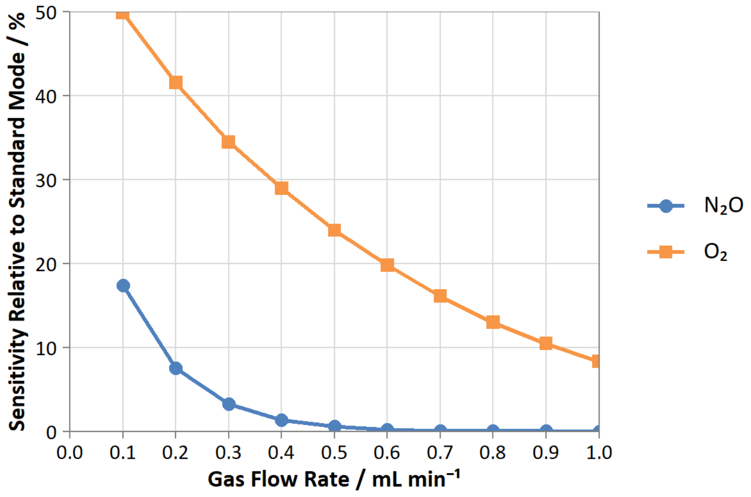

On-Mass Removal (as Interference) with N_2O

Variation of $^{138}Ba^{16}O^+$ Interference with N_2O Flow Rate (On-Mass: Q1 = 154, Q3 = 154)

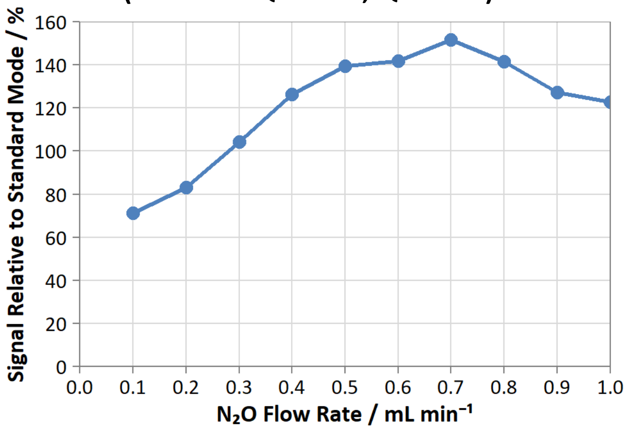

Variation of $^{138}Ba^{2+}$ Interference with N_2O Flow Rate (On-Mass: Q1 = 69, Q3 = 69)

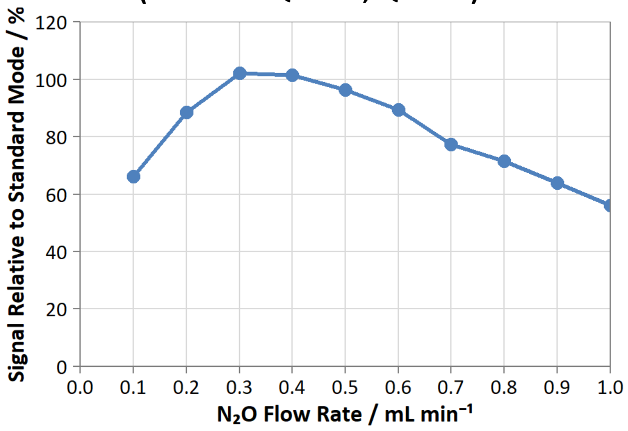

Asymmetric Charge Transfer (with N_2O)

[NO CHARGE TRANSFER REACTION OBSERVED]

Lanthanum

Supplementary Information B to: Characterisation of gas cell reactions for 70+ elements using N_2O for ICP tandem mass spectrometry measurements
Shaun T. Lancaster[a]*, Thomas Prohaska[ab] and Johanna Irrgeher[ab]
[a]Department of General, Analytical and Physical Chemistry, Chair of General and Analytical Chemistry, Montanuniversität Leoben, Leoben, Austria.
[b]Department of Physics and Astronomy, University of Calgary, Calgary, Canada.

Mass-Shift with N_2O and O_2

Major Product Ions of $^{139}La^+$ with N_2O Reaction Gas

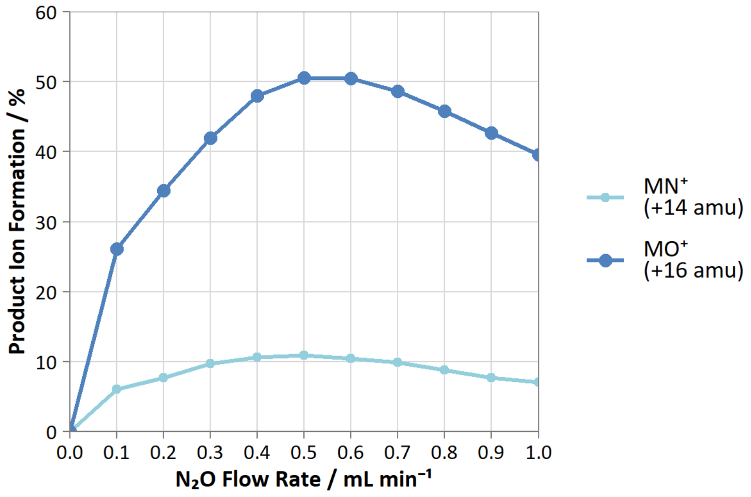

Major Product Ions of $^{139}La^+$ with O_2 Reaction Gas

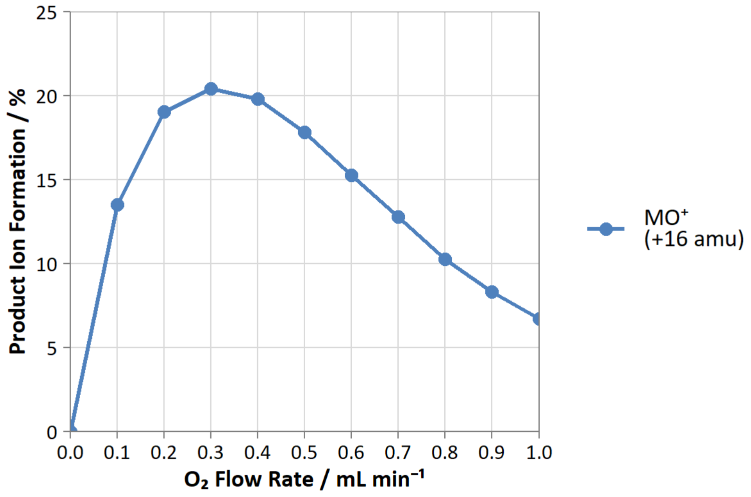

On-Mass with N_2O and O_2

On-Mass Profile of $^{139}La^+$ with N_2O and O_2

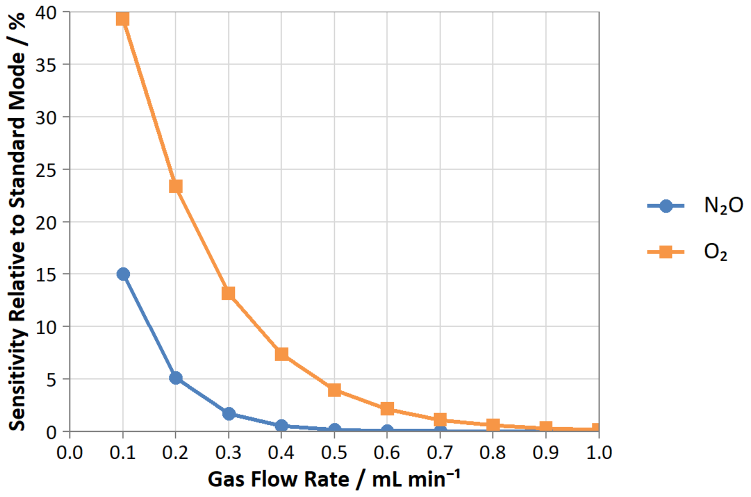

On-Mass Removal (as Interference) with N_2O

Variation of $^{139}La^{16}O^+$ Interference with N_2O Flow Rate (On-Mass: Q1 = 155, Q3 = 155)

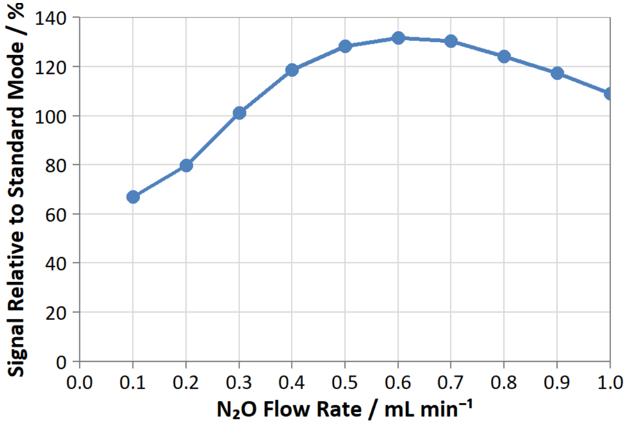

Variation of $^{139}La^{2+}$ Interference with N_2O Flow Rate (On-Mass: Q1 = 69.5, Q3 = 69.5)

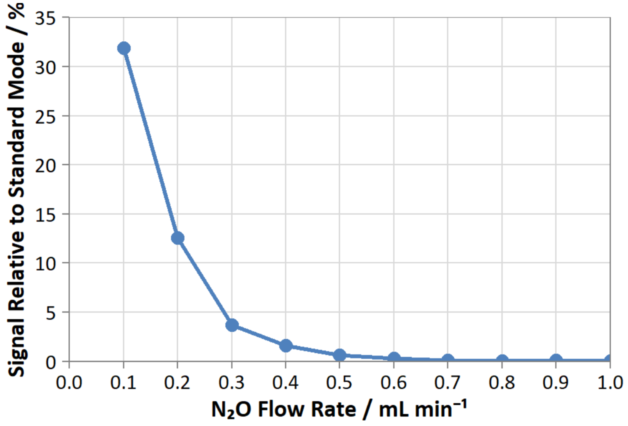

Asymmetric Charge Transfer (with N_2O)

[NO CHARGE TRANSFER REACTION OBSERVED]

Cerium

Supplementary Information B to: Characterisation of gas cell reactions for 70+ elements using N_2O for ICP tandem mass spectrometry measurements
Shaun T. Lancaster[a]*, Thomas Prohaska[ab] and Johanna Irrgeher[ab]
[a]Department of General, Analytical and Physical Chemistry, Chair of General and Analytical Chemistry, Montanuniversität Leoben, Leoben, Austria.
[b]Department of Physics and Astronomy, University of Calgary, Calgary, Canada.

Mass-Shift with N_2O and O_2

Major Product Ions of $^{140}Ce^+$ with N_2O Reaction Gas

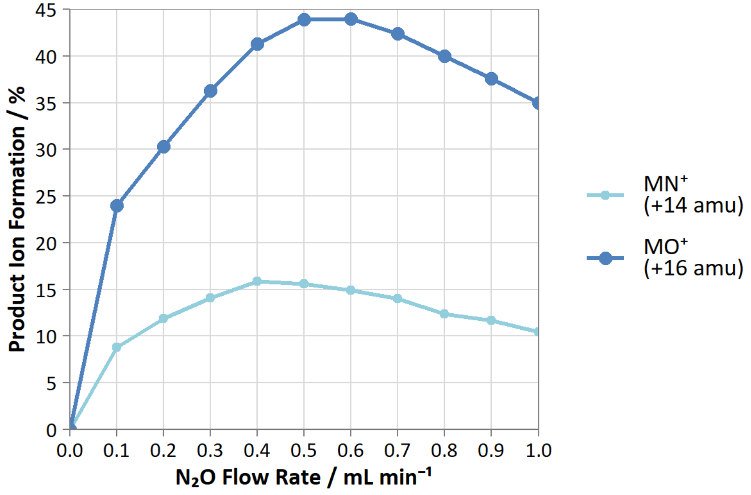

Major Product Ions of $^{140}Ce^+$ with O_2 Reaction Gas

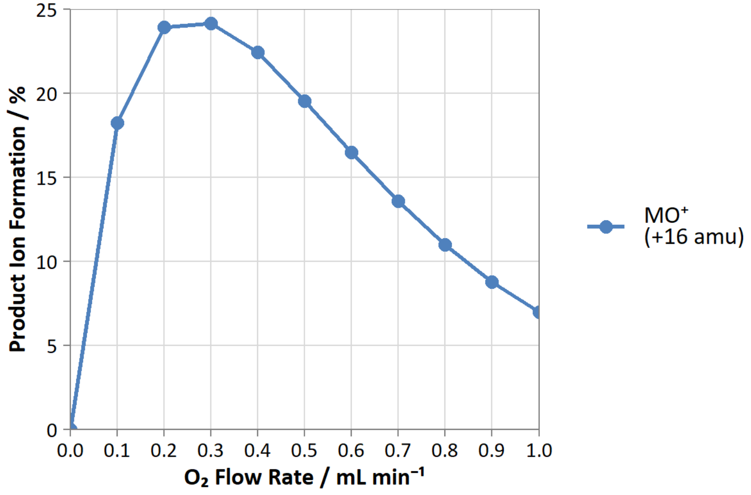

On-Mass with N_2O and O_2

On-Mass Profile of $^{140}Ce^+$ with N_2O and O_2

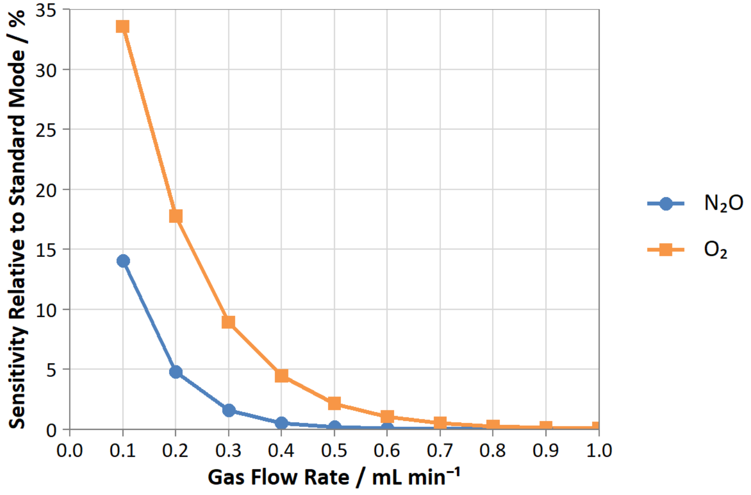

On-Mass Removal (as Interference) with N_2O

Variation of $^{140}Ce^{16}O^+$ Interference with N_2O Flow Rate (On-Mass: Q1 = 156, Q3 = 156)

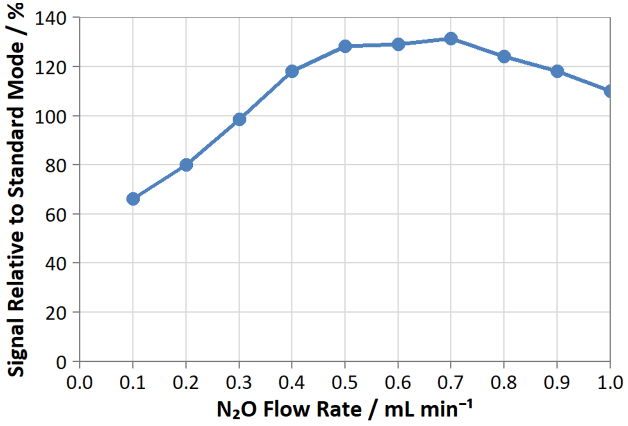

Variation of $^{140}Ce^{2+}$ Interference with N_2O Flow Rate (On-Mass: Q1 = 70, Q3 = 70)

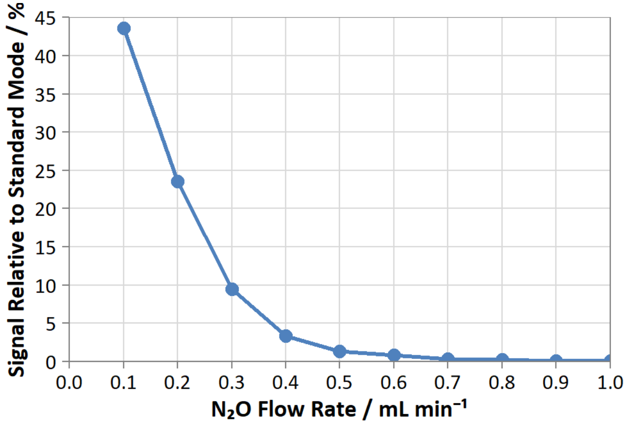

Asymmetric Charge Transfer (with N_2O)

[NO CHARGE TRANSFER REACTION OBSERVED]

Praseodymium

Supplementary Information B to: Characterisation of gas cell reactions for 70+ elements using N_2O for ICP tandem mass spectrometry measurements
Shaun T. Lancaster[a*], Thomas Prohaska[ab] and Johanna Irrgeher[ab]
[a]Department of General, Analytical and Physical Chemistry, Chair of General and Analytical Chemistry, Montanuniversität Leoben, Leoben, Austria.
[b]Department of Physics and Astronomy, University of Calgary, Calgary, Canada.

Mass-Shift with N_2O and O_2

Major Product Ions of $^{141}Pr^+$ with N_2O Reaction Gas

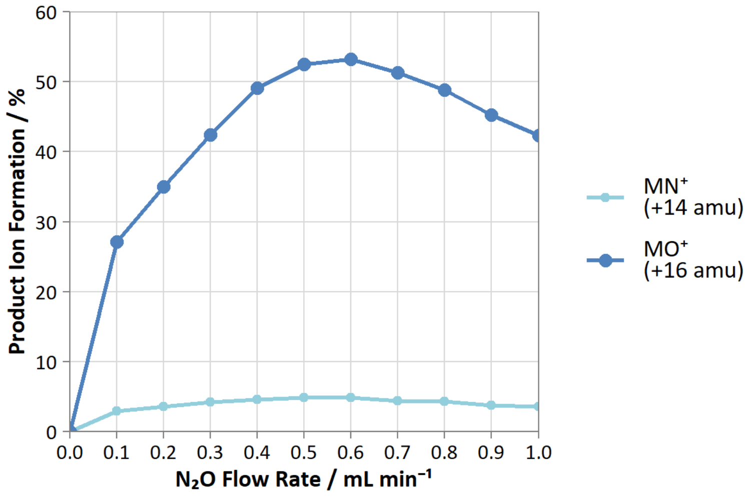

Major Product Ions of $^{141}Pr^+$ with O_2 Reaction Gas

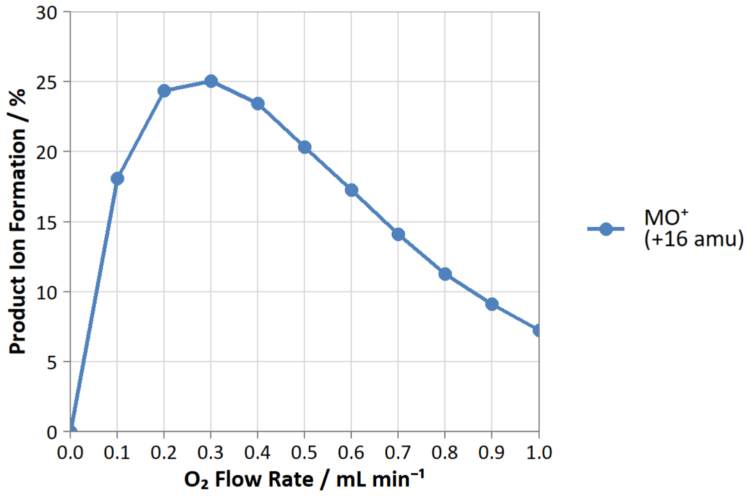

On-Mass with N_2O and O_2

On-Mass Profile of $^{141}Pr^+$ with N_2O and O_2

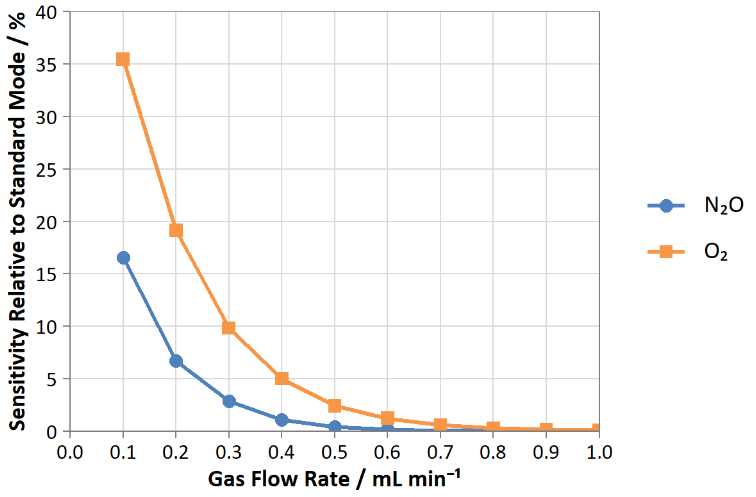

On-Mass Removal (as Interference) with N_2O

Variation of $^{141}Pr^{16}O^+$ Interference with N_2O Flow Rate (On-Mass: Q1 = 157, Q3 = 157)

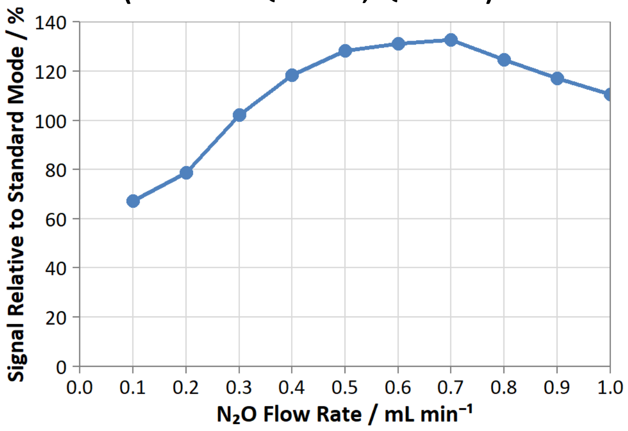

Variation of $^{141}Pr^{2+}$ Interference with N_2O Flow Rate (On-Mass: Q1 = 70.5, Q3 = 70.5)

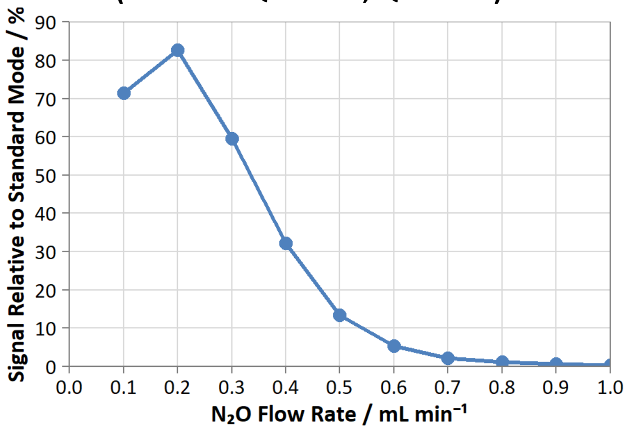

Asymmetric Charge Transfer (with N_2O)

[NO CHARGE TRANSFER REACTION OBSERVED]

Neodymium

Shaun T. Lancaster[a]*, Thomas Prohaska[ab] and Johanna Irrgeher[ab]
[a]Department of General, Analytical and Physical Chemistry, Chair of General and Analytical Chemistry, Montanuniversität Leoben, Leoben, Austria.
[b]Department of Physics and Astronomy, University of Calgary, Calgary, Canada.

Mass-Shift with N_2O and O_2

Major Product Ions of $^{143}Nd^+$ with N_2O Reaction Gas

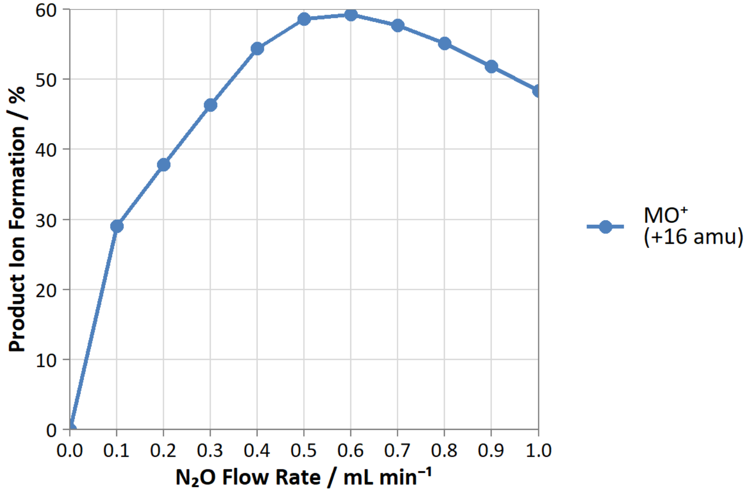

Major Product Ions of $^{143}Nd^+$ with O_2 Reaction Gas

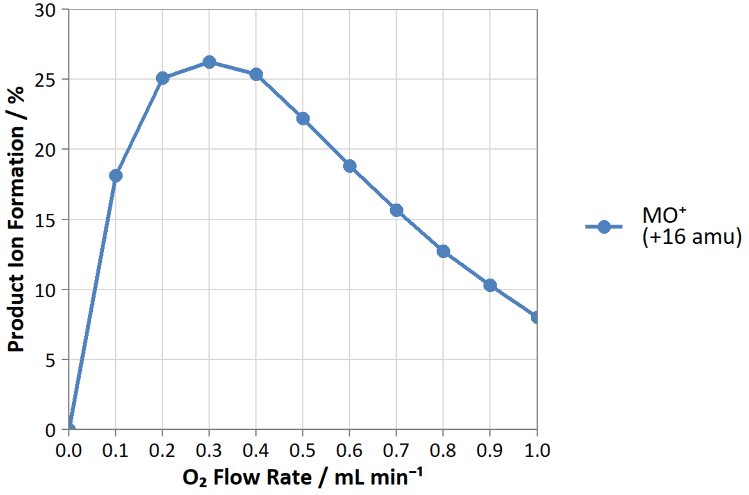

On-Mass with N_2O and O_2

On-Mass Profile of $^{143}Nd^+$ with N_2O and O_2

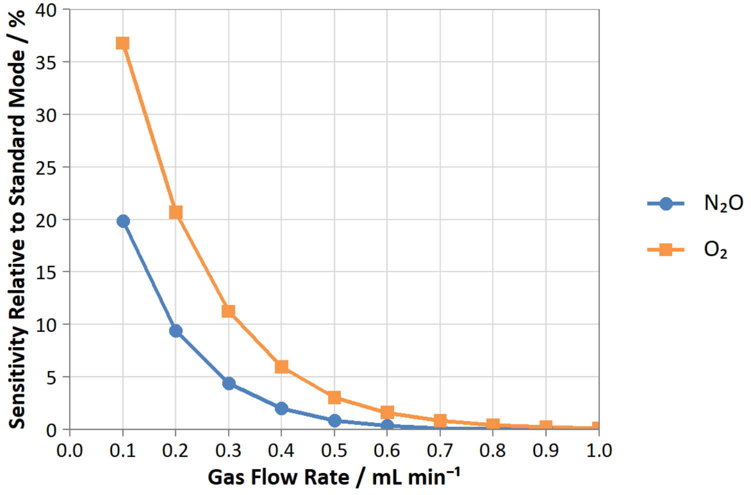

On-Mass Removal (as Interference) with N_2O

Variation of $^{143}Nd^{16}O^+$ Interference with N_2O Flow Rate (On-Mass: Q1 = 159, Q3 = 159)

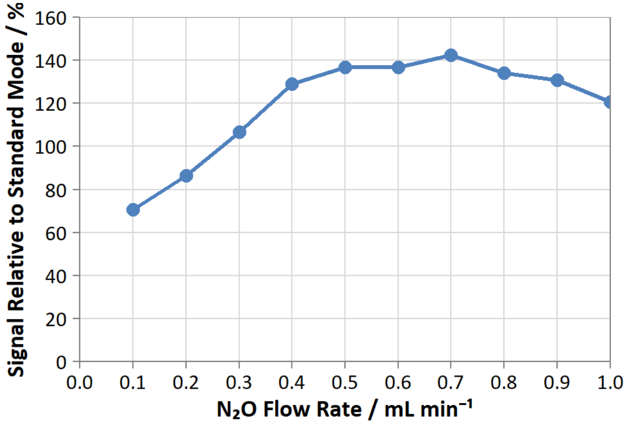

Variation of $^{143}Nd^{2+}$ Interference with N_2O Flow Rate (On-Mass: Q1 = 71.5, Q3 = 71.5)

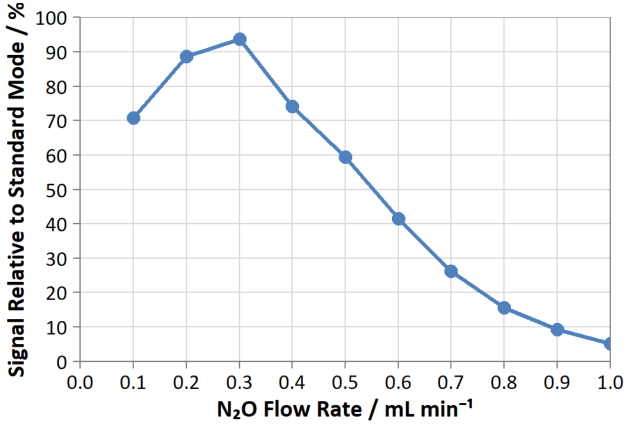

Asymmetric Charge Transfer (with N_2O)

[NO CHARGE TRANSFER REACTION OBSERVED]

Samarium

Supplementary Information B to: Characterisation of gas cell reactions for 70+ elements using N_2O for ICP tandem mass spectrometry measurements
Shaun T. Lancaster[a]*, Thomas Prohaska[ab] and Johanna Irrgeher[ab]
[a]Department of General, Analytical and Physical Chemistry, Chair of General and Analytical Chemistry, Montanuniversität Leoben, Leoben, Austria.
[b]Department of Physics and Astronomy, University of Calgary, Calgary, Canada.

Mass-Shift with N_2O and O_2

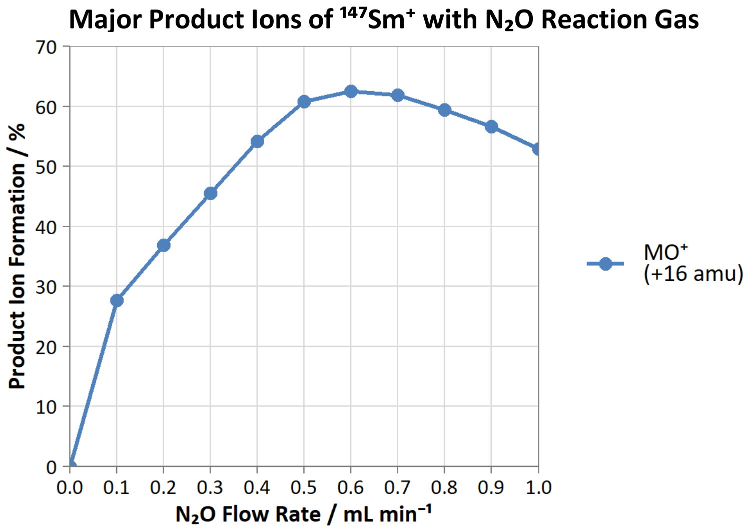

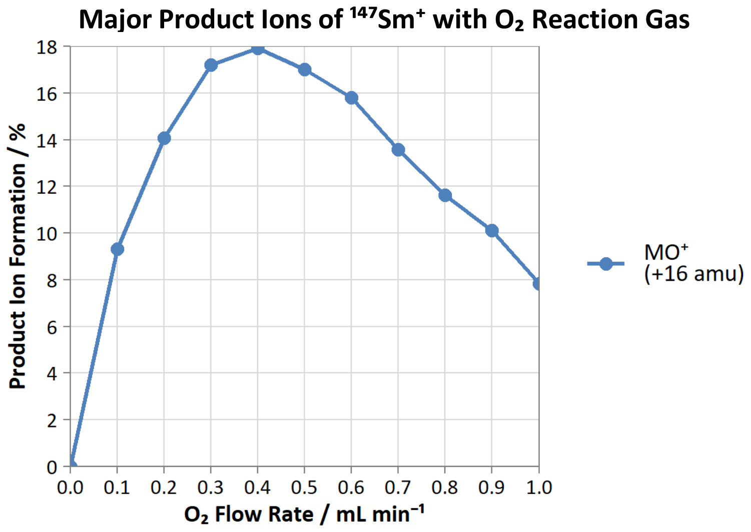

On-Mass with N_2O and O_2

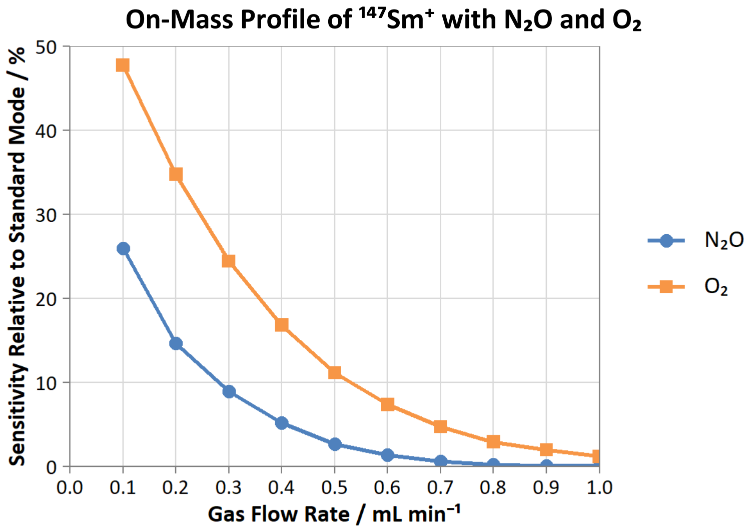

On-Mass Removal (as Interference) with N_2O

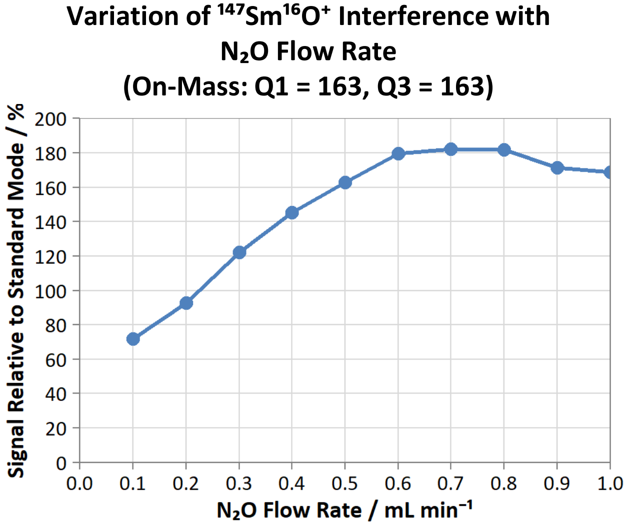

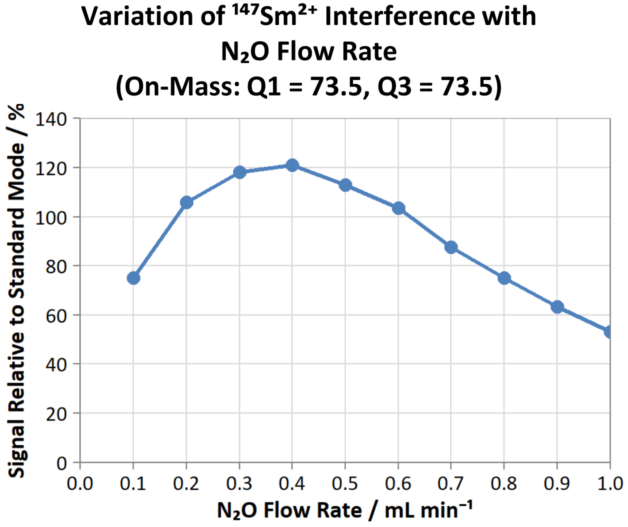

Asymmetric Charge Transfer (with N_2O)

[NO CHARGE TRANSFER REACTION OBSERVED]

Europium

Shaun T. Lancaster[a]*, Thomas Prohaska[ab] and Johanna Irrgeher[ab]
[a]Department of General, Analytical and Physical Chemistry, Chair of General and Analytical Chemistry, Montanuniversität Leoben, Leoben, Austria.
[b]Department of Physics and Astronomy, University of Calgary, Calgary, Canada.

Mass-Shift with N_2O and O_2

Major Product Ions of $^{153}Eu^+$ with N_2O Reaction Gas

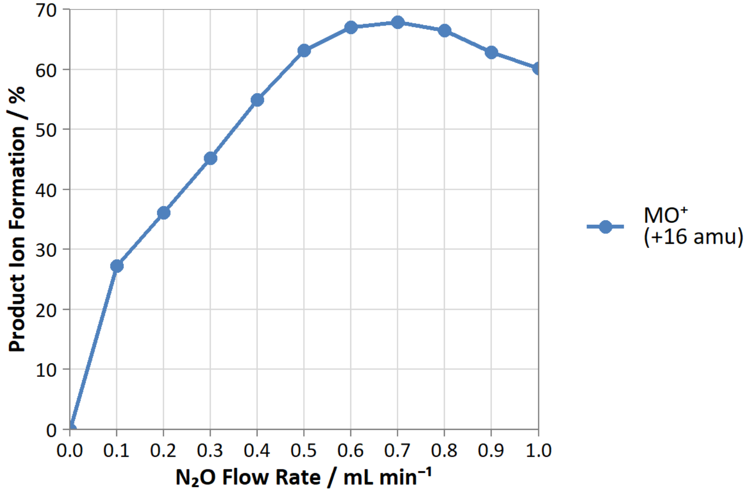

Major Product Ions of $^{153}Eu^+$ with O_2 Reaction Gas

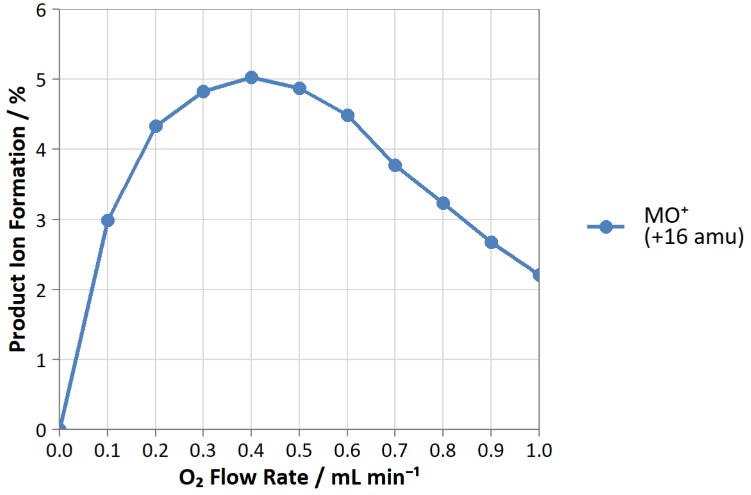

On-Mass with N_2O and O_2

On-Mass Profile of $^{153}Eu^+$ with N_2O and O_2

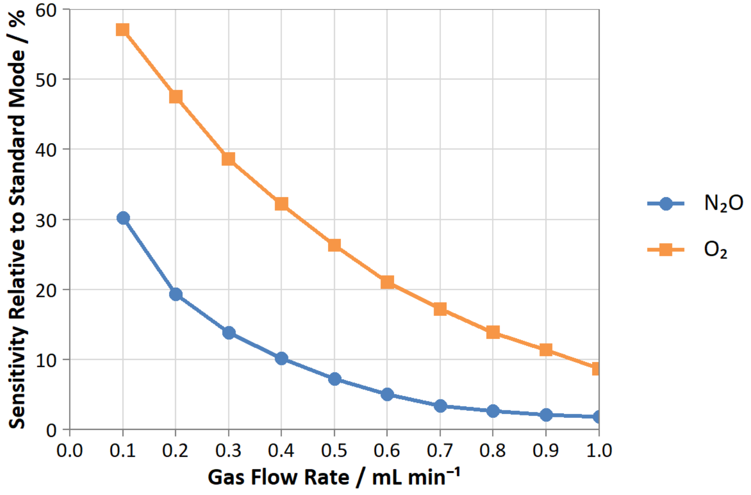

On-Mass Removal (as Interference) with N_2O

Variation of $^{153}Eu^{16}O^+$ Interference with N_2O Flow Rate (On-Mass: Q1 = 169, Q3 = 169)

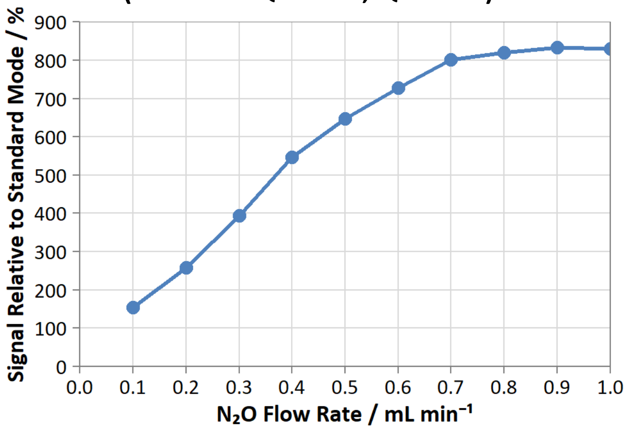

Variation of $^{153}Eu^{2+}$ Interference with N_2O Flow Rate (On-Mass: Q1 = 76.5, Q3 = 76.5)

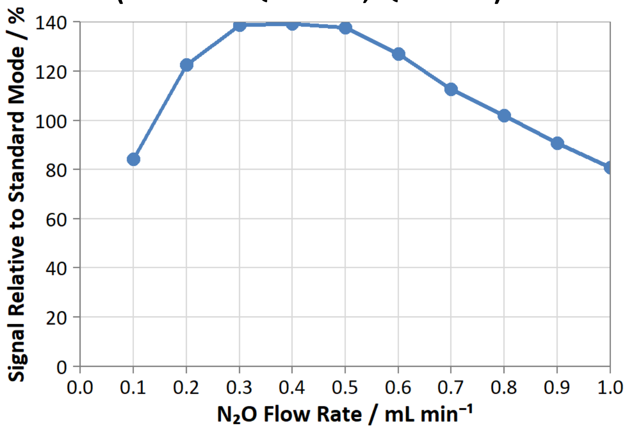

Asymmetric Charge Transfer (with N_2O)

[NO CHARGE TRANSFER REACTION OBSERVED]

Gadolinium

Supplementary Information B to: Characterisation of gas cell reactions for 70+ elements using N_2O for ICP tandem mass spectrometry measurements
Shaun T. Lancaster[a]*, Thomas Prohaska[ab] and Johanna Irrgeher[ab]
[a]Department of General, Analytical and Physical Chemistry, Chair of General and Analytical Chemistry, Montanuniversität Leoben, Leoben, Austria.
[b]Department of Physics and Astronomy, University of Calgary, Calgary, Canada.

Mass-Shift with N_2O and O_2

Major Product Ions of $^{155}Gd^+$ with N_2O Reaction Gas

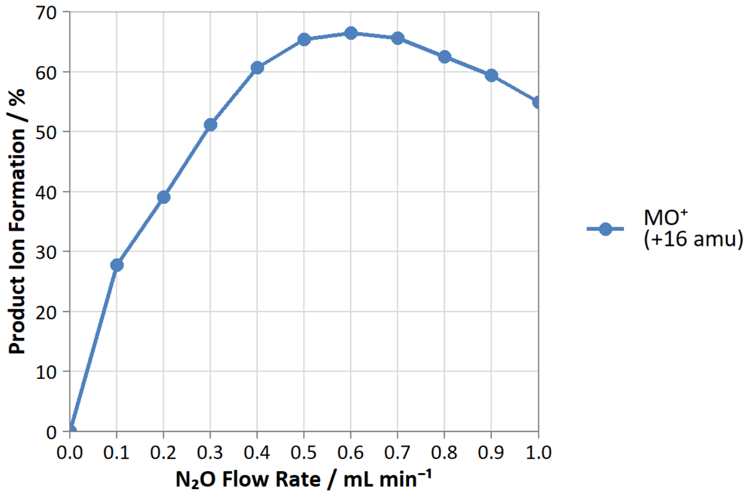

Major Product Ions of $^{155}Gd^+$ with O_2 Reaction Gas

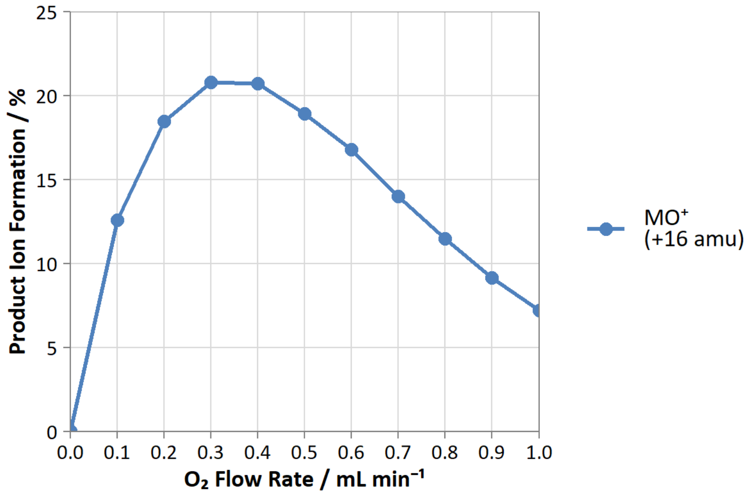

On-Mass with N_2O and O_2

On-Mass Profile of $^{155}Gd^+$ with N_2O and O_2

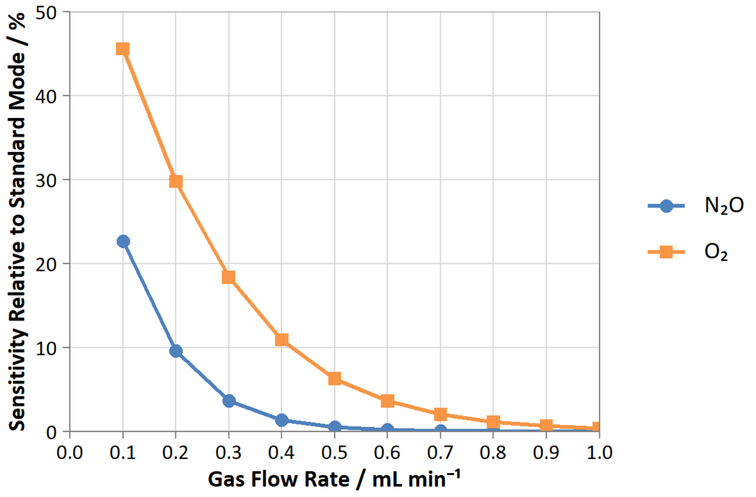

On-Mass Removal (as Interference) with N_2O

Variation of $^{155}Gd^{16}O^+$ Interference with N_2O Flow Rate (On-Mass: Q1 = 171, Q3 = 171)

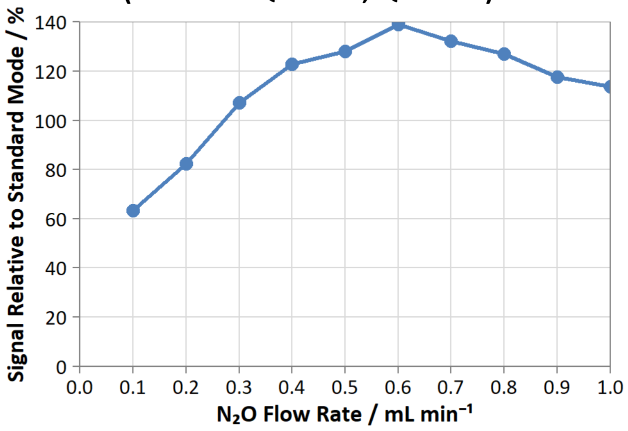

Variation of $^{155}Gd^{2+}$ Interference with N_2O Flow Rate (On-Mass: Q1 = 77.5, Q3 = 77.5)

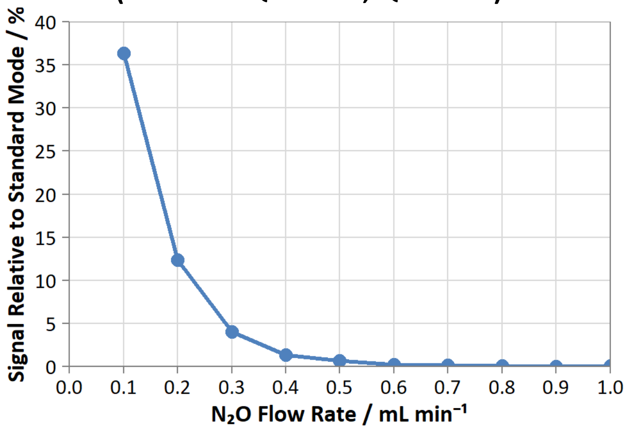

Asymmetric Charge Transfer (with N_2O)

[NO CHARGE TRANSFER REACTION OBSERVED]

Terbium

Supplementary Information B to: Characterisation of gas cell reactions for 70+ elements using N_2O for ICP tandem mass spectrometry measurements
Shaun T. Lancaster[a]*, Thomas Prohaska[ab] and Johanna Irrgeher[ab]
[a]Department of General, Analytical and Physical Chemistry, Chair of General and Analytical Chemistry, Montanuniversität Leoben, Leoben, Austria.
[b]Department of Physics and Astronomy, University of Calgary, Calgary, Canada.

Mass-Shift with N_2O and O_2

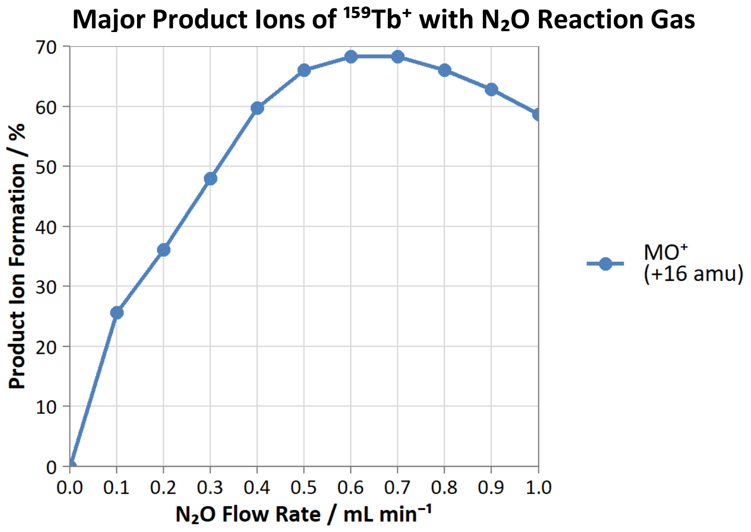

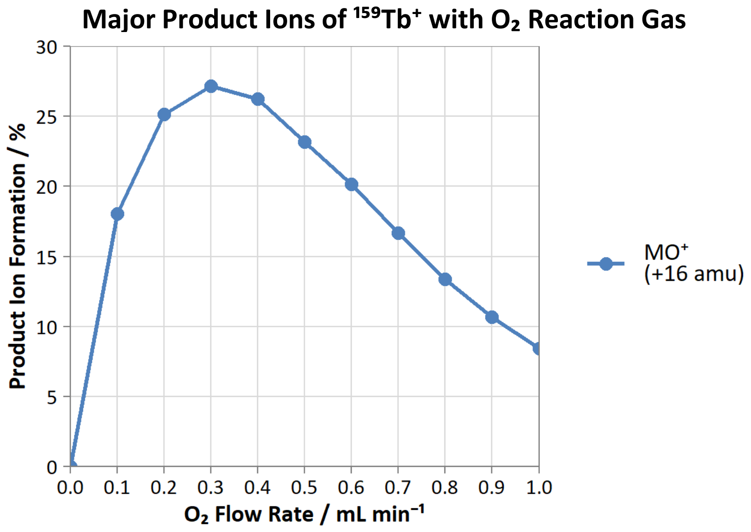

On-Mass with N_2O and O_2

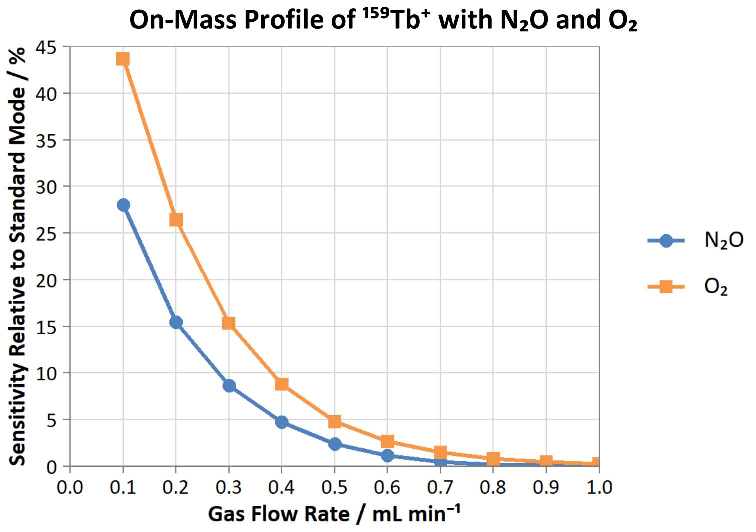

On-Mass Removal (as Interference) with N_2O

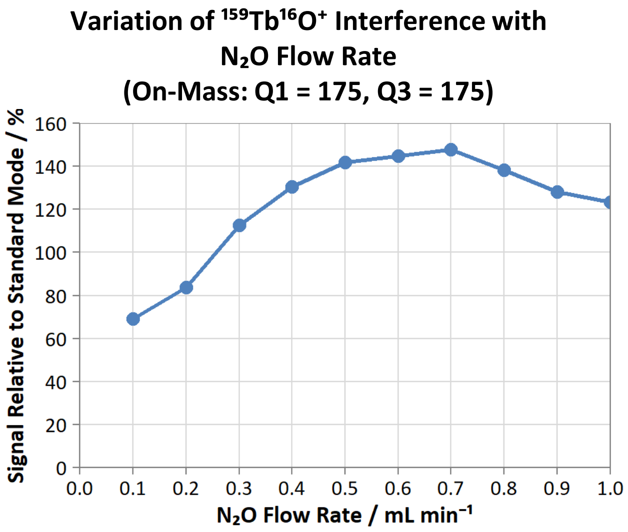

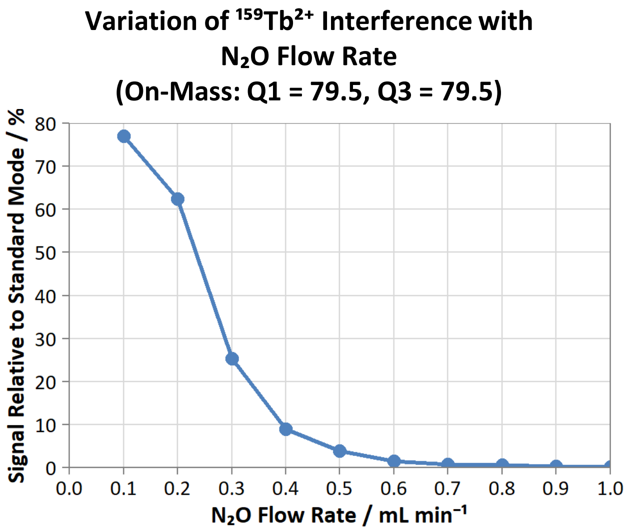

Asymmetric Charge Transfer (with N_2O)

[NO CHARGE TRANSFER REACTION OBSERVED]

Dysprosium

Supplementary Information B to: Characterisation of gas cell reactions for 70+ elements using N_2O for ICP tandem mass spectrometry measurements
Shaun T. Lancaster[a]*, Thomas Prohaska[ab] and Johanna Irrgeher[ab]
[a]Department of General, Analytical and Physical Chemistry, Chair of General and Analytical Chemistry, Montanuniversität Leoben, Leoben, Austria.
[b]Department of Physics and Astronomy, University of Calgary, Calgary, Canada.

Mass-Shift with N_2O and O_2

Major Product Ions of $^{163}Dy^+$ with N_2O Reaction Gas

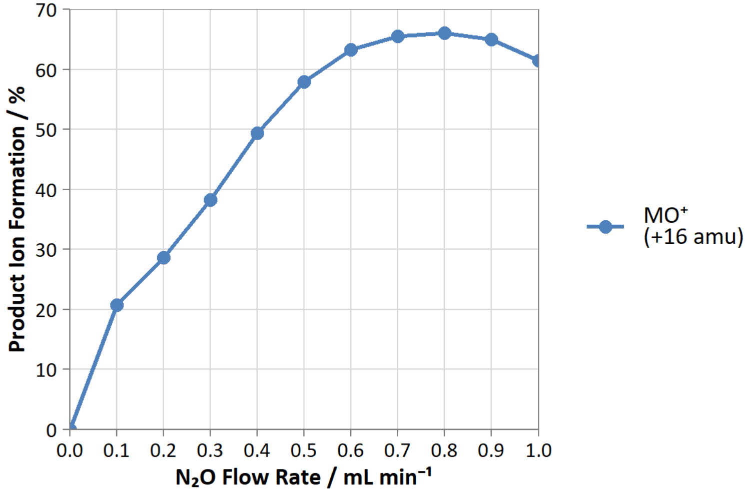

Major Product Ions of $^{163}Dy^+$ with O_2 Reaction Gas

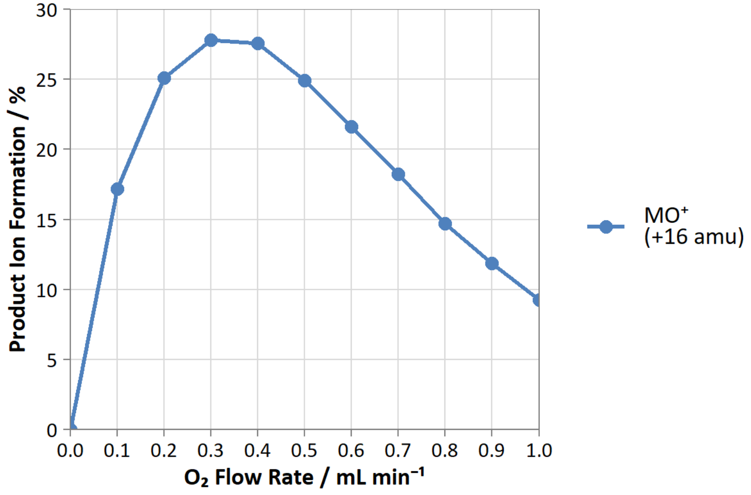

On-Mass with N_2O and O_2

On-Mass Profile of $^{163}Dy^+$ with N_2O and O_2

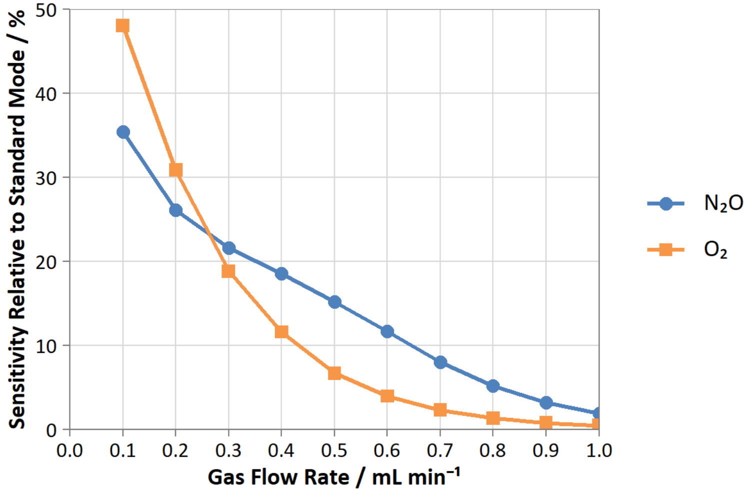

On-Mass Removal (as Interference) with N_2O

Variation of $^{163}Dy^{16}O^+$ Interference with N_2O Flow Rate (On-Mass: Q1 = 179, Q3 = 179)

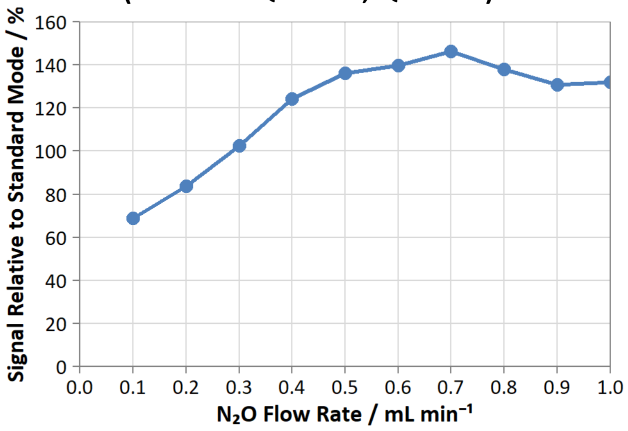

Variation of $^{163}Dy^{2+}$ Interference with N_2O Flow Rate (On-Mass: Q1 = 81.5, Q3 = 81.5)

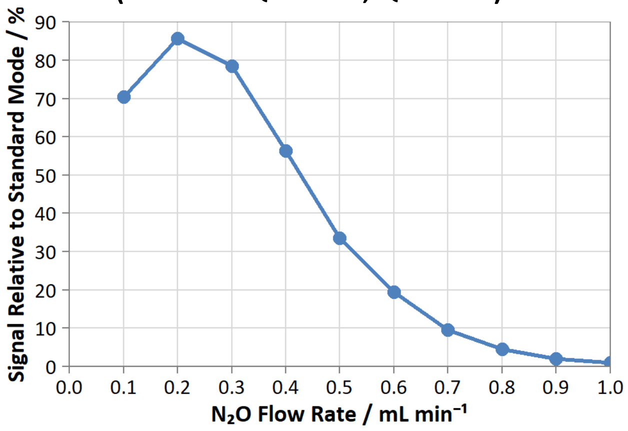

Asymmetric Charge Transfer (with N_2O)

[NO CHARGE TRANSFER REACTION OBSERVED]

Holmium

Supplementary Information B to: Characterisation of gas cell reactions for 70+ elements using N_2O for ICP tandem mass spectrometry measurements
Shaun T. Lancaster[a]*, Thomas Prohaska[ab] and Johanna Irrgeher[ab]
[a]Department of General, Analytical and Physical Chemistry, Chair of General and Analytical Chemistry, Montanuniversität Leoben, Leoben, Austria.
[b]Department of Physics and Astronomy, University of Calgary, Calgary, Canada.

Mass-Shift with N_2O and O_2

Major Product Ions of $^{165}Ho^+$ with N_2O Reaction Gas

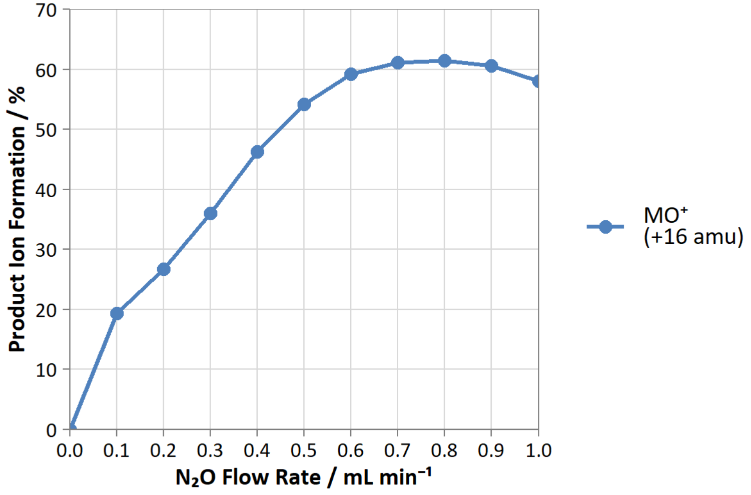

Major Product Ions of $^{165}Ho^+$ with O_2 Reaction Gas

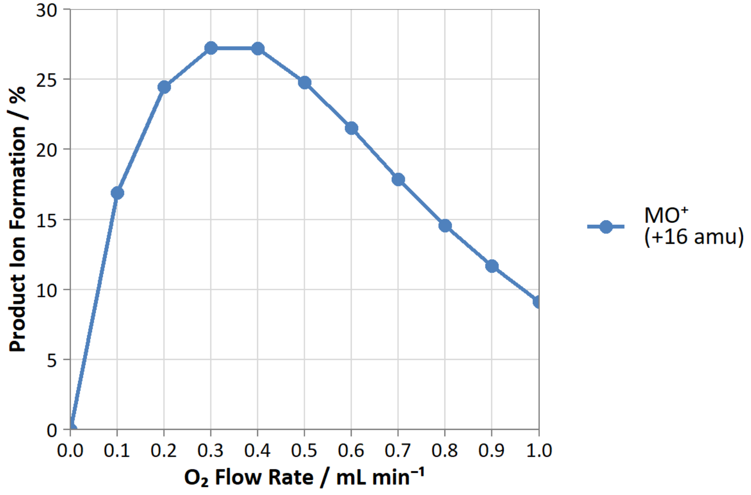

On-Mass with N_2O and O_2

On-Mass Profile of $^{165}Ho^+$ with N_2O and O_2

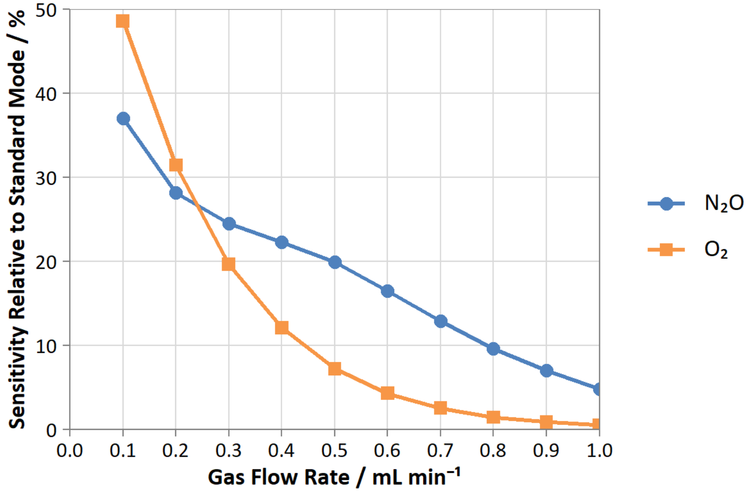

On-Mass Removal (as Interference) with N_2O

Variation of $^{165}Ho^{16}O^+$ Interference with N_2O Flow Rate (On-Mass: Q1 = 181, Q3 = 181)

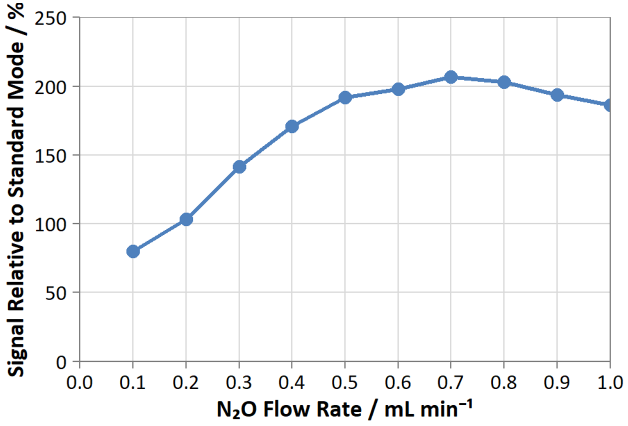

Variation of $^{165}Ho^{2+}$ Interference with N_2O Flow Rate (On-Mass: Q1 = 82.5, Q3 = 82.5)

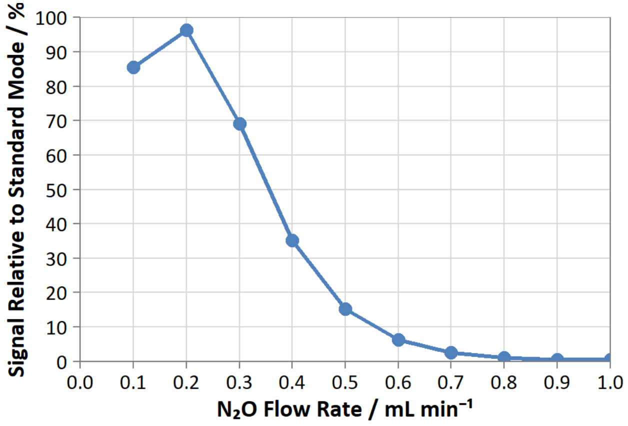

Asymmetric Charge Transfer (with N_2O)

[NO CHARGE TRANSFER REACTION OBSERVED]

Erbium

Supplementary Information B to: Characterisation of gas cell reactions for 70+ elements using N_2O for ICP tandem mass spectrometry measurements
Shaun T. Lancaster[a]*, Thomas Prohaska[ab] and Johanna Irrgeher[ab]
[a]Department of General, Analytical and Physical Chemistry, Chair of General and Analytical Chemistry, Montanuniversität Leoben, Leoben, Austria.
[b]Department of Physics and Astronomy, University of Calgary, Calgary, Canada.

Mass-Shift with N_2O and O_2

Major Product Ions of $^{166}Er^+$ with N_2O Reaction Gas

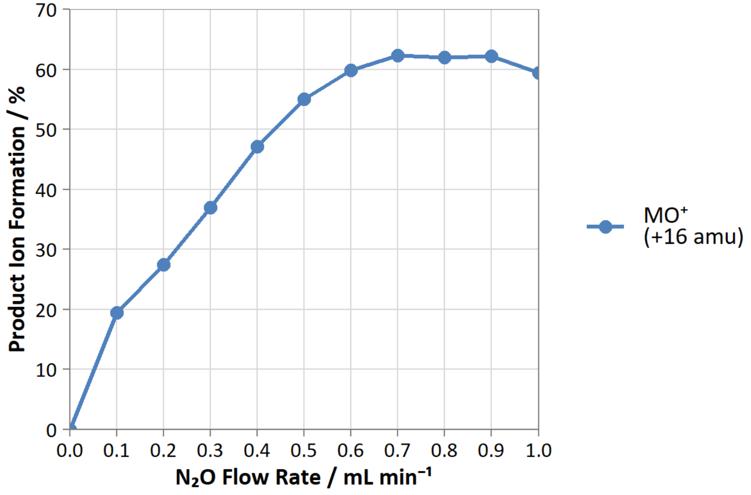

Major Product Ions of $^{166}Er^+$ with O_2 Reaction Gas

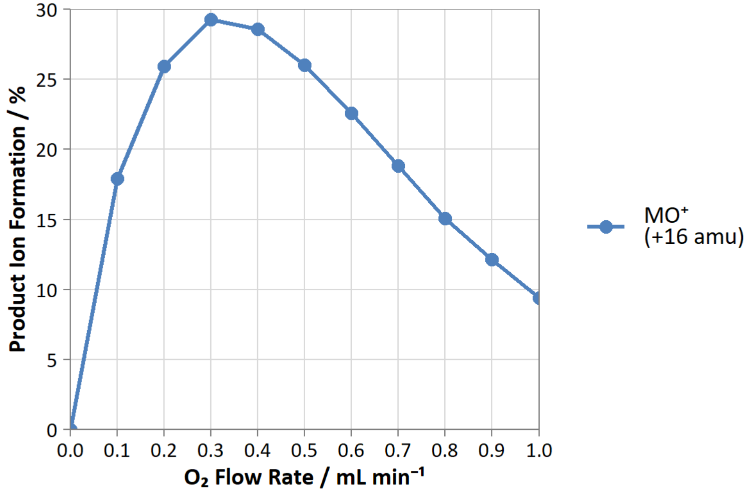

On-Mass with N_2O and O_2

On-Mass Profile of $^{166}Er^+$ with N_2O and O_2

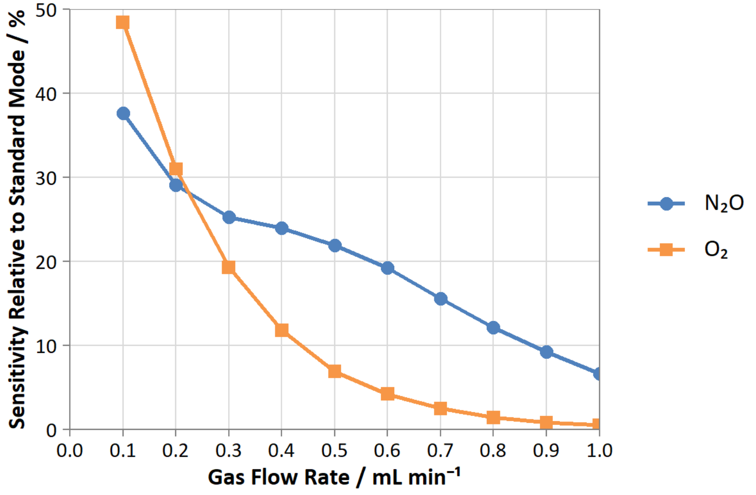

On-Mass Removal (as Interference) with N_2O

Variation of $^{166}Er^{16}O^+$ Interference with N_2O Flow Rate (On-Mass: Q1 = 182, Q3 = 182)

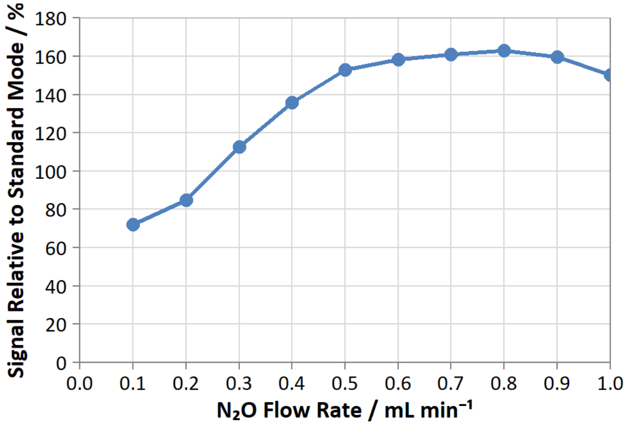

Variation of $^{166}Er^{2+}$ Interference with N_2O Flow Rate (On-Mass: Q1 = 83, Q3 = 83)

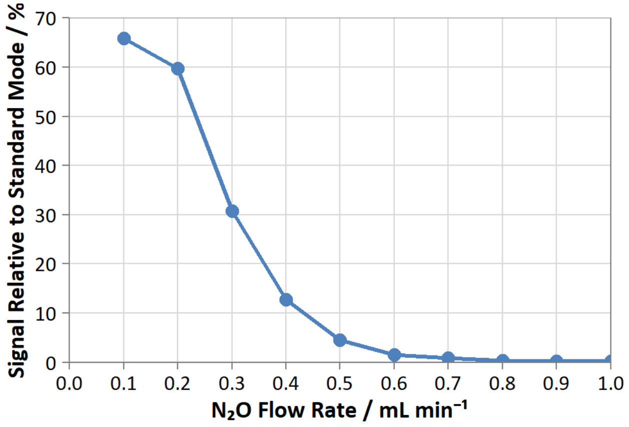

Asymmetric Charge Transfer (with N_2O)

[NO CHARGE TRANSFER REACTION OBSERVED]

Thulium

Supplementary Information B to: Characterisation of gas cell reactions for 70+ elements using N_2O for ICP tandem mass spectrometry measurements
Shaun T. Lancaster[a]*, Thomas Prohaska[ab] and Johanna Irrgeher[ab]
[a]Department of General, Analytical and Physical Chemistry, Chair of General and Analytical Chemistry, Montanuniversität Leoben, Leoben, Austria.
[b]Department of Physics and Astronomy, University of Calgary, Calgary, Canada.

Mass-Shift with N_2O and O_2

Major Product Ions of $^{169}Tm^+$ with N_2O Reaction Gas

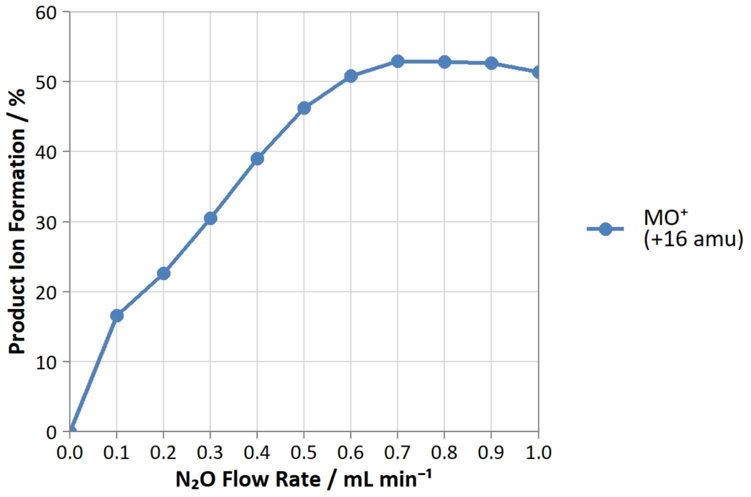

Major Product Ions of $^{169}Tm^+$ with O_2 Reaction Gas

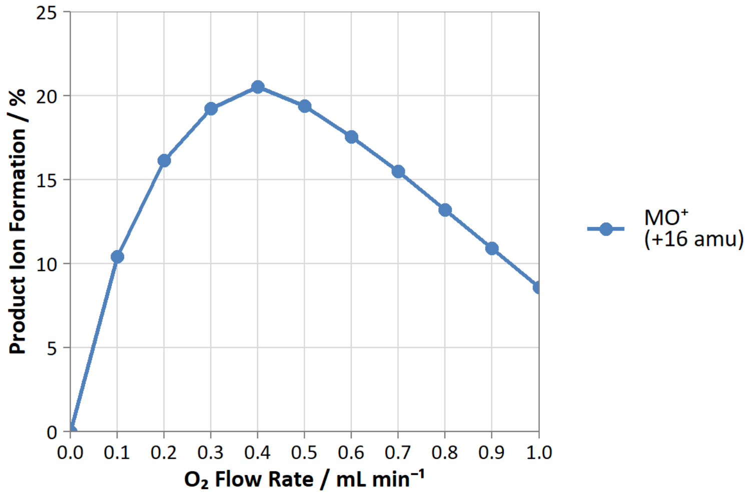

On-Mass with N_2O and O_2

On-Mass Profile of $^{169}Tm^+$ with N_2O and O_2

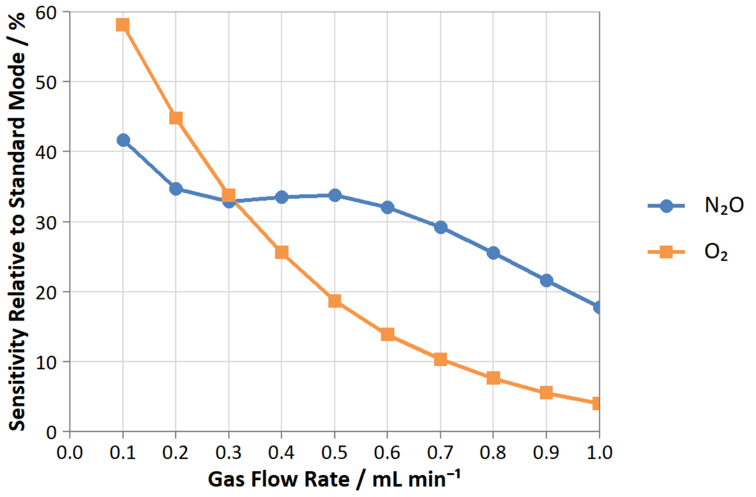

On-Mass Removal (as Interference) with N_2O

Variation of $^{169}Tm^{16}O^+$ Interference with N_2O Flow Rate (On-Mass: Q1 = 185, Q3 = 185)

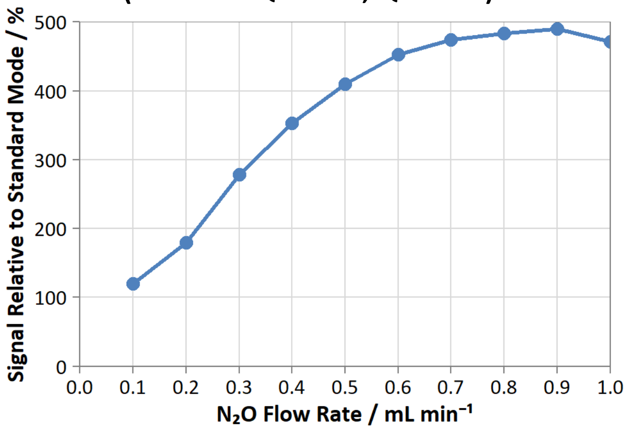

Variation of $^{169}Tm^{2+}$ Interference with N_2O Flow Rate (On-Mass: Q1 = 84.5, Q3 = 84.5)

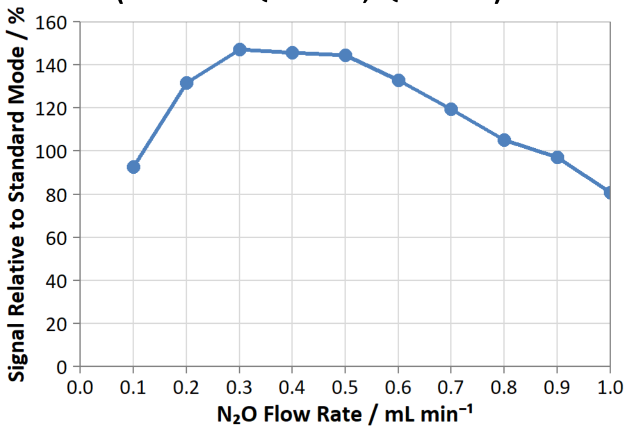

Asymmetric Charge Transfer (with N_2O)

[NO CHARGE TRANSFER REACTION OBSERVED]

Ytterbium

Supplementary Information B to: Characterisation of gas cell reactions for 70+ elements using N_2O for ICP tandem mass spectrometry measurements
Shaun T. Lancaster[a]*, Thomas Prohaska[ab] and Johanna Irrgeher[ab]
[a]Department of General, Analytical and Physical Chemistry, Chair of General and Analytical Chemistry, Montanuniversität Leoben, Leoben, Austria.
[b]Department of Physics and Astronomy, University of Calgary, Calgary, Canada.

Mass-Shift with N_2O and O_2

Major Product Ions of $^{174}Yb^+$ with N_2O Reaction Gas

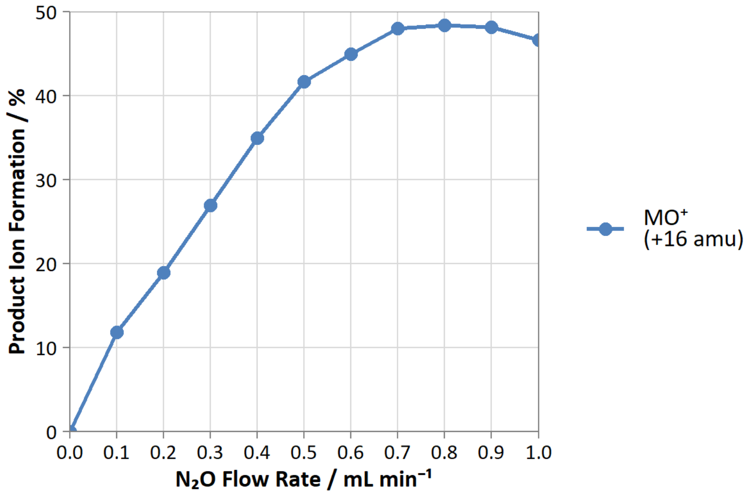

Major Product Ions of $^{174}Yb^+$ with O_2 Reaction Gas

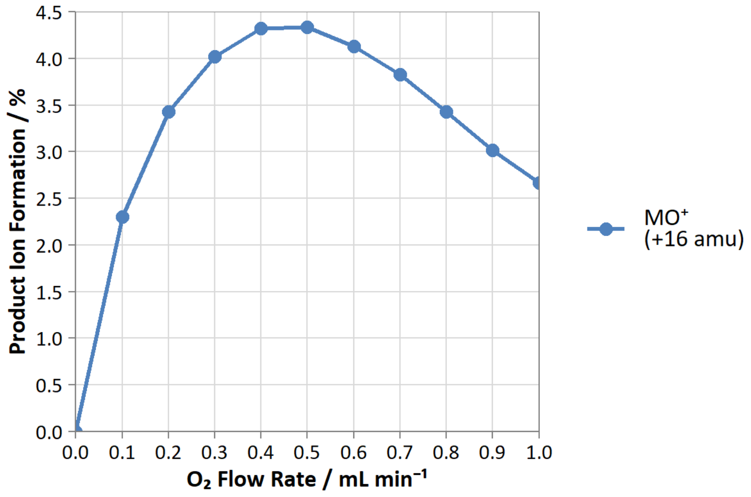

On-Mass with N_2O and O_2

On-Mass Profile of $^{174}Yb^+$ with N_2O and O_2

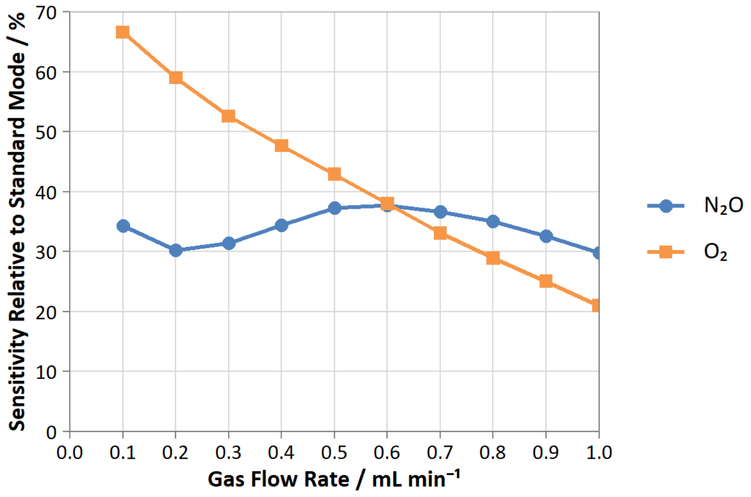

On-Mass Removal (as Interference) with N_2O

Variation of $^{174}Yb^{16}O^+$ Interference with N_2O Flow Rate (On-Mass: Q1 = 190, Q3 = 190)

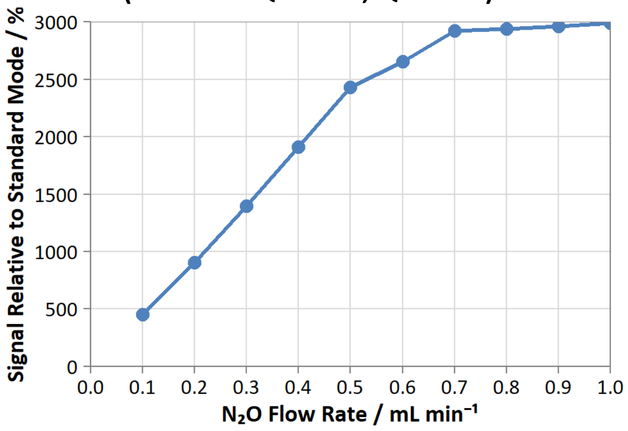

Variation of $^{174}Yb^{2+}$ Interference with N_2O Flow Rate (On-Mass: Q1 = 87, Q3 = 87)

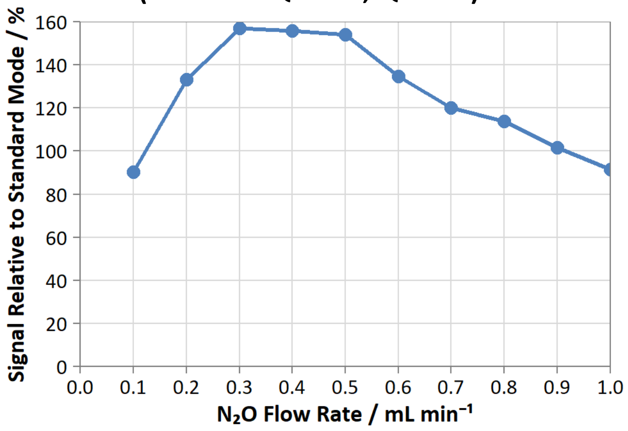

Asymmetric Charge Transfer (with N_2O)

[NO CHARGE TRANSFER REACTION OBSERVED]

Lutetium

Supplementary Information B to: Characterisation of gas cell reactions for 70+ elements using N_2O for ICP tandem mass spectrometry measurements
Shaun T. Lancaster[a]*, Thomas Prohaska[ab] and Johanna Irrgeher[ab]
[a]Department of General, Analytical and Physical Chemistry, Chair of General and Analytical Chemistry, Montanuniversität Leoben, Leoben, Austria.
[b]Department of Physics and Astronomy, University of Calgary, Calgary, Canada.

Mass-Shift with N_2O and O_2

Major Product Ions of $^{175}Lu^+$ with N_2O Reaction Gas

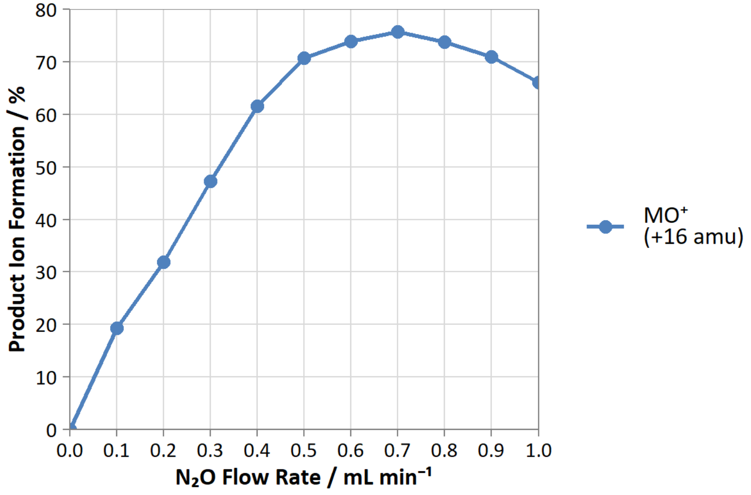

Major Product Ions of $^{175}Lu^+$ with O_2 Reaction Gas

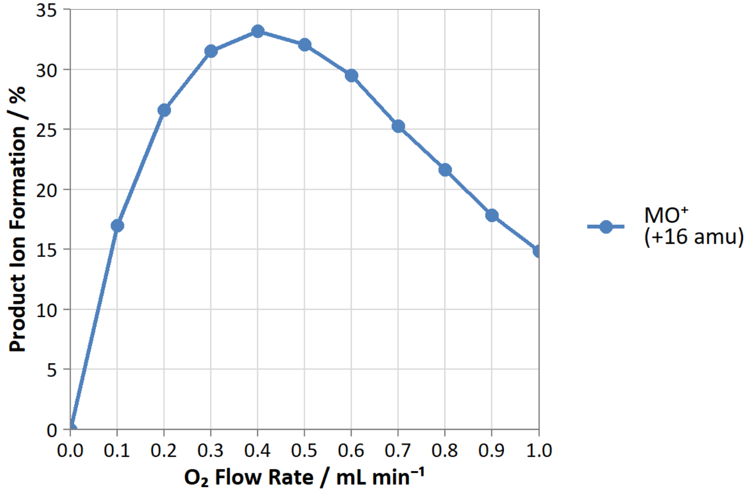

On-Mass with N_2O and O_2

On-Mass Profile of $^{175}Lu^+$ with N_2O and O_2

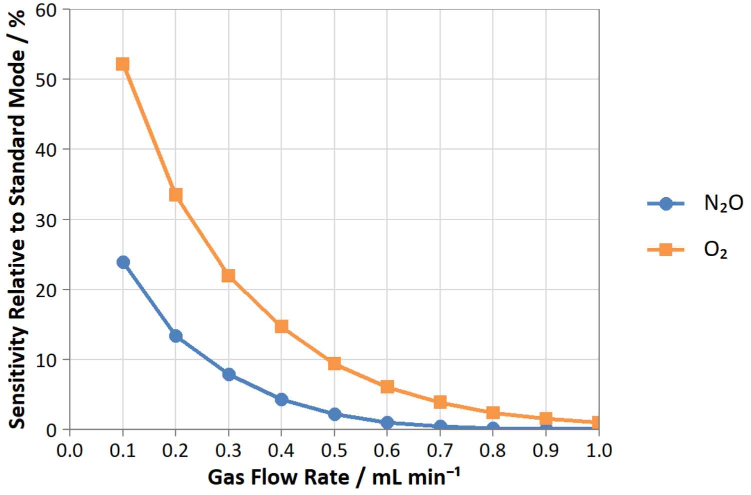

On-Mass Removal (as Interference) with N_2O

Variation of $^{175}Lu^{16}O^+$ Interference with N_2O Flow Rate (On-Mass: Q1 = 191, Q3 = 191)

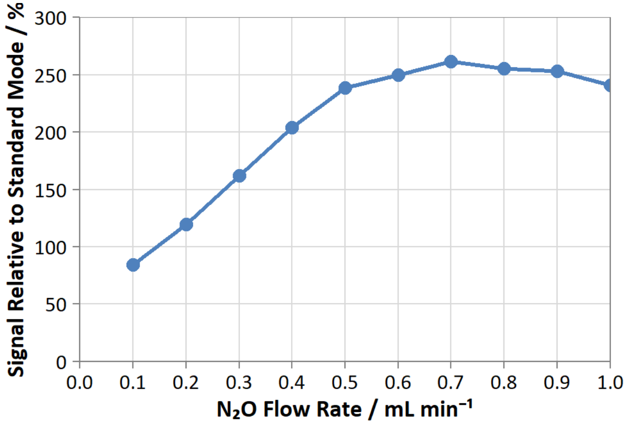

Variation of $^{175}Lu^{2+}$ Interference with N_2O Flow Rate (On-Mass: Q1 = 87.5, Q3 = 87.5)

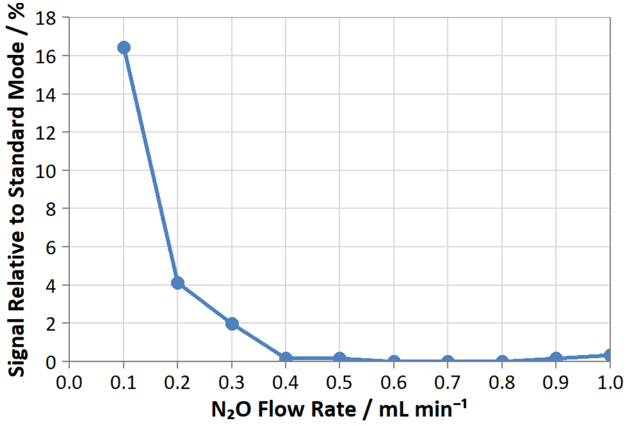

Asymmetric Charge Transfer (with N_2O)

[NO CHARGE TRANSFER REACTION OBSERVED]

Hafnium

Supplementary Information B to: Characterisation of gas cell reactions for 70+ elements using N_2O for ICP tandem mass spectrometry measurements
Shaun T. Lancaster[a]*, Thomas Prohaska[ab] and Johanna Irrgeher[ab]
[a]Department of General, Analytical and Physical Chemistry, Chair of General and Analytical Chemistry, Montanuniversität Leoben, Leoben, Austria.
[b]Department of Physics and Astronomy, University of Calgary, Calgary, Canada.

Mass-Shift with N_2O and O_2

Major Product Ions of $^{180}Hf^+$ with N_2O Reaction Gas

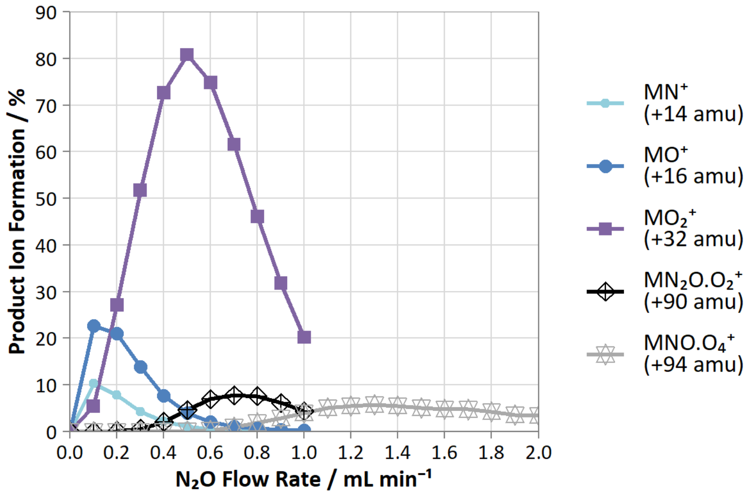

Major Product Ions of $^{180}Hf^+$ with O_2 Reaction Gas

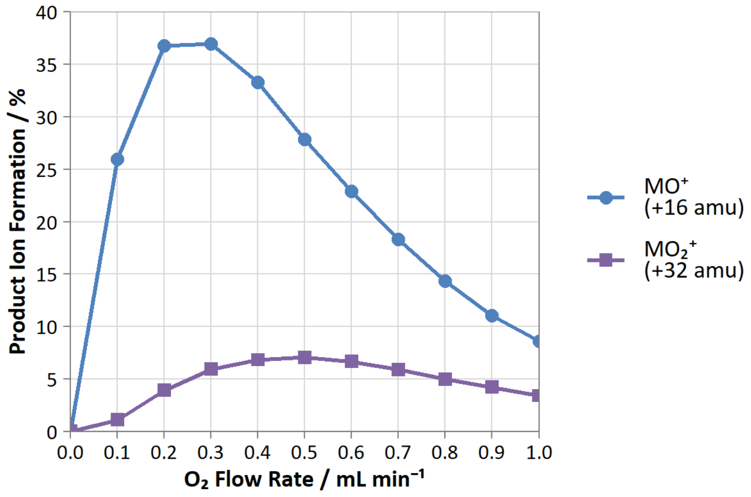

On-Mass with N_2O and O_2

On-Mass Profile of $^{180}Hf^+$ with N_2O and O_2

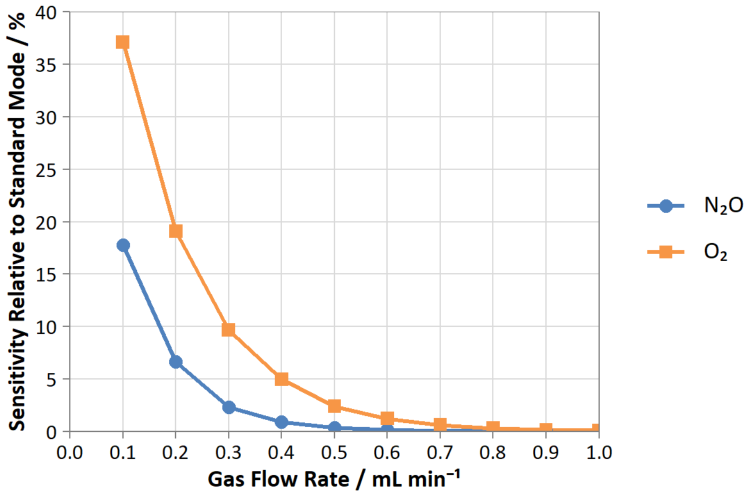

On-Mass Removal (as Interference) with N_2O

Variation of $^{180}Hf^{16}O^+$ Interference with N_2O Flow Rate (On-Mass: Q1 = 196, Q3 = 196)

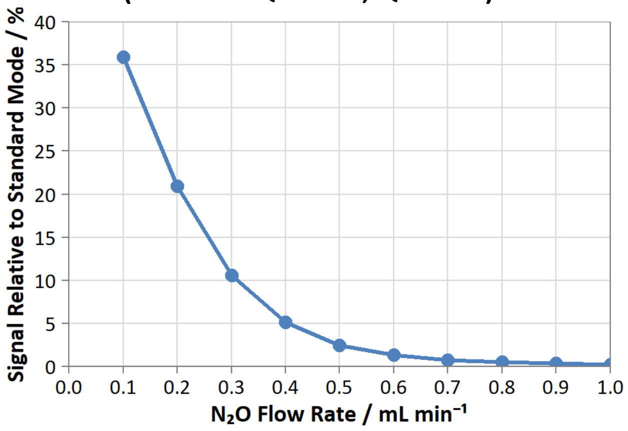

Variation of $^{180}Hf^{2+}$ Interference with N_2O Flow Rate (On-Mass: Q1 = 90, Q3 = 90)

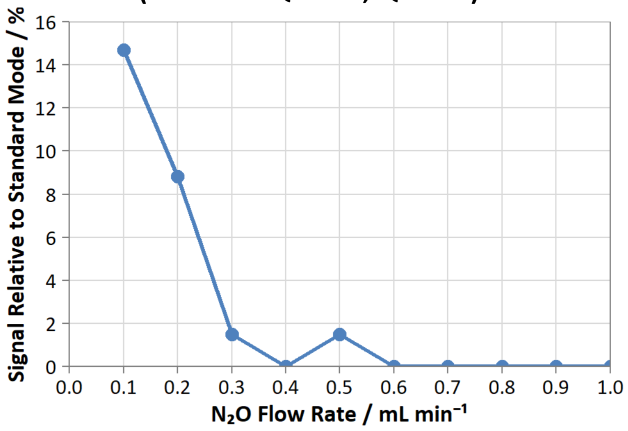

Asymmetric Charge Transfer (with N_2O)

[NO CHARGE TRANSFER REACTION OBSERVED]

Tantalum

Supplementary Information B to: Characterisation of gas cell reactions for 70+ elements using N_2O for ICP tandem mass spectrometry measurements
Shaun T. Lancaster[a]*, Thomas Prohaska[ab] and Johanna Irrgeher[ab]
[a]Department of General, Analytical and Physical Chemistry, Chair of General and Analytical Chemistry, Montanuniversität Leoben, Leoben, Austria.
[b]Department of Physics and Astronomy, University of Calgary, Calgary, Canada.

Mass-Shift with N_2O and O_2

Major Product Ions of $^{181}Ta^+$ with N_2O Reaction Gas

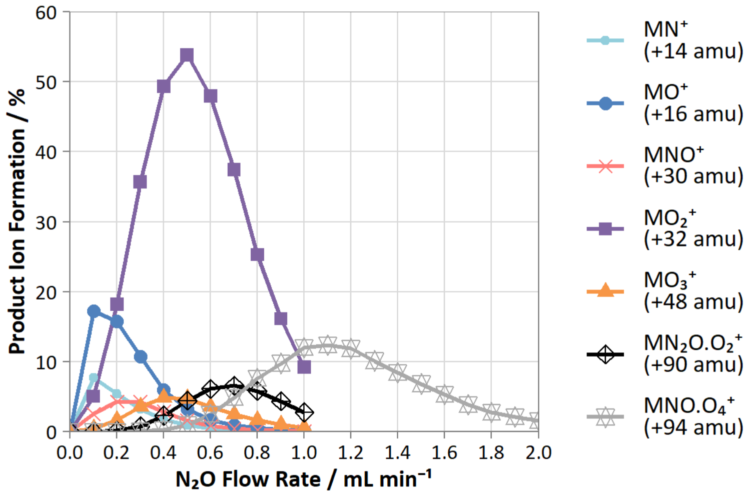

Major Product Ions of $^{181}Ta^+$ with O_2 Reaction Gas

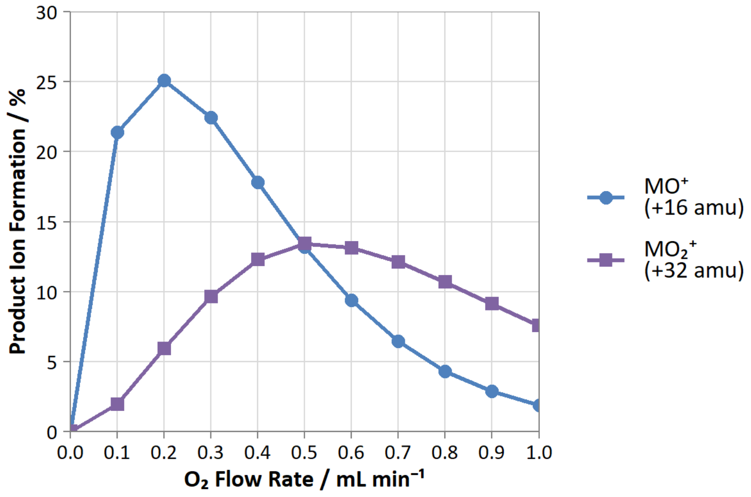

On-Mass with N_2O and O_2

On-Mass Profile of $^{181}Ta^+$ with N_2O and O_2

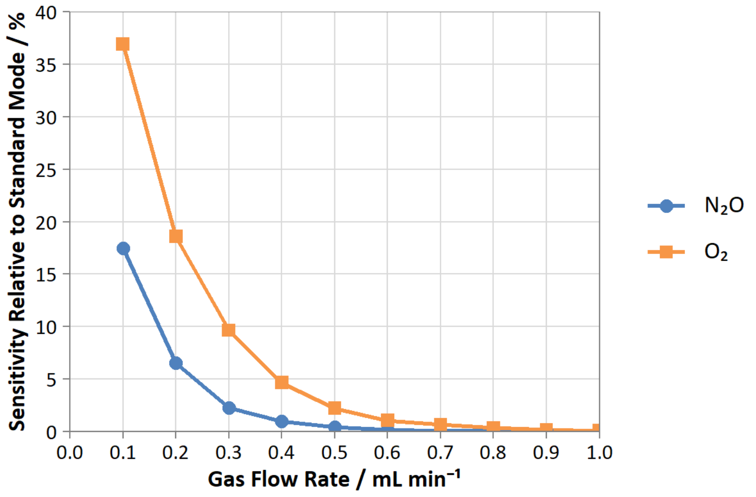

On-Mass Removal (as Interference) with N_2O

Variation of $^{181}Ta^{16}O^+$ Interference with N_2O Flow Rate (On-Mass: Q1 = 197, Q3 = 197)

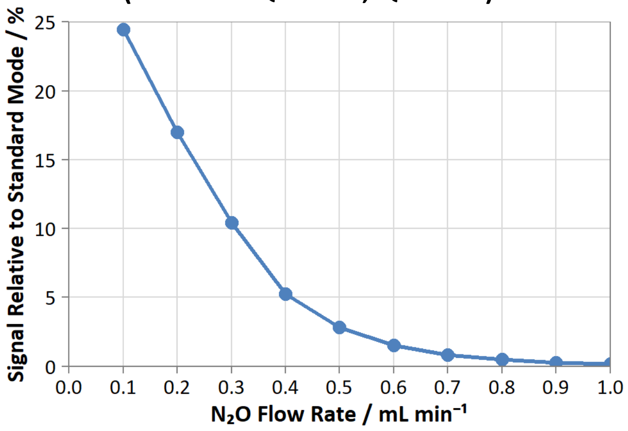

Variation of $^{181}Ta^{2+}$ Interference with N_2O Flow Rate (On-Mass: Q1 = 90.5, Q3 = 90.5)

[INSUFFICIENT DOUBLY-CHARGED ION FORMATION OBSERVED]

Asymmetric Charge Transfer (with N_2O)

[NO CHARGE TRANSFER REACTION OBSERVED]

Tungsten

Supplementary Information B to: Characterisation of gas cell reactions for 70+ elements using N_2O for ICP tandem mass spectrometry measurements
Shaun T. Lancaster[a]*, Thomas Prohaska[ab] and Johanna Irrgeher[ab]
[a]Department of General, Analytical and Physical Chemistry, Chair of General and Analytical Chemistry, Montanuniversität Leoben, Leoben, Austria.
[b]Department of Physics and Astronomy, University of Calgary, Calgary, Canada.

Mass-Shift with N_2O and O_2

Major Product Ions of $^{184}W^+$ with N_2O Reaction Gas

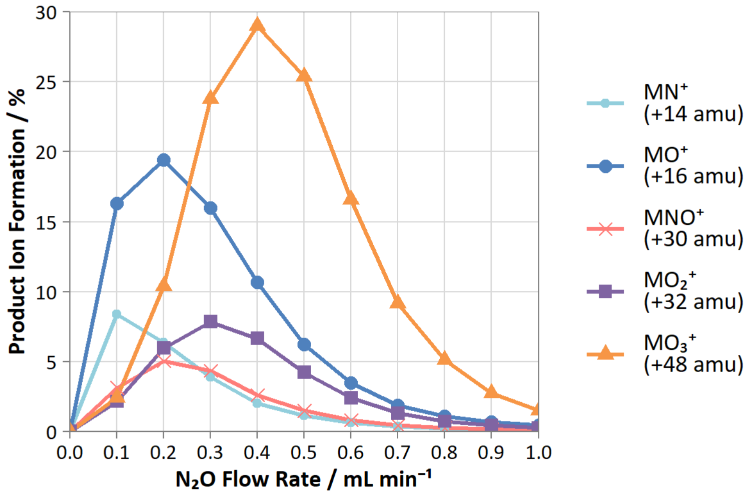

Major Product Ions of $^{184}W^+$ with O_2 Reaction Gas

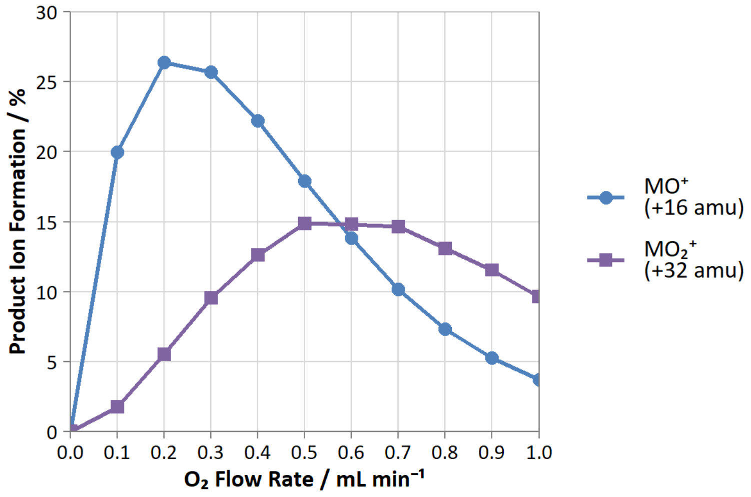

On-Mass with N_2O and O_2

On-Mass Profile of $^{184}W^+$ with N_2O and O_2

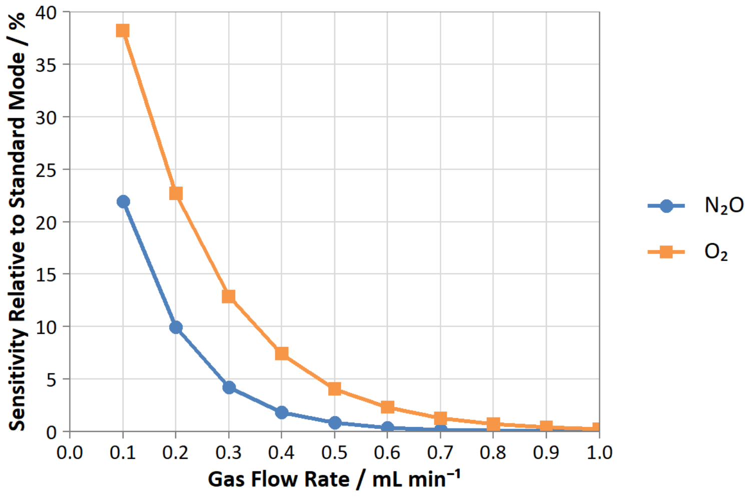

On-Mass Removal (as Interference) with N_2O

Variation of $^{184}W^{16}O^+$ Interference with N_2O Flow Rate (On-Mass: Q1 = 200, Q3 = 200)

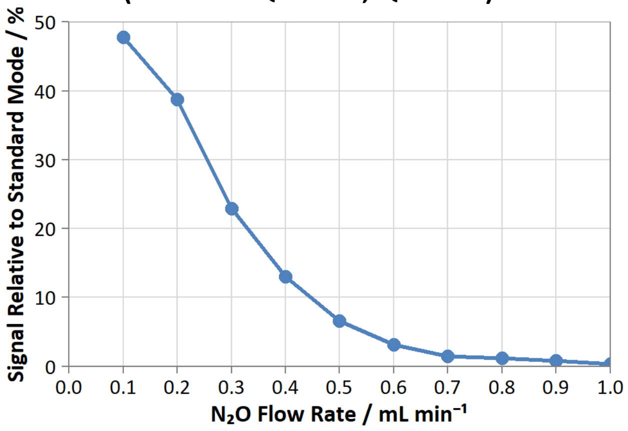

Variation of $^{184}W^{2+}$ Interference with N_2O Flow Rate (On-Mass: Q1 = 92, Q3 = 92)

[INSUFFICIENT DOUBLY-CHARGED ION FORMATION OBSERVED]

Asymmetric Charge Transfer (with N_2O)

[NO CHARGE TRANSFER REACTION OBSERVED]

Rhenium

Supplementary Information B to: Characterisation of gas cell reactions for 70+ elements using N_2O for ICP tandem mass spectrometry measurements
Shaun T. Lancaster[a]*, Thomas Prohaska[ab] and Johanna Irrgeher[ab]
[a]Department of General, Analytical and Physical Chemistry, Chair of General and Analytical Chemistry, Montanuniversität Leoben, Leoben, Austria.
[b]Department of Physics and Astronomy, University of Calgary, Calgary, Canada.

Mass-Shift with N_2O and O_2

Major Product Ions of $^{187}Re^+$ with N_2O Reaction Gas

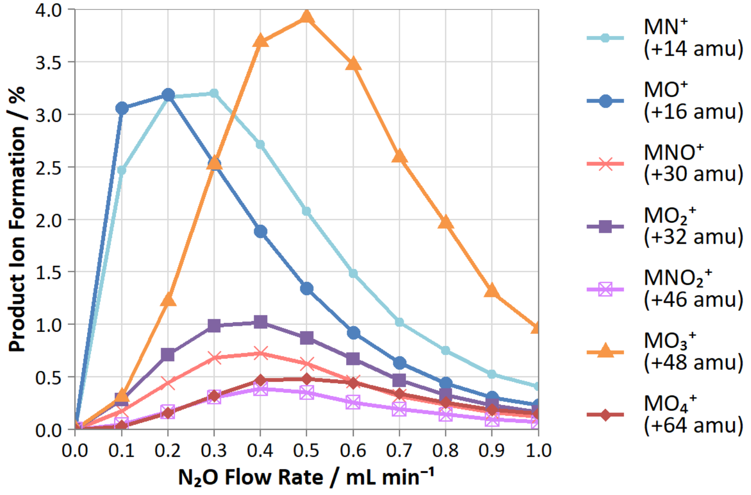

Major Product Ions of $^{187}Re^+$ with O_2 Reaction Gas

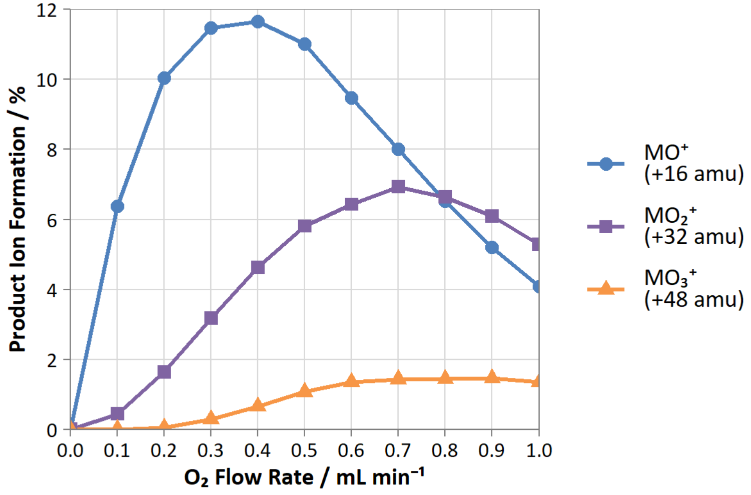

On-Mass with N_2O and O_2

On-Mass Profile of $^{187}Re^+$ with N_2O and O_2

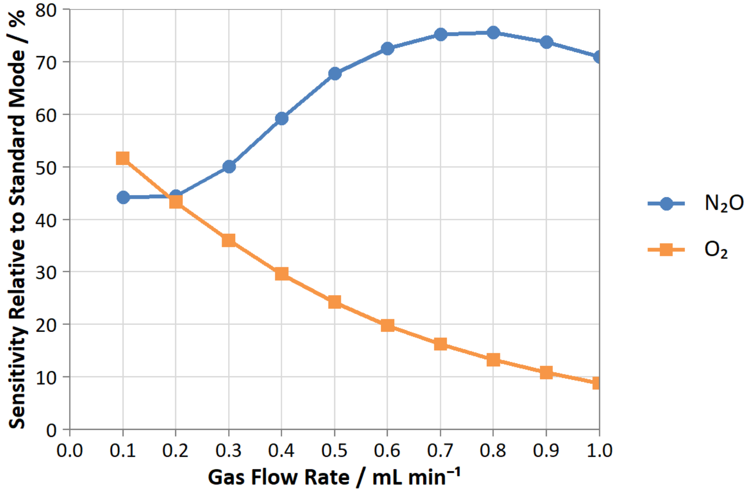

On-Mass Removal (as Interference) with N_2O

Variation of $^{187}Re^{16}O^+$ Interference with N_2O Flow Rate (On-Mass: Q1 = 203, Q3 = 203)

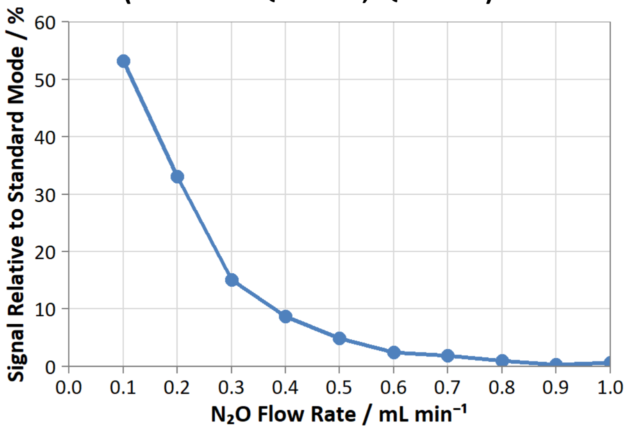

Variation of $^{187}Re^{2+}$ Interference with N_2O Flow Rate (On-Mass: Q1 = 93.5, Q3 = 93.5)

[INSUFFICIENT DOUBLY-CHARGED ION FORMATION OBSERVED]

Asymmetric Charge Transfer (with N_2O)

[NO CHARGE TRANSFER REACTION OBSERVED]

Osmium

Supplementary Information B to: Characterisation of gas cell reactions for 70+ elements using N_2O for ICP tandem mass spectrometry measurements
Shaun T. Lancaster[a]*, Thomas Prohaska[ab] and Johanna Irrgeher[ab]
[a]Department of General, Analytical and Physical Chemistry, Chair of General and Analytical Chemistry, Montanuniversität Leoben, Leoben, Austria.
[b]Department of Physics and Astronomy, University of Calgary, Calgary, Canada.

Mass-Shift with N_2O and O_2

Major Product Ions of $^{189}Os^+$ with N_2O Reaction Gas

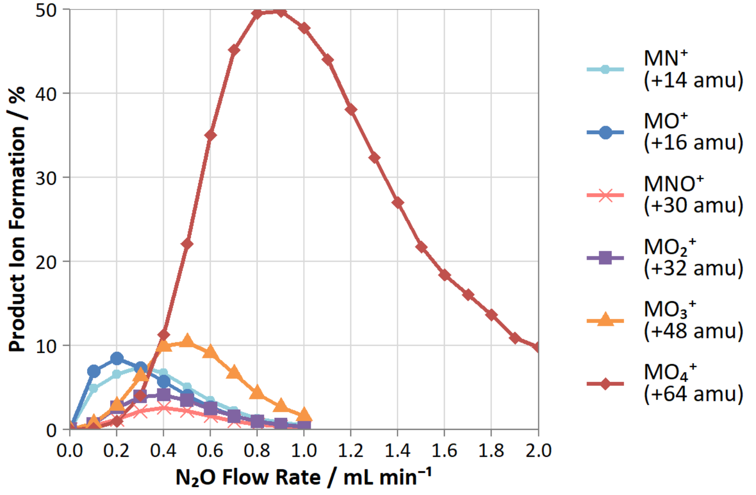

Major Product Ions of $^{189}Os^+$ with O_2 Reaction Gas

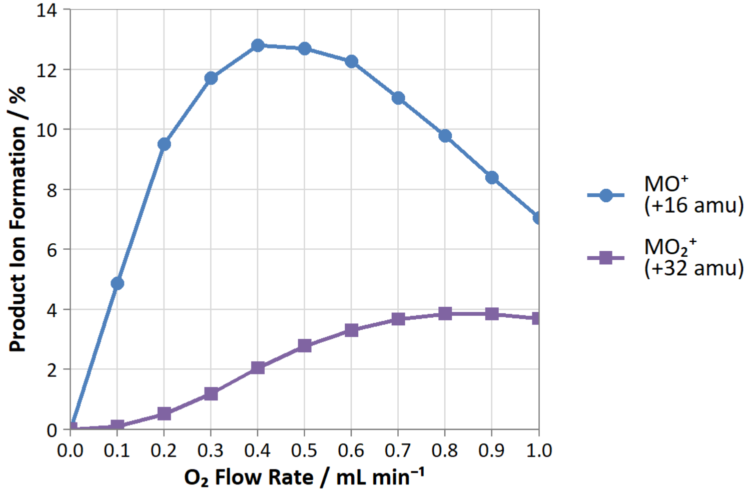

On-Mass with N_2O and O_2

On-Mass Profile of $^{189}Os^+$ with N_2O and O_2

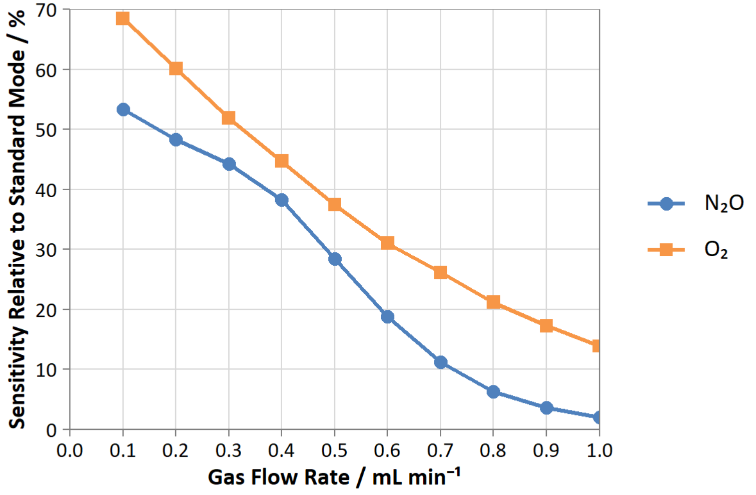

On-Mass Removal (as Interference) with N_2O

Variation of $^{189}Os^{16}O^+$ Interference with N_2O Flow Rate (On-Mass: Q1 = 205, Q3 = 205)

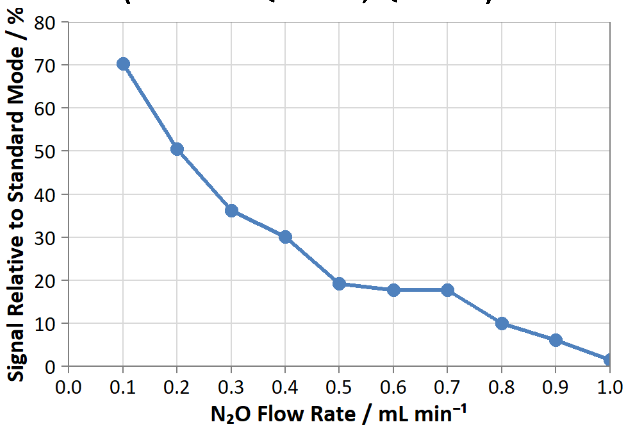

Variation of $^{189}Os^{2+}$ Interference with N_2O Flow Rate (On-Mass: Q1 = 94.5, Q3 = 94.5)

[INSUFFICIENT DOUBLY-CHARGED ION FORMATION OBSERVED]

Asymmetric Charge Transfer (with N_2O)

[NO CHARGE TRANSFER REACTION OBSERVED]

Iridium

Supplementary Information B to: Characterisation of gas cell reactions for 70+ elements using N_2O for ICP tandem mass spectrometry measurements
Shaun T. Lancaster[a*], Thomas Prohaska[ab] and Johanna Irrgeher[ab]
[a]Department of General, Analytical and Physical Chemistry, Chair of General and Analytical Chemistry, Montanuniversität Leoben, Leoben, Austria.
[b]Department of Physics and Astronomy, University of Calgary, Calgary, Canada.

Mass-Shift with N_2O and O_2

Major Product Ions of $^{193}Ir^+$ with N_2O Reaction Gas

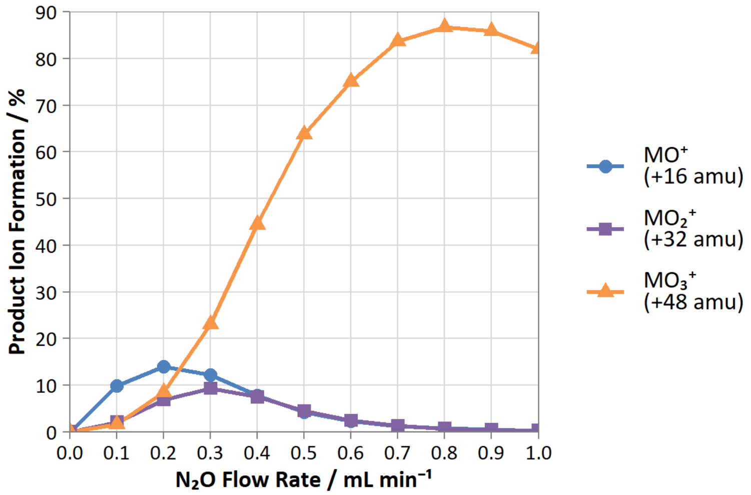

Major Product Ions of $^{193}Ir^+$ with O_2 Reaction Gas

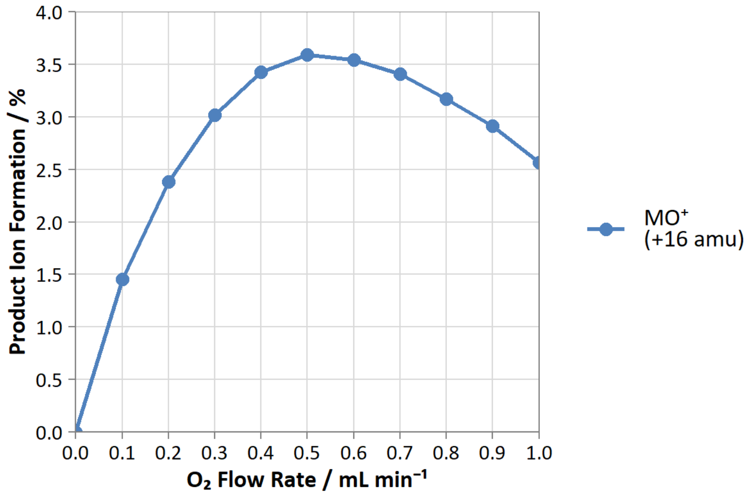

On-Mass with N_2O and O_2

On-Mass Profile of $^{193}Ir^+$ with N_2O and O_2

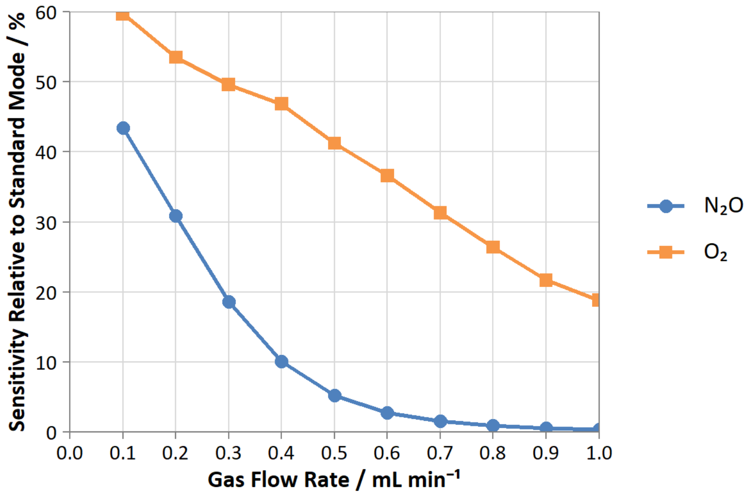

On-Mass Removal (as Interference) with N_2O

Variation of $^{193}Ir^{16}O^+$ Interference with N_2O Flow Rate (On-Mass: Q1 = 209, Q3 = 209)

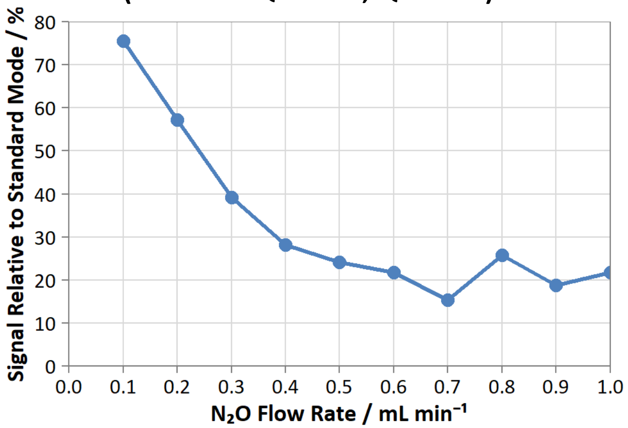

Variation of $^{193}Ir^{2+}$ Interference with N_2O Flow Rate (On-Mass: Q1 = 96.5, Q3 = 96.5)

[INSUFFICIENT DOUBLY-CHARGED ION FORMATION OBSERVED]

Asymmetric Charge Transfer (with N_2O)

[NO CHARGE TRANSFER REACTION OBSERVED]

Platinum

Supplementary Information B to: Characterisation of gas cell reactions for 70+ elements using N_2O for ICP tandem mass spectrometry measurements
Shaun T. Lancaster[a]*, Thomas Prohaska[ab] and Johanna Irrgeher[ab]
[a]Department of General, Analytical and Physical Chemistry, Chair of General and Analytical Chemistry, Montanuniversität Leoben, Leoben, Austria.
[b]Department of Physics and Astronomy, University of Calgary, Calgary, Canada.

Mass-Shift with N_2O and O_2

Major Product Ions of $^{195}Pt^+$ with N_2O Reaction Gas

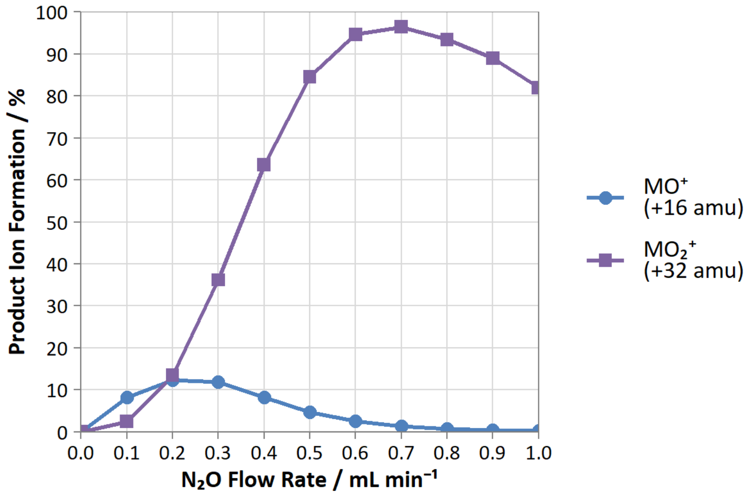

Major Product Ions of $^{195}Pt^+$ with O_2 Reaction Gas

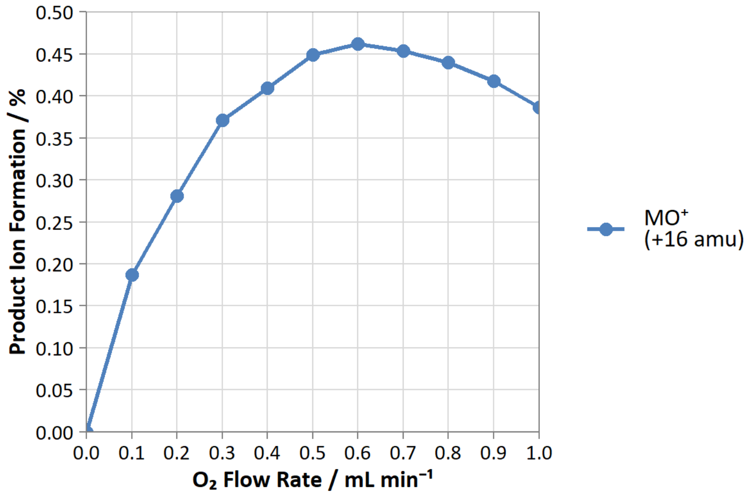

On-Mass with N_2O and O_2

On-Mass Profile of $^{195}Pt^+$ with N_2O and O_2

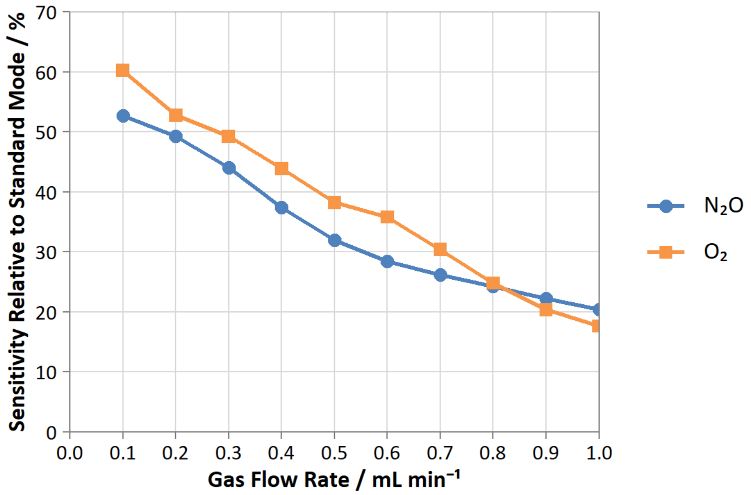

On-Mass Removal (as Interference) with N_2O

Variation of $^{195}Pt^{16}O^+$ Interference with N_2O Flow Rate (On-Mass: Q1 = 211, Q3 = 211)

[INSUFFICIENT OXIDE FORMATION OBSERVED]

Variation of $^{195}Pt^{2+}$ Interference with N_2O Flow Rate (On-Mass: Q1 = 97.5, Q3 = 97.5)

[INSUFFICIENT DOUBLY-CHARGED ION FORMATION OBSERVED]

Asymmetric Charge Transfer (with N_2O)

[NO CHARGE TRANSFER REACTION OBSERVED]

Gold

Shaun T. Lancaster[a]*, Thomas Prohaska[ab] and Johanna Irrgeher[ab]
[a]Department of General, Analytical and Physical Chemistry, Chair of General and Analytical Chemistry, Montanuniversität Leoben, Leoben, Austria.
[b]Department of Physics and Astronomy, University of Calgary, Calgary, Canada.

Mass-Shift with N_2O and O_2

Major Product Ions of $^{197}Au^+$ with N_2O Reaction Gas

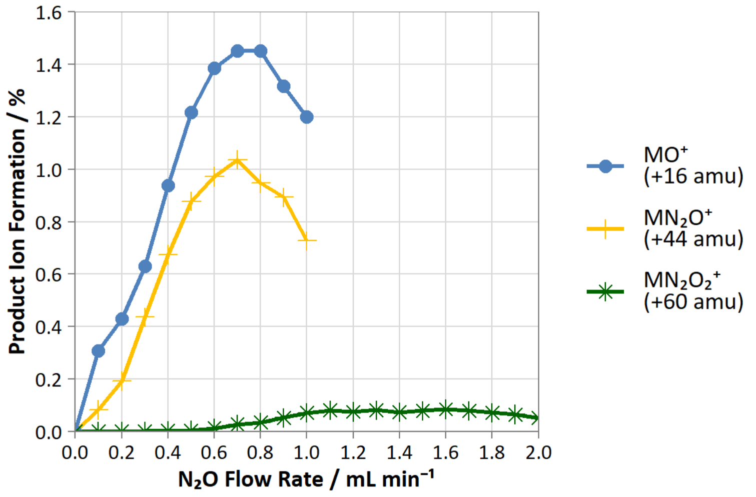

Major Product Ions of $^{197}Au^+$ with O_2 Reaction Gas

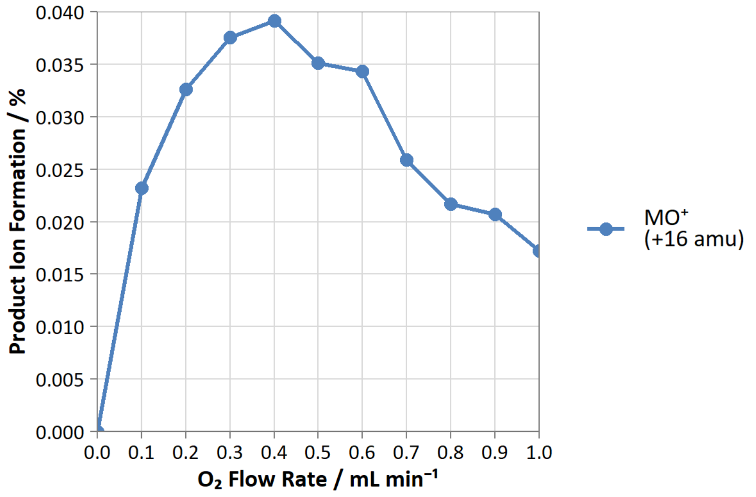

On-Mass with N_2O and O_2

On-Mass Profile of $^{197}Au^+$ with N_2O and O_2

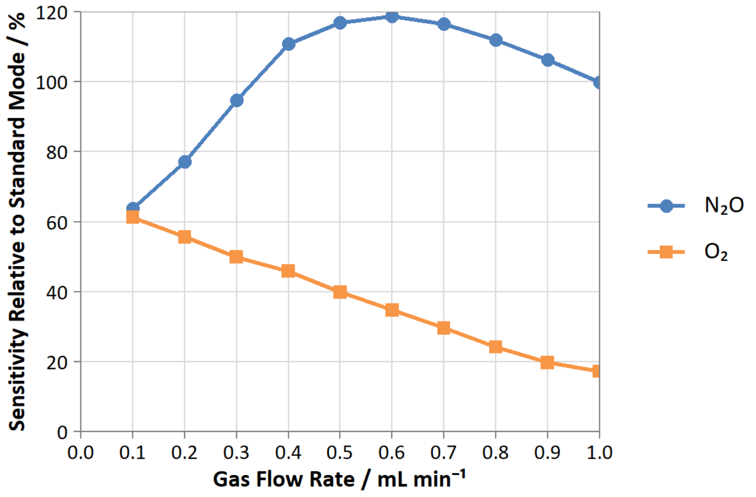

On-Mass Removal (as Interference) with N_2O

Variation of $^{197}Au^{16}O^+$ Interference with N_2O Flow Rate (On-Mass: Q1 = 213, Q3 = 213)

[INSUFFICIENT OXIDE FORMATION OBSERVED]

Variation of $^{197}Au^{2+}$ Interference with N_2O Flow Rate (On-Mass: Q1 = 98.5, Q3 = 98.5)

[INSUFFICIENT DOUBLY-CHARGED ION FORMATION OBSERVED]

Asymmetric Charge Transfer (with N_2O)

[NO CHARGE TRANSFER REACTION OBSERVED]

Mercury

Supplementary Information B to: Characterisation of gas cell reactions for 70+ elements using N_2O for ICP tandem mass spectrometry measurements
Shaun T. Lancaster[a]*, Thomas Prohaska[ab] and Johanna Irrgeher[ab]
[a]Department of General, Analytical and Physical Chemistry, Chair of General and Analytical Chemistry, Montanuniversität Leoben, Leoben, Austria.
[b]Department of Physics and Astronomy, University of Calgary, Calgary, Canada.

Mass-Shift with N_2O and O_2

Major Product Ions of $^{202}Hg^+$ with N_2O Reaction Gas

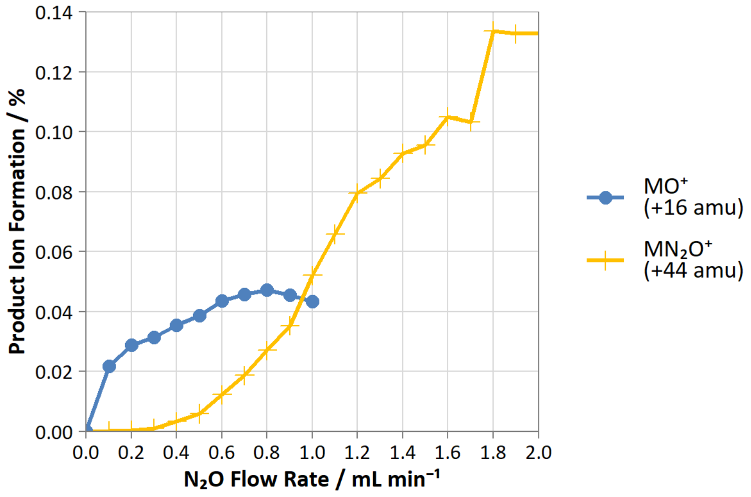

Major Product Ions of $^{202}Hg^+$ with O_2 Reaction Gas

[NO ATOM TRANSFER REACTION OBSERVED]

On-Mass with N_2O and O_2

On-Mass Profile of $^{202}Hg^+$ with N_2O and O_2

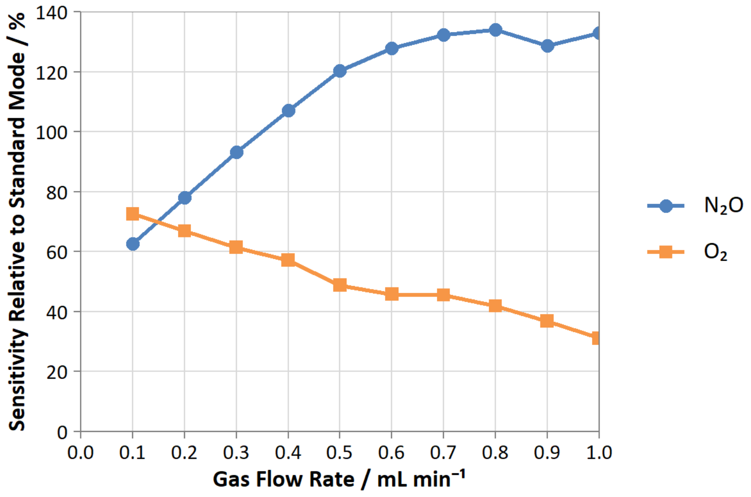

On-Mass Removal (as Interference) with N_2O

Variation of $^{202}Hg^{16}O^+$ Interference with N_2O Flow Rate (On-Mass: Q1 = 218, Q3 = 218)

[INSUFFICIENT OXIDE FORMATION OBSERVED]

Variation of $^{202}Hg^{2+}$ Interference with N_2O Flow Rate (On-Mass: Q1 = 101, Q3 = 101)

[INSUFFICIENT DOUBLY-CHARGED ION FORMATION OBSERVED]

Asymmetric Charge Transfer (with N_2O)

[NO CHARGE TRANSFER REACTION OBSERVED]

Thallium

Shaun T. Lancaster[a]*, Thomas Prohaska[ab] and Johanna Irrgeher[ab]
[a]Department of General, Analytical and Physical Chemistry, Chair of General and Analytical Chemistry, Montanuniversität Leoben, Leoben, Austria.
[b]Department of Physics and Astronomy, University of Calgary, Calgary, Canada.

Mass-Shift with N_2O and O_2

Major Product Ions of $^{205}Tl^+$ with N_2O Reaction Gas

[NO ATOM TRANSFER REACTION OBSERVED]

Major Product Ions of $^{205}Tl^+$ with O_2 Reaction Gas

[NO ATOM TRANSFER REACTION OBSERVED]

On-Mass with N_2O and O_2

On-Mass Profile of $^{205}Tl^+$ with N_2O and O_2

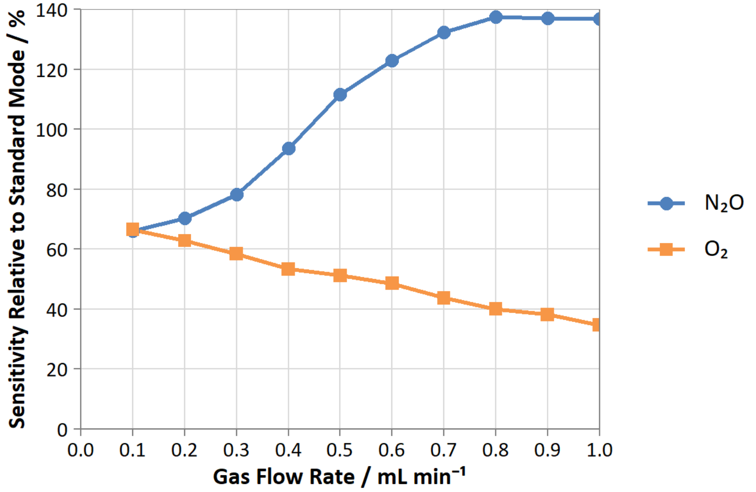

On-Mass Removal (as Interference) with N_2O

Variation of $^{205}Tl^{16}O^+$ Interference with N_2O Flow Rate (On-Mass: Q1 = 221, Q3 = 221)

[INSUFFICIENT OXIDE FORMATION OBSERVED]

Variation of $^{205}Tl^{2+}$ Interference with N_2O Flow Rate (On-Mass: Q1 = 102.5, Q3 = 102.5)

[INSUFFICIENT DOUBLY-CHARGED ION FORMATION OBSERVED]

Asymmetric Charge Transfer (with N_2O)

[NO CHARGE TRANSFER REACTION OBSERVED]

Lead

Supplementary Information B to: Characterisation of gas cell reactions for 70+ elements using N_2O for ICP tandem mass spectrometry measurements
Shaun T. Lancaster[a]*, Thomas Prohaska[ab] and Johanna Irrgeher[ab]
[a]Department of General, Analytical and Physical Chemistry, Chair of General and Analytical Chemistry, Montanuniversität Leoben, Leoben, Austria.
[b]Department of Physics and Astronomy, University of Calgary, Calgary, Canada.

Mass-Shift with N_2O and O_2

Major Product Ions of $^{208}Pb^+$ with N_2O Reaction Gas

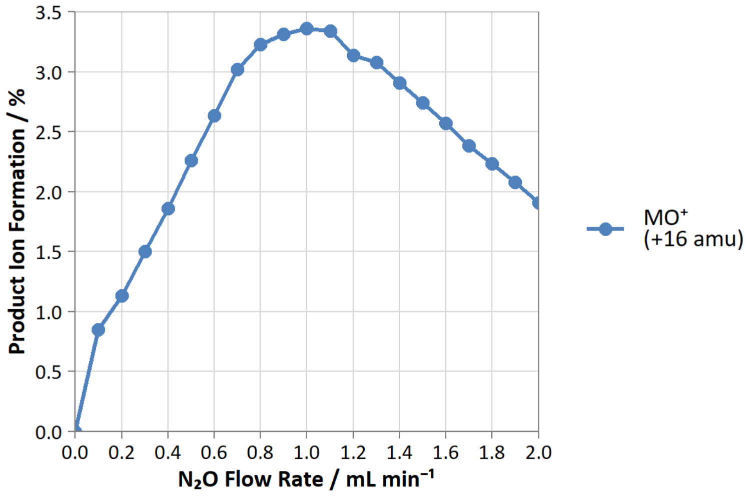

Major Product Ions of $^{208}Pb^+$ with O_2 Reaction Gas

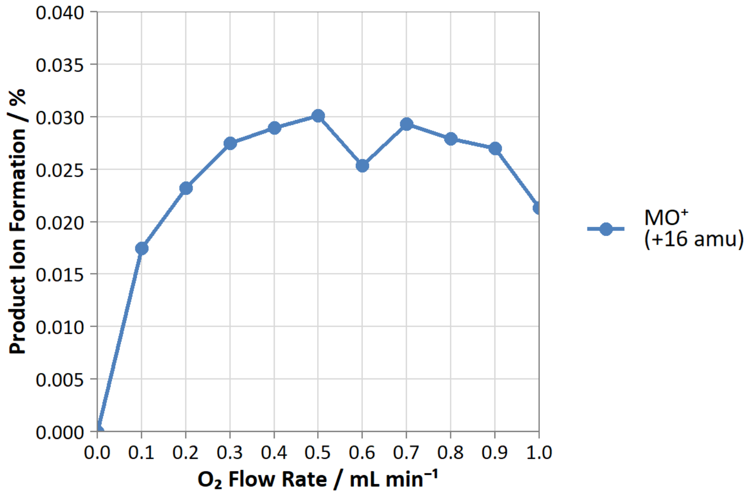

On-Mass with N_2O and O_2

On-Mass Profile of $^{208}Pb^+$ with N_2O and O_2

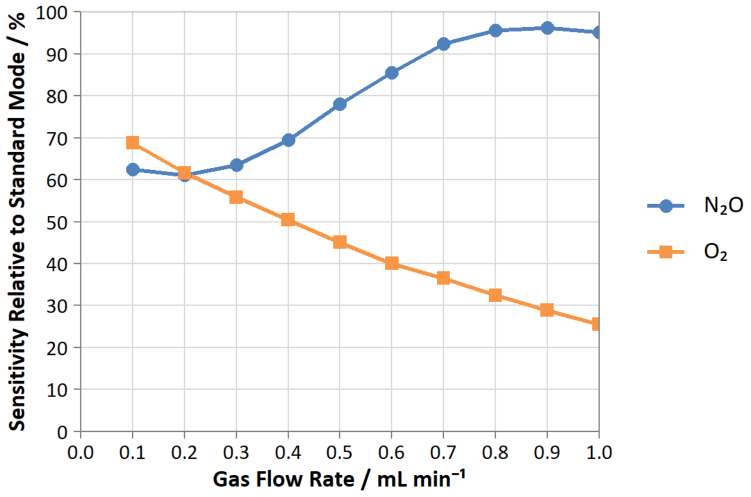

On-Mass Removal (as Interference) with N_2O

Variation of $^{208}Pb^{16}O^+$ Interference with N_2O Flow Rate (On-Mass: Q1 = 224, Q3 = 224)

[INSUFFICIENT OXIDE FORMATION OBSERVED]

Variation of $^{208}Pb^{2+}$ Interference with N_2O Flow Rate (On-Mass: Q1 = 104, Q3 = 104)

[INSUFFICIENT DOUBLY-CHARGED ION FORMATION OBSERVED]

Asymmetric Charge Transfer (with N_2O)

[NO CHARGE TRANSFER REACTION OBSERVED]

Bismuth

Supplementary Information B to: Characterisation of gas cell reactions for 70+ elements using N_2O for ICP tandem mass spectrometry measurements
Shaun T. Lancaster[a]*, Thomas Prohaska[ab] and Johanna Irrgeher[ab]
[a]Department of General, Analytical and Physical Chemistry, Chair of General and Analytical Chemistry, Montanuniversität Leoben, Leoben, Austria.
[b]Department of Physics and Astronomy, University of Calgary, Calgary, Canada.

Mass-Shift with N_2O and O_2

Major Product Ions of $^{209}Bi^+$ with N_2O Reaction Gas

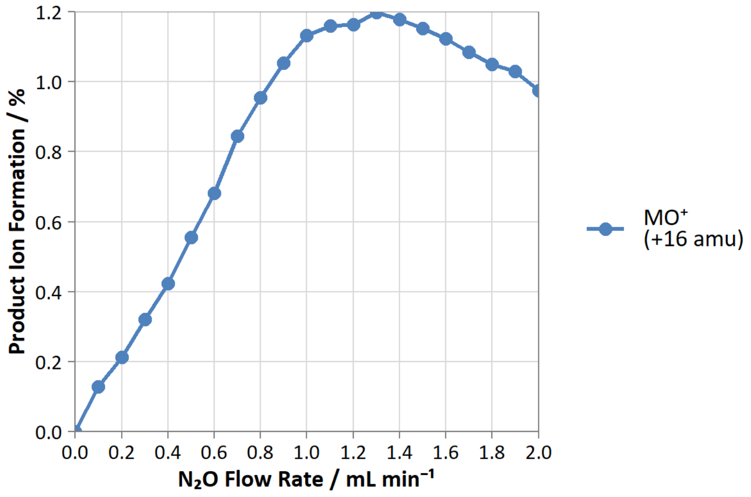

Major Product Ions of $^{209}Bi^+$ with O_2 Reaction Gas

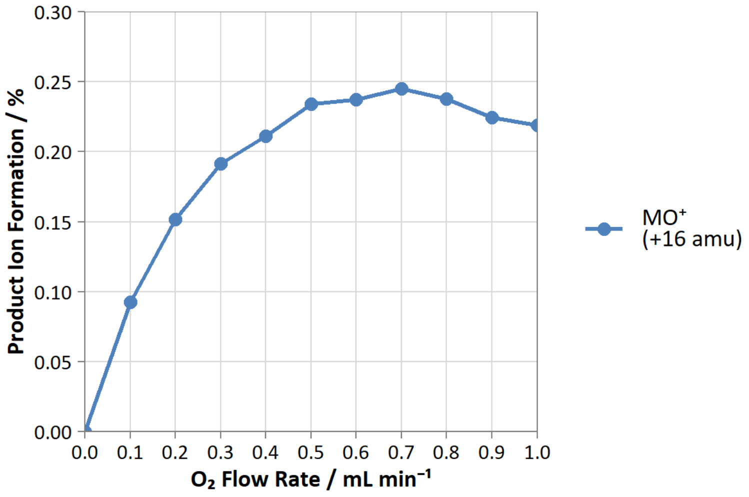

On-Mass with N_2O and O_2

On-Mass Profile of $^{209}Bi^+$ with N_2O and O_2

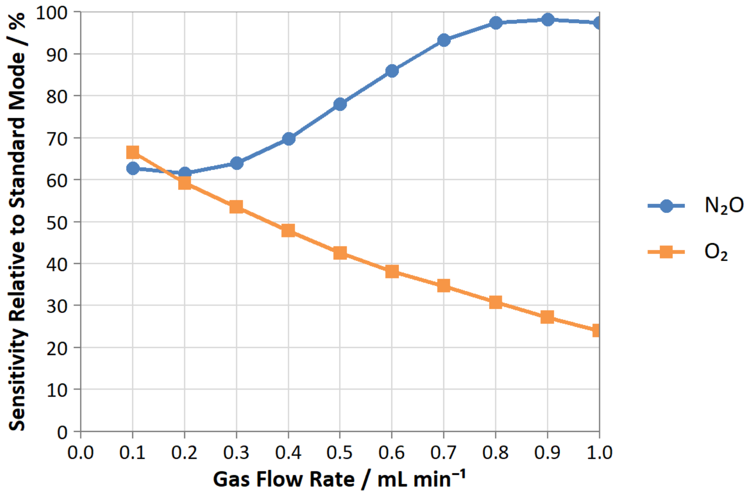

On-Mass Removal (as Interference) with N_2O

Variation of $^{209}Bi^{16}O^+$ Interference with N_2O Flow Rate (On-Mass: Q1 = 225, Q3 = 225)

[INSUFFICIENT OXIDE FORMATION OBSERVED]

Variation of $^{209}Bi^{2+}$ Interference with N_2O Flow Rate (On-Mass: Q1 = 104.5, Q3 = 104.5)

[INSUFFICIENT DOUBLY-CHARGED ION FORMATION OBSERVED]

Asymmetric Charge Transfer (with N_2O)

[NO CHARGE TRANSFER REACTION OBSERVED]

Thorium

Supplementary Information B to: Characterisation of gas cell reactions for 70+ elements using N_2O for ICP tandem mass spectrometry measurements
Shaun T. Lancaster[a]*, Thomas Prohaska[ab] and Johanna Irrgeher[ab]
[a]Department of General, Analytical and Physical Chemistry, Chair of General and Analytical Chemistry, Montanuniversität Leoben, Leoben, Austria.
[b]Department of Physics and Astronomy, University of Calgary, Calgary, Canada.

Mass-Shift with N_2O and O_2

Major Product Ions of $^{232}Th^+$ with N_2O Reaction Gas

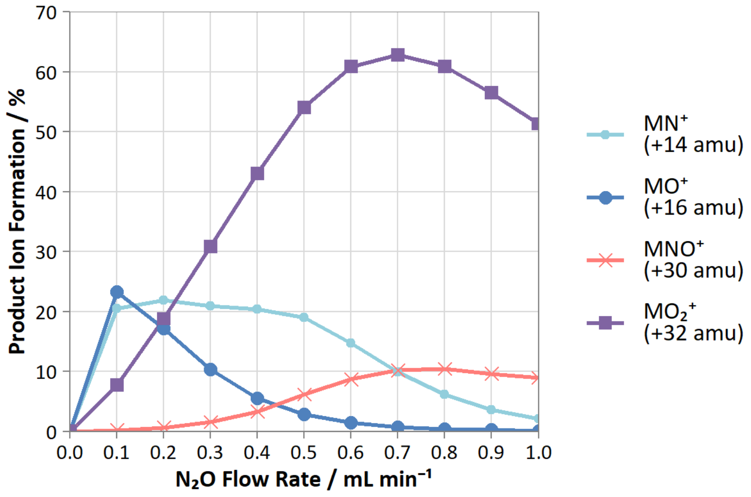

Major Product Ions of $^{232}Th^+$ with O_2 Reaction Gas

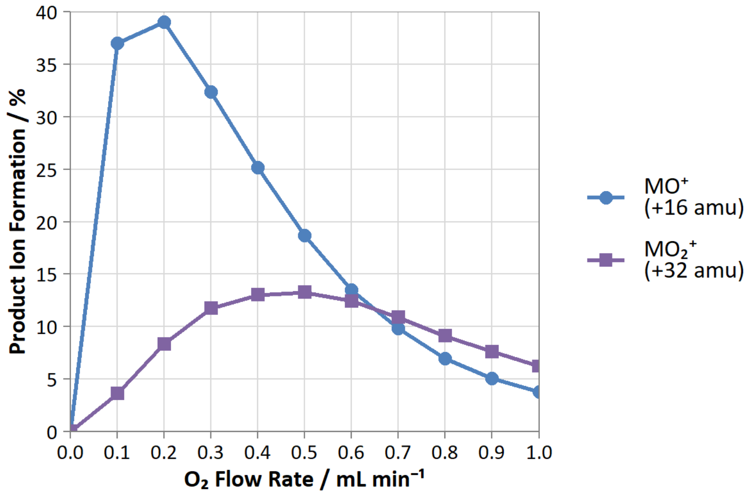

On-Mass with N_2O and O_2

On-Mass Profile of $^{232}Th^+$ with N_2O and O_2

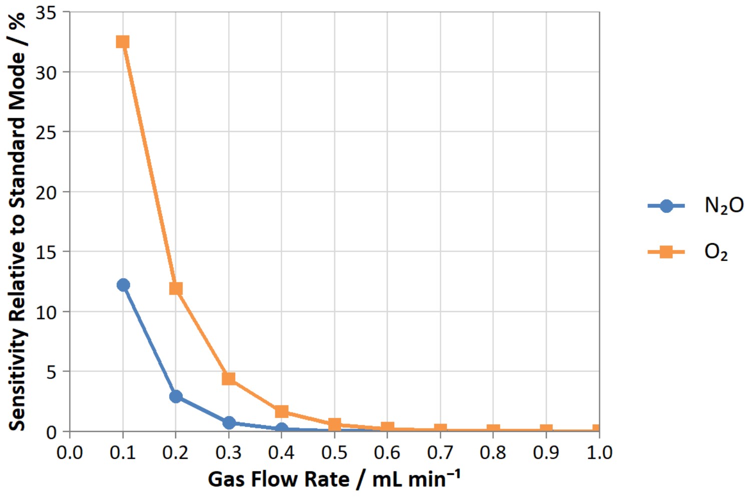

On-Mass Removal (as Interference) with N_2O

Variation of $^{232}Th^{16}O^+$ Interference with N_2O Flow Rate (On-Mass: Q1 = 248, Q3 = 248)

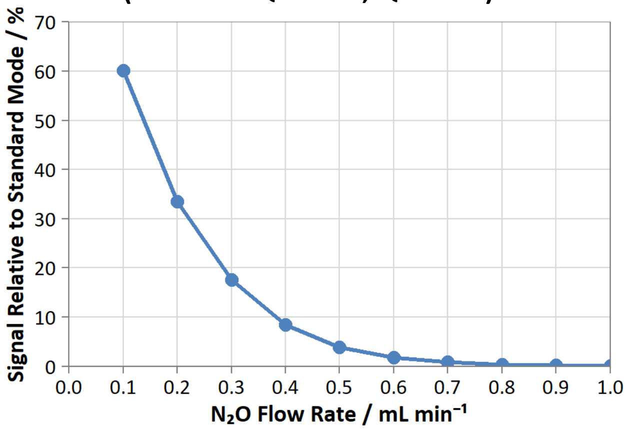

Variation of $^{232}Th^{2+}$ Interference with N_2O Flow Rate (On-Mass: Q1 = 116, Q3 = 116)

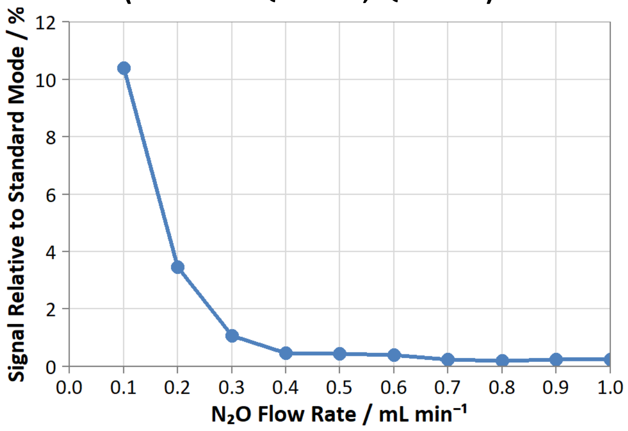

Asymmetric Charge Transfer (with N_2O)

[NO CHARGE TRANSFER REACTION OBSERVED]

Uranium

Supplementary Information B to: Characterisation of gas cell reactions for 70+ elements using N_2O for ICP tandem mass spectrometry measurements
Shaun T. Lancaster[a]*, Thomas Prohaska[ab] and Johanna Irrgeher[ab]
[a]Department of General, Analytical and Physical Chemistry, Chair of General and Analytical Chemistry, Montanuniversität Leoben, Leoben, Austria.
[b]Department of Physics and Astronomy, University of Calgary, Calgary, Canada.

Mass-Shift with N_2O and O_2

Major Product Ions of $^{238}U^+$ with N_2O Reaction Gas

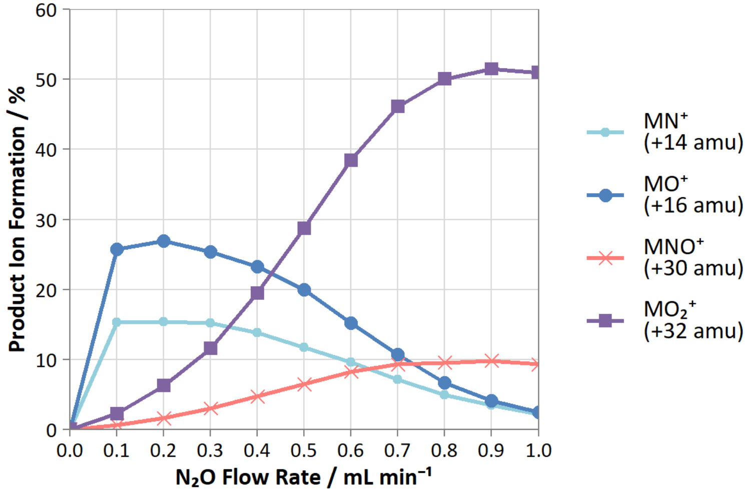

Major Product Ions of $^{238}U^+$ with O_2 Reaction Gas

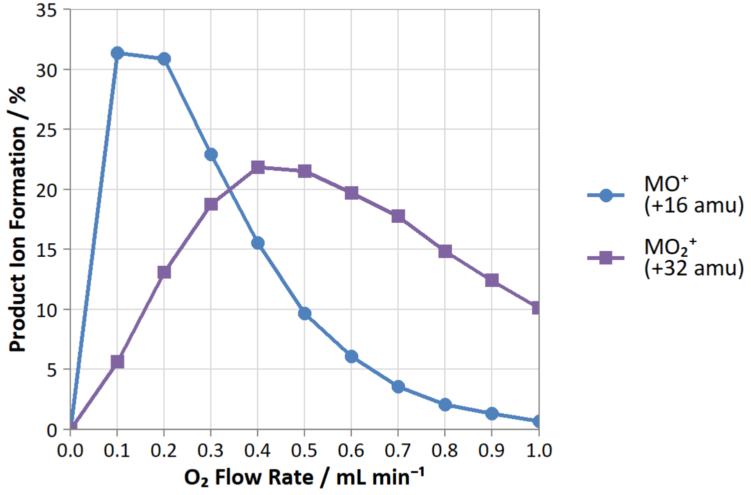

On-Mass with N_2O and O_2

On-Mass Profile of $^{238}U^+$ with N_2O and O_2

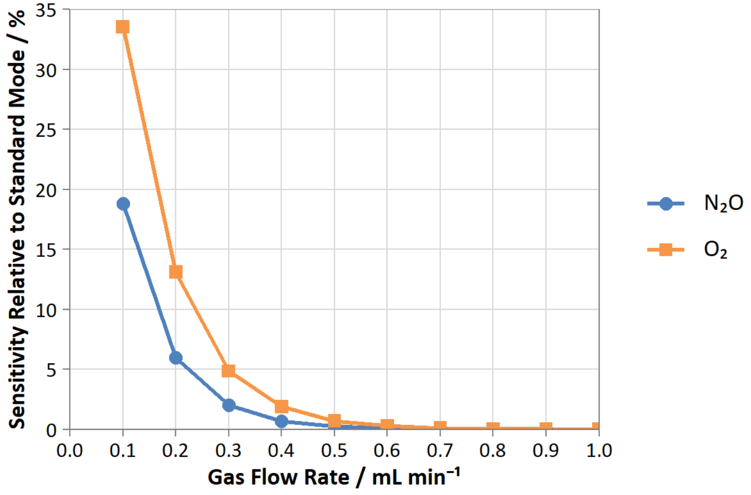

On-Mass Removal (as Interference) with N_2O

Variation of $^{238}U^{16}O^+$ Interference with N_2O Flow Rate (On-Mass: Q1 = 254, Q3 = 254)

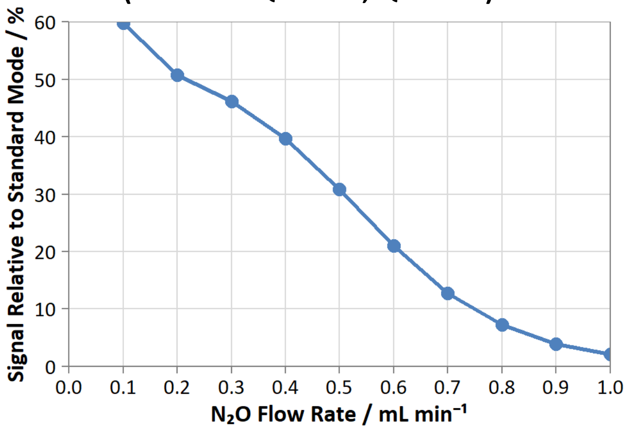

Variation of $^{238}U^{2+}$ Interference with N_2O Flow Rate (On-Mass: Q1 = 119, Q3 = 119)

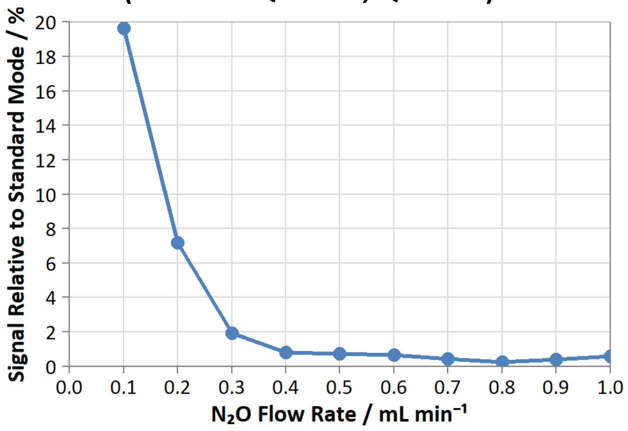

Asymmetric Charge Transfer (with N_2O)

[NO CHARGE TRANSFER REACTION OBSERVED]
